# iROS-gPseKNC: Predicting replication origin sites in DNA by incorporating dinucleotide position-specific propensity into general pseudo nucleotide composition

**DOI:** 10.18632/oncotarget.9057

**Published:** 2016-04-27

**Authors:** Xuan Xiao, Han-Xiao Ye, Zi Liu, Jian-Hua Jia, Kuo-Chen Chou

**Affiliations:** ^1^ Computer Department, Jing-De-Zhen Ceramic Institute, Jing-De-Zhen, 333403, China; ^2^ Information School, ZheJiang Textile and Fashion College, NingBo, 315211, China; ^3^ School of Computer Science and Engineering, Nanjing University of Science and Technology, Nanjing, 210094, China; ^4^ Center of Excellence in Genomic Medicine Research (CEGMR), King Abdulaziz University, Jeddah, 21589, Saudi Arabia; ^5^ Gordon Life Science Institute, Boston, Massachusetts, 02478, USA

**Keywords:** origin of replication, position-specific dinucleotide propensity, general pseudo nucleotide composition, random forest, iROS-gPseKNC

## Abstract

DNA replication, occurring in all living organisms and being the basis for biological inheritance, is the process of producing two identical replicas from one original DNA molecule. To in-depth understand such an important biological process and use it for developing new strategy against genetics diseases, the knowledge of duplication origin sites in DNA is indispensible. With the explosive growth of DNA sequences emerging in the postgenomic age, it is highly desired to develop high throughput tools to identify these regions purely based on the sequence information alone. In this paper, by incorporating the dinucleotide position-specific propensity information into the general pseudo nucleotide composition and using the random forest classifier, a new predictor called iROS-gPseKNC was proposed. Rigorously cross–validations have indicated that the proposed predictor is significantly better than the best existing method in sensitivity, specificity, overall accuracy, and stability. Furthermore, a user-friendly web-server for iROS-gPseKNC has been established at http://www.jci-bioinfo.cn/iROS-gPseKNC, by which users can easily get their desired results without the need to bother the complicated mathematics, which were presented just for the integrity of the methodology itself.

## INTRODUCTION

**Figure 1:**
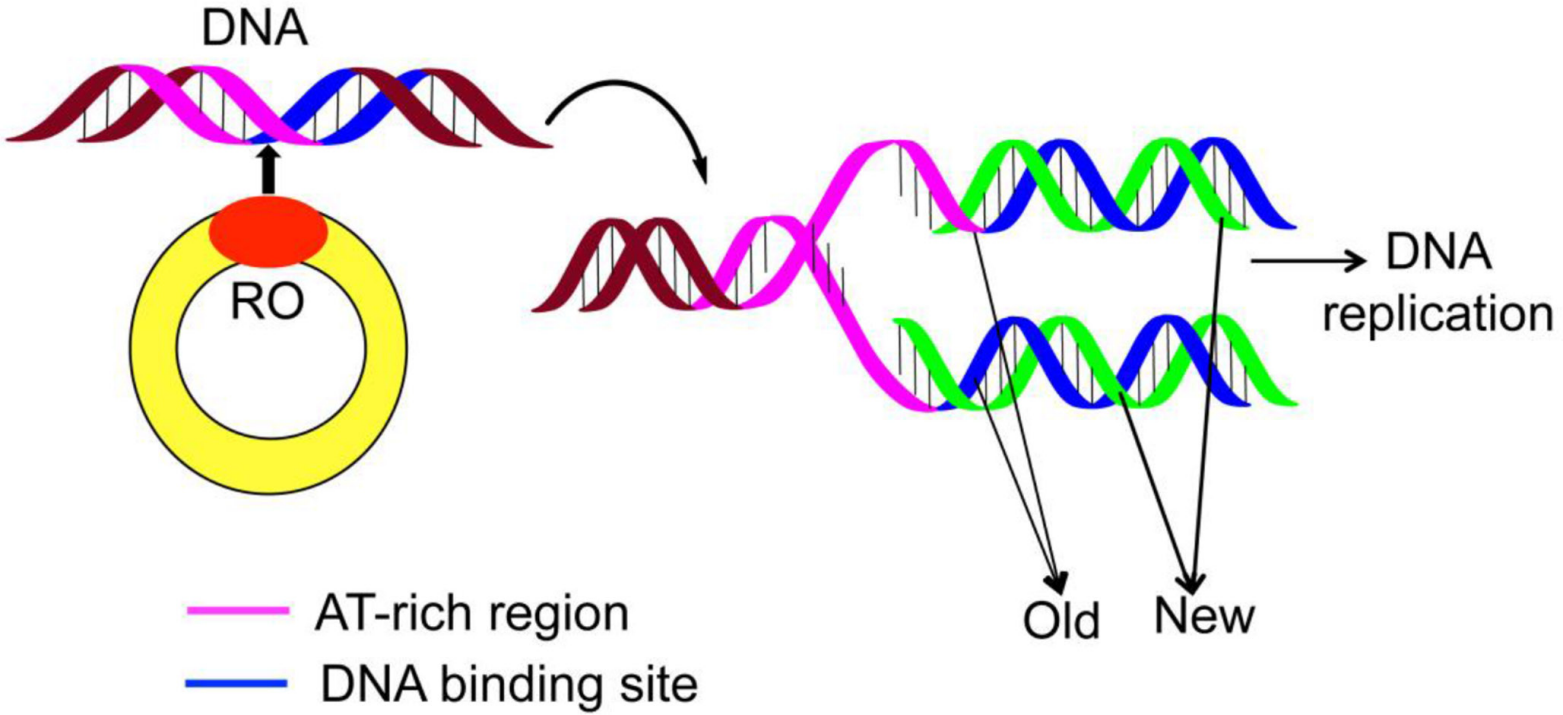
A schematic drawing to show the DNA replication origin (RO)

During the cell-replicating process, the genome duplication is an indispensable step. Although the processes of DNA replications are different for bacteria, archaea, and eukaryotes, they all share the same core components as elaborated in [1–2]. For in-depth understanding the genome duplication, it is important to find the “origin of replication region” (Ori), or “replication origin” (RO) (Figure 1).

For small DNAs, such as those in bacterial plasmids and small viruses, a single origin would be sufficient to ensure a complete and opportune replication for each cell cycle in the entire genome. It is quite different, however, for eukaryotic genomes that contain substantially more origins [2–3]. Actually, it is quite natural to establish the replication forks at multiple locations [3] in order for timely duplicating their larger linear chromosomes. Therefore, to in-depth understand the process of cell reproduction, it is fundamentally important to acquire the RO information [1].

There are many experimental methods that can be used to determine the RO sites, such as chromatin immunoprecipitation (Chip), ChIp sequencing, and surface plasmon resonance (SPR). But it would take much longer time and spend more money to purely use experimental methods alone to acquire this kind of information. Therefore, it would be wise to develop computational methods to do the job, or at least as a complementary tool to the traditional experimental approach.

Actually, many scientists have endeavored to do so, as reported in a series of publications [2–12]. Unfortunately, all these reported methods have some limitations, such as in limited accuracy and practical application value. Particularly, most of these methods are without a web-server, and can hardly be used by most experimental scientists. In view of this, further work in such an important and urgent area is definitely needed.

According to Chou's five guidelines [13] and many recent publications [14–20], to develop a sequence-based statistical predictor useful not only for theoretical scientists but also broad experimental scientists, we should observe the following five guidelines and make their concrete processes crystal clear: (1) how to prepare benchmark dataset; (2) how to formulate the biological sequence samples; (3) how to operate the prediction engine; (4) how to validate the predictor's results; (5) how to provide a publically accessible web-server for the predictor. In the rest of this paper, we are to address these five aspects one-by-one. To fit in the style of the Oncotarget journal, however, their order may be subject to some sort of change.

## RESULTS AND DISCUSSION

### A new predictor with its web-server and user guide

A new and much more accurate sequence-based method, called iROS-gPseKNC, was developed for predicting replication origin sites in DNA. Moreover, to attract most experimental scientists and maximize their convenience [11, 21], the server of iROS-gPseKNC has been established along with its instructions, as given below.

**Figure 2:**
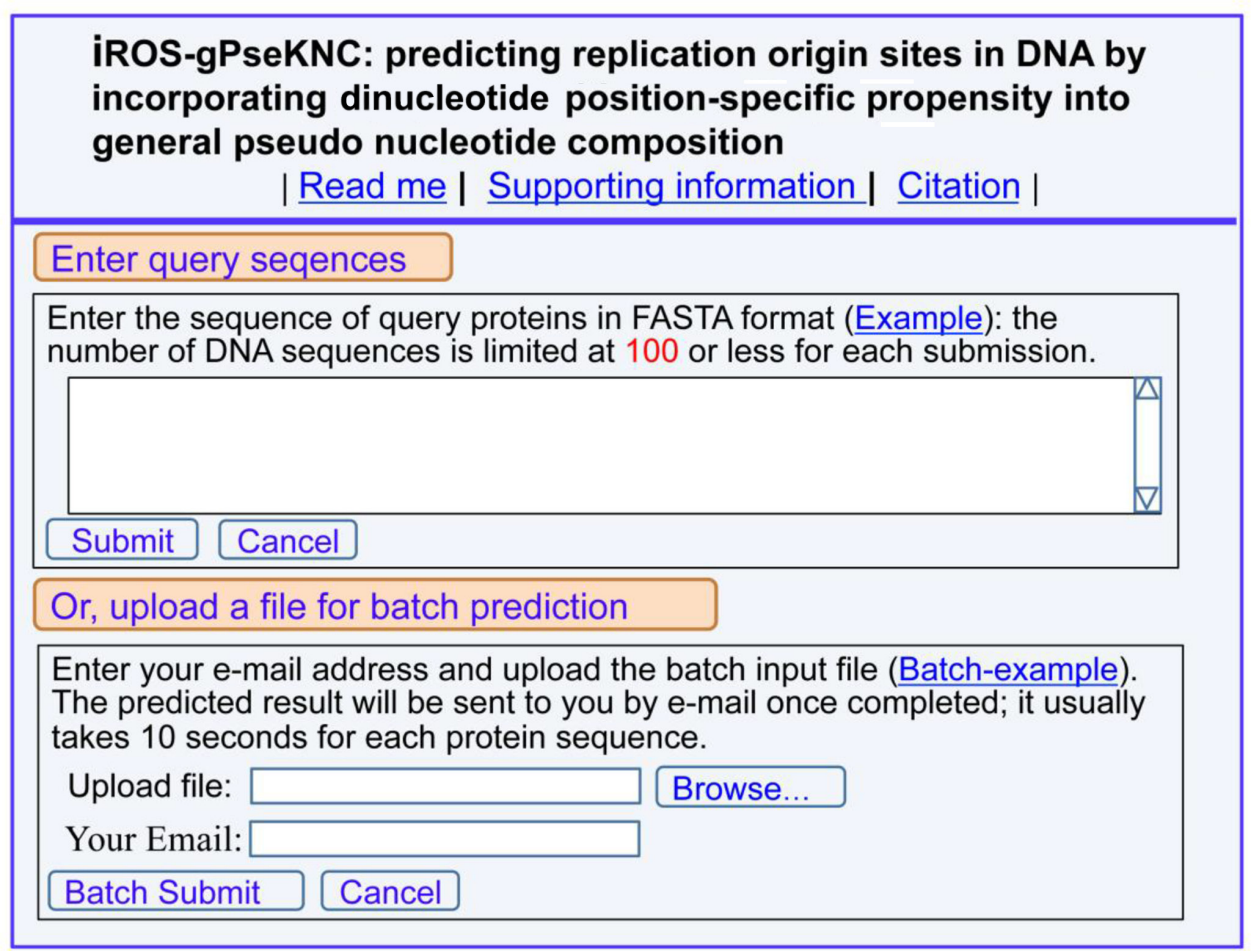
A semi-screenshot for the top page of the web-server iROS-gPseKNC at http://www.jci-bioinfo.cn/iROS-gPseKNC

(1) Click the web-server at http://www.jci-bioinfo.cn/iROS-gPseKNC, the top page of the iROS-gPseKNC will be prompted on your computer screen (Figure 2).

(2) Enter your query DNA sequences into the central input box (Figure 2) by using either typing or copying/pasting operation. The entered query sequences should be in the FASTA format. If you are not familiar with it, please click the Example button nearby.

(3) You can see the prediction results by clicking the Submit button. For example, if your query DNA sequences are none but those listed in the Example window, the following results will be shown on the screen: (1) DNA region 1 is the replication origin site; (2) DNA region 2 is non-replication origin site. All these outcomes were confirmed by experiments.

(4) If you have a lot of query sequences and need much longer computational time, you are also allowed to use the batch prediction. To do this, just use the Browse button to select the desired file (in FASTA format of course) and follow the online instruction.

(5) The benchmark dataset used in this study is available by clicking the button of Supporting Information on the top of Figure 2.

(6) To see the papers relevant to the development of this server, just click on the button of Citation.

### Result analysis and comparison

The success scores achieved by iROS-gPseKNC on the benchmark dataset (Supporting Information S1) by the jackknife tests are given in Table 1. Shown in that table are also the corresponding scores obtained by the existing methods. It can be seen from Table 1 that iROS-gPseKNC achieved remarkably higher scores than its counterparts in all the four metrics, clearly indicating that, compared with its counterparts, the proposed predictor has the highest sensitivity, specificity, overall accuracy, and stability.

**Table 1:** A comparison of the proposed predictor with the existing methods via the jackknife tests on a same benchmark dataset of Supporting Information S1

Predictor	Sn (%)^d^	Sp (%)^d^	Acc (%)^d^	MCC^d^
BC-based^a^	81.23	80.30	80.76	61.53
iORI-PseKNC^b^	84.69	82.76	83.72	67.46
iROS-gPseKNC^c^	**96.42**	**99.74**	**98.03**	**96.11**

a The prediction method developed by Chen [4].

b The prediction method developed by Li et al. [12]} that was deemed the most powerful one among the existing methods for the same purpose.

c The prediction method proposed in this paper.

d See Eq.7 for the definition of the metrics.

Why could the proposed method yield so high success rates? It is not easy to give a simple and intuitive answer for this problem. Fortunately, many biological systems and the complicated relations therein could be revealed via the intuitive graphical approaches (see, e.g. [22–31]).

**Figure 3:**
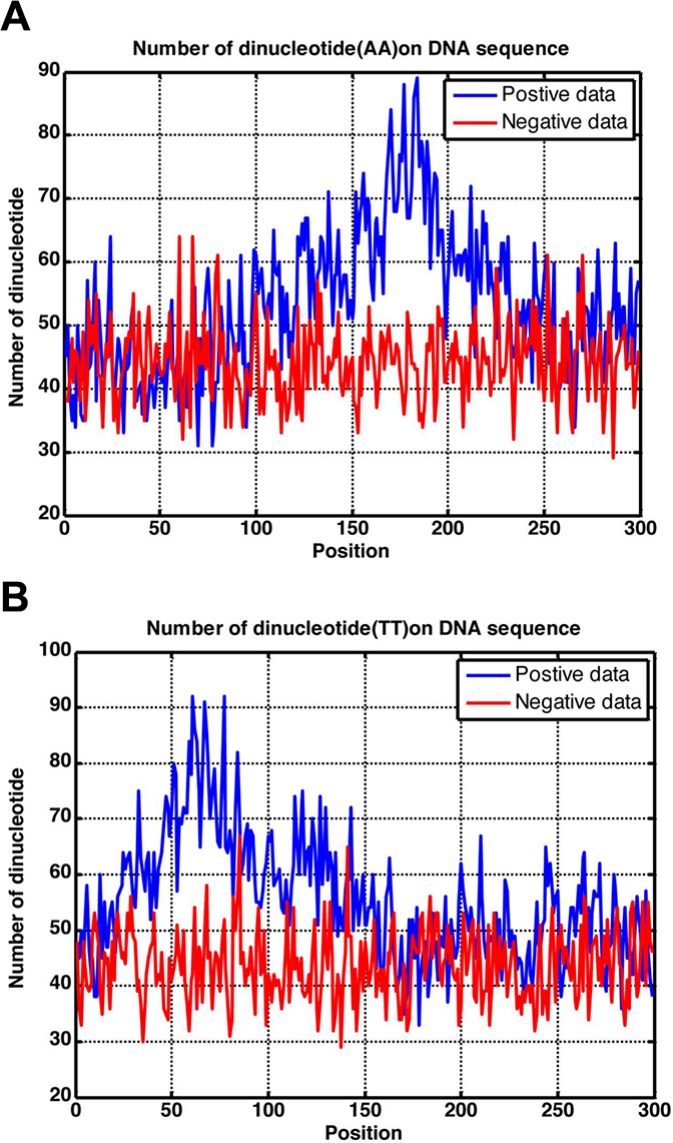
Graph to show the statistical distribution of the dinucleotide occurrence frequency for (**A**) AA and (**B**) TT along the 300 bp region. See the text for further explanation

In this study, using the intuitive graphic method, we obtained various statistical distributions for different dinucleotide occurrence frequencies along the 300 bp region as shown in Figure 3, where panel (A) is for dinucleotide AA, and panel (B) for dinucleotide TT. Of course, we could draw a total of 16 such panels, but two are more than enough to make the point clear. It can be seen from Figure 3A that the AA profile for the positive samples (blue) is remarkably different from that for the negative samples (red). The same is true for the two TT profiles as shown in Figure 3B. Consequently, it is self-evident why the proposed method, which was established by including the dinucleotide position-specific propensity with the general PseKNC (see Material and Methods section), is so successful.

**Figure 4:**
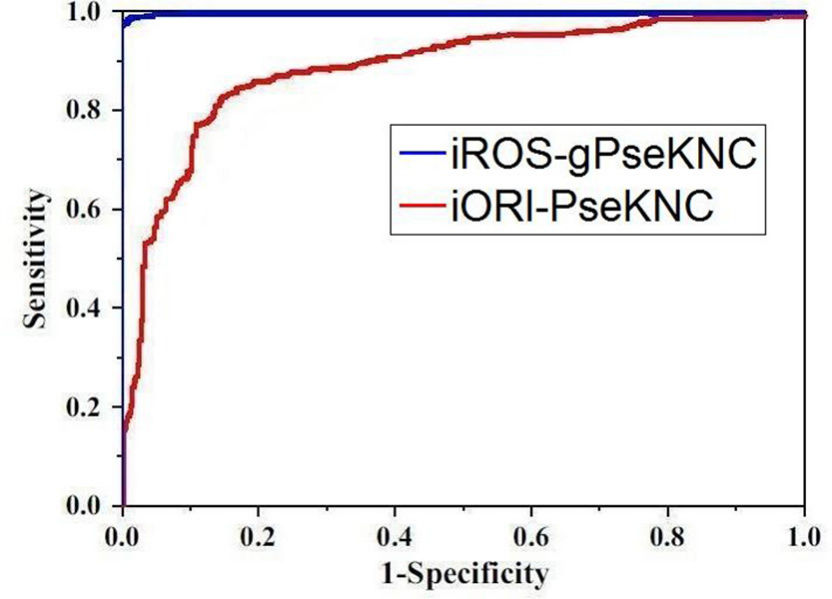
Graph to show the ROC curve [32, 33] The one with red is for iORI-PseKNC predictor [12]}; while the one with blue is for the proposed predictor iROS-gPseKNC. The area under the blue curve is remarkably larger than that under the red curve. See the text for further explanation.

To provide an intuitive comparison of the proposed predictor with its counterpart, the graph of ROC (receiver operating characteristic) [32, 33] was adopted as shown in Figure 4, where the ROC curves for the iROS-gPseKNC and iORI-PseKNC [12] are in blue and red, respectively. The greater the AUC (area under the ROC curve) value is, the better the corresponding predictor will be [32, 33]. It can be easily seen from Figure 4 that the area under the blur curve is substantially greater than that under the red one, clearly indicating that the proposed predictor is no doubt superior to iORI-PseKNC [12], the best existing predictor for identifying the origins of replication in DNA sequences. Accordingly, we anticipate that iROS-gPseKNC will become a very useful computational tool for predicting DNA RO sites.

## MATERIALS AND METHODS

### Benchmark dataset

In this study, we used the same dataset recently constructed by Li et al. [12] that was specialized for studying the replication origin sites. The reasons are as follows. (1) The dataset was constructed rigorously based on experiment-confirmed reports only, and hence is more reliable. (2) None of samples included had pairwise sequence identity to any other, and hence the dataset is more stringent in excluding homology bias than the other relevant ones. (3) Most important, it will facilitate the comparison of our new prediction method with the existing ones since a fair comparison should be based on a same benchmark dataset and same cross-validation approach.

In literature, the benchmark dataset usually consists of a training dataset and a testing dataset: the former is constructed for the purpose of training a proposed model, while the latter for the purpose of testing it. As pointed out by a comprehensive review [34], however, there is no need to separate a benchmark dataset into a training dataset and a testing dataset for validating a prediction method if it is tested by the jackknife or subsampling (K-fold) cross-validation because the outcome thus obtained is actually from a combination of many different independent dataset tests. Thus, the benchmark dataset taken from Li et al. [12] for the current study can be formulated as

$$\text{S}=\;{\text{S}}^{+}\;⋃{\text{S}}^{-}$$ (1)

where the positive subset S^+^ contains 405 replication origin samples, the negative subset S^−^ contains 406 non-replication origin samples, and the symbol ⋃ denotes the union in the set theory. The 405 + 406 = 811 DNA samples are each consist of 300 bp [12], as can be generally formulated by

$$\text{D}=-\;{\text{N}}_{1}{\text{N}}_{2}{\text{N}}_{3}\cdots {\text{N}}_{i}\cdots {\text{N}}_{300}$$ (2)

For readers' convenience, their sequences are given in Supporting Information S1.

### Feature vector construction

Biology is a natural science with historic dimension. All biological species have developed beginning from a very limited number of ancestral species. It is true for the biological sequences as well. Their evolution involves changes of single amino acid or nucleic acid residues, insertions and deletions of several residues, gene doubling, and gene fusion. With these changes accumulated for a long period of time, many apparent similarities between the initial and resultant biological sequences have been gradually disappearing, but the corresponding sequences may still share some essential common features. That is why the 3D (three-dimensional) structure of a protein derived from the template [35] of a remote homologous protein [36] is often quite successful although their sequence similarity may not be high [37, 38]. Also, it has been reported that the bacterial replication origins share similar nucleotide sequence motifs. Therefore, the key is how to “unearth” this kind of motifs deeply “buried” in extremely complicated DNA sequences.

Actually, with the avalanche of biological sequences generated in the post-genomic age, one of the most challenging problems in computational biology is how to formulate a biological sequence with a discrete model or vector, yet still considerably keep its sequence pattern or order information. This is because almost all the existing machine-learning algorithms were developed to handle vector but not sequence samples, as elaborated in [21]. But a vector defined in a discrete model may completely lose this kind of sequence-pattern information. To overcome this problem, the “pseudo amino acid composition” [39] or Chou's PseAAC [40, 41] was developed to deal with protein/peptide sequences. Encouraged by its successes in computational proteomics, the idea of PseAAC was recently extended to dealing with DNA/RNA sequences in many important problems of genome analysis [12, 16, 18, 42–47] by introducing the pseudo nucleotide composition or PseKNC [9, 10, 14, 48, 49].

According to a recent review paper [11], the general form of PseKNC for a DNA sequence can be formulated as

$$\text{D }={\left[\phi_{1\;}\phi_{2}\cdots \phi_{\text{u}}\cdots \phi_{z}\right]}^{T}$$ (3)

where T is the transpose operator, while *Z* an integer to reflect the vector's dimension. The value of Z as well as the components ϕ_u__*u*_ (*u* = 1, 2, …, *z)* in Eq.3 will depend on how to extract the desired information from the DNA sequence.

Recently, by incorporating the dipeptide position-specific propensity into the general PseAAC [13], Xu et al. developed two predictors for identifying posttranslational modification (PTM) sites for proteins: one for cysteine S-nitrosylation sites [50], and the other for hydroxyproline and hydroxylysine sites [51]. Stimulating by their approach, here we are to develop a new method for predicting the replication origin sites by incorporating the dinucleotide position-specific propensity into the general PseKNC [11] or Eq.3.

There are 4^2^ = 16 dinucleotides: AA, AC, AG, AT, CA, CC, CG, CT, GA, GC, GG, GT, TA, TC, TG, and TT. Thus, for a DNA sample with 300 bp (Eq.2) as given in Supporting Information S1, its profile (or detailed information) of the dinucleotide position-specific propensity can be summarized by the following 16 × 299 matrix:

$$\text{D}=\left[\begin{array}{cccc} P_{1,1} & P_{1,2} & \cdots & P_{1,299}\\ P_{2,1} & P_{2,2} & \cdots & P_{2,299}\\ \vdots & \vdots & \cdots & \vdots \\ P_{16,1} & P_{16,2} & \cdots & P_{16,299} \end{array}\right]$$ (4)

where

$$P_{i,j\;}=Q+(2meri|j)-Q^{-}(2meri|j)\;(i=1,\;2,\;\cdots ,\;16)\;(j=\;1,\;2,\;\cdots ,\;299)$$ (5)

In the above equation, 2mer_1_ = AA, 2mer_2_ = AC, 2mer_3_ = AG, 2mer_4_ = AT, 2mer_15_ = TG, 2mer_16_ = TT, and Q^+^ (2mer_*i*_ |*j)* is the occurrence frequency of the *i*-th dinucleotide (2mer_*i*_) at the *j*-th subsite on the sequence of Eq.2 that can be easily derived from the positive dataset S^+^, while Q^−^ (2mer_*i*_ |*j)* is the corresponding occurrence frequency, but from the negative dataset S^−^.

Thus, the DNA sample of Eq.2 can be uniquely defined via the general form of PseKNC (cf. Eq.3) with its dimension *Z* = 299 and its *u*-th component given by

$$\phi_{u}=\{\begin{array}{llll} P_{1,u} & when & N_{u} & {\text{N}}_{u+1}=\text{AA}\\ P_{2,u} & when & N_{u} & {\text{N}}_{u+1}=\text{AC}\\ P_{3,u} & when & N_{u} & {\text{N}}_{u+1}=\text{AG}(1\leq u\leq 299)\\ \vdots & & & \vdots \\ P_{16,u} & when & N_{u} & {\text{N}}_{u+1}=\text{TT} \end{array}$$ (6)

### Random forest classifier

The random forests (RF) algorithm is a powerful algorithm and has been used in many areas of computational biology (see, e.g. [52–56]). The essence of BF is to randomly generate many trees by the recursive partitioning approach, followed by aggregating the results. Its detailed procedures and formulation have been very clearly described in [57], and hence there is no need to repeat here.

After training by the relevant benchmark dataset, the RF classifier can quickly indicate which attribute an input query sample belongs to. For the current study, the input are DNA sequences, while the output are which of them belong to the replication origins and which of them do not.

The predictor obtained via the aforementioned procedures is called iROS-gPseKNC, where “i” stands for “identify”, “ROS” for “replication origin site”, and “gPseKNC” for “general PseKNC” approach.

As pointed out in the beginning of this paper, in developing a new predictor it is very important to clearly report how to evaluate its anticipated success rates [13]. To realize this, let us consider the following two things: one is what metrics we should use to quantitatively measure the predictor's quality; the other is what kind of test approach we should adopt to calculate the metrics rates.

### A set of four metrics for measuring prediction quality

In statistical prediction, four metrics were often used to measure the quality of a predictor; they are: (1) overall accuracy or Acc; (2) Mathew's correlation coefficient or MCC; (3) sensitivity or Sn; and (4) specificity or Sp [58]. But their conventional formulations are not quite intuitive, and most experimental scientists feel difficult to understand them, particularly for the MCC metrics. Fortunately, if using the formulation introduced by Chou [59] in studying the signal peptides, the set of four metrics can be equivalently defined as follows [60, 61]:

$$\{\begin{array}{ll} \text{Sn}=1-\frac{N_{-}^{+}}{N^{+}} & 0\leq \text{Sn}\leq 1\\ \text{Sp}=1-\frac{N_{+}^{-}}{N^{-}} & 0\leq \text{Sp}\leq 1\\ \text{Acc}=\wedge =1-\frac{N_{-}^{+}+N_{+}^{-}}{N^{+}+N^{-}} & 0\leq \text{Acc}\leq 1\\ \text{MCC}=\frac{1-\left(\frac{N_{-}^{+}}{N^{+}}+\frac{N_{+}^{-}}{N^{-}}\right)}{\sqrt{\left(1+\frac{N_{+}^{-}-N_{-}^{+}}{N^{+}}\right)\left(1+\frac{N_{-}^{+}-N_{+}^{-}}{N^{-}}\right)}} & -1\leq \text{MCC}\leq 1 \end{array}$$ (7)

where *N^+^* stands for the total number of replication origin samples investigated, whereas $N_{+}^{-}$ for the number of replication origin samples incorrectly predicted to be of non-replication origin; *N*^−^ for the total number of non-replication origin samples investigated, whereas $N_{+}^{-}$ for the number of non-replication origin samples incorrectly predicted to be of replication origin. With such formulation as given in Eq.7, the meanings of sensitivity, specificity, overall accuracy, and Mathew's correlation coefficient and their rate scopes would become more intuitive and easier-to-understand, particularly for the Mathew's correlation coefficient, as concurred by many investigators in their recent publications [20, 55, 56, 60, 62–72]}[16, 20].

It is instructive to point out, however, the set of metrics in Eq.7 is valid only for the single-label systems. For the multi-label systems as emerging increasingly frequent in system biology [73–75] and system medicine [76], a completely different set of metrics is needed as elucidated in [77].

### Cross validation

With a set of well-defined metrics to measure the quality of a predictor, the next thing is what kind of validation method should be used to score these metrics.

In predictive analytics, the following three cross-validation methods are often used: (1) independent dataset test, (2) subsampling (or K-fold cross-validation) test, and (3) jackknife test [78]. Of these three, however, the jackknife test is deemed the least arbitrary that can always yield a unique outcome for a given benchmark dataset as elucidated in [13]. Accordingly, the jackknife test has been widely recognized and increasingly used by investigators to examine the quality of various predictors (see, e.g., [79, 80] [81–84]). Therefore, the jackknife test was also adopted in this study to score the metrics of Eq.7. In the jackknife test, each of the samples in the benchmark dataset is singled out one-by-one and tested by the predictor trained by the remaining samples. During the jackknifing process, both the training dataset and testing dataset are literally open, and each sample is in turn moved between the two. The jackknife test can exclude the “memory” effect; it can also avoid the arbitrariness problem occurring in the independent dataset test and subsampling test as pointed out in [13] because the outcome obtained by the jackknife test is always unique for a given benchmark dataset.

## CONCLUSIONS

DNA replication is one of the most important life processes at the cellular level. To really understand such vitally important biological process, the knowledge of duplication origin sites is fundamentally important. The iROS-gPseKNC predictor presented in this paper can be used to identify the duplication origin sites based on the DNA sequence information alone. Its accuracy is better than the best existing predictor in this area. By running the iROS-gPseKNC web-server according to its step-by-step guide, users can easily obtain their desired results without the need to go through the detailed mathematics, which were presented in this paper just for its integrity.

Although the new predictor can yield significantly higher success rates than the existing ones, there still are plenty rooms to further improve it from the following two angles. One is with the increase of experimental data available in future, the dataset used to train the current model can be further refined and its coverage scope being much wider, and hence the predictor will be even more powerful. The other one is that many studies [80, 85–94] have indicated a predictor formed by fusing an array of individual classifiers may significantly enhance the prediction power; we will try to develop an ensemble predictor in this regard by fusing an array of individual classifiers with each being based on different modes of PseAAC [13, 39, 95, 96].

## SUPPORTING INFORMATION

Supporting Information S1. The original benchmark dataset. It contains 811 DNA segments, of which 405 are ORIs or positive samples, and 406 are non-ORIs or negative samples, where the benchmark dataset was taken from Li et al. [12]. Each segment sample contains 300 nucleotide residues. None of the samples include here is identical to any other. See the main paper for further explanation.

## SUPPLEMENTARY MATERIALS TABLES


