# Supplementary material for: iROS-gPseKNC: Predicting replication origin sites in DNA by incorporating dinucleotide position-specific propensity into general pseudo nucleotide composition
Source: Oncotarget. 2016 Apr 27;7(23):34180–9. doi: 10.18632/oncotarget.9057 (PMC5085147; doi:10.18632/oncotarget.9057)
Supplement: Supplementary file 1 [file oncotarget-07-34180-s001.pdf]

iROS-gPseKNC: Predicting replication origin sites in DNA by incorporating dinucleotide position-specific propensity into general pseudo nucleotide composition

Supplementary Materials

Supporting Information S1: The benchmark dataset consists of 811 DNA samples, of which 405 are experiment-confirmed replication origins and 406 non-replication origins

I. Positive subset contains 405 replication origins with each having 300 bp.

>Positive Sample 1
CTGCCCTTAATACATACGTTATACCACTTTTGCACCATATACTTACCACTCCATTTATATACACTTATGT
CAATATTACAGAAAAATCCCCACAAAAATCACCTAAACATAAAAATATTCTACTTTTCAACAATAATACA
TAAACATATTGGCTTGTGGTAGCAACACTATCATGGTATCACTAACGTAAAAGTTCCTCAATATTGCAAT
TTGCTTGAACGGATGCTATTTCAGAATATTTCGTACTTACACAGGCCATACATTAGAATAATATGTCACA
TCACTGTCGTAACACTCTTT

>Positive Sample 2
GTTGATTAGTACTGTTGGTCTCTCATTGAAATTTTACGTGACACCATCATTTTACTTCCACATAAGTTCT
AATGTTACGTAGTTCAATTTTAGTCGACCTAGCTTCATATTTATTTTAGAAGCAATTCGTAATTATCATT
TTGCTTTCGAAGAAAATTAAGACTTCATTTACTATTCTCGTGATATTTTAGTAGGCGCTTCTTTTGTATC
GAACCATTTTATTGCAATGGCCCTTAAGTTACCGTTATTCATACCAATTTGACGTTAATTTTAAATGCGT
TCTGAAGTTTCTTAAATAAC

>Positive Sample 3
CAACCTAATTTCATCATCAGTTAAGAAAATAGAATAACAGTTGTATGGGTCACCTGGCAGGGAGTAAAAT
CCAATAAGGCCCACTACGACAGACACAATAGCGTCAATAATAAAGTTCCATCTCCATCCCTCTAAACCAT
TTACACCATTTAACGATGAATATACGGCTGACTGGATCCCACCAGCGGATAGAATACCGATATACTGGCC
CAAATAGTAAAAAGCAGAACGACGCACCATTTCATCATGTTTGTAAAAGGAACCAAACAAATATTGGTAT
GCCAAATAACTTGGCGCTTC

>Positive Sample 4
ACGCTTTCAAAGTTTTCTTCTATAAACATACTTGTGGCAGCTTGTTTTTTTTGTTTTATTTTTAAGTTTT
GTTGGGTCTCTGAGAACTTTCAAAAAAAGAAAAAGTAAAGTATGATAAAACGGAGCACTTGCCAAAGTAA
TTAACGCCCATTAAAAAGAAGGCATAGGAGGCATATACATATATATATATATATATATATATGGCTGCTG
ACAGATATTCTGCACTTAAAAACTAAAAATATTATACCAACTTTTCTTTTTCTTCCCGTTCAGTTTGCTT
GATTGGCCCAGCTCTTTGAA

>Positive Sample 5
AAAAAGCAGCAGTACTTTCATCATTACCTCATAATACTGTGCACACGGTTATAATATTTGGTGGTTACAG
ACAAACCGGTGATGATCGTTACGAAGCAATGAATGATTTGTGGAAGATAGAGATACCCGTGATACGTCGC
GGTAAAAAAGGTTATTGTAAGTTTTCAGAGACAGCTAACGCGATACTACTGACGCCAAGCGAAAAGGACA
AATCGGATTGGCCCGAAGAAAGAGCCTTTTCTGCCTTTTCTGTTCATGGGACTTCGTTAATGGATAGGAG
TTCTCTTGACATGAGACTAT

>Positive Sample 6
CAATGTTTTATCTACGTTGGAGTAAGATCGTTTATCACTTCCATATTTGGACCAAATGAAAAGTTCAATC
GGCCAAGTATTTCATGGATGGAATGACGTTTGGTAAGGAAGTGCTTTTTCTTTTTCCACATATTTTCCCT
TTCTCTCGGGGAAATTTTGTTTCTAAACATAAAAAATAAAGCAACAGCAAAAAAGAGGGTCTGTCCAGCG
AATAAGAAGAAAACCTCCTTTTCGGCTTTTGAAGATAGGTTGCAGTTGTCTGCGGGCACAAAATGGGCAA
TTTTTTTAATACTTTTTACG

>Positive Sample 7
AATATCCAGTAGAAACAATGCTTGGTATGTCGTTCTTCTTACTTTCTTCAGTAATGAGTTGGTAGTTTTC
TCTATTTAAATATGAATAAATCAATATGTACTTTCTTTCTTTAATCAAAATGTTAATATGATAAAAATAC
AGCATCCAAAGCAGTTAATCAAGACTTAAATATAAAAAATTTACATATTTAGAAAACAAAGATAGGGTAA
ATATTGAAATTACATAGTAAACCATACCTTAAAAGCAGAAATACTGATACCACATGAACTATTGATCAAT
ACTACTGCTATTCTCTTCCT

>Positive Sample 8
AAATAATAGGTGTAAAATGACTTTGGCTTTTAATATGCAACGGTTGGTGTTTCGTAATTTGAATGTTGGG
AAGCGCATGTTCAAGAACGTCCCCTTATGGAGGTTTAATGTCGCCAATAAATTAGGAAAGCCCTTAACTC
GCTCTGTAGGGTTAGGCGGTGCTGGCATAGTTGCTGGTGGCTTTTACTTGATGAATCGCCAGCCTTCTAA
GTTGATATTCAATGATTCTTTAGGGGCAGCTGTCAAACAACAGGGTCCCTTGGAACCAACTGTGGGCAAC
AGTACGGCAATTACCGAGGA

>Positive Sample 9
GCAACGCTGAGAGCTTACAAATTCTGGACTTATTGAATCAAGACTCTAATTTTGAAGCTGCAGCTATAAT
TCACGAACGCTTGGAAAGTTTTAACCTAGCAGTCAGGGATTTATTAAGTTTTATTGAACAATGTCTAAAT
GAAGGGAAAACAAATATATCTACTTTATTGGAATCTTTGAGGAGGGCCTTTGATGATTGTAATTCTGCTG
GTACCGAGAAAAAAATCGTGTTGGATATTATTGATTACATTCCTGATCACTCTATATGGGAAATATCCTTC
ACACGATGAAAGGAAAGATT

>Positive Sample 10
CATTCAGCGGGAAAGTAGTTGTTTATCACTAGACATATAATTATGTTTATTTATATTTAGTGGGAGCAAA
ACAGTTTATTGAATGTTTACCAGAACCGAAAAAAAAGCTCTTCTAAACTGTTGACATCCAGTTCATTTAC
TTCCACGTGTAGATGTGAAGGAACAAATATTTTAGCATCGTTCATACAAGTAATTATGCTATATTATCGA
TCCTCGGATTTCAGCTTCCGTTATATCGGATGATTGTTACTCGACCTTTATGTCGTCTTTTTACATCATA
TATGATAATATGCTAGCAGT

>Positive Sample 11
CCATTATCTCTTACGTGTAATTTAAAATTGTTTATAGTACTATTTGGTTATGCTTGTATGCCTCTATTAT
TTACTTGATCTTTTTATGTTTTCTTATGATTGAATTATTTATATTCTAAATTCCTCACGAATTTATACTG
AAGATTTCCTTCCAGGCGAGAATAATAAACACATATTTATGATGATAACAAGACGAACGTGTATTAAGCT
CCCAGTACGAGGGAAGCAGTAAAAATTATCCCAAGATCCATTTAAAATGGATAACTCCACGAGCTACAAC
AAAATACTAAGGGAATAGGC

>Positive Sample 12
CACGGTACAATGGAGATATTTGCATGCCTATAGAAACAAGTAATAGTTATCATATTATTTTCTAGATTTT
GTCACTGAACTTTTCCACTAATGAATCCTATCAAAATTATATATCCAATATGGCTGCATTCCCAACTAAA
TATTAAAATGCCGCTAAGTATAAAATGTCTCCGCATCGGTAAAAAGCATTACAAATGCGTATTATACTAG
CGAGAAAAAAGTATAAGTATCAATGCCAATCACCCTCTGACCATAAACTTTCTAAACATGAATAATAAAG
GTGTTGAGAGTTATTATCCT

>Positive Sample 13
CTGCTGGTTTGAGAACCAATGCTTTAAGTACTTTGGCAGGTATTTTCATCCTTGCTTTTTTTTAAAATGA
GTGCGTAACCGTACTTTCCTAAAAATAACTAAGTAGAAAGTATTTTAATATATAAACGTCAGTGTAAACA
TTCAAGTGATTTTAACTTTACGCGGTTGAAGAATGCTGTGTTCGAACTATAAAGCGTCAGAAAAGATGGT
TTAGCGAAGGCACCATTATGAAGATAGACACATTCTTCTTTTTTTTTTTTTTTTTTTTTTTTTTTTTTTT
TTTTCATTTACTTTTATTTC

>Positive Sample 14
CTTGGGACGATGATGTCAACGATGACATTTTGAAAAAATATGATACCAAATATTTGAGTGGTATTGCCAA
GAGATTAAACAAGGAAAACAAGGGTTTGAATCTGACTTCAAGTGATGCAAACACTTTTTTTGCATGGTGT
GCATATGAAATAAACGCTAGAGGTTACAGTGACATCTGTAACATCTTCACCAAAGATGAATTGGTCCGTT

TCTCCTACGGCCAAGACTTGGAAACTTATTATCAAACGGGACCAGGCTATGACGTCGTCAGATCCGTCGG
TGCCAACTTGTTCAACGCTT

>Positive Sample 15
TGAAACGTTAACAAATGATCGTAAATAATACACATATACTTACCCTACCACTTTATACCACCACACATCA
CATGCCATACTCACCTTCACTTGTATACTGATATGCCATACGCACACGGATGCTACAGTATATACCACTC
TCAAACTTACCCTACTCTCACATTCTACTCCACTCCATGACCCATCTCTCACTAAATCAGTACTAAATGC
ACCCACATCATTATGCACGGCACTTGCCTCAGCGGTCTATACCCTGAGCCATTTACCCATAACTCCCACG
ATTATCCACATTTTAATATC

>Positive Sample 16
ATTGTGAAACCGCTTTGTTCTGGAGGGCGAGAAAAAGTTACGGTCACTCTATCTTTTTTATAATTTGGTC
TAACAAACTTGATATCAGAACCACGTAGAAGAAAAAGAGACTAATAGTAAAAATATCAAGAAAGTTGACC
AATTTTTGTTATATATGTCCGTAAAAGTTATGACTTGGCACTGGTCTTGGTTTACTCATCAATCAGCTAG
GATCAGCGCTGAGTGACTGCTTGCGGCTGGGCGGCTAAGAAATGGGAATATGTACAGTACAAATTCCGAA
CTTTGCGAGCCTGCCTCGAT

>Positive Sample 17
TCTAAAGAATTGTTGGCATCTCTAAAGAGTGCTACTGAATCATTTGTTGCCACTTTTTAATGTGAACTAA
AAAAATAAAAATGAATATAAGGTACGTCTCCAAAAGAAATGTAAATATAGAAATTTTAAAAAAAAAAACG
AAAAAAAACATAACTAAATTTAAAGTGCAGCCCAACAATAACCCTGAAAAATCTAAATATCTTAGAATTT
TTTTATTTTGATTATTATATATTATTATTATTCTTATGGTAAATAATGCCCCTACTTTTCTTCTAAGGAA
GTGAGTTACCACCAAAATAA

>Positive Sample 18
TACGGCATGACTTAACAGTACAGTCATGATTTAGGAATTTGAGTGTTCTCCGGGGTTTTGCAGATAAAAA
TTTGTTTTCTTCTCTGCCATTCCTGGAGATTTTGAAAAGAGTTGGTAATGTGTTTCAAAAGAAAAAAATA
CATTCCAAGAATAAATACCAGGATTTCGTGACAAAAATTAAAAAAAAGGGAAGTATGGCATGCCTAGAAA
TCTTTTCTGGAAAACTTGAAGCATATCATATAATTGTATGAACTTGTCCTTCAAAAGATGTTACCAAATA
TTCAAGAGTATGTGAGCTTT

>Positive Sample 19
AGATTCCTGATTGCTTGCATGCCTAAATACTTCATAATTGTATTAGTTTAAAAGAAAACCTTTTACTGAG
ACAAAACTATAAATTTAAGAAAAAGGTTCCGGAGCCGGTTTAAAAAAAGAAGATTAAAGATATTATATTT
ATGTAAGAATTAAATTAATACTGTCAGAGAAAAAATAGTTTTAAAGGAGACGGTGAACCTTTACCTTCCC
TTGATATGCAAATATTTTTCAGCTTTTCCTAAAACTAAACATTCTCGACTTTTTTTAGAAGACTTTATG
GGTGTGTTAGAAACAAAGCT

>Positive Sample 20
ACTCTTGAACAAGTGCCCTCAGCAAATTCATCTGCCTCGAAATTTGAACTTATTTTTAACGTGTTCAATA
TTTAGCTAAAACGTCGTTTTCAGTCCGGGTACCGCAGATAAGCCATCCCGGATCTTTAAAGGAAAACGAG
GAATTCAGTGGTGCAGACTACACTTTAATCTCAATTGACCAAAGTGAGTCCTCAAGTAAGACGAATATTA
TTTTTCTTTCACGACTCAATTGAATTCCTAATAACACTTTTGTTTGCTGTATTAGCTTTTAGCTTTGAGA
GCGATGAAGCAAGAGAGAAA

>Positive Sample 21
GGAAAAGATTTGATGAAGACCAAAAACAGCATCGAAGTGCTTTGCTACGATTTCTTTTTTTACTCTCCGG
TTATTTTGAGCTATCTTGTCATTATATCTTTAAATTTATATATTTCTTTGCCCAGCTTTTAAGCGTTCAC
TGGAATTGCCCATATGTTCACTAAATATAAAACTGATTATTCTCTCCTCTATATTGTCATCTCGGCAGAA
AAGCTACTTTTAAATAAGTTTTAAACACCAGTTTTCTGCAATGATAAAAATAGCTACTTTGGATAGAGGC
TGAGGCCTAACTAGTTGGAA

>Positive Sample 22
CAAAGAGGTGTGTGGAGTATTATTAAGTAAACTGTACCAAAATGTGTAGATTATAGGATATTCACCATTT
ACTTTCTGGTTTCGTTTCTCGAAATTTTTCGTTGTCCTATTTTCTTCGGTAAGTAATTAGATTTCCTAAA
AAAGGCATATGATAAATAATATTACACGCGATTCCGCCATCTTGGGTTTCCCACATACATACTTCACGAA
GAAAAGAAAGAATTTAGTATTGTGTAATAAAATTGATGTTTATGTTTAAATATTTACAGAATGTTTAAAC
AGGTATTTAATCCATTTAGA

>Positive Sample 23
ATAGACTTTCATTCCAACACATCTGTTGACTAGTAAGATGGATATTAGTCAATAGATGATGTTTCTTATT
CCAAACTATCATGGGTTTGTAAGAAAATTTATTTTGAGGGGAATTGATTACATTTCCTTTTTTTTTCAAT
GTACTTTGAAGTTTTTTCTTGTTTAACAATAAGCTTGCCTAATCATTAAATTAGTGCATAAAAAAAAAGG
TAAGCCGAGATTAAATAAGTAAACAAAGACATACTAAAAAAAATCATTCTGGTACATACAGCATTCTAGT
TAATGTTAGATTAGCAGTGG

>Positive Sample 24
GACATCGTAGTAATAAGGTGCAAGCTTTTGTATCCTTCTTAATGATTTGTAAGCAAAAAAAAATTACGTA
CATCAATTTGTAATGGAAAGGAAATAAATAGTATAATATGAACACTCTTTCTGAGAACAGTTATCTTTAG
CTTGCCCCACAAACTGAAAGCACATTTTTAAAATGTTTTACAGATGAAAATGAACCCTTGTACTGTTATC
CTCTGTAAGTCGTTATTTTTCTTTTGCCTTTTTCAGGTTGACTGCTACTGTAATCGTAAGAATATTCAAA
ATCAATCATCTCGCATCGCA

>Positive Sample 25
TTTTATTAAAGGGTTTGGAGCTGTTTTCGGACTGAAAGCCAGTAACAAGCTTGTGTAGGAGTTTGTCTGA
AAACCATCTTCATTTCAAATAAGAGAGCGAGTAATTTTTAAATAATTTCTTTTCTGAACTACTTAAATCG
CTATGTACCTTGCGGAAATTATTCTTTAATAGTTTAACTAAAAGTAAAAAGTTAGAAATCATAAATAGAA
AATCAAAGAGACAAATCTCGTTGCAAAAGCTTGAAAAATAGTGCTTTACCAACAGGTTGGCAAACCAGAA
TGCTAACAAAACTTAAAATC

>Positive Sample 26
TTTAATCTGCTTTTCTTGTCTAATAAATATATATGTAAAGTACGCTTTTTGTTGAAATTTTTTAAACCTT
TGTTTATTTTTTTTTCTTCATTCCGTAACTCTTCTACCTTCTTTATTTACTTTCTAAAATCCAAATACAA
AACATAAAAATAAATAAACACAGAGTAAATTCCCAAATTATTCCATCATTAAAAAATACGAGGCGCGTGT
AAGTTACAGACAAGCGATCCCTATTCCATGCAAGTTCGGTAAGTAGCAGAAATAATCAAACTGTTTAAAC
CCAATTAAAATTAAATTAAA

>Positive Sample 27
GGAGGTGGGCGCTCCCGCTATAAAGGTTTCTGATGGAGCTATGAAACTATATTACCTAAGTTACTGGGTA
GGATATGGCTTGAGTTTTTCTTCTTATACTGCTCTGTGTTACTTCTTCCCTGTACCTGGGTGCCCTGTTA
ACAACATTATAAAGGACAAGGGCTGGTTCCAAAGATGGGCCAATGTCGATGATTTTGAAGAAGAGTGGAA
AGACACAATTGAGAGGGATGACCTGGTAGATGACAATATTAGTGTCTACGAACACGAACACGAAAAGACT
TTCATTTAAGCTCTCCATAT

>Positive Sample 28
AACTATCGCACTATCGAAACTTTTGTTTCACAGGGAATGGAAGAAAGGGCCTTTATTTTTTACATTTTTT
TAAGCCATAACGGATTTTAGTTACCGAGATTTTCAATCCTATAAGAATTACCTAAACTATTAAAAGCGGA
CCCATCTAAAACATAACGTTCGGCAAGTAGATGTATTGTTCCAAAAAGGCAATACATCCATACAACGGTG
CCACCGATCTGTAATGAATGCTCCGTAATGTTTGGGGGATTCATTAAATTTACTGCTGGAACCGAGAGAG
AAACACGACAGAGCAATTCA

>Positive Sample 29
GTGGTAAAGGCACCTTATAATACAGCTTGGTTCTATGTATTAATTTCAGACTATTTTATGGTCTGCTTTT
TTTTACGGTCAGAGAAAAGATGAAATAGATGCCACAAAACAGGGATATATACGAAAACAGCATCTTATAT
ACTTTTTTACTCCAACATTAATTTCCAAAACAGGTGAGATGAGGTAACCCATTCGAAAAATTTAAATGTT
CAGACACTGTCAAGTTAAAGAATATAACCTGGAGGTTTTCTTTTCTTTTTGTCCATCAAAATGCACTTTT
TTTGAAAAAAAATGCCTTGT

>Positive Sample 30
GCTTCTTCCCACAAGAAGAACAAAATAATAAAAAATATTTTGAGAAATTTTTCGTAACAAAAGGAAAAAC
TATTGAGTTTAACTTATGAATGTACTTTACTGGCCAAGAATCCGTCTGGAACCATTCTACGGTGCTCTCT
TCGTAGCGCTAAAGACAGCTATAGTGGATATTCAGACGGTATTGAAAATCACTAATGATATCAGTTAACA
AAGTAAGAAACATGTTTCGTCAGATAAGTTCTACACTGCTGCAAGTGCATACAATGCTCGTTTTTATAAA
GTCCTAAATTATATAACCCT

>Positive Sample 31
TTAAAGAGTTGAATTTTACGGTGAAAGTAAATTAACGCTACTTCTGTTGTTTTGGAAAAGCCAAGTTCAA
GTTTCTTTTCTTGATTATTTATTCTCGTAACGATTTCGCTTTTCCTCTCTATATATTTTCCTACTCCATT
CTGTAAAGAGCTTCCAGAACAAAATATAAATAAATAAAAAAAAACTTCTCTTCAGGCAAGAGTACGTTCTT

CGCTGGTTTCCTACAGAATCAAAAGAGTACTGCAGCTTATTGTACACACTTACAATATTCAAAACATGTG
TGGCCTACTCGGTTCCTGGC

>Positive Sample 32
GGATAAAAGTTCAGTTCCTGTTAAAAAAGAATAATTCTTACTGAGAATGAAGTATTCAAAGAAATTTAAT
ATTTTTGAAATATTTTTTGGGATTGCGCAAACATATGCCCTCTCAAAAGCAAGACAAAAATTTAGCAAAC
TTTATAACGATTACACCATTATTTCTCATAATTTTCGCTAGACATTTCTACAGTACTTGTGGACGTTGG
AAATGAAAGACATAGTATGAAAAAGATGATCAATTTTTCGCAATTGGTTGAGTAAATTTCAGGTCTTTTT
TGGGGATATTTTTCAACTGA

>Positive Sample 33
ATTCTGATGAACAGCCATCTATGGAGCACTTTTTACCAATTGAGAAAGGCTTAGAAGAATTAAATTTTTG
TGGAATCAGGTCTTTAAACGCACTAAGCACTATCTCAGTATCTAATGATAAAATTCTTCTTACCGATGAA
GCAACAAGTTCCATTGTTATGTTTAGCCTAAATGAGCTTTCTTCTTCTAAAGCAGTAATAAGTCCTTCAA
GATTCAGTGACGTTTTTATTCCTACACAAGTTACGGCAAATCTCACAATGTTATTGAGAAAAAATGAAACG
TACTAGCACTCATTCAGTAG

>Positive Sample 34
AGATATTTTCGCTCTTGAAAGCTATAGAGATTAATTATGTTTTTCCCTTGGTATACCTTTTCTGTGTTGT
CTTTCAGTTCCTGTCTTTAGGATGTTATTTATCGATCTTTTTCAGAAAGACCAAAAGCGAAGAAGCGAAG
AAGAGGACTTCTCTGTACTAAGACCAAATTATGTAAAAGGAAAACTAAACATGAATATGTGAATTTATTT
CCAAAACACAGCAGAGCTTAAAGATGGGGTAAACTCATATTCATATTCATATACACGTATGTATATAAAA
ATTCATTTAAAAGGATTGCT

>Positive Sample 35
ATATTGCTGAATTATTAAATCACAACATTCCGGTATTAATATATGCGGGTGATAAGGATTATATTTGTAA
TTGGCTGGGAAACCATGCTTGGTCCAATGAGTTGGAATGGATCAATAAACGTAGGTATCAGAGAAGGATG
TTAAGACCATGGGTCAGTAAAGAAACAGGTGAAGAGTTGGGACAAGTCAAGAACTATGGCCCTTTCACCT
TTTTGAGAATATACGATGCCGGTCATATGGTGCCCTATGATCAACCGGAGGCAAGTTTGGAAATGGTCAA
CAGTTGGATTTCCGGTAATC

>Positive Sample 36
TAAAACACTTGGAAAAGAGAATATTTGGCTTAGAAAAATCTCTTGAAAAAAAAAAAAAACAGGTAAGGGC
TGACAGCGTTAGATTAGACTTGAATAGATATACTATCGACCAGTTTTTGACATTACTGAAAAGCCTCAGC
GAGGTTTTGCAATTCCACAATGTTTATGGCAATGATTTGAAGGAAAACGACGATAATATTATAAAGATTG
AAACCTGCTGTAGTGCGCTAAACATGAAAAATTGTTTCGAAGACTCCTCATTTCGTTTACAAGAAAACAG
CTTCAAGAGGCAATTGGGCC

>Positive Sample 37
TATGTTTAGCTATAAAATTTAACTTTTAGAATTTTATTATATTATTTAACTTTCAAGAAAATTTTCTCAT
CACTCATCCCAAGGACTTCAAGAAATAGAACAATGCATCATAAGTTTATGAATTACGTGCATTTGGACCT
TCAAAGAATAAAATCGAAGTTATAGTTAATTATATTAAATTAAGCCAAAAGTACATATATCTAAAAGGAA
AAAGTATAAAAATTATGCAGGTCCAAGACATACTTAAGCCTTCAAAGAAGAAGCTCTTCTCTTTCTGATT
TCGGCCTTTTCAGCCTTTCT

>Positive Sample 38
ACGTCTTGAATAACACCATGTGAACCAAACAGCTTTGTCTCAAAGGGATGTGAAATTTTGGACTGTCTTA
TTCTAGTAGAGCCGTGTTTTGTTCTATCGAATATGTAGATTGCACCATCTGAAGAGGCGCCGGCTATAAT
ATCTGGATTTTGAGGCAAATATCTTGCCCTATTGCATTCCCCGTTTGGGAAGAAAATACTGATGTCATTT
ACTAAGTGTTTGGAGGGAAACCTTGTCGAGTTCTCCGGTTTGAATTCCATTTCGTCCATGTCGAAATTAT
TTAAAGATGACCATTTTATA

>Positive Sample 39
GAAACAGCGTGATTTGTAATAAAAATAAATAAGGACATATATGTATATAGAAAGTTGCTTAAACTTTTTA
GACCACTTGGAAATATTTTTTATTTATATTTTTCTTCAAATGTAAAGTTGGGTACCTAAGCTTAAATTCA
CCTGAAAATGAATTATATGAAAGATTTTACACCACATGTAAAAAAAAACGTACAAAAAGCCAAATATTTC
CACTCAATCATAATGCATGAATTATAGGTATTTTAAGTTATATTGCTGACGTAAAATTCAGAAGAGACTA
CAAGAAAAATTCTGAACATA

>Positive Sample 40
TTATTTGACCAAATATTATTGATGTTTATTTTTTTCTTTTTTGCATAGCCTTTCTACATTTCATGGCTTT
ATTTATTTATATTTATTTTTCGTTTCCTATTTTTTTGCATACATAGACACTACCTTTGATTTTTGCTTGT
AAGATGTACGATATAAAGTATACTATGAAAAATTTGAAAATATTGGTCTTAGTTACTTTTTCTGGTTTGT
TTTATCAATTCATGTAGCAGTGTGCACGAAAATCTAGTTACGCAATGTGAAATCTAGAAAACCTTCTACA
ATTTGGAATTATCAATCACC

>Positive Sample 41
ACACGTCTGAAGAGCAAAATCTGAAGCCGTTTGTAGCGCTTTGCAATAATAAAGACTGTGGGTGTGTTAA
CCACTTGAAATGTCTTCACAGGTACTTTCTAGATGATGAACAGTTGATGGTAGGGAGAAGAAATTTGATA
CCTCGAGGTGGGAAATGTCCGAAATGTGATATGTTTTGCGACTGGACGACCCTAGTCAAGTTTTCGACAA
GAATGAAGTTAGCTCACGGGAAGTAGGGCCTGACACAATTTTCGGTTCTTTTTTTTCTATAGATAATGGT
TATATGTTATATTTACGGAT

>Positive Sample 42
AAAGTGTACCGAGCACTGTGCCCAGAAGCTAAGCTTTTTTCGCTAACATATATATATATTTAGGTAAATC
ATTTACACTTTTCGTCGATCTAAAAGCTCATCGCATTTTTTCGGTCCTGATGTTAAAAATTTATTTCGTT
TATTAAAAAAAAAAAAAAACATTACTGAAACGTATAAAAGAAAAGTCTTAATCTATAAATATGGTTTTATC
ATAATTAGACTGTTTTACTCTGTCCATTGGTCATTATAAAGCCTGAAAATACCCACTGAATGGTTTTTTT
CGCAGGAGCAAACCTTTTCC

>Positive Sample 43
AAGCAATTTTACTTTCAACTGTTTAATATTTTTTAGAGGCTACTCGGCCGGCCATATCTAGACAATTAGT
AATTCCGATGACAAAGAGTTTAAAACCACAATATCTGCCACACATATCGTGAAAGGCATTAGTTCCATTG
TCTCCGTATTAGTGCAAAGCCCCTAAGCCAGCCTGCTTTAAAAAATGTGCAGGTACTTTTGTTATTGTTT
TGATATATAAAGAACTACTATTTGCTAATATGTGATGTAGCGCAGAGAACTTGCATTTCCCTGCATGGCT
GACCTTGACAAGAATACAAT

>Positive Sample 44
TTTTTTTATTTGCATGATATACATATACTATTTTTTATTAGTTTCTTATTCTACTATGTATTTACGTAAC
AATAAATTTACAGGTCTCTCTTTTTGTAAGTATCTTGCACGCTGATATCTATAATACTACTTCACAAGTT
GATCAGCTTCCGAGATAGACATTTTATGTATCAAGTCGCCATGTCCGTTCTCTACTGGCCGGTGTATATT
ATGATATTTCGACCGGTTCTAATACATAAGCACCGCCCACTCTGAGGAGAAGACATTTTTACAAGAATTA
CTAGCCATATTTTCCCTTGT

>Positive Sample 45
AACATAAGGAAGGCGAACAGGCAGCGAATCAAGCAAAACAATTTCTTGAGCCAACTTTAAGCCTCCGTTA
ATAAGATCAAGTTTGGTATCCTTGTAAATAAATATGTGTGTACGTGGTTAAATAGTATGTTTTTAGCATA
ATGCCTTATTTTGTACCAACAAAAACATGATAGTTCCCACATATCTTTTCGCCTGGGGAATACCAAGTGG
GACAAACAAAACGTTAAAAATTAAATCCTTTAGAGTACCACAAATTCGCAAGAAACAAGATAGTTGAATT
GTCAATCATGGCTAAGATAA

>Positive Sample 46
CGATGTAAATCTAAGATAGCTGTCCTTACTATATAAATATTTGCTGCGGCCCTTTTTTAACGATTTGTAA
AGGATTACAGCAGTTCGTTATACTTGGCTGATAGCGTTAATTTTGCATACTTTTCTTATAATTGGTAATT
TTCCTACGTATTATACAGTTGTACGTATACTTATATAACAAAGTTCATGTGTACTTCATATTAACCTCAG
CGATACCCCTACAGATGATGTGCCAATCCATAGTTTTGTCTTTTTCGCGATTATAATGCTTTTTCCACCA
TTTGAAGAGCTCATATACGA

>Positive Sample 47
TCCTTTAAAAAGCAAGAGCCTTCCCCCATTGAAATAATCTATTGCAATGAAAACAATGGTTTCATAAACA
TAAAATCCTTAGAAAGCCCCACTGACGATAGCATGGAGGCGGACATTTCACATAGAGAAATGGCTACAAT
CCTGACTAGAAACAGAAACAATTTGGGTAAGGTTGCCATTGATAAGAAAGGCGTTAATAATCATTGCATA
GATTTGAATGAGCTGAAAAAGGGCCTTGTCGCCAATGAACACAAATTGGATAACGATAACTCAACACGGC
ACCAGAACACATATTCACCA

>Positive Sample 48
TTATTTCATGGTTTTTAGGCGGAAAGAGTTTGGACGGCTTGTCTCTCCTAATGTGGAAGAAATTATGTTT
TGTTATGAATCTGAAAAGAAAAATACTATTTTAGCAATACAATGAATTTAAAAAGTTACCCTGTACTTTA
ATCCTTCAATTAAAACACTTCTTAAAATAGTATATTTTGAAGATCATCGATATATATACAGAGGCAGTTT

TGCAAGGCAGATAACGTTATTGAGTTGTATGTAGGCTGTGTGATTTTAATCTCATAAATCAATAATGGAA
TAATTTTCTTATTGTTAATA

>Positive Sample 49
AAGTCAAAAGGCCATGTTTAGGGTCAAATTGAATTTCTTATGGAGTTTTACTTACAACTTATTTGCTATC
CTTTTGGCGGCTGGTGCCTTTGTTGACTTTCATATTCCACCAGAGTATGCGGGTTTAGGGGAACTAGTTA
GTATCCTCCCTGTAATTTTTGTAGCTATACTTCTGCGTTATGCAAAGATTTAGTCCAGGTACTGCTGCTA
TTTGATTGTAGATTTTCTTTTTTCTTTTCTTTTTTTTTTTTGACGACTCTGTAATACTCTATTTATTATT
TAGTTTAGTTTTCTATTTAA

>Positive Sample 50
TGTGAGTGATATTCTTAAGTCTTGCTTTTCGAGGGTGTAAGAAGCTATGTTCTTCAGGCGAGATTATTCT
ACTCCTGCCTTACTTGTTTGTAATATTTAGTTCTGATGGTCATGATAATTCTATATACAGTTACATTAAG
TATATACTTAAGCGGGCAGCTTACTAATATAAATTTTGTGGCATTTTTGTTGGGATATGAGAATCATGTA
TCGTTGATTTACAAAGCGAATTTACGTTACCAGGAATAGGGAATACTCTCTTGAATTCTAACATAAGCAC
AGAAATGCTGAAAGAATACG

>Positive Sample 51
GCACGGCACTTGCCTCAGCGGTCTATACCCTGTGCCATTTACGCATAACGCCCATCATTATCCACATTTT
AATATCTATATCTCATTCGGCGACACCAAATATTGTATAACTGCCCTTAATACATACGTTATACCACTTT
TACACCATATACTAACCACTCAATTTATACACACTTATGTCAATATAACCACAAAATCACCACTAAAATC
ACCTAAACATAAAAATATTCTACTCTTCAACAATAATACATAAACACACTCAATTGCGTATCTATACCAC
CATGACGTCATTAACGTAAA

>Positive Sample 52
AGAATCAAATAGGTGTATCGCAATGGAATGTAATTTCTTAAGTATTCTATATGTACTTAAAACCTATTAA
TATATGGATCAACACAGTATCTTATGAATGGGTTTTTGATTTTTTTATGTTTTTTTAAAACATTAAAGTT
TTCGGCACGGACTTATTTGGAATTCAAATTATTAATGAAAGAACAATTAACTAATTAATGTACTTAGTAT
TTGGCCATTATTATCGATTTCGGGGGCCAAATCTAACCAAATTCAACCTACATTTTTTCAAATTGATTCA
AACACCTTTCACAATAAGAT

>Positive Sample 53
GGAATTCAAATTATTAATGAAAGAACAATTAACTAATTAATGTACTTAGTATTTGGCCATTATTATCGAT
TTCGGGGGCCAAATCTAACCAAATTCAACCTACATTTTTTCAAATTGATTCAAACACCTTTCACAATAAG
ATTTTTATATCTAGCGCACATAGAATGAAATGTAAACAAAGATTTCAGAAAAATCGTCATTCAAACTTGT
ATTAGACGAGGGACGGAGTGATTTTTGTGTTTGTTTTTATTAATTGTGGGATAGGATAGTAGCAACTCTT
GGAGGAGAGCATTGTCAGTT

>Positive Sample 54
TAACTTACTTCAACATGAAAGCCCGACGTTTGCAATGATATCCTCTTTTTCACCCGATTATTTTCATTTA
TTAGTAAGTGGGGTTCTGGGTGAAAAAATCAAAACAAAAATATGAATTTGTAATGATTTTTATATTTTCG
CCGGAGGTGCTGGAAATGGCAAACGAAAATACTATGACATAAAAGCTGGGCACACTATGTACGTTCTTTT
TAATTTTTTATCAACATGAGAAAAAATTCATGAACACTGTACCTTATATTATCTAATAAATGAAGTAAGCT
TTGCATCTCCGTAACAGCTG

>Positive Sample 55
GCTGCATATATATAATTAAGCGGGAGCTTTCTTTCGATCTCTTTCATTCCATATGTTTATTATGTACTGA
TGAAAAGCATTTTACTAAGTTGAGGTCTCTGTAAGTATATTGTGACCATTATGCTAAAATACTGGGGTCT
TCATTTGTGTCAAATTCTACGTAATAGTTCCCCTTTTTCTTACATTTTTATACTAAAGAGTTACGGTATT
TATACAAACGAGGGGCTTTACTGTAATATATGATTAAGATTCAAGCTGAAACATTCTAAGTTAGAGGTAT
AAAATGATTATTGATGAAAG

>Positive Sample 56
GCTGGGAAGATTATTACGGAACACATTCTTATAAATCTATAGGAATACATAGGATTGTATTTCTATTGAC
AAAGTACAACCTATATTTTATGTTATGTTATGTTATGTTTGCTATGACTATAGTTGCTTTTCTTCACTTA
GTAAGGATTAACTAACTCTCGAGCTAGTAAATTTGACGTGAGAAGCATAATTATATTACTAATAGAACGA
TATCTGGTGTGTTTTATTACAGACTGCACTGAGTGTTAGAGGTAGAAATTATAAGTTAAAAAGCAGTAGT
TTATGCTTTATGCTCGAGTA

>Positive Sample 57
ACTAGTGCTTAAGTTCTGTTGACGCACACAGTACCTATCTTTGATTCCTTCGTGCAAACAGTATTCCGGC
ACGTTAATTGATACCAAAAAGATTTCAAAATCAGATGCTTCAAGACTAATTGTATACTTTATGTGACTTC
TGGAGTTTAATTTGTTGTATTGATGAGATAATATTGTCTATTATATTGCCAATAACAACCAGCGCCAGGT
GTACTAAGTCGAGATGACAGAAAATTTATAATAAGTGTAAATACAATAAATTTTGTAAATAAATAACTAC
TTCAATAGAAATCTCAAAGT

>Positive Sample 58
GCGACGCCCGACGCCGTAATAACTACTTTCGACAGACCACTTATGACAGTATTTCAGGCCGCTCTTATAA
AATGACATGTTAACAAACAGTTCTGATTATTCGCCTTTTGACAGGACGATAATGTAAATAGTTGTGGTAG
TATCATTCAGGTATGTAACTGTTTACTTTGTATCGCTTGAAAAAAATAAGCATTTCAGAGCCTTCTTTGG
AGCTCAAGTGGATTGAGGCCACAGCAAGACCGGCCAGTTTGAATGCTCAACTCTTCAAAAGAAATTCCTC
AAATATGTCCAGTTTCATGT

>Positive Sample 59
AAGCTTAACTTCTTCGTGAGGAAGGAAAGTGTTGTCTCCTACTTTTTTCAAATTTTCGAATTGTATTTAT
ATTTATTTAGTACTTCTTGAGTTTACATATCCTTCGTAAAAATGCAACTTTTGTCGAAAAACACTTCCAA
AAAAAAATAATAATGAATTTATGAAGCATACTAACGAGCGAGCACATCGCTGAGGTATCATTACTTCATG
AGATAAATTAAGATCTCCTCATATGCGAATTTCCTGTTCAGTGATAAACGTTGATTACGTTATTGATAAA
AGTCTTTTCTTCTGGCAAGG

>Positive Sample 60
GAATTCTAGGTGATATTGCAATTACTTCTTCTCATGCACTAACAAGTGAATGATAGAAATATGTTGAGTT
CCTAACTGCCTGATTTTAAATAAGTTTCATATTATAATCTTTTAGCATATATATATATATATTGATCCTC
TCTCTTCTTTATTTTCTGCCAGTAACCCATGTGTGAAGAAGAAAACATAAATAAAAAAGCAGTAGCACAT
GGACACATTCACGCCCGAACACTCCTAAAAAGCAGCCCACACAAGAAAGTAGATATAATGTAGGACACCC
AGCTTGTCCATAATTGCTAA

>Positive Sample 61
CGATCAGCGCCAAACAATATGGAAAATCCACAGAAAGCTATTCATTGAAAAAATAGTACAAATAAGTCAC
ATGATGATATTTGATTTTATTATATTTTTAAAAAAAGTAAAAAATAAAAAGTAGTTTATTTTTAAAAAAT
AAAATTTAAAATATTAGTGTATTTGATTTCCGAAAGTTAAAAAAGAAATAGTAAGAAATATATATTTCAT
TGAATGGATATATGAAACGTTTACTGGTGGAAGTTTTGCTCATATATTATTATTCAATAGAAGTAATAAA
GAAAAAGTTGGTAAAGCAAC

>Positive Sample 62
TAACTGTTTATAACCGTACCTTGCTTACATCATCTATATTTTCGTAATCATTTTTGTACATTGTTTATAT
CTTGTTTTGAAGTTTTAGCTTTGAAAAACATTTAATTGCGAAGTAACATAATGTTATTAATAGTGTACGA
CTGAAACAGCCATTCTATTTAAACATATAAAAGTACGATTAGTTGGTGTGGTTATATAAGTCATAAAATA
ATTATCATATTAAAAAATTGAAAAATAAAAAACTACTCTTTTTTATTTCAATAGTTCTCGTTATTAGTAG
GTCGTGCTCTTAAAAGATTA

>Positive Sample 63
TATCTTGTCTCGTTATGATATTTTGTGAGATAGGTATCCAAAATTAATAAGAGAAAATGTCGTATTGTGT
TCACAATTCACCTGATTGTTTGACTAGTCCAACCCCTGGTATAATTGTCACTTAATATATTCTTAGCTTC
CAGGTGATGTTTTATTCCATTATTTATGTTATGTATATATAATCGTAACTCTTAGAGCAAAAAGTAAAAA
AAAAGGTGGTACTTACCGAGGGAGAATATATACAAATTTATTCGTAAATGTAAATAATTAATTGAAGCAG
AGCTAAAAGAAAAATACAAG

>Positive Sample 64
TGCCACTGTATAAAGATGTTCAGAAAGATGCATAAAATCTACTGCAATTTTTTACTTTTAGTTTGTTAAA
TTTTAGTTTTCGTCATATATATATAAAGTTTAAAAAGGTACAAAGAATGCAGTAATAAGAAATGTAATTACA
TGAGGGCAGTTTAAGTGCTACCTTTTAAGACGGAAGAACTAATACCATAACCGAGAGTAATTACGCAGAC
AAACGCTAACAACGAAGCAGGAACACCTCTGGAGATAAAAGCGCCTACAGTTAGCCATCTATTACCCTTT
TTATCGGTCATAGAAATAGC

>Positive Sample 65
GCCTAAAGATACGGTAATTGAAACGTTTCCTATGCACAATCTTAAACCTTTTTAGGTAATTGATTAAGTT
GACTGTAATATCTGTAAAAGATTACATCTAATTTACGTCTGGTTTCTTATCAAAAAATATTATCTACTGT
AGTTATGCAAGTATTCTTAATTTTCAGTACTAAACATGAATATTAATTATTTGTGAGCGTACAAGAAGCA

AAAGGAAGTCATCTTTCTTTGAACAGTTCTGTTTTTAGGTAAGCCTTGGAACCTTAGCTATACTGACTTG
ATACGCCCTGTCTTTGTTGT

>Positive Sample 66
TCTAGAGGGTACAATAATCTGGTATTACAAATGCCTAATGCTGCAAAATCCATATGCTTAGAAGGTGCGG
TAGGTAAAAACACCATAGAGTAACAAGTGAAAGGATAAGACCCAAATTCTTTCGAGTAGAAATCTATAAT
TTTTTGGCAGACAACTGTCGAATTTATAACTGTTAACTCATCTGTTTCCTGCGTCGGAAGTGTGAAAATT
TGAATGGGTATAATATCCGAACCAATGTCGTCATCCATAGTATCGTTAACATTGTCCACAACATTCTCTC
TCAACTTGTCTTCCTCTACT

>Positive Sample 67
TGGGTATTTCCGTGTATATGGTTAATAATAGTAGTATCTTGTCAGTTTTTTTTATGTTTTTCTTCGCGCG
TCAACTTTCTACCAAGAGAAAAACAATATAAGGTCTCCTTACTCTATAGGAGAATAAAACAAACAAAAAT
AAAAAGCACATCGTAGCGCCAAGAAAATACTGCAAATACCAAATACCACAATAATACTACAATTATCTAT
ACACAAGTGTTTTGCCGCTTAAAACTTCGATTTTCATAGTACGAAACTATACACCCTTGGTTTTTCTCTT
TTCTAAATACATATCTACCT

>Positive Sample 68
AAAATAACGGCATTATCGTACACAACCTGGTTTTCTTTATTGGTTAAGTTGTCTTTATTCAAAATTTTTC
ATTACTTCTTATAAACAGCTTCTTGACGCTTGTAATTTCATAAGTTTTTGATACAAGCAATGGAAACAAA
ATTAAGAAAATGTAGGCAACGAAGAACTAAATTTGAAAAAAAAAAAAAAAAAAAATTTACTGCCAAGATCA
TCTAAATTATCTTATAGTAGCAAAGCTTGAGCATAAAAGGATCAATCTTTTAGTTTTTTATGGCAAAGTA
CATTTGCAGTAAGTAATTAG

>Positive Sample 69
TGCAAACAAAACTTTTCAATAATAATTTTATAAATAGTATCAATATATATATATATATATATATTTATTT
GTTTACTTTTTCTATCAGTGTTTTCAATTTTTTATTAAACAATGTTTGATTTTTTAAATCGCAATTTAAT
ACCTAAATATAAAAAAATGTTATTATATTGCAAAAACCCATCAACCTTGAAAAAAAAGTAGAAACGTTTTAT
TTAATTCTATCAATACATCATAAAATACGAACGATCCCCGTCCAAGTTATGAGCTTAATCTTCCATAAAA
ATATTTGAAAGCAATAGATC

>Positive Sample 70
CTGGTTTTGGTTTTGTAGAGTGGTTGACGAATAATTATGCTGAAGTACGTGGTGACGGATATTGGGAAGA
TGTGTTTGTACATTTGGCCTTATAGAGTGTGGTCGTGGCGGAGGTTGTTTATCTTTCGAGTACTGAATGT
TGTCAGTATAGCTATCCTATTTGAAACTCCCCATCGTCTTGCTCTTGTTCTCAATGTTTGTTTATATACT
CATATTTCTATGTGTTTATACAATTGCTATTGTTTATATAATGTAGTGACATTTTCTCTTAATCTTATAC
TAATTTCTATGACATTTATA

>Positive Sample 71
CGTACGCCCTGGTTAAGACTTGGTATAGTTCCAATAATTGGAATATTACTGCATAGTGGTCCAATAGCAG
GATTTAGCATAAACATGATAATATTTAGAGATGCTTTCATCCCTCTGACGAAGGTGGTAGAGAACAAGAA
AATGAATAACATTATATCATATTCCTATATATATATATATATATATATATATATCATATAACGGTGGAAA
ATCCCGGCGATATTACAGATAAACATTACACCCGCATGAATGTGAGCCACTACTATATTATAACTTAGTG
AATAAAGAGTGTTACAATAG

>Positive Sample 72
TCCTTTATATGCCCATCTTGTATAAGGTAGTTTTAAATTCTAAGTTATTCAATACTACTGACTAATCATT
GTTAATAGCAATTTCGCAGTCATGTTGGGCTCAAAATACTATAGTCATTTTTGTGATTTTTTTTTTTTAT
TATTTTGATTGAAGAAGGAACAATTCGAAATATGGAGAAAACGTAAAAAAATGTTAGAGTTATAATCGAAA
TTATCCTAGTTCAGATACTGAAACTTAGGCATAGGTGTAGTCACAAGGTATTTCCCTTTACATATATATT
TGTATATGTGCATTCTCTTT

>Positive Sample 73
GATTTCTCAAAAACAAAGGTGCAATTACATGTTAAGCACCGAATTTCTATGCGAAACAGGTCCGTCTCAT
CAGTCATAGCTTTCCAAGTATATAAAACTTGAACGCAAAAGTGCGTCATCGACGCCTTTTTTTGCGCAAG
GATTGTTACTATTCATTTAATGAACTCTGATGTCTTAATAAGCCAATGGTATCCATAAGTGAAGGTGAAG
TCTACTGTGTTACATTAGCAAGTCAGAACTCCTATACGATACAGTATACTGCATTATGGGCACTAAAATA
ATTTAGATTATAGAAAATAT

>Positive Sample 74
AGTTAAGAAAAAGTCTAAAAATGGTTTTTTCATCCAAAATATTAAATTTTACTTTTATTACATACAACT
TTTTAAACTAATATACACATTTTAGCAGATGCGCGCACCTGCGTTGTTACCACAACTCTTATGAGGCCCG
CGGACAGCATCAAACTGTAAGATTCCGCCACATTTTATACACTCTGGTCCTTTAACTGGCAAACCTTCGG
GCGTAATGCCCAATTTTTCGCCTTTGTCTTTTGCCTTTTTCACTTCACGTGCTTCTGGTACATACTTGCA
ATTTATACAGTGATGACCGC

>Positive Sample 75
GCTGATGTTATCGACACAATTATGTCCAAACAGGAACCATAAATGTTCAAACCTTTATAAAGATTTTATA
TTCAGAGTTTATAAGGTCTAGCTTATATAGTCAGTATTTTTAATTTAATTATATTATAGTTTATCCTATC
TCACCGTTTTAGCTTGTTTTCTGCGCCCAAACATAAAAATCAAGAAGCGCGGATAATTTATGATCAGCTC
AGCGGGAATGTTAATGGCAGATACTTAGAATTTGGTGGAAACGATTTTCTTCTCTAATCCTCATCAGAGG
AGAGGTAATCGAGCAAGGGA

>Positive Sample 76
CGTCGCTTGTTTCACAAGATTACCAACGCCAGCCATATTGTAACATAGATGTATAACTAGAACAATTTAC
CACATATACATAACTATTTCTTATGTTTATTTTCCTAAAATCCTTTATCCAACTACAATCTACTAAGGCT
TAAGCTATTTTCCTTTTCATTATTTAATAACTGTTCTTTCAGTTTACAGCTCCATTTATATCCGGTTAAT
TTTCTATTGCTACCAACGATTCTATGGCAAGGTACTAACAATGCCAGGTTATTTGAGCCGCAAGCTCTTC
CGACAGATCTTGCGGCAGTT

>Positive Sample 77
AAAAATTACGTAACGGTTTATAGAAATTCTAGTATGAGAACAGTAATATATGAGAAAAAGTTGTTTTCAC
TTTGTGTTCGAAAAAATAAGTATTTGAAAACGAAAAAATAGTTTGTATGTTTGTATATAAGTTGTTATTA
TCAAAAATTAGAGGGAAAAAGTAACTAAATATATATTAAAAAAAAAAAAAAAAATAAATGTATGTTGGAAT
GAAATAGCGTAGTAAGGATAAAGGTGTAATAGATATATATTATAAGAAATAATCCGGTGTCTTCCTGGTG
ACGGTTGGTTCACCGGGTCT

>Positive Sample 78
GCTTTGGCAAGAAATGATTCTTTTGTACTTGGAGTAATGGTTTCTTGGAGTCTTCTTGTTTTTGTAACGG
GAATACTTTCAACAGTTTATATATAGCATATAAGTAACGACCGTGCACATATTAGGGTGTTCATATCTTG
AAAAATCTTTATGTTATCCATTTTACTTTAGGGGAGGTGGAGCTTAATAATAAAATTTACATAAGAACAA
CAATCTGTAAGTAATTATAAAAATATATTAAAGGGACATTTTAACCCTACTTTAAAAGTTTAACTACATC
AGTGGTTCATCAATCAAAGG

>Positive Sample 79
GAAGCCAAATTTGACGACATTTTAAAACGATATAAATGTTACAATTTCCAAAATGCTAGATTATTGATAC
GGAAAATTTGGCAAAACGAGGCTGATGGCATCAGTGAGCATGACCTGGTCCACATGATTGACGAATTAGA
TTACAATATTAATTTTGCCTGAGCAGCATATATTTTCTTTCCCCGCCCCCTGGGTGTCTTAAATTATTGT
TTCACCACACAAACATATATATGTTCTTTTTTTTTTTTGTAGGACGTACGGATTCAAAAACAATTTATAC
CTATACAATGTAGAAATGCT

>Positive Sample 80
TGTTTAACATATTTCTGTAGGGTTTTTTTTTTTTTTTTTGTACAGATTAGAAACCATACAGTGTAAACCA
TTATATAGGTCAATTAATATATTTATAGATCATCCTGTCAATTTTTTTTATTTTTTTTTGCAAAGCTCC
CTTTTTCCTGAAACCAACAACAAAATATAAAAAAAAAATGATAAGCAGGATTTTTTCTTTTAATAATTCC
TTAAAATTAACTAACAAGCTGAAGCCCTTACAACAAAGTAAGTCAAACCCAATTCACAAAAAAAAAATCT
GTTTTATTTCAGATATGCAG

>Positive Sample 81
GAAATTTTCCGAAAGTGTGAAAAAATTGGATTTTTTTTTCTCCTATTGAACTCAATGGAAGCTATCAGTT
TGGAGAGGGACAATTATTTCCTTCTTGTTTTTGCCAAGAGAACAATATTGTAGTATTTAAGTTCTAAT
TTAATGTTATATATACTTATCTAATAGTTTTTTTTTTTCAATTAGATTGTTTTCCTTACGACGTTAATCC
CTTGTCATCCTCACTTACGAACTTCGATGAATATGACATACGTAATCGGGAATGTTTTGTAAACTAATTG
ACGTAGTTCCTTTTTCGTAA

>Positive Sample 82
ACGACCGAGGTCCTACAGCCTCCCTTTTTTCTTATTTTACATATTAATATACATAAATATACCATAATGC
ATTCTTTTTCTAATGATTTAGCTCTCCTTTTTTTGACAAATTTGAAAATAAATATAAAAAGGGCCATAAA
ATATTGAGTTAAAATTGACGATTTTTTTATAGAAATGACTTGAATTTACGATTTAAAATAAAAATATAC

CTGGCATATAACTAACTATACAAATCATCATCTTCCTCCGCATTAGAACCAAATGCAGCACCTGCTCCAC
TTGGAGCACTATTATTACTG

>Positive Sample 83
GCAATAATTTTATGCATTTCTTCTATCTATTTCAGAAGTAATTGACGTGATTTTTTATAGTTTTGCTTTG
AATCTTTGTGTTTCCACACCGGAGAAAAAAAAAGTTCATGAAGTGAAAAAGCGGTTAAACATGAATTTTCT
ACTATCTAAAGATAAAATTACAGATGCAGTTACTTACATACATTGAACAGTTTCTGACGGCAAATTTAGC
TTCTATTAATTTTTTTCTGTAGCTGATGAAAAATTCTGTCCCTATGATGCGCCAATGGGTCATCAACATC
CGTCGGAAAAAATTCATTTC

>Positive Sample 84
CGTTAACTCCGGCCATATAAATGTGGTAAAAAAGGGTACTGCGCTAATGGCCTACAAGTGTTCAAAACCT
CTTCTATATAATAAGTTATAGTGTTCTTGTACATATGTCGCAGGCGCGAATACGTGATGTGATTTTAGCT
GTTTAATTTTAAATTTTGTTCTTTTCCTAAACAATTCACGGAAAATCAACTGAGAGATTTTTAATATATA
TAAGAAATACTAACAACAATAACAGCAGCTGTTAAGGGATCTTACTTATTATTTAGCTCATTTATAACTT
CTCCCAAAGTCTTCTGTTTA

>Positive Sample 85
TGAATAATAGAATGAGTTACATACGTAAACGTGTAGTATTATTATTATTATTCTATTTCCTTTCCGTTTT
TATTATTTATATATATATATATATATATATATATTTGGGTGTTTATATTTAGGTAGGGCATAAGGATTTA
CTGTCGGCATGAACCTACTTGAACAAAAAAATAAAAATCAAAAGAAAAATAATGTAGGCTTTTGCACATC
GACTATATATAAATATATATATACACACTTCTGTGCAGAAGAAAAGGAATAAAACGTCAAACTAATTTAT
TTCTTCTTGCAACGATTCCG

>Positive Sample 86
GAAGGCAGAGAACTCTAAGTTGAAGGAAGCTAACGAAGATCGTTCGGAAATTGATGACTTGATGCTCTTG
GTTACTGACCTAGATGAGAAAAACGCCAAATATCGCTCAAAGCTGAAGGATTTGGGCGTTGAAATTAGTT
CGGATGAAGAGGATGATGAAGAAGATGATGAAGAAGACGAGGAAGAAGGGCAAGTGGCATGATAGAGTTA
AAATATGTGCATAAACAAATCGCTTGAACGATCTGTTTGATTATGTGGGCAGCACTACTTTTTTGTAATT
ATATATGCATATATACATTT

>Positive Sample 87
GTGAATTTTCTATCCGAGTTTTAACTTTCGATATTCAGAAAGAATAGCCGTTCATCTCTTTTATATTATA
TTTAGCGATGTAATGAAAGTAAAAAAACAAAAAAAAAAAAACAACAATAATCAAATGACGCATATCTAAAT
ATTCCAGAAAAGAATATAAATAACGAGGGTGATAAAGCGGGTGCAATTAAATTCTCTGTAGTACAAAAAT
ATGTAATAACCGCAGAACTTGTTAGTGGAATCTCTTTTAGATGTAGCTTGAAGGCTCAATTCGCTCCCCT
TCGGTTTGCTGAAGGAAGAA

>Positive Sample 88
AAACAGTGGAATTGCAACAATTATCAAATTATTCACCTAATAATCACCTTTAACATTCAGGGAGGTTTAT
TTTTGCTTCCGAAATTTTTACAGTCTACAGGGAAGAAATAAATATTTAGTTGAGAAAGTTTTGTTTATTT
GTAAGTTATTAATTTGAAAAATAAATACCATAACTAGCGCAAAGCTTATAAAAGAGATGTTTGTTGAAAA
ATGCAGAAACCCCATATATAAAACCATGAAATGTCACAATGGACATGATGTACATTCTTGGAGATAACAT
CATATTCGAAATATTCAATA

>Positive Sample 89
TTGATGTAAGCGGAGGTGTGGAGACAAATGGTGTAAAAGACTCTAACAAAATAGCAAATTTCGTCAAAAA
TGCTAAGAAATAGGTTATTACTGAGTAGTATTTATTTAAGTATTGTTTGTGCACTTGCCTGCAGGCCTTT
TGAAAAGCAAGCATAAAAGATCTAAACATAAAATCTGTAAAATAACAAGATGTAAAGATAATGCTAAATC
ATTTGGCTTTTTGATTGATTGTACAGGAAAATATACATCGCAGGGGGTTGACTTTTACCATTTCACCGCA
ATGGAATCAAACTTGTTGAA

>Positive Sample 90
AATGCTTCATCCTTCTTAAACTGCAAAACACAGTCGTACGTAGGGTTGTTTGTTCATTTCTTTAACCTTC
GACGGACAGATCTGGGGTACGTTCTAATAAAAGTAACCGCGCAGCCTATCTCCACGCGAAGGGGTAAAAG
ATCAAACAAGTCCTTAAGAATATACAAAAAGCTACATAAATATAAAAAAATAAAGGAATTGAGCGTAAGC
ATCTTTGTATTTGTCTTTACTAGATCAAAATAGCAAATTATTTTTTTACTCTTTTTCTTTTCTTAATAAA
CGCATTATCAACTGAATATT

>Positive Sample 91
ACTCCTTTTCACGTTTTTGCAAGAAATATTCCTTCTTCTTGCCATGATAAAAAGTTGTATTTAGATATTA
TCTCGATTTAAGTGGCTCTTCTTTTATTTCTTGCTACTGGTGGTTTTTTTTGTTGTCTTTCGTCCCAAGA
CAGAAAAAGAAAAAGAAGAATATAGGCAAAAATATAAAAAAACAAAACAAAAGAGGAAAGGGAAATTAAA
AGAATAGCAAATGCTTTAATCACACACTTTGCGCTTCCTACAAAATTACGGCAATGATTTTGGATGTAAC
ACGTAAAGATAAATAGCATA

>Positive Sample 92
AATAACAGTAATTGAAAAGCAATATTAAAATATAGAAGCCCAAATTTTATATTTCAAATGAATAAATGTT
GTTTTTTTTGGCAATTTTTTGCTCTTCTATATAACAGTTGAAATTTGGATAAGAACATCTTCTCAACGCG
AAAATGACGCCTCCAGTGAAAAAACATAAACTTTCAATGCAGTCTTCGGTACACTTATGAGTAACCCATA
TAGAGATCGTACACATTTTACAAGGATTTAGAGACAAAGTAATATTCTTCGTTGATATAGAGGTGTTCAA
TTGTTAAATGCTTTTCTTCT

>Positive Sample 93
TACTGAATCGGAGGTAACAAACGTAGCAGCTGGTGTTTTCATCGAACATGATGAGCTTTAAACAAGGAGG
AGTATAAACTTTTAATTTTTTTTTTTCACGTTTTGCCTTTTATATACCGATTAAATATTTATATTTCTGA
CTTTTTCTTTACCGACACCAACAGGTGTAGGCTCAAAACATAAAAAAAAAGAGTATTCTATAAACAGTTC
TTACGAATAGAACAACTCTTAAAATCCTTTTCTTGCCAAGTCATCGGTTATCTTCAAGTAAAAATTCAGG
ATGTCAACCTTCTCTCCGAC

>Positive Sample 94
TTCCAATTCAGTTTGTAGTAAACCATGATAGGCCACAAGACATTTTTTTTCAACTGGGGAGAAATTTAAC
AAGGACGTCTTTTTTTATTTAGACGATAAAAAACCAAGAAACAAAACTAGTAAAGGTTCTTGGAAGCATT
TATCTGGATGCAAAATAATCGTTATTCTTGGGCATGCTGACAATGCTGAAAAAGTTATCCAAACAAAGTA
GTCAAATATTCAAGATCACTATTAGATCGCTAAACATTAATTTCTCATTTTGCGACGATACTTTTTTTAC
AGAATATAAATTCATGTTAA

>Positive Sample 95
ATCGAATATTTTCCTGTAATAATATTGTTTCTTTTAGAAAAATTTTGGTTTTCTATAATATATTTATTCT
CCATGTTTTAATTTTCTCAGCTATTAATGTTCAACTTATTCCTTATAAATTTGTCATTCATTTTTCATAT
ATATCGTTAATATATAATCTTTGCCTGTTCTGAATCCTTTTTCAACGACCAAAACTTTAAAAAAAATAAG
TAAAAATCTTACATACATCGTAAATGAAAATACTTAGGATTGTAAATCATCAATTATACTAAATAGACTT
TTCAAAACGTTCCTTTTTAT

>Positive Sample 96
TAATGTCCAAGGGCTTTCCAGGAGGTCAGTATATTCAAAGCCAAAATAATTAATGTTTTGGTCTGAATTT
TTATTTTTCAAAAATGAGGTGAAAACCTCCAACGAAACTTTTTAATTAAAACCATCCGATACAGAATTTT
AAATCATCTAAAGCATTTATTCGGCATTTTATCCTTGAATAACGTACTATGCAACTCATATAAATACTAC
AATATACTTTCTCTTTAGGAAATTCGTAGCTATCAACAAAGGTAGTGTCATTGTATATATACTTCCGCCT
TCCTAAAAAAAAACTTCATTA

>Positive Sample 97
AAAAATTCCTAAAGTTATAGTTCACATTGTTATATATGTATCTATAAAGCGAAATTTTTTAAAATTAATT
GATTAATTAATATTTTGTATCTAAAGTGTTCATACTATATTATTTACTTAGATTATACATAATTAAAAAA
AAAAAAAACTTTTTTTCCTTGTTTTCATTTTGTTTTTATTATTAAATATTAAAAAAAGTGTCCATAACAT
CAATGTTTTTTTTTACCTTTGATTAGTTTTCATTGGCGAAACCATAATCCCCGTTCCATTCCCATCATAA
CTACGCTCTCTTCTGAGTTG

>Positive Sample 98
GGGAGTTATCGACAAAGACTGCCCAGATATTGGAAAAACAGATTACTTAGACACTAAACTTATTTTTTAA
GCTGTATACACAATCAAGTATTTTTCTATTCACTGCGATAAAATTGTGTTTTTCAACATGTTAGGTCAAT
GGAAAGTCATTATTTTGAAGCAAAATGTAAAAAAAGAATATTATTAAGAGAATAAATTCTTTCAAAAATG
TAGAGCAAGTTATGTGGAAATCAAAAGAATATTAGTTGATTCTATTATATAATAAATAGTTATAATCATT
GTATTTTGATGATGAAAATT

>Positive Sample 99
CGCCTGAATTGAAGAGTACTTCAATACGTAGTGTACTGTCCAAACTCTCTTCCAAATTTTTAATATTTAG
CTGGGGTTGGGTAACAAGTGAGCAAGGGAAAAAGTGAACATTTTAAGAAGAACAATAAAATAGCAAGAGA
TGGAATGGTAATGCTTGGCTCTCGAGAAGAGTAGCATAAAACGAGACTTGTTAAAACAGGATATGACAT

ACTTCAATTCAGCTTTCCCTATCAGCCGCTCGAGCAGTTATATAGGTGTGTTGCCGGAGTAATTTGGCGG
AGGCCAACAGTGGCTAGGCG

>Positive Sample 100
GCATTACTGGTGTTCCCAAAAGCGCTAATTGACGTATTGCCAGTATTGCCTGTATTGGTACCAAACTGGG
GCATTCCAAATGCTGAAGTGGTGGTTCCTCCTGTCATTGTGTTTGCTGTTCCAAAACTAGGTCTTCCAAA
TGCACTTCCTCCCATGTTATTGGTACTAGCAGCATTATTTGTGAAAGGGTTGATACCACTTGTATTAGAA
AGATTAGGCTTAGCACCCGACGTGAATGGGTTACCGAAAGCTGACATTACAGTTGAGTCGTTGCTCGGGT
CGAAATTTAATGATAGCTTG

>Positive Sample 101
TTCCTTTGATAGTCTTCAATAATTCTGTTTTTTTCGTCTATTTCTTTGTGTATTTGATAAGCATTTTCAT
CGTTTGTATCTGGATTCCCAAGTGTTCTACTTGCACTATTTCTTCTGGTGGAAACATTGCTGACACTTCC
CCTTGAGGTTTCGTCGATATAGCTATAGTTTGGAATATCATAGTTGTTTTTCTTCTCAGTTAACACTTTA
GCCCTATAATTTTCATACAATACGTTGTTTGTTACATCTTTTAATTCCTGAAGGTGAGAACCTAACAGTA
TTGCTTTTAAGTATATAAAA

>Positive Sample 102
GGTGGCATTTATTTTATTTTTAGTATTTTTTTATACGTGCGTATTCTATTGTTCATTTTTTTACCAATTT
TTTTTTTATTTTTATATTTTGTTTCTTGCGTGGAGTTTTTACAGGAAGTTAATCACAACAGAGGGTTCAA
TCTCCAAGGCATAAAATAAACAAAAATTGTAATAGTTAGCAACTGCAATACGAATTAAAATTTGAGGAAA
AATCTAGTATTGTTCACACGAGCCAATGCTCTGAAGTTTCGAAAGCTAATAAATATCATGTGGAAACTTT
TCTGAGTCATTAATAAGCAA

>Positive Sample 103
TTTTTCTTTGTCGTTTTCTTCTATAATGAACAATTTACTTTACGGTCTTTAATCGATTTTAATGATTAGT
TGGGCTTTTGGCTACATATGTAAAATTACATATAATATAATGGACACATAAGCAAAAAAAAAAAAAAAAA
AAATAAACAAATGAGCAACCCATCAAATACCAAGAACAAAGAATTTAATATAATATATAGTTATTAAATT
TAAATGTATATATGCAGTTCTGCTCTTATTTTGTGACCAATTTCTTTAAGTCATCCAAACCGACCTTGCC
ACTATTAGATTTGCTTGAAT

>Positive Sample 104
GAAATGTGCATTTGAATTTGCGCTTGTTGTGAAGGGGTTGGTTCTCTGCCCAATGATGTTGTTTTCTTGT
AAATATTCAATTCCTTTGTGATCGTTTCCTGACGCTTCCTTGGACTGTGTTAATTCAAGAGAATCTCCTA
AGGTATCCTCAGCATTCAAAAATGGATTAGTATTCTTTAATGTTTCCTTTTGTAATGGTGCCTGGATAGA
ATGGCTCCTGCTATGAGCATTCCTATTTGGACTTCTTGGCGGTATATCAGGGGCAGAAGTTTCAGCCGTC
GAAGTTCGTGCGATATTTGT

>Positive Sample 105
CAGCTAACGCAAGTATTGAATTCTTTCTCTTAAAGTACCCCTAAGTTGAAGAATTCATTTTAATCTTCCT
TGCTTTGTCCCCCCCCCCTAGAAGCATAGTTTTGTCTTAACGCTGTAACGGTCTTGTATGCTTATTTTGG
TGGTAGGGATCGGTTTGCACCTTGGAAATGCTATGTTTACTTTATTAAAAAAATAATTAGTTTTCCTCCA
AAACGTAAACAAATATAAGTTTGAGAGACAAAGAAAAGAATGAAAGAGTCTTATATATATATAAGTTAGC
TGGTAGCATAACCATTTTTC

>Positive Sample 106
TATGCTTCCAATTCTGATAAGATCAATGAAAAAGTCGGAAAATATAAATCTTGTTTAGAAACTCTTCAAG
AAAGGATTGCAACGTTGACGAGCCATAAGAATAATCAAGAAACTAAATTGAAAGATTTAAGACAAAACCA
CCAACTATATCAGAGAAGGATAAGCGGATTTAAGACAAGTATTGAAAACTTGAACAAAACAATAAATGAT
TTAGGGAAGAACAAAAAGGAGGCGGATGCAGAATTAATGAAAAAAGGTAAAGAGATTGAATACCTGAAAA
GAGAACTAGATGATTGTTCA

>Positive Sample 107
CTTGAAATCTTCGAAAACTGATGAGAATTCTTCATTATTTTTTTTTTTCTTCTTTTATTATTTTTTATGT
TTATTCCATATATACACTTATAATTTTTTTTTTTGCGATTAATATAAGTTTGCCTGGATACACGAACATA
TATATGATTAAAATGGTTGTCTCGAACAAAAAAATTCACTTTTTAGAGGGGTTTCTTATTTATTCGTATA
TGTACGTAATTTTGATGTTTAGGAAAAATTCACGGAAAAGAATATATATTTGAACAGAATCAAAAATTAG
GAGGGAGGCAAAGAAAGTAA

>Positive Sample 108
TTCTTCATTTAGATTGTCTCGGCTATGTCACTTTTCTATCGTTTCACCTGCACATGGCTTTGTCTTGAAT
ACTGAAGCCGTTATTATAGTTCTATCTTCATGTTTTTAGAGGTTTTTTTAAATGAGTTTATTAAAATTA
AATTTTGAATGTTATTTTTGAATAAAAATATAAAAAAAATAATATTAATAATTTTTCATTAACTGCTCAT
GATATCAGTGCATTAGAAAATCTTTTGGTTCCACTTTTCCTATAAAAATAATTCAAGTTTTATCATTATA
TCATTTTTAAATATTTGATT

>Positive Sample 109
TGTTGATATAGATTCCAACGAATGTTTCTTCACCTCCTTCAAACAATCAGCTACCATAATACTGGCGCTT
ATAGTAACTATTCTGTGGTTCATGCTGAGAGGAAACACTGCTAAAGCAACCTATGACCTGTACACTAATT
AAAACACTTTTATCCATTTTTTTTCGTATAAGCATATTTTACATCTTTATATATATTTATTTTTTTTTT
TTTATTTTGGTAGAATAGATTACATATACAACTTTTTCATAATGTCAAACTATAGAGTTTTATTTCACAT
AACGGAAGAAGCCTATTGGA

>Positive Sample 110
CACTGATTGTGAAAAGCTCCGGCTCATAATTAATTTTTTTGATGAATTATAGAGTACTTTGTTTATAATA
GAAGACTTTTTTTTTCCTCCTAAGGTAGCTTATGCGAGAAAGAGCGCCTACTTCGCTTCTAGCGCTACAA
AACAATAACTTTGAAGGGTAAAGCTGTTAACTTTTCTTCAAAGCTTAGAAAAGAAAAAAAATTAATAAAA
AATTATACTTCTATTGGCAACATCTTGCCATACAATATAAATCGTAAGGGTTCATTAAAAATATAAAATC
AGAATTAGAGTGCATTTCAA

>Positive Sample 111
CTAAGATTCCCGGTGCTATGTACTTCTCTCACAATAATACAAGTAGTGAATGTCACTTAATAAATTTTTG
TAATAAAAAGTCTACAAAGTTTGTTTGTGACTTGTCGAAATTCAATTCTGTTGCTTTCATTTTTCTGTAG
AGAAGAAATAATATTCCGACAAAATGTAAATAAATATGCTAAAGTGGGTTACGACAAATTTCTTGAGAGC
CATTGATTTTTGCTATAGAGGCATGAAAGCATACTGCACACACATCCAATACTTAACTTTGGCAAATGAG
GAGCCTAATTACAGAGATAA

>Positive Sample 112
ATTGCCTGAATTCTTCACTATTTTATGATTTTTCCCTTCCTTCTTGAAATTTGATTTATATTTAGTCCAA
ATATTACAGAAAAAAAAAAAAAAAGATGCCTAAGCCTTGGGTCTTATTTCGAACAAATCCATCCATCGGTA
ACAATTCAAAAAAAGAATATTTCGTCTCGAACAATTTGCTAAGTATTGTGGGTAAATGATATATAAATTT
TTATAAAAACTCTTGGCCAAGAAAAAAAAAAAAAGACTATGTCTAAACGTAACGTTATCATTATTATTAAA
TCATTTTAATGTTTTATAAG

>Positive Sample 113
CGCTACATTGGTCTACCTTTTTGTTCTTTTACTTAAACATTAGTTAGTTCGTTTTCTTTTTCTCATTTTT
TTATGTTTCCCCCCCAAAGTTCTGATTTTATAATATTTTATTTCACACAATTCCATTTAACAGAGGGGGA
ATAGATTCTTTAGCTTAGAAAATTAGTGATCAATATATATTTGCCTTTCTTTTCATCTTTTCAGTGATAT
TAATGGTTTCGAGACACTGCAATGGCCCTAGTTGTCTAAGAGGATAGATGTTACTGTCAAAGATGATATT
TTGAATTTCAATTGACGTAA

>Positive Sample 114
TCTGCAAATACAAAAAGGAACTTTTGAGTTTTACGTATTCTTATGTATTAGAGTAGAGAATATTTTAAAA
TAATAAACTAAGATAGTGTGTATCAGTTGTTATTTTATATGTGGTTTATGTTCATTTTTTTTACTTCTCT
TTCGTTAATTCCATTTTCATTAACTAAATATATATTTGCCTGAAAAACATTATGTGCATGGCCTTCTTTT
CTAATTGAAAGGTTTACATACATCAATGGCATGATAGGATGTTGCAACTTTCAATCTAAACATGGTTGCC
ATATAAAATATTACTCTAAA

>Positive Sample 115
GATCCAGATTTGCATCTCTATCGCTATTGTCGTCATCGACACTGCTCACTTAGTGCTGCAGGTAAATTCC
GTTTTTTTCCAAATTAATATTTATGAACGTTATGATGTCAAGTTTTTTCAAGAAGTAATTATCCGCGAAA
AAAAGAATATAAAAAATACAAATGTGCATAGATCCTCACATAGTATACAACTAAAAAGCAAACAAAAGAA
CATCCTCAAATGACTGTTATAAAGACAGAACCAACAACAGAAGTGACATTATATTCTCCACCATCAAAAG
AATCTTTAAGTAAAGATGAC

>Positive Sample 116
CCATCGAATAGAAATGCAACCATCTACCGCTACCGCCGCTCCAAAAGAAAAGACCAGCAGTGAAAAGAAG
GACAACTATATTATCAAAGGTGTCTTCTGGGACCCAGCATGTGTTATTGCTTAGTTTCTGCGTACAAAAA
CGTTGTTCTCCCTCCTTTATCTTCCTTTTCCGCTACACCAATATATCATGTTTGTTCGTAATATTTCTTT

TTAGACCTAATAATAAATATCCTAAGTAACTATATTATATAAAATATTTGATACCCTGTACCTGCTTTT
GTTATCGTTGTACATCCATG

>Positive Sample 117
TAATTGTCGATTTCCTTGAAGTACATCTATCATATAAAATCATTTTCTTTTTATATTTGGAATGTTTTT
GTGCTAAAAAAATCATTATTCTGGGCGACATACAGTAAAATAATATTTAATATGAAACAAAGCGTATCAT
TTTGTAGACACCGATATCACGTTTGAACAGGTGAATAAAAATGCTCTGAATCAAACTGACCCAGCGTACG
TAATAAAGAATCCTGATCATTGCATACTTTCATAAACCAGCCTTTGAATTTACAATCTTGTAAATAGCCT
TCCCAAGTTTTTGATATTGA

>Positive Sample 118
GATCCTCTTTGGTGCGGCGCTAACAATAGAAAAAAATACACACACATTAGATTGGAATTAGAGCTTAAGT
GGTACAAACTAGGGCTAATAAAGAGGTAACGGTCGGTTCTCTACTAAGGTTCGTATTGTGTGGCACCGAT
GTTAAGCACTTTTAAGCGGAATAACTCGAGTGGAAATTTTATGTTTAGTTAGGTTTTACCTTGAATTTTT
TTAAAAAAAAAAGAGTCAGACAGGCTCGCTCTTTCCTACTAAATATTAGGAGCAAAGCAGTAAAAAGTCT
CTGAATAAGGATAGTAACCT

>Positive Sample 119
TTGTATATATCATATCCCATTTTTTAAGAGAGTAACTTCTTGTCGGCCTCATTGTTCATAATTTGTAGCC
AAGTTGTAGTGATAAATAATAATTTAGGCAGATATTCTTATATGCTTGAGTGGAACTTCTAGCATATTCA
GTATACTTGATATTAAAGCCTTTAACAACAATTAATGTTCGGAAAATTAAACTGTTACATAATTTATGTT
TAGCTCTTACTTTAATCCCAAAGAGGGCAAATAGCTTTTAAATCTTCAACTACTATAACTATAACTACTT
TTCTTTCATATATAGTTAGA

>Positive Sample 120
TGCGTAACTCTATCCACTGCTTTATACACATCACCAAAGTTACCTCTGCCAATACATGATTGGATGGAAT
ATAGCTTCGATGGAGGGGTTCTTGACCTAATTGATATTTCTTTGCTTTCCATTTTGTGCTATTTTCTTTT
GTTGTTTAGTAAATGAATTAGCTAGTAAAAAAACTAAATTTAAAAAGCAAATGCTGGTTTGCTTTCTTTT
GAGAAATGTTTGCTAAGACAACTGACTAGCCAGGTGTTCACTAAATCGATAATATTGAAAATGGCAAAAT
AAGAAAAAAAAAGAAGAGAA

>Positive Sample 121
AAAGCATTTGGTGCATGTTTAATGTTCTATTTTTAATACACGTAGTAAACTTCGTGTTTTCTTCTCTCTG
ACCTAAATTATTACAGCCATATATAACTTTTTTTTTAAATTCGGATAACTGGCACAGTACCAAAAGCTAA
AATTTCTCACAAAAACATAATTATATGACAAACTGAACTACTCATTGCTACAGGTAATTTCAATAAAATG
CGCTCAATGAAGTTGTGAATTTGCTTGCTTGTCTTTCTCGACCTTCTTTCAGTATTATACTCCATTTGAA
TCTGATCACAAACACGAAAG

>Positive Sample 122
TAACTGTTACCAAGCGCACATATTTGCATTTGCCTTAGCACAGTGACAAAATAAAACACGTAATCTGAAG
TGAGTCCGTCAAGCGTCTTTAGTCGAGGCTCCGATGAACCGTTCTTGTTGTAATCAGTGTACAGTTGGAT
GCGTCTTGTTGTATCATCGACGTATACGTTGCGCTGAGGACCGGCAAAAGCGAGTAGCTGAAGCTCTGGA
TAACGGTAAGGGTATCCTACGGCAAAATGGATCATCCACATAAGTGGCACCGTAAGCTCGTCAATTGCAA
CAGCGACTTTATTTTTAAGA

>Positive Sample 123
AACACTTTGACTTGAAGTATGAATACTACCATGGCGTCATTAACTTAAAAGTTCCTTAATATCGTCATAC
CTCTATACTCTATTCAATATATTGTAATATAAATGCACTCTATAGTCATACAAATACTTTTACTTCACCC
TATCTTCGCATACCATTGTCATAGAATCTCACACTGACGCATGATTAAAACGAATAATTTTACTATAAGG
CCTCCCATCCGTGCTCTATCCTTTTGTTTGCAATATTTATATACAGAATCTCAAAACAAGCGGGAGAAGT
GGTAATTACCCAGAGGTCAT

>Positive Sample 124
TTTTACAAGATATTCCTTAATAGAACAATCTAAATTATTCTATTTGAAAAAAATGAACAATAATTAACAC
GAGAATTTAAACCATACTCGGCCGGATTAGCGATCATTTCGTTAGTAATTAAAATTAACATTTCCATGTG
GGACTCTAACTGCTGCATCGCCAAGTTGATAAGCCATGAAATCACCAATATCAAAGATAGCAATCCAACT
GGAAAAATTATTCTCGGCATATGGGTGATGATAAAAAGCGTAGCTTAACAAACACCAGTGCTGGTTATTA
TCTGTAAAATTTGATTAGAT

>Positive Sample 125
TTGGTAATCGCCTAGTCACAGTCATTTAGAAGAAACTTAGCATCATTATTCCAGCATCATGCACCAGAAC
AGGAAAATGCCATTTCTAAAAGTATTCTCATAGCCATTAAACGATTCAATACTTTTTGGTAGCTAAGTC
AATGCCTCTCTGAATATTGTATACTATATGAATAAAGTGATCCCATAATCAGACTACCCAGCACTTAGTA
GCGCATTTTAGAACTAAATATATATTGCCTTTTTAATACTGTAACTTGAGAATTCTTAATAAGGAACAAC
AATGGTCCAGAAGGTGTTCG

>Positive Sample 126
AAATCATATCGGTCCAAATTTGCTCAAATTGTACATGAGATATAACTTTTTCTTCTAATGCTTTCATTTC
AGAGTTTGTTTTTTTTTTTTCTTTTTTTTTTTTTCCTTACCGGAGTCTGAATCTCTTACTTCCTTAACT
TAGATGTTAGTGACTAAAATCTGATGGACAGTATCAAAATGTAAATATTGTACCTAAAAAGAAAAAAGTA
TTAAAGTGGTTTGCCATAAATTTTAAACAAGTGAAAAGTTTCAATATTTATATTTACATGATTGTGGACA
AGCGGCATTGTCTACAGGCT

>Positive Sample 127
TGGGTGTATTGTTAAGTTTATGAAAGTGCTGTATTATTTGTAAAACAGCTTCTAAGTATACGAAAATTAT
AATAGCTTCAAGTTTATATTTTGTTGCTAGCGTTACGGTTATGTTTTTACGATACATATCGCCTAAGCAG
GTCTCTGCGCCTAAAAAAAGTAAACATATAAATTGGGAAAGCAATATAATTAAACATACTGCTATAGTAA
ATTCATTACTAGGCCAATTTAACCGTGCCTTATTATGATATGAAATACCAGTCAACAAGTCCCGCTTAAA
TGCACTCTAGTAAAGCTACA

>Positive Sample 128
CGTAGGGGAAGAAATGTAGATTTGAGGTTCATTTGGCTCGTGAGAACGACTTGAACCTTATTCATACTTT
ATATAATATTTTAGGAGTATTGTAGCATTATGTATATTTTTCTTGCGGGTGTGCACTGCCGAAATAGCTG
CTGAATATCCAGATTAAAAAACTAAACAAAAAACTGTTTCTTGTTCGAATAGTAAAAGAAGGTTTTCCCA
AATGCTATTGTTATTTAGAAACGCTGGAAATACAGTATTTTTTGAAGAACTATCCGTTTTACCCGAACCT
ATTTTAATTCAATTCTTAAG

>Positive Sample 129
CTCCTTATCGACTGCTTTTTATTTCTATAAATGAGCTTGTATACACATGTATAGTTTGTATTTGGATTAT
TTATACTTTTACGGATTATGTTCGTATTGTTTGTATTTCATTCCCTAATTTTCCTTTACAGATATAATTT
CATGATTAAATTTCAAGGCAAAATATAAAATTATTCAGAAATAGCTTCATCACACAGTGTCTAGCGAAAA
TATTTGTTAAAATGCTTAACCCAAAGTGTCATTCTCGACAGTGAACCTTTCCTAAAAACTTACAATTGTT
ATTACAAAGTTCTTCGGGGG

>Positive Sample 130
CGCATTTCCGCTTGGAACTGTTGCAGCTGTTGGCGCGCGTGGTTTTGCTGACGTGCCAGTACCTGGGCCA
CGTCCTTTTGGAACCCGTCCATCCGGCGCTCAAGGTCCGAAAATGCGTCTATGCTGGCCATTCTTGTATG
TGTGTATGTGTTGTATACGTATGAGCGGTTTTAATTGAATCGGCTTAGCCTGTTTTTGTTTTTGTTTTTG
TTTTGTTTTGGTTTGGTTTGATTTATTTTGGTTTGGCTTAAATCTTCGAGGAAATTTGCGCTTTCCGCGT
AAAAAGGGAAAAAAAGCAGA

>Positive Sample 131
CGTCAACTAAGGAAAAATATGCTCATAACTTACATATCTCGTACACCCTAAAATCCCACACAGGGATTAA
AATTAAAATTATGTTTAGGTAATGAAAAAATAGTAAAAAAATGCTTCCTTAAGTTAATTATGAATTTCCC
TATTAACACACATGCGTACACAAAAAAAGTATACGGAAACTATATTAGACTACACTACATCAACCTTATT
TTCTTTTAAAACCTGGTGGGGGCTGAAACTTCCTTGTTTGAGGGGGCGCTTCATTATTAAACCTCGACCT
ATTTGCGCCGATATTATCGT

>Positive Sample 132
TGAGCTCAAGTACTTTGGTCGTTAACGAAAGTTCATTTAAATGTTTCGCTATTTTAGTCTTTGTAATGTA
ACATAAATGGCTCTAGTATGCAAATCATTAAAAAAATAGCATGTATGTTAATTAAATTATCACGGCTCATT
CATTTGAGTTGATAACGCAATTCTGATAAGCATATCACGTGACTGTGGTTCTAAGAGTTACTCCGATCGC
CAGTAAATACTTTATGAGACGTTGCATTATTTCTGTTTAGTTGATAAATTTTGATTTCTAGTCTGTTGAA
AGCCTAATGCATGCAGATGA

>Positive Sample 133
GGTGACATTGGGTTCGAGTTAGGCGGAAGTTGGGCGTTCATCATCTATAACATTGTAAGACCTTTAGAAC
TCAAATATTTCGGTCGATGACTAAATGTTAATTGATTAATATTTTTGCTAATCATTGAAATGATTGGTTT
TAATGGATAAATCTCGACATTCGAATAGATAGCATATAATTCAAAAACGATTATAATGGGATATATATAT

AAGTTTAATAATATTAACTCCCTTCCCATTTCTTGAAGAGAAGAGATGTGTTAACTCCTCCAAATCCGAA
GCTGTTGCATAATGCGTACT

>Positive Sample 134
ATTTTAACTGTGTTGCCTTCCCTTGCTCGCAACTATGCTACGCTATATTGTACTCTTGAATTTTATTTAT
GTTTTGTTCCCAGCGGGCATCTTTCAATAAAATGAACTCAAGAAGAAGTAAATAATAACAAGCTATAATG
GGTGGCCATTTGTGATCAAATTTGGAGTGAAACACGTATGATTATGTTTGAACTCTCTTTCTTTGATACG
AAACCTCAAGCGACGTAGGTGATGGATATATTCACAAGATAACAAAGGCCGTGTTACAACATTAGATATG
TAAACAACGTGCTAGAAGTT

>Positive Sample 135
TAGCATGTGCTATTGATAACCCAGAAGCTTGTGAAATGTGTTCGGGTTAATGAGGGCTAAGTTTTTATTT
CTTCAATTCTATTTATCAAAATTTGGGTGTTTACTTCGAGGTGTAATTTATATTTTCTTTTGTTTTCTCT
GATAAAAAAATTGGTTTCCATTCTAATGCCAAGCTAAAAGGTACCTATATATGTTTACTAATTATTTTTC
TACTTTGCATATATACATAAAAAAAGTCGAATAATTTAACATGAACATTTTAAGCTGTCCTTGTAAGAAG
GCCATGATACAGATTCCCCC

>Positive Sample 136
CGGCCTTTCTTAGTTCTATCATATACGACGTTATACTTATTTACTTTCCCAGCGTTGAAATTAATGTGTC
TTTTTCATAATATTTACTTTTTGGTGCGGAAATCTGTAATATTTTAAAACAAAATCTCATCCTTTTGAAA
AGAAACGATGAAGAAAAAAAAATAATAACATACATTGTACCTTCACTAACACTTGTAAAACAAACGCTTAC
ATTATAATGGCACAAATGAGCAATCAGTGTCACTTCTTCCTACATAAGCATAATTCAAGTATCGGGATAT
AAAGCTATTATATACCGCTT

>Positive Sample 137
AAGGTTCAAGAATTGGAGAAACCGTGAGCGACTTCTTTGATACTTGGATGTAAGCTTTTATTTAGTTGCT
TTTTCGTTTTCTTTTATTTACGCCCTTGTTTTGCGTGTATACTTTACTTTTCTAATTCACTTGAGAGCGG
GACTATATATAAAAAAATGCACAATTACAAACTTGAGAATGAATGCATATCGTGAAGATTTTGATATTAA
ATTAAGAAGCCAAAAAAACGTAGGCAACCATAACAAATTTCGGCATTGAACTTGATTCTAAATGCTACAT
ACAATTACGCGCGCTGTTTT

>Positive Sample 138
ACTCTTGCCAAGCGATACAAGCTTTACAATCCTGTAACAAAAACATACTTTTAGCGGTTCCGACGGTATT
CTTCTTTTTCTCCATATATTTTGCTTATGTTGGCTACTTTAAAATAATTACCGCTTCCCCCACCAGAAAC
TAAACATGAGCTAAAGGCGCAAAACTTAAAAAATTACATAGCCAAGACTGTTTTCAAGAAAAAAAATATCA
GACCACATTTGATAAAGTACATACCTCTGTTAACTCCCGTAATACACCCATATTCTTGTACTTTCTACAA
TCTATCACTCAAACTTCTCT

>Positive Sample 139
GCTTCGGCGGTTTAATTTGGCGGTGCCATCAGGATTTACTCGCACATTGTGGCCGTTCCCTCGGGGATGG
AGTGTGTCCTGAACCATATTTTTTAACATTTTTTTGAACTAATTAACCGTCCCAATGAGGGATGTTGGAA
GAGTAAGTTGATTTAATGTCCTATTCTTGTTCTTTTCGAATATAAATGACGATTGGAAGGTGCATGTCAA
TGTTTATCATCATGTTCTTGTTGTAGGTAGTCCTTACCTTGGTTGTAGAGTGAGTAGCGACAGGCGGCAG
CGGAATATAAGAAGGATAAA

>Positive Sample 140
GTTTTGTTATAAGGTTGTTTCATATGTGTTTTATGAACGTTTAGGATGACGTATTGTCATACTGACGTAT
CTCATTTTGAGATACAACACAGTGGGCTAAGAATATACAACCGTTTTAGTCAATGTGTTGAAATATCATA
TTTTAATGCATTGCTCGAATCTCTTCCTTCGACTTACTTATATTGATTAAGGGTATGTATTTGATCCTTC
CACCTGGAAAAACTGAGGACCTTACAGTGTTATTAAGTGTATGATGTATCACTGTCGTTGTAGTATTTCT
CGGGAAGTTTTTCACTGGCA

>Positive Sample 141
AAAAGGAATGAAAAAAAAAAAAAAAAAGTTCAAAAATAGTAACAGATATCTTTCGAAAAAGTTAAAAACTG
GTAAAAAAATTTTGAATAGAGTATATAAAAACTAATAAATATGTTTAGTTATCATACACCATAGAATAACT
ACTAGAGATATTATTTCATTTTCGTTTTCATTTTTTTTATTTTTTTCTTTTCAATAAGGTAACAGAAAGT
TATTTATGCATTCTTGTTCTACATGTCTTATGTATACTAATAAGCTACTACAGGTATTTTGGTAGTATGG
CTTTTAAATGAGTTATTAAT

>Positive Sample 142
TTTTCTTTTACTTTGTGTTACTAATATTATTAATATCTTGTTATGATTCTTTGTTTTAGCAGCTCCAAAA
GAAAGGAATATTATAATATAATTGTTGTAATTCGGAAAAAAAATATTAAATTACTATGGTGCATATGCGT
ATATAAAATTGAGCATTACCTAACGCCATATATTACTATCTAAAGTCCTCACAGATGATGTGTTTTCAAA
ATCAAATGATTGGAGCTCCTCATCATCATCCCCGGTAAACTGCTCGGTTTCAGATCCATTATCCAGATAC
CTCAACGGGCTAGTGTACGC

>Positive Sample 143
TTACATTATGGGAGTGAAAGTATAATGGAGCACCCGTGTGAGTATGTGCTTGTTGACGTTAACGAGGATG
AACAAACGACAAATAGATTCGACGTATTGAAGTACTTCTTGGCCACTTTTCATCTTCTGACGCGGTGAGC
TTTTAAGAGCCCTTCTTTTTCGACTGGCCACGTGAGAAGACATACAGTTCAGCTTACATAGCTTTGGCCC
ATACAATACCTATGTGCCCCAAACCGCAGAGACGATTAGCACACCTTTTTACCTGCACCACAGTCATTTC
TCGATAATGGTGAAAACACA

>Positive Sample 144
ATATACACTTATGCCAATATTACAAAAAAATCACCACTAAAATCACCTAAACATAAAAATATTCTATCCT
TCAACAATAATACATAAACACACTCAATTGCGTATCAATACCACCATGAGGTCATTAACTTAAATGTTCC
TTAATACCGTCATACCACTATGCTCTATTCCATATATTGTAATATAACTGTACTCTATAGTCATACAGAC
GCTTTTACTTCACCCTATCTTCTCATACTATTGTCATAGAATCGCACACTGACGCATGATTAAAACGAAT
AATTTTTACTATAAGGCCTC

>Positive Sample 145
GTGACTTGATGGGTGGTATTTCCATCCAATCAATTAATGAAGCAGGAGGCAAAAACGGTGCAAGACTTTC
TGAAATCGGATGGTAGAACTAGCGATGCTCACACAACGCTAATTTGTTTCCTTCGAAGGGGCAGTCGGCT
ATTGTAGTTTCTATATATTATGTAGCACAACATGGAAACCATATTCAGCCAGTTTGTATATATGAGAATT
AAACGTTAAAACTTTTTCATCCTATCAGTCTTTCACATTGGCCTTGCCTAAAGGGCACAATCGTTCACGT
ATATACATATGTATGTTTAC

>Positive Sample 146
CATTATTGTGAATATAAGCTATTTGAGAATTATTTACCTTTTTACAGCCGCCCCCCCAGGATAAATATT
TCAAGTTGTGGTAACGGAAAAGGGCCTTACATACTCTAGTGAGCATAACACGTACAATGAAACTATGATT
AAGTCTTCTGCGCTTTTTAGAGCCGATGTAAAAAAACATGCTTTTTTATAACCAATGCATGAAGTATAAT
TTAGGCCCCTTTTGAGTTAAACTACTCAGTGTCGAACACAAGTTTGAGATTTCGCAGTCAATTTTTGGGT
TGACTCGATTATTTGCTTTG

>Positive Sample 147
CTTTTAAACTCTTGCGGAATCTCTGTGCTCGGAGAAAAATATCCTGGAAAAGAAGATGCGGGGTAGGCGG
ACTTAAAAGATAAGTGCACATTTTTCCGGACTTCGAGTGGCGACAGACAGCCTGTTAACTATTTTTGAGA
ATAATTAATCTTTAGGGGGGTTTTAGTAAAACAAACAAAAGACTCAAAATAACGCCAAGAACATCCGAAG
AAGTCCTGGTCGCGCTTTTTGCTGAGGTGGCTTTAAAAAACTAGTTTGCTGAGAGATGTAATAATGGCTT
TAACAGATAATTCCTCGTTT

>Positive Sample 148
CGTAAATTGTTCCGACAAAAGAGTTCTTGTCTATGATTATTCAAAATGCTGACTTTTTATTTAGCGCTTA
TTGGAGTCTTTGCTGTCTGTAGAAATCTGACCAGGCATACAAATTATAGTTATTTACTAACGCAAGAAGT
TTATTGTGGTTTTCATGTGTATGTGGCCACGTAGATTTATTTTGTAAATAAGTATCGCGACAGCTTCTAT
TTTCTTGGTTTAATACGCCTGTACTAATTTCCATTTTTGAGTTTAGGCGGTGCTTTCGTAAAAATGATAT
GATTATTCCGCTCATAAATG

>Positive Sample 149
TCGCGACAGCTTCTATTTTCTTGGTTTAATACGCCTGTACTAATTTCCATTTTTGAGTTTAGGCGGTGCT
TTCGTAAAAATGATATGATTATTCCGCTCATAAATGGTAGTCATGGTAAGGAAGATAAAAAAAGCGCAGT
TCCACTGTCAGGCTTCGTTTATTTAACAATATAGATACATCATAAAAGTTTTTGGTAATGTTCTCTGGTT
TTACTTCGTACTTCTGATCAGAAGTCTGTTATATCATGGTTCGGTACCAATTTTTTTTATACACCATTGT
GATGATGTTAAAACTAAACG

>Positive Sample 150
TATCTAGATGGCTTTTATTCTATTAAAATGATCTATCATAGACACATTCATAGGTTAAATAGGACGTTCT
CCAAAATATGTAACAATACTATATAGAATAATATATGTGTTTTATTCTGCTTTTAATATTTCCAGATTTT
AAAGTTTTGATACAAAATATGAAATTAGGGACTTCAAAAATTCTAATTTAGCCTCGAAATTGAGAAATGA

AATTAGATCATAAATTAACTCTAACAAAGGAGAATATATATACGTACATATATATATATATATATATAAG
TTATTGTAATTGAGAAAAAA

>Positive Sample 151
TTATTTTATGTTGATATCCAGCTTCCGTTCGTCAACTATTCTCTCGAGCTCAGTTTTGGGTTTTGGCATG
TAAACAATGCCTTCGCTTGTATTACGCAAAAAAAAAAAAAAATAAATAAAAAAAAAAAAAAAAAAAATAAGG
TATAAATCGTTGGTTCTTTTATGCACAATTATTTAACTATAGTTATCTATTTACGTAAAGGCTTCTATTT
TTCCTTATCTACAAGAAATTGCATGAAGTTTAATTTTTTTTGTCACCTTTGATCTTCCTGAATGTGTTGG
TAATGAAATTTTCTAGTCTC

>Positive Sample 152
TTACAAAAAGGGTGCCGTTTATTAATTAATGTTTCTTCCCTGAAAATATGGAAAGTACAAGTTTTTAGTT
GAGAAGGGTTTAAGAAAGTTTTGAAAATGATCTAAAAAAATATAAAAGCAATCAAAGAAATAAAAGCTGG
AAAAATGCGTAATAACCGAAGTGACTAAAATTTCTTTACGCGCCAAATAAGAAATCGATGCTCTTGAAAG
TAGCAACCATTTTTTAAATAATATATTCCTGATGGTTCTTGGCCCAAGAGTTTTCTTGAACTTTTAACGT
TAAGAAGTTGATTCTGCTGA

>Positive Sample 153
AAAGCTTGCCAAAAAGAAAAAGCGTGAGTACTACCTCTCTCTGTTAGTGGATTTGCAGGACAAACTTTTA
GACAAATTAGATAATGAAAAAGATATGGGGTGGAAGATAAGAATGTTCATTTTACGTTTTGTTACACAAA
TCCAATCGAACCTCGAAAGCAAACCCGATAAAAGAGCAGTATTTTCAATAATCTCCCAAATCTCCACAAA
ACATCCCGAAATCATACATTTGGTTGTAAAGTCATTGTTGTCGACGTGCAACAAGATAATATCTCTCTCT
GACTATGAATATGACATCAC

>Positive Sample 154
CATTAAGTGCGTCTTTCAATGACTTCACGCTTTGTAAAGTTTCCTTTAGCAACAAAACGTAATTAATTCT
TTGGTCAGGTTTTATTGCAGAATGATTAATGCATAATAACCTTGAAAATAACTTATCTAGATCAGGCAAT
GATTTCATTTCTAGCCTTAATTTTTGTAGAAGGTCATCATTGGCTTTCAATTCTTCTAGGGCCTCTAGTC
TCATTTCGATACTGCCCCTATCAGTTAATGGCTGCAGTATACTGTTCCGAAGTGATCTTTGTCCCATTTT
AGTGGACGTGGTATCCAAGA

>Positive Sample 155
ATGAAAAATCTAATAATGTTATTTGCCGAATTTTATTATTGATTTGGTTGGTCTTCTTGATAATTCTGTG
GGCGCTTTTTTTTGTGACATATTAAAATTGCGAAATTGCAAACGAAAATATAAATATATATGAATGTTAAT
AAATAAATTATTTAATTGATGATTGAGGATGAAATACACTTTTATGATCTGTCCTTACGCGTAATAATTA
CAAGAAGGTGAGAAAATTGCCTTTTTTTGCTAATTTTTTTTTTTTGGTACCATATTCAATAATTGATGCT
AAATTATAAATTGATTTTAT

>Positive Sample 156
ATATTATTTCGAGCTTTGTCTTGTTTATATTTAGTTACGTTGGGATCAAGTTTTAAAAAACTAGATCTGG
AGTGACCAAAAAAAAAAATACCCAGCGTAAGGTAAATATTATGGATATGCTTTGGAAGAAAAGAATTTTGA
GACTTACACATTATTCGGCACAGTAGGTACTAGAAAGCATAGAATCACATTCGCCTTTAAAGACATTCTT
TTTTCTTGCTCCTTCTATGGACTGCGACACAATAGTTGTTTTTGTGTCGTACCGAAGAAAAAGCCTTGTG
CAGAAAGCAAAAATAGTACA

>Positive Sample 157
CGACTTGGATGCCGCAATTGAAGAATTAAAGGAAGCATCAAGATACATTTTAGAAGTCTACTAAATTAAT
ATAGTAATAAAAACTAAATATCTATTTATTGAACCTGTCTTGAACATTTCTATTTTTTTTTTACTTTTAG
TTTTCTTCTATGCGCAAGCTTTTCTATTGGCTGCCAAATAGAAAATTATTGAAATATGATTACATTACAA
TATTTATTTGTCTTATGAAAACTAACCATCACATTATACTAACTACGGAGGTACCATCTTGAAATCTCGT
CCTGAAGACCCTATTAGCAT

>Positive Sample 158
ATATTTGTATATATAATATGTGCACAAAATTACTACAAAATAAACAACTTTTTCTCTCCCGGAGAACAAA
GTAAGTGGAAAAAAATTATACTAATAACAATCTAAAAATGTTTATTAGCTAGGATTATACACATAAATATA
TATATATGTGTGTGTCTATATTTACATATACTAGACCGCAAAAAAAAACATAAGGGCATCACTCATAAGAA
TAATAATATTAAGGGAGGGAAAAACACATTTATATTTCATTACATTTTTTTCATTAAGCGCCAATGATAC
CAAGAGACTTACCTTCGGCA

>Positive Sample 159
TTGTCTGGAGGATCAATCGTCCTCTCAGTGCGTTTTCTTGGCGAGACAATTCAATTCGCTTTATGCTCTT
TTTTTTTGTGAATTTAGCCAATTCCGGAAAAAACATAAAAAAATGGTTATGCATTCTTTATTTGAAGAA
TGAACTAGATATACAGTAATATACTAAATTGTCAGCTCTCGGCATAATTCAATGGCCGGAAAGTAGTACT
ATTAAAAAGAAGTGCGCTCAAAGAATAACTCCGGGAATAGGTGATGTAGCTTTTGGTGCTGGAAGCGAAC
CGCTTTCATGTGTTTTGGTC

>Positive Sample 160
GCATACATATGAATATTACAACCACTCAAAGAGAAATTTACTGGAAGATTCGACAAATAAAAATTCAGCT
TTTTCAAGTGCAAGCGTAACAAAGCCATAATGCCTCCTATATTTAGCCTTTTTTGATATAACTGTCGGAG
AGTTAACAAGCGGCTGGACTACTTTCTGGAATAGCGTTCGGAATGTGTTTTACTTAAGGATTCGAACGTG
ATCCTAACGAGTGGATGCACAGTTCAGAGTTATCTAACAATATTCGTGAAGGATATGTCAAAATTGGATA
CGCTTATGTTTATGATATAT

>Positive Sample 161
AGTGCTAATGCTATCAATGTTTATTATATACAACGTACAATAGTATTATTCTCTAAGATCGTTGAGTTGC
TCATAGAGAAATCCAAAATATTGTGCACAATAAATTATATTATCTTTTTGAGACGATGAAATCTTAAAAA
TTGTCAGCAAATAAATGTTTATTTATCGAATACTAGGTCGAAACATTTTTTTTGTTTATTCAAAATGATC
ATGTACCTTCTTCTCGCGAAAGTTTTAATTAAAATTTACTGATAGTTTTTAATGATGAACTTCTATAAC
TAAACCATAATAATGAAAAT

>Positive Sample 162
AGCTATAAGTTATTGAAAGTAAGCTAGACAATAATTTTTGGTGAATAGTTGGTGAACATTCCTGAACTAC
AAAGGACTATTGTTATATTTAGTTTACTTCAATTGGCCCCCAATAGGCTTCGTAATTTTTACATGTCACA
AAATTAAATAAGGCAAAACGAGAAACAAAATATTAAAAATGTAGGAAAAAAAAAGTCAAAATTGGTTTGA
CGACTTAACCTAATCAATTTAAGTCGGAATGATGATCAACAATAACATCTTTAATTTTATCATGAATATC
TAATTCGGTTAATTGACCCA

>Positive Sample 163
CAACCACTAAACAATCTCGTTCAAAAGCTCTAATTACTTGTATATTATTTTATACGTTTATGTCTATTTC
AATGCAATTACGTGAAAAATAAAAGAACGCTACCATAAAAATGTTAAAAACTGGTTAAAAATGTCAAAAA
ATGGTCCGAAAACTGAAAAAATATTCGTGATACAGTTTGCATAAAAAAAAACTATTGTGCGCGATTAATA
TAACCTACTAGACTTAAATGACGACACTTTAATATGAAGTTCAGTAGTGTGAATTAAATAAAACAGTCAG
TTCTTGATGGATTGTACCCT

>Positive Sample 164
GGTACCTATGCAGCTGTCTCTCCTGAGAAACTGACTGAAGGTCTGAAGAGATTAGGTGATACTTTATACG
AAGAATTTGGTATTTCCAAATAGAGAGGTAAATACGTTGGAAGAATATATATAAGGTGAAAAAAGGACGT
ACGTATTTTTGTATTGCTTAAATTTCGAAGTATTTTTTCTTTTTGTAAAAGACTGCAAGTGGAAGCCAAA
ATATAATATTTTGTTTTTTCATTAAGAGTACAACTTTATACAACAATTATAACTTGATTTGAGAGCCAAG
TTATGTAAGATATTTTTCTC

>Positive Sample 165
GGAGGCTTTAACAAGTCATTAATATCTTTATTATTTTTACTTGTTGAGTTCATTAAAATTCATTTTATAA
TTTGTTGTTACAAACAGTATTTTACAAGTCATAATAAACGTTCAAAAACAAACACAATATTAAAAGAATT
GGTATTAGGATCCGACATGACAATTAACTTAGATCCTTCTATCAAGTTTCTAATCATATATTACATCTAT
TAAAATAATTTACATATATCATGCTAAAGCTTATAAAGAGACATTCTTCTTCTCGTGCGCTGGGCGTCGC
TAGCCTCAAATACAAGTGAT

>Positive Sample 166
AGTAGACCTAATATTTGTCGAAGGTATAAATGAGGAAGGAGAAGGATTAGCTGTTATGAACAGATTGCGA
AAGGCGGCTGCAAATAATTGTATACAGTTTTAAATAAAACTGTGATTAACTTTTGAAAATCATAACCACT
GATTACGGAGATTTTATATCTAGTCCGGAAAAAGAAGGAAAGAATAAAATTTTATATTGCTTTGTATATA
CGAAAAGTCGCCCATACATGTTTATCTTAGTATCCGTTGTAAATATATGTACATATATACATCTACATAT
GTTCCCATTAATTAGTACAT

>Positive Sample 167
TACGATCCGGAAGAGATTTTTGATGTTTACATATGAGCCGTTTGATAGAAATTTTGCAATTTTTTATATT
TATTAAAAGAATAAAAGTAAAGTGCAGAAAGAAAACAACATCATAAAATATGAAAGAAAAAAAAAGGCAA
TGTTAGTGTTATGAAGGGAGAAACAATTACCGTATACTGTTTGCAGGTGTCTATCCTCCACGGTATAGCA

CGTTAGGACCATTTATTCGCGCTACGGATATAGAGTTTAAAATGACTTAATTACATGAATAATACCATAA
TTCTTATAACCGTCATCAAT

>Positive Sample 168
TACCAAGCGCTGAGTTGGTCAATTCAATTAATGTTTTGAGAAGATTGATCAACAAATTGGATTCAATGGA
TATCCCACTAGCTATTTCATTAAAGGTTAAGAACGAATTGTTAAATGTTATTGTTAACTATTTTACGGAG
TCAATTCTAAAGTTGAACAAATTTAATCAAAACGGTTTAAATCAGTTTTTACATGATTTTAAATCTTTAA
GCAGCATTTTGAGCTTACCGTCACATGCTACCAATTATAAATGTATGAGTTTACACGAACTAGTAAAAAT
ATTAAAGCTAAAGTATGATC

>Positive Sample 169
CCTACGGCATCTACCTGTGTTAATTTATTAAAATTGCCAGATTATAGAAACAAAACAATTTTGAGAGAGA
AATTATTATATGCAATAAACTCAGGCGCCAGGTTTGACTTATCATAATTCCCAGAAGATTAGGGAGGTAA
TTAATTAATAGGCAGATTTCTAGTTCTTATATATTACTATAGATAAGGAAATTATACGCTTGTATGCAGA
GATAATTTAAGTTTTGCTTTGTTCGCCACCATTCTAGTTATGACCTTTATCTATCATTCTATTTTTTATT
GGAAAGGCTTTACAAAAAAC

>Positive Sample 170
AAATACTTCCCCGCTATTTGTATATATGCATATCTTTATATTATGAAAGATATTTTTTTATTTACTTTTA
GTCCGTATACTTTTATTTTGTTTTGTTAGACAATCTCGAAGAACAAAAAAAAAAGATAAAACAGAAAGAT
AAATGTGCACTCTTTTGTACCTCGTTCTGCTATAGTACTTCCAACCAGATCGACTGAAAGATTACTAGTA
TATACGTCATGTTTTTAAATAGTAGTTCCCATTCTGCTTCAGTGAATTAGATATTTTTTTCCAATCATTC
TCTTTAAACCCTCTGCGATA

>Positive Sample 171
ATGCTCCTTCTTCACTCATTTTCTTGTCCTGTTGTTGAGACATTCAACTTTGATCCTGCTCTTGTACTTT
TATGATTCATTACTTATATATTGGTCTTCATTATTCTCGAGATGAAATTCTCAGGGTCTTTTAAAAAAAA
AGTTTCAGTAGGAAAACTAAACGTAATTAAACTATTCTTCGGTTCGAACCTGAAGTTGCGTGAAAAAAAG
GGTACTGCAGTCTTGAAAAAATAGAAGCCAAAAAGAAGTTAATATCAAAACAAATAAATCTTGCACATAT
TAAAGCGCACAAAAGTATTC

>Positive Sample 172
TGATGGTGAAAATGTGGACTCTTCTGATTTATTAGTTCTATTAGAAGACCAAGTTCCTGTTGAAACTAAG
GCATGAACCGGTTAGTTCTCATTTATAATGTATAATATACCCGAATCTTATTTATTTACCTTTCCTATTT
TTTGACGACCAGTAAATACTAATACATAATTAGGAACAAAAGTTAAATAAAAAAAAAAAATAATAATTTAA
CGCATCCAATTAACGTGTCCTTTTTTCATCATTAATTTATCTACTATTTCGATTTAAATTCCATATACAA
TAAATCCTAGATACATTCCC

>Positive Sample 173
TAATTGACGCTGATTTCTAGTTAATTAAAAATAAAATATATGCAAGAATTAGCACCAGGCAGTCATTTCT
GTAAATTGCTTTGTTTTCTATTTCAAATTTGTTTTTTGTATATGTTTCAATGTTCTCGTGACAATGCAGG
CTTTTTTTTGTGCGAATTAAAGTAAAGCTAATCAAAACTAAACAATAATTTATCTTGTTCCCGAAAGAGT
GGATCATGAGGAGGAAAGCTAACCGTCCAAGATCCTATATAATTTTTGAACTGAAAACCAACTGTTCACC
TAGAAGACAGGTATCTGGAT

>Positive Sample 174
TCAAGCATGAATAATACAAAATAAATATTTGAGCCAAAGGGGATGATTTAATTATCAAGTTTTTCTTTTC
ATACTTCGGTTTACAAGCATTGTTTACGGCTCTTCTTTTCTTAGTTCTTGGCAGTTTGTGTTTTCTTGAT
GAGAATAAGAAAAGTATCGATCTTACGGCAAAACATAAATAAAACACAACTTTATCTTTTTTTGTCGCAG
TAGTGTGGTCCTACAGCTCCAAGCCTGCCGCTTTTAAAATAGTGAGCTGTGCATACTGATATATGTTCTA
TTTTACCCATTTGAGGGGCC

>Positive Sample 175
GTACTTTCTGTTGGATAGCAACATTTCCCGCTGGGATACTTCAACATGGGCTATGAATTTTCTTTTGTCT
TTTATCTTTTATCTTTTATCTTTTATCTTTAAATTATTTGAGCTCGGTTAAGTTCATAACTATTGCGTGC
CCGAATGGTATTCTTTCAATTCACAACCAAACATTAAAATGAAGAAAGCATACTCTTATATTATATCAAT
GTCTTTTTCTTTACTTATTTGTCTTTGCTCATAAATTTCTACATAATGGTAGTAATGGTGATGAAATGTT
GTTACTTGTGATATATTACA

>Positive Sample 176
GCCGATATACTCTTCAGTTTGTCATTCATGATTTATTATAATATAGACTTCATAAGTGAACTTTGTAGTT
TCTGGTGATTACTATAGCGATACTTTTCGAATAGTCACAACAAGAGCTTCTGCTTCACGTTTTTGGAAA
CAAAAGACTGAGGAGGGGCTAAATATAAATACTGATTCATATTACGAAAGGAAATAAAAAAATAACTATA
TGTAGATTTATATTAATATTGTATAATTGCAGTCCTTGTATTTGAAGAGAATGTGGATTTTGATGTAATT
GTTGGGATTCCATTGTGATT

>Positive Sample 177
CATCTTCTAACACCGTATATGATAATATACTAGTAACGTAAATACTAGTTAGTAGATGATAGTTGATTTT
TATTCCAACAGTATTTATGTTTTGTCATTCTTTTCTACATAATCTTGAAACTAGGTAGATCTACAATTGA
AAAGTAAATACTAACATTATTTACTAAATTTAAGTTAGAAATCGGCACGAAAAAAATTTGACAGATTACG
AGAGTCCAGCCAAAATATGAGTATATTACTATTTCCCCTTGGTGAAAGAAATGAAAGATGTTATTTTTTA
CCGGCTTAGTAATACTGAGC

>Positive Sample 178
CAAATAGAAGAGGTAAACCAATCAATGGCCCGTTAGTTTGCCATTTGCTGCATCCTTCCCATGCAAAGTG
TCTTCGTATTTAGTGATGTTTTGTTAGCGACACAAAAGAGACCTCGATGACAGACATTTTTTTTTCTTTA
CTTAATGTATTATAAGTGTGTCTGACCTCGACTATCATTATAATATTAATTTGAAGTTCTATTTTACATT
TATATGAGTTATTCCATTACATAATAAGGATATCAAGAGCAGATTAGTGTCTTTTAGATTATACATCTTT
TTCCCCCCTTTTTTGTTGTT

>Positive Sample 179
TTCCTTCATTGAAATGCTCATCTATTGCCAAGTACATAGAATCCACAGTGCATAGGTTTATGAGATGCTT
GGAAGATGTACGATCGCCTGCACTATATTAGTATATTTTTTCAGGCTTTACAAAACCAGAAAGAAATTAC
CGACTGTAATACTTAATTTCCATGATTTTAATCGTATGGTCCGTGAGGAAAGAGGAATTTTAGGTAAAAA
AAAACCTTTGTCTATCAAAACATAAAAGAAAAGAAAAAAATTAAATTGAATAAGTCAGCTTTTTAGCATG
ACCACAGTAATAATAGTAAT

>Positive Sample 180
TTGTAGCATCCTAAAATTTACTTCTATTGAATATATACATGTATTTATTACTTGGACGTTCTACTTTAAA
TGTAGTCAGTCGTTTTTTTCTCGATATTGTTTTTTTATTGTTTCCTAATAACTTCATTTGTTACTTTTTT
GCCTTTTGTTATTTATGGAAATAATGCAGGTTAACATAAGTAAACATAAAATATGAGAAAAACTAAGAAT
GTTTGCTGATGTATTGAACAGTTCACATCGTTGTGTCTACTATTAACACTAAGTTGTTGATCAAAACATT
CCCTAATTAATAGCTGCCCC

>Positive Sample 181
TATGGAGTAGTTGGGCTCTATAAAAATTATTTAGTTCCAAAGTTTGTTTATATTTTGTTTCTATAATAAT
GTATTGAAGATATAAACAAACATAATTTTACTGTTAACATTTAGGCGCAACAAAAGGACGATCTTTTTTT
ATTTATCACAAGTTAAAGATTTTCGATACAAATAATATTGTGGTTTAAGGTACGTCCAACTCTACTTAAA
TCACTGAATCCTTCAGATAACGAAATTACGAATAATATCTACTCTGTTTCTCCTTGTCTTTGTTCTTTCT
TGCAGCCAGCCAAGCCCATT

>Positive Sample 182
CGGTGTTTTTGATGTATGTTTACTGCTAAGAGTATACCGTCCTGTTTACTTTTTTACCTTTTGTTCCGTT
ATTGATTTTTCGCAGCAAGGTTGAAGCTTCAAATTTGAAAATGAAATTAAGACGTAGAGATGAAAAACAG
CGATGAAATTACTTTTTATAGCTTCTTTCCCATCTGACTCCGTTTTATAAACAGGGGTTCTTTAAGGGCT
TACAAATAAATATAAACACTAACACATATATATTATTCGACCTACAGTTTTTGCGACCAATGAAGTTTAC
CTATTTTGAGTTAGTGCTTA

>Positive Sample 183
GCAAGTGTATACTCTCATTAGCTCTGTTCGCGATGTGTATTTCGTGGTGCTTTATATAGGGTTTTTAACT
TTTCCCCATGAACATTCGCTGTTATGTATTCGCAACTAAAAATAATGTCTCTCTTTAATTTTAATTTACA
TCCAAAGTACGGAAATGGTAACTAAACTATATACAAAAAAAGTTCGCTCAAAAAAAAGTTTAACTATTGT
CCAATTGTGCCCATTTTGGTAAATCTGTTTTGAACTTGAATTTGCCATTAATCTCCTCATACTTCCAAGC
ATGCAACCATAATTCTAGGT

>Positive Sample 184
AAGGGTCTAGGACATTTCTATCAGTTTCTATACCAAAAGAAGAAAAAGTGGAGAATTGTTTGTTTATTTT
AATATTTTGTTCTTTAGTTTTAAAAATTCAGAAGAGAAAAAAATTTTATTTGTCACAAGGAAATAAAACA
TAAACAAAAAAGAAAAATTAAGATTTGCAATTCTGCCGCTTAAGCAAAAGTAGATCATTCACTGGAATAT

ATATATATAAACGCATTTATAATCTTGTAACGTGCACTCAATTTAGCTACGAAAGCCAACCAGCAAGGAA
CCTTTTATTTGAGTAGGTAC

>Positive Sample 185
TGGGATTGTCCGATATCAAGGTTGAGTAACGATCGTTGGCCTCGGTATCACCTCCCCCTTTCCTCTTCCT
CTTTACATATATCCTAACCACACAAGCACTCATTTGATATGATAATACTTATTCGTTTTTATTCAAATAG
ATAGCGCAGTCTTGAAGATTTACCTATATTTTTAAACTTTTGTATAATAGTTGAAATAGATAATACAGCA
TTTTTTGGCTCCTGCTTCATATCTTTTTTTTTAGGTTTTTGCTTTATATTCTTTCTTTTAACTCAACTTG
TGCGGAGCAGAGGTAAAGAG

>Positive Sample 186
CATCGATGAACAAAGGTGTGATCGGTACTAATCCGCCAATAACGCACTGATTACGTAAACGATAATATGT
TCCTGAACTCGCATTTTTTTAATGATTTTTTATGACCTCTTATATATTCTTTCATTATATAACCTCTATA
TTACATCAAAAGATGGAAAAATATAAAAAAATTAAAAAAAAAAAAAAAGAAATTTTTTCCAAAAGAGTATA
TTTATATGTATGTATACATGTAGGGAAAATAAGAAACTTTATTAATAGTAAAAAAGCATATATACTTTAT
TATTAACTCTTTTGTTTTTC

>Positive Sample 187
CAGCAGTAGTTGCAAAAGTGTCCTTTTTTACTTTTTGGGCTACATTATTCTTGAAAAAAGTTCATGTTTT
GTTATTTCTTTCTCACATACTTTATAAAAAAAGATAATTTTTTTAAAAAAAAAAAAAAAATACAAGTGGAA
CATTATCAAATAAAAAGCATTATAGCGCCACATGCGATGAACCTGAGCTTTTATCTTCATTAATATATAG
TAATTACAACATGTTATTTGAAATGCACAGCACTTATCCAGAGTTATTTGATATAAAGATTATGACTCAA
ATTCTTCTCGACAAACAAGA

>Positive Sample 188
AAGAATTAGGTGACAACGCTGTTTTCGCTGGTGAAAACTTCCACCACGGTGACAAATTATAAAGCTTTTG
ATTAAGCCTTCTAGTCCAAAAAACACGTTTTTTTGTCATTTATTTCATTTTCTTAGAATAGTTTAGTTTA
TTCATTTTATAGTCACGAATGTTTTATGATTCTATATAGGGTTGCAAACAAGCATTTTTCATTTTATGTT
AAAACAATTTCAGGTTTACCTTTTATTCTGCTTGTGGTGACGCGTGTATCCGCCCGCTCTTTTGGTCACC
CATGTATTTAATTGCATAAA

>Positive Sample 189
AAAATTTCGAACTCTTTGCAAAATTTCATCGAGAAAAAAAAAAAGATTCGTATTTGCGGGTAATATTTTTT
GGACATTTGTATTAGGAGCGTTATGTTCATTAATGTCATCCAAAAGATAGTTATAAATCTATGATTGTAT
GGTTTCTTTGACATATTTATAGTTAATATATATATATATATGTGTTTAATTTTATAAAAATTCCATCGTT
TATATTCGATTATTTTTATATGTCAACCTTTTTTTTAAGAAATGTTCATATTTAGTCAAGTTCTTAATGA
AATGAACGGTTGAAAGTACA

>Positive Sample 190
ACTCGATAATCGTAATAGTACTTTTATAAAACTTTAGTGCGGGTACTGTGAGAGTGTGCCGTAACTTTGG
TTTACATTTAAGGTGCGACCAGCAATGTCACTACTTTTACAACAACCGCCATATGGCTCGAGAATTTCAT
TATCACATGGAATGCCTGTGACAAAACTGTGTAAATATCTAATAGAAATTAGATGTAGCTGTCACAAATA
TTTACACAGGAAAGAGCCTGTCCTACGAGTATCTTACATGAAGATTCATAGAACCAATTTACTTGCGAAT
GTGAACAACCTTTCAACATC

>Positive Sample 191
TCTCAACTTACCCTACTTTCACATTCTACTCCACTCCATGACCCATCTCTCACTAAATCAGTACTAAATG
CACCCACATCATTATGCACGGCACTTGCCTCAGCGGTCTATACCCTGTGCCATTTACCCATAACGCCCAC
GATTATCCACATTTTAATATCTATATCTCATTCGGCGGCCCCAAATATTGTATAACTGCTCTTAATACAT
ACGTTATACCTCTTTTTCACCGTATACTAACCACTCAATTTATATACACTTATGCCAATATTACAAAAAA
ATCACCACTAAAATCACCTA

>Positive Sample 192
CCCCTAAAGTAGGAACAAATGTTCCTTTTCCAAAATATACTATGTGTAGTTGATCGAATATCAGTAAGTC
AAAACGTATTTTACTTGTTTATTATTATTAATACTCCTTTTCGCATATGTTTTATCACTTTACACCATAC
AATAACCACTAAATTCAAAAACACTTATTTTCAATATTTGTTATAGAAAAATAACCACTAAAATCACCTA
AATATAAAAATATTCTACCCTTTAGCAATAAGACTGAAACACTTTACTTGGGGTAAGAATACTGCCATGG
TGTCATTAACATGAAAGTTC

>Positive Sample 193
TCCAAGCTTGCAGAACAATATCGAGGAAAACTTACGGTTGTTGAACAGTATTGATTCAACGAGCAGAATT
GAGTTGGAATTGAAAAATATTGGTCTATGAACTCCGAAACTTTTTCATATTGATAAATAGAACAGTTAAT
AAAACTTATCTAGAAAGACGTTTGTTTATAAAATAATGTATAGTATCGGTACTAAATATTAACTTTTCCG
AAAAATCAAAACATCAAAGTATTCAAAAAGTGCTTGAAGAATATTGATTAGAGATAAAAATTCGTATGCA
AGGTGTTAAAAATTGTGTAG

>Positive Sample 194
GATTTTCCTTGGGTAGTAGACTTTCTTTTACGTTATTCCTTTTATAATTTTAATTTTAGTTATTGATTTC
AATGAAACAAAAGGAAACTGACGGCTTCTTAAAATAAATATATTTAACATGAAACATTAAAAAAAAATTG
TCACTACACAGAGAGAAAATAAATAAATATAAAGAATCACATAACTATGCTGAAAAAATGAAGTTATTTG
TCATGAGGAAACTTCTCTTCCAAGAACTTTAATTTACTCTTGAAAGTATCAGAATCGAAATAGCAACCCT
TTAACCATTGCTGGTTTGAG

>Positive Sample 195
TAGTTCGTGCACTAAGGACAATATTACGGATATTTCCAAAATTGGATAATACCTCTCCTTCGTTACATAG
AACGCAGACAGATGTTCGTGTAATGGTACCCGACACTCGATGAGACTTTGTTATTTTTATACGCCTGAAA
TTAGGAAGAATTTGAGTTGGTTTCAATATTCCTCAGTTTTAAGCAAAACTGTTGTCGTGATTTATTTAAA
TTTCGTTAATGAAGTTGTGAATTAGAGTGGGATGTGTTATAAAATGTCCAAATATACACGATAAGATATA
TGAAAAGAATTTTTATGCCA

>Positive Sample 196
CCACGTATTATTTGCCATTTGTTCGTTCTGCTATCCTAACTAATTGCAGTATTAATACTTTACTAACGCT
AGAAAATTTTTTCATAGTCTTGTCTCTTTTCGCTACTGCATAAAATATGAAAAAGAAATCAAAAAACCTG
ACAGCAAAATGTAAAGTAAGATACGGGCATTTAGCGTGTCACTGCTGAAGATAACGTAAGACTAAAAAAA
TTTCAAGATTAATTCCCAACCTATATATATATATATATATATATATATACTGTTTAGATCAAGTATATCTTC
CTGCTAAATAATTAATATGC

>Positive Sample 197
TTATCAATTCTTGCTTTTAGCTTCTATTGTATTTGATAAAATATTTCGGCTTCTTTTATGTGTATGCGAT
TGCGTATTACTAAATATAAATTTATATTTCCTGCGACCATTTGTCCGTCAGAAAAGGCTCTCAAAAAAAC
AACACTACCCTTTTACCGGAAATCACAAACTAAATGTTAAAATAAGAATTTAACATCATCCATTGATTAT
GAATATTGAGTGAATGATTTAATGATTGTTGCGATTTCCTTGTTGGTGAAGGCTATGATATCAGCTATGC
AGAATATACTAGTAGTTATC

>Positive Sample 198
TAACACCTGTGGCAATCAGCATGGAGTGCTCTACAGTTGTTAAGCAATCGAATAGTGGCTCTTTGTCTTC
TTGCATGTCTTTATCATAAGTAGATGGGATACCCTTCAAACTCATCAAGAATCCTGTCAGATCACCAAAT
ACTCTACCGGATTTACCTCTTAACAACTCTAACGAGTCTGCATTCTTCTTCTGAGGCATTAAAGAAGAAC
CTGTTGAATAGGCGTCGCTCAACTGTATGAAACCAAATTCTGCTGTACAATATATAATCAAATCTTCAGC
AAAACGAGAAATATGGTTCA

>Positive Sample 199
TGAAAGGTACCATTAAAGTCATATTCTAAGCGCTTGGTTTTTTTTATCTTATCTTCCTTGTGTCATATTT
AGACATTTAGTTTAGTATTCTAGGTGCTTAAATAATGAATATTGTCTATAGATGACTAATCATAAATGAA
CGAAAAGAAAGAATTCAAGAGGCGATAACAAACTTCCGCGGAGTACGATTAAGATAAGCGTCGGAGGAGG
AATTTGATATACGAGCTACAACAAGAGCACGTATATATATATATATTTGTATACGTTCATATACATCATA
TATACACATTGTCTGTAAGC

>Positive Sample 200
GGCTTACTAATTATAAAATGCATCATAGAAATCGTTGAAGTTTGCCGTAGTAATACCCAGATTATCAGAT
TCCAAATCCTTGTCAATAATTATACTCCTTTGGACAACTTCTCTTTCCATTAAAAAATCTGAAATCTCCT
TAAATTTTAAATAGATTCTGTTCAGTTCACTAACGGGGAATTTCAAGAGAACATTTTTGTTCTTCGCCGA
CTGACTATAATCTGTAACATTATTGTTATCAGAGTTTCTCGCAAAATTTTGTTTTTTCTTGCTAAATCTC
AGCATATATTTAATCAGATT

>Positive Sample 201
ATCAATCTTTCACACTAATTTTATATTAACAGTTCTACACTATTTTTGTTTGCTTGACTGTAAAAACATT
CTATTTTATGATTAGTTGCAAAACTTGAAAAAAACAATAGAATTTCATCACATAATTGAAAGATAATGAA
CTCTTTCAGTCAGTTAGATCCATGTTTTTGAAGAAAAGTTTACGAACACAGGATCTTACAAAAGGTGCC

TAAATTTGGAATTGAAAGTCTATTGCTATCAAAACTTGTCAAAGATAACATAGCTTGGAACATCTTATCC
TTTATATGTCCTGCTAAGTC

>Positive Sample 202
ATGAGTGCGGGATCAGAAAGTGAATTACTTACTGTCCCCTAATGTGCACAGCCACGGGGAATGCGGATCA
ACTGCAGAGAAAAGGGCGCACAGAGTTAGAATGCATCCCTTAATCCGGGAAGTAACAGTAACACGTCATG
CGGATGTTTCTTCGGTGGGTATGGGTCACAATTTCGTCCACCAGTACATCACAGCATGCTCTCATTTCTT
TACTTTTTGCTAGCAGATGATTACTTTCATTTCGGGTGGGATGGGCCACCTTTTTAGTTTATCCTCCGGG
ATGCCTTAAACAAGAAAATG

>Positive Sample 203
ATACTGGTGGTGAATGACAGCAAACTCTTTCTGGAAAACTTCTTGTCCTAAAGAACAAAATCAGTGTAAT
GTGTAGGCCAGTGTTATGTTTTCATGAATATGACATCTGATAAGAATAAAACAACTGGTAAAATGACTGC
TTTTTCGGCTTATAAAATTCTAACATGTACATATTTTTTATAAAATGAAAATACTATTGTCCTTAATTGT
ACAATTTTTACATTTGGAGGCCAAATTATAAAAATAATAGCGCTGCTACTCCAACTATATAACCCATGTT
CTTGGATTCAACCTTCATAC

>Positive Sample 204
GAAGGTACAGCATTTCATTTACTCACTTATTTGATCTTTTCTTTTGTTTTCTGGTAAACATCTTTGTTTT
AAATGGGCTCTCTACAACAAAACATGAAAAGTACTCAAGAAAAAAAATCTTTATTAGCGTTGGAATCAAA
AAATATATAGCATTAGCAGAACGTTAAAGAGGATATTTGGTTTCTAGTCTAAATGGTGTTCCCTTTTCGA
AACTGTATAAAATGAAAGGTAAGGTTCGAACTTCTGTGGCCTTACTTATTGCGTTCATATTCCGTGAAAC
TAGTATATATTAAAGGTTGC

>Positive Sample 205
GTCACCAAAGCTTCACAGGTTAAAAAATTTACCAAACTGATTAATGATCGTGATAAAAACGAAACAATTT
TGATTGTTGAATGGAATTGGTGTGTTGAATCAATTTTTCATCTCAATGTTGATTTTACATCAAAGAAAAA
TGTTTTGTATCAAAAGAAAAATAACTAAAAATGGACACTATATATTACGTCATCTGACATACGACACTGA
TAACAAATAAATATATAATAATTATAAAGATGCGTTGTAGAAAATCTTATGTTCAACAAACAACCCACAT
ATGATTAACAGACCATTTTA

>Positive Sample 206
AGCATTGGTACATATTGTGGTGCCACTTTCCGTAATTAAGCTACTAAATTTTTGACTCTAACGTTTATAA
CATTGAATTATTAAAATGATCTTGCTATACTATTTTGCATACATTATTTTTTGTAGTTTTAACAAGAAGA
CAAATTCTTGACTTTACTCACAAAACATAAACATAAAATGTAATAAAGTCCTCAATGTTCACCGTATTTT
TCAAAAAGGATCAGTGAAAGTGTCGGTAAAGCTACCTTTTTGATACTTTCTTTTTATGAGAATAAGTTGT
ATTTCTTTATTATGTATGAT

>Positive Sample 207
GTCAATGCCGCTAGTTTGAGGAATCTTTCCATTAAAAAAACTCAAGTCAAACCGGAGTCTGCAAGTGAAG
AAAGCAAAGTTCTTGCTGCAGCTTTAAATGATGCAAAACAAAACTTGGATGAAAACGTCTGGAGAACTCC
TATCTAAAAAACAAACGAATATAGTTTGAACACTCATTCCATGTTCTATGCTTATTGTTGTACATTTATT
ATTTATATATTCTTAAATATATTTCGAGAGATCTATACATAAACATCAATTTATTATAATTCTACACAAA
ATTACATTTAAATATTAATG

>Positive Sample 208
TTTTACGGTTCTTTTCACAAACCATTATACTTGCTAAAGGTTGTAAAAGTCATACACTTTTTAACATCAA
GATTTCAGTACAACAATTTCTTACATGTAAAATCGTTTGCAAATTAAGTTCGCCAAATTGTATCTTAATT
AAAATCTATGTGTTCAGAAGTTGAATATTGGTAAAAAGAAACAAAATGTATAAACGAACGAAAAAGGAAG
TGATCAAAATAGAGTAAGGAACCAAGGCATTAGGTTCACCCGAACGTTATCATATATACGCTCATATTAT
TCCAAGAAAGACACATTTGT

>Positive Sample 209
GATTTGGAGATATTGAAGTCACAATTTACAGTCCTTCGGACCCAAATGCATCAACAGTAACGGCTGAAAA
CAGTCAGTTTCTCTGGAGAGCAGCTATTACTCTCAATCAATTTAAAGAGTTTGTGAAAAGCTGGAGCATA
TACACTTCTTGGGCTAGAGATAATCCCTCGAAACCGGATATTGCCGATATATTCATTAACGAGCTCAAAG
AAATCTGTCATTGTGAAGATTTGAATGTACCTTTAAAAATAGAGTGGTCAACGTTTTATTACTTATGTAG
GAAAAGAGAATGAGGTTTAC

>Positive Sample 210
TGTCAAAAACAGTACGTTTATTATCTGGACAGCCAAAAAATAAGATCTATTCAAACATGGAATATAGCGT
ATTTTTATTTAATCACGGTACAATGGAGATATTTGCATGCCTATAGAAACAAGTAATAGTTATCATATTA
TTTTCTAGATTTTGTCACTGAACTTTTCCACTAATGAATCCTATCAAAATTATATATCCAATATGGCTGC
ATTCCCAACTAAATATTAAAATGCCGCTAAGTATAAAATGTCTCCGCATCGGTAAAAAGCATTACAAATG
CGTATTATACTAGCGAGAAA

>Positive Sample 211
TGATGTTATGTTATTTAGTGCTGTCCGTATGTCCTCCTGTTCTTTTTTGGGCTACAATTTAATTGATGGG
GTGTTATGTTTACGATATTTCAATTATGTTAATATTTAGTATGTTTATATCTTCTCATTAATGAATAGTT
AATTTTTACGTTTAGGTGATTTTGGTGGTGATTTTTCTATAATATTGAAATAAGTGTATATAAATTGAGT
GGTTAGTATATGGTGAAAAAGTGGTATAACGTATGTATTAAGAGCATTTATACGGTATTTGGGCCCGCCG
AATGAGATATAGATATTAAA

>Positive Sample 212
TTCACACCGGATTAAACAAAAAAACTGTGTTAATCGGATCCGGGAGAAAATGCTAATATCTCCTTCTTGT
TTGTGAAATGATGATAACTGGGCTGTTGAACGAAAATGAAACTTCTCAAATACACCTATTTATTAATAAA
TGTTAATTTATATTTTAAAAAAAGGTAAAAATTAAATGATCAGGTTTGCGATCCTGATTTCAAAATCATG
GCTTGGATTATATCTTAGAGACGTATGGATTTCAAACAAATGCCTATTTGATTGAATATTACAGATCTAC
ATTATCTCTTGTACATAAAA

>Positive Sample 213
CAAATATGATGTCAAAGGCGATAATAGTAACTTCTAAATGCATTCACATTTACAGCGATAGTTTCCTTGA
AGTCGTCGGAATACGCTCTATGCGCTATGGTTGATTTTTAATTGTTTTTTTGTGTTGAGACGCGAAAGTA
AGGAATATATAAAGAAACTTAAATTTGCTAACAGAAGAAATATTTTATAAGCTGTTTGTTTATGTATTTA
TGAAACCAAACAAAAAATTGCCTGTATAATGAAAGAATTTTCATATTTGATAAGCTTCTATGTAATTAAG
GAAAGGAACCAAACGGGGAA

>Positive Sample 214
CGTCATTGGAAAACTCTGAAAACGAATTAGGAAACGTCATTGAAGAGTTACGAAATTTGGAGTTTGAAAC
TGAACATAACGTAACAAATTAATAAAATAAATGATATATAATACCCGAGCAATCTACAGCAAAGCTCAGC
CCCACCTTTCCGCATTTTAATAACCTGCATTTTGTTCAATTCCACCCTCACTCCCCTTCTCAAGTATTCC
AAACCAATTTAAGTAAGTATTGATATGTAGTTATTTTTAAAATAAATATATGTAAGTATATTTGGATGCT
TTAATTAGGAATGTTCTATT

>Positive Sample 215
AATAATGGTGATAAACTGCAATAACAAAAGAAAATAGTACCAATCCTGGATCTAGTTTTCCCTAAAATCC
AATTTTTACTTATCAGCCTCACTTCCTAAACTACCTCACCTTCAAAAGCTCGATAATTAAAAGAAAACAT
GAATAATATAGTAAACAATGATAATAGTTTCTCTATAAGATTATCTAATTTTCGAACAGTATTAGCTATA
TGCGAAATATCTTCATCATTGGTACCTTATCATAAACCAAGGCCTGCTTTATATTTATTTGAACGAAAAA
ATTAACTTATATTCTTGGCT

>Positive Sample 216
GTTTCGACGTGAAGAAAAAAAGTCGTAAAACTAAAAAGTCTCTGCCCCCACTTTCCAGAAACAGATATCT
ATATTTTATAACAAAGACGTACGGCATCATTTAAAGTATCTAAAATTTCTTTGAAGAGATCACATAAAAA
ATTTTCACCTTCTTTTTCTCTTTTTGGCTGTCCAAGTAAACAAAACATAAACTGCTTCAACCAAAGATAA
AACTTGAGAAAATCCAAAATGAACAATAAGGACATTTCTTGGTGACAGATTCGTTAAGTTTCATTATTTT
TTTCACTTATTTTATAGAAA

>Positive Sample 217
TGGGTCGACGCCTTCGGAATTATTTACGGAGCTTTATAGTAATTAGTCAAGGTTTTTTTTTCTTATTTT
TTTCTATTTCATAACTTAGTCATATCAAAATATATCTATTAAATTATTTTCCATATATACGTAGATATTC
TTCATTTGCATAATTCTTTGGGAAAAAGGAAAACAGTTATAGAAAAATATAAACAAAAAACCGCAACACT
TCATGATTGCGTCGAACAATATACCTTAAAGAAGAAAAAAAAAGGTTGGTTTTATCACTTCATTAGTCTA
TTTGAATAATTCATAAAATA

>Positive Sample 218
ATGGCCAAGTTGGTAAGGCGCCACACTAGTAATGTGGAGATCATCGGTTCAAATCCGATTGGAAGCAATT
TTTTTCTTCGGCACGTCATACACCTTGTAATGAGTGTACTAATTCTCTCTTCAAAAAGATCATTGTATGT
TTTATGTTTTGTCTGGAAAAACATATAGTACGGATATTTTTGAAAAACTTTGAAATAATATGTAAATTAT

GTAAAAAAATTAAAGAACCATTATAAAAGAATATTACCAAAAATGTAACAGATGACATTTTTATTGTTTT
GAGTTTGTTTTAAATTTGAA

>Positive Sample 219
AGAGAGGTGACAACGTCTTAGCTTAATGAGATTTTATGTAGTTAAATTTTGACTTATTTAATTTATGTTT
TGTAAAGAATAAAATGAGTATGATGAAGGAATCTTACCTCTATTAACTATTTCTTCTTTTTTTTTCCTTC
GATAAGATGTGCGCGAATTCAATATGGAAGAAAGCAATTTGGTATGCAGATGGTGTTCACCGGCAGTGTT
TAAGTTACGAATATATAAAAACAAATTCAGGAACCCGCCGAATTTGGCCCAAACATATACCTATATACAA
ATTAGTGACCATACGCTATT

>Positive Sample 220
GGATTTATGAGCAAAACAAGTAGGCGAGCAAGACATGAACGAAATTGTAATGAAGCGTACTTCCTTTTCC
CTACAACCTTAAAAAGGTCTCACTGATATATCCATTCGCAATAATGACAAGATTTTTGCATGGGACTTCT
TTTTTTCTTTTGAATAAGTCGCGTTATAAGGACAAAACATTAAAAAAAAATATGTATTGATTCACTGATT
TATATATACAGAAGTAAGTACCGGGGTACCTAAATATACGCATAAAAGTCTCTTTCTTTTTTTTTTTTTT
TTTTTTAGCTTCCTACATTT

>Positive Sample 221
GAGCGAAACAAGTAGAAAAGCAAGACGTAAACGGAATCATGGTGGTGTGTACATTTCTCTTTTTTTATTA
CACCAAGAAAGCTTAATTGGTACGTATTGTTCGTGATAGTAGCATGAATCTAGCGTGGAATTCTTTTATT
TAAGGTCGCGTTGTGACGACAAAACATTAAAAAAACCACATGTGTTGATTTGTAGACTTGTATACACTCC
AGTAAGAAGGTGCACACCAAAATACATGCGCATATAAATTATTTTATATAGCATGCTATATTTCGTAAAT
TCATAAAATTTCGTTAATTC

>Positive Sample 222
GGTTCTCCGCAGAAATTCTACTAAATTACGTATAAATTGTGTATTAGAAGAAATTCAGAAACGAAATATC
TGAACAGACCTACTTTAGTATTTTTATATGATAACTTTTTGAGTTGTATCCTTTTCTTCATTTATTTTTA
GAAACTTTCTTTTTTTGGCCATAACATAAAATTTAAATAACGACGCTGAAACAAAAAGAAAAGAACACCA
CGACTAATAAAACAAAATTAGAACATTATGATTAGGTACTTCTTCAGTACGCTTTTCAAGCTACTTCTTA
CGATAAGTAAACTTCTCACC

>Positive Sample 223
GGTCTTGTTTGTTTCTGCTTGGCTTTCATAGTAGCAAATAGTTTCGACCTTAATGTAAGAAATAAAAGCA
ATCGTTAATTTGAGTAAAGATAACCACTCTTCCTCTTCTACTATCAATATAATAAGAATAACGCTTATGG
TTTATTTTAGCTTTTTTGAAAGCTAAACATTAAAAAAGAATAAAAATGCACGGCATACCTACATATTTTA
AAAACATATATTTTATGATTGACTTTAGCAAAAGTTGGCGCTGGGTACTTTGAGACTCTTTCCTACAAAA
CGTTACCAACACTTGCTAGT

>Positive Sample 224
CTTCATGACAGAAAGGTTTTGCATTGGTAGGCGTGGAAGATTACAGATTGTACTCGTCGTTGGCTCAATT
TTTATATTTTGTTGCTTCAAAAACAGTGCCGTATCCTTAATGAATGAAAAATTCTAAAGAAAATAAGTTG
ATACTAAAAATATAATGTCCTTGAAGTATTTATGCGATATATAGAAAAATCTATAAATACTTAAATATTA
CAGCAGCGTTATTTTTGTATAGACGCCGACGTATAAGAGTCTCCTATCAAAAATCAGAATCTGAGCCCAG
TATGTCATCTAATTCTGCGT

>Positive Sample 225
AAGAGTTTGGGGGTACTGATAAATACTTAACTACTGTATATTTTAAACCAATGCACTCTTGGCACTTTAA
AAGTAAAGGTATAAATGTAGTTAAAAGTTTTTGTTTTGATAATTGAATGTTTTCATTTCGCTATCCCCAC
GCTACTTTATTTACTTCTTCTTCTTCACGACAAAACATTAAAATTTACACTCTATAAAAAACAATCATAT
TTCTATTTATTTTTGTGAGTTGACAAATCTTATCATAGTGATTAGAATCTGTAAACCGATCTCAATCGTG
TCCAGAAACAGCGTGCCATC

>Positive Sample 226
ACTGGGTGATCAACAATGCCAGAAATAAATGAGAAGCTTTTTACTCTTGCTTTTGCTTCTTCCTCTTTAT
GTTCAAGCCAGCCAAGCTATGTTATAGTGTTGAATGTCTTGAACCGTTATAGTTTCTAAAAATAACTTTA
CATTTTGTTATGCAATCTTCATCATCTCGAACTTTTCAACAAACGTTCAACAGTGATGGTCTTCCCCAGT
GAACCAAAAGACATATGACGCTCGTATGGTATGTTAAAAACAACATAGAGGATAGTAGGTCGGGGTTTAT
TGTAGAAAGGACATAAATAG

>Positive Sample 227
AGGTAGGTCTTTTAAACGAACGAAATTGTTCACGCATTGTATGTTGGGTTTCTTACATATCAATCACTC
AGAAGCCCATTTTCTACCTCCATCACCGTACTTATAGAATTATTTTCTTTCTTGACACAATATACTGTG
GCTAGTTTAACATTTCGTGCCCTTTAAATATTTTTCATGGTGTGGACAGAACAACGTCAATTGTAAGGAG
CATGGAGGTCAAATAAACAATACCTCAAACTAAATAATGAAGACCCCAAGAAGCCTTGCAATCGGCAGTA
ATTATATTCATGGTACTAAT

>Positive Sample 228
TTGAAAGTTACTTTCTCGGAATGGAGTCTCACAGCTCCTTTAGGAAAAGCTTTACATGATTTGAGTTAGT
TCACCTATATATCAATATATAAAGAGGGGTTTGCTTTTGTTAAGAACAAGGCCTGCACCTCACTAAGATC
TGTTGATTTGGCACTATGCTTTTTAAAATTTTGTTTATACTCAATTTCGTCAAGATTTTTTTACTAGTA
CAATCCATACAGTTAACAAAATACTGGGCAATAAGGCGTGCATACTACGAAATTTTTAGTTTTGTTATAA
TAAACGACTTTTAGCTAGTG

>Positive Sample 229
TTGGGTTCATCGAGTACGTCTGAAATATCTGAGGGATGCCCAGAAAACGAAGTCGCTGATTTGAATTTAA
AAAATGGTGTGACACTAGAATCGCCAGAATAATGACATGAGTTTATATGATAGATAAAATTAATAAATTT
CCACATGTATAATACTTTTCAATCTTTTTCGCCTACTATTTACTGCCTAGGCTAATAAATTATATGTTTT
TGTCCTGGGCGCGACATTCCAAAAAAAAAATAAAAAAGAAGAGAAAATACTGGCTTATGAATACAAAAAT
TACATAAACGAAGCAAAATG

>Positive Sample 230
AAGACATTGCATTCGGTTAATGAACACGTGGATGTCCCAGGTCATTTATCTGCCATTGCCTTTGTTTACG
AGTATATCGTTAATGTTAACGAATACGCTTAAGCGCAATCATTGAATAGTCAAAGATTTTTTTTTTTAA
TTTTTTTTTTTAATTTTTTTTTTTTCATAGACTTTTATTTAAATAAATCACGTCTATATATGTATCAGT
ATATAACGTAAAAAAAAAAACACCGTCAGTTAAACAAAACATAAATAAAAAAAAAAAGAAGTGTTCAAAT
CAAAGTGTCAAATCAAAATT

>Positive Sample 231
TTGACTAATGATTTCTTCCAAGGCGTTGATCCCAGCCGTTTCCAATAAATAGTCCATCTCATCTGAAGAC
ATTAAACGAGTTGTTTCATCCTCGTTTGACATTGTTCTGCGCCTTTGCTTCGCACGGTTTGAAGATGTAT
ACGTGAAATGCAAGAACTAGGGTTTGTATTCCTTATAAGTCTATTAGACTTCTTGCAGATCAGCCCTTCA
TCTTGTTGGTTTTTAGGAATCCCTTACCCTTTATTTTTTTTTTTTTTGATTTCGATTAAGGGCGGGAAAT
CATGATAAACTTCCACGGAA

>Positive Sample 232
CTTTATATATCTATGTTTAGTATACCCAAAGGGTATTTCGGACGCAGGCAAGCTGCCATTATTTATAGCG
GAAACTCTAAGAGTGGTAAATCTATTTCTAAAAAAAAAAAAGATTCATTCAAGCGCCCTCCTTGGTTAGT
TAGTTAGAAACATGTATTCAGAGGGAATTAAATAAAGATGAGTATAATTAGTTCTTGGTTACTTGTCTCT
ATTATTTGCTTAACTACTTCTATTGTAACAAAATTGCAAGCTGCTGGAGTTACTACCCATCTATTTTATT
TAACAAGAGGTGCTCCCTTA

>Positive Sample 233
TTTGCGTCATCTTCTAACACCGTATATGATAATATACTAGTAACGTAAATACTAGTTAGTAGATGATAGT
TGATTTTTATTCCAACAATTATCATATACGGTGTTAAGATGATGACATAAGTTATGAGAAGCTGTCATCG
AAGTTAGAGGAAGCTGAAGTGCAAGGATTGATAATGTAATAGGATAATGAAACATATAAAACGGAATGAG
GAATAATCGTAATATTAGTATGTAGAAATATAGATTCCATTTTGAGGATTCCTATATCCTCGAGGAGAAC
TTCTAGTATATTCTGTATAC

>Positive Sample 234
CCAATCGGACAACACTAGCTCTATTTATACTTTTCTGTTGCTCCAAAAACAGGAAAGAACAAATTTTTCA
ATTTAGTGTGACCCGGCTAATAATAAAGAGGGAAGTAATAGGTAGGGTTTTACTGGCAAGAAATCCATAT
TGTTGCTCCCGAATAATATTATAAATTCTTGTAATTAATACAAAGTTTCTAGATCCACCTTGTTTAAAAA
TATAAAGTTATGCTTAAAATTCTTCAGGGAAGAAGGTTTTCAATGGATGATTTTACATTTTTGACGAATT
CCTGAGAAACTAAATTACAA

>Positive Sample 235
AGGGTTAAATGATAGTGAGAATAAATATATTCTGGTTACCAAAGATAATGTTAGTTTTGTTCCGCTAAAC
TTAAAAAGTGTAGCAAAATTATCCAGTTTCAAAGAATCTGCTCTCACAAAATTGGGAATCAATCACAAAA
ATGTCACTTTCCATATGACAGACTTTGATTGCGATATTGGTGCTGCAATTCCAGATGATACTTTGGAATT

TTTGAAAAAAAGCTTGTTTTTGAACACTTCTGGAAAAATTTATATCAAAGACCAAATGAAGCTTCAACAA
AAACCGAAACCTGCTCCTCT

>Positive Sample 236
TGGATTTGCATCATTAATAAAAGATTTCAAGAAAAAATGATACCAGAAAGTCTCAGTAGAATACACGTCA
CAGAAAGCTAGTAAAAAAAAAATAATCAAGCATATAAAAGTATTAATATATTAAAAAATCTTCATGTAAAC
CAATTCTTATTTACCTCTTTTCCCAGCAAGCGGGCAACTTCTTTAAGAAAAGTAATCTTTTTTGTACGTC
GAATTATCAAAGCTTGGTTACTATATTTAACATTATTGTTTAGTAACAGTATCATCTTAAAAGAAAATTT
TTTAAAATTCCAATATAGGA

>Positive Sample 237
TACAGTGCTTCGGAAAAGCACAGCGTTGTCCAAGGGAACAATTTTTCTTCAAGTTAATGCATAAGAAATA
TCTTTTTTTATGTTTAGCTAAGTAAAAGCAGCTTGGAGTAAAAAAAAAAATGAGTAAATTTCTCGATGGA
TTAGTTTCTCACAGGTAACATAACAAAAACCAAGAAAAGCCCGCTTCTGAAAACTACAGTTGACTTGTAT
GCTAAAGGGCCAGACTAATGGGAGGAGAAAAAGAAACGAATGTATATGCTCATTTACACTCTATATCACC
ATATGGAGGATAAGTTGGGC

>Positive Sample 238
TTCCATTGTTATTAAAGGCTATAATATTAAAGGTTATTTGGGTCTTTCTCTCTCTCGGCTTTATTTTATT
TATAGTTTCTATCTTTCAGCTTTCAAAAAATAATATATATGGCGCTTCATGAAAAGAGAGCTAGCTACAG
AAATATAAACAGTCATGGATGAATAGGCGGCGGCAGCTATGCCGGTGCATGCGCACAGTATAATGGACTT
AAACTGCCTTAGTACAACTGTTCAAGTTATTGAATAGCATTATATATTAGTGCCAACCAAAATATGAAAC
CGATGCTTCCAACTGTCACC

>Positive Sample 239
GTTGTATTGTGTTAGTTCCTGCAATCACAACAATGGTGAAATAAAAATATATGAAAAGCTGGAACCTCAT
TTGAATTAGTTTACTTCACTAGGCTCTTTTATCTTCACTTATTGTTTTGTGGTGTCTATTTTTTTTACTG
ACAGTTTACACTTTTATAGTCTTTAAAAAAGCAAAAAAAAATAATTCTTTTATTTTTATCCCAAGATAATA
AATATTTATGTATTGGATAGTAGTATAATTGAGTATAAAAATTATTTTTGTAGTACTTTTTCAAACTAAT
CTCTGCAAGACATATCTTCC

>Positive Sample 240
AAAGATGGCAGTCATTGTATAGAATACCACAATTTGTGAGCATCTCTGAGGTTTTTTACAAGCTTCCGTA
ACTCATACAGACTTCTATCGTCCTACTCTAGTATTATTTACCTATTCATAACCACTTTTTTATCCTTCTA
TTTAAATTTATAGATGCAAAGGGCGGAAAGAATTGACTGGAAAGTAAACTAAATATAAAAAATATCGCCA
ACTTATTCATACAATATCGTAGCAACTGACAACTTTTAAAAATCTGCAAAATTCTTTGTAGTGTTCCTTA
ATAATGTTTAAGCTAAGGTT

>Positive Sample 241
ATTGAATGGCGGTTAGCTGTTAAGCATTTGAAAAAGTAGCTCTCATCGAACTCTTTCATGGTGATGAAAT
TTTTTCATCTACTTACTTAATCAGTGGTCTACTGAAACAGCCTTCGGGTAATGAAACGAGCGGTAACGCT
CACAAATTTTTCTTTTTACGAACAAAATATAAATTTAATACTATGATAAAATCTGCGGTGAGACGGAAAA
GGATAAGAACTAGAAAAGTATTATTAGAGTTAAGATATGAACTGTCCATTTAACTATGGTTGAAGAAAAA
AAGAGCCAGGATGAAAATTA

>Positive Sample 242
ATCGTTCAAAGCAACAACTTCGACGTTCTTTCTTTGCAAAGCAATTCTCATAACCAATCTACCGATTCTA
CCGAAACCGTTAATAGCAACTCTAACCATTTTGTTTTGTTTGTTTGTGTGATGAATTTAATTTGAAACTA
AGTTCTTGGTTTTTTTAACATTTAAACAAACAATAATAAAATGAATAGACACACAAGAAAAAAACTATCT
TGAATGAGTGCAACCTTTATATATTCTTCCAAACCTACAAAAAAATTGCAGGTCTGCGATCACTGGGTAC
TATCAAAACACATATTAGCA

>Positive Sample 243
TCTTGTTGTTTTCGGATATTCCGTCATTGAGCAAAAGTTTGGTTTGTAAAGACGAAACTGTACTTTCATT
AGATTCTCGTGTTTCACTCATACGCCTTTATATGCTATCACAGTTACCTTATTTGTCCTTTTTCTTGTAG
AGCTATTCTCTATTCTGCTTTGACTTAACTAGCGTGAAATTAAAACCAACGCCGCTTATCTAATCACAAT
AAAAGATCAGACTGTGAAATTAATAAAAAGGAAAGGATAAAACGACAGATATCGCACTAACTTTGTTAGA
AACATTATGCAGACAAAAGA

>Positive Sample 244
TCTGCGAATTTCATACTCCATAATAGAGATTTTGATGCCGAGTGACAATATAAATGGATACTTACAGAAT
GAAAAACATTTTTTTGTCGTCGAATTTCCCCACTCTTCAATCAGTTGATTCATTTTTGTCTTTTGAGCTA
CTCCTCTCCAATGATCGAAATCTTGTTTATAATCTATGAAGTCTAGCGTTATATTGTAAAAGCTTTGAAT
TGACAACGCTCCACGGCCATTTCTTCTAGTATTGGCTACATAATAGATGAACGTCAATTTTGCTGCAGAG
TGAATATGCCAGTCTTCCTC

>Positive Sample 245
TTCAGAGCAATACGTGTTTATTTTTTCACAACTTCGCATGGCATTATGTATCATTGCAGCGTAAATTTTT
ACATTTAGTAATATCTAATAACATATACACAAAAAAGGGACAGATTTTTGAATTAAATAAACCAAGCGTA
AATCAGTATTGCTCTTACCGCAGTAGGGGGAGTTATCTACTCCTGCTTAACGTAGAGAAACAATAGTAAA
AGTTATGCTTGTGTTGGAATAAAAATCAACTATCGGCTGGCAACTAATAGGGACACTACCAATATATTAT
CATATACGGTGTTAGACGAT

>Positive Sample 246
TTAATTGTGACGAAGGGCCCAAATTTGATGGGTCGGTGTTAATGATTAGTCCTCATTGTCATAATAAAGT
GTGATGATGGAGGCAATGATGATATACGGTAGTACTACTGCTCGAGGTGCTATCTTTTAACCAATCCTTT
GAGATTCTTGTCGCCACGGAGTTACTACCTTTTACAAACCGTAATGTCACATTTTGCATATATCTTATGT
ATAAATATATAGTTCACTTACTACTTGTTCTCGTTTTGTTAACTTTCTTGTTGTAGTTCTTCTTGTTCTT
GGCGTTTCCCCCTTTGTTTT

>Positive Sample 247
GGTTATCAATTATTAACCCAATGTAGCGCATGCAGCTTATGGCTGAAGGTTAATTTTGACATCGAGGAAG
AGCAAGAAGGACAATAATGAAAATGGGAGGGGAAATTGAGCATATCAGATAAATCTGTTTATAGAATTAT
TATTTTACTTCGTGGGAAATCGAATGGTGTATATAAAAGAGGTTGTAAAATTGACGAAATAAATAGTATT
TAGGCAACTAAAGATAAAAAAATATTATTTATTTTTATTCGCGGTGCGTTGCCAGATTTTTTTTGACATG
CGGAATTTTGGTAAAAAGAA

>Positive Sample 248
AATGCTAGGCAAAAATAAACGAGAAGGAAGGTGAGTTTTAGTTCATTGACTAAAAATGAGCAAAAAAAGT
CAGGAGTTTTTGTTTTGAAGGGCACTTCAACTCTCGAACTATGGATTTACAGTTTCTTCCTCGTCTTTTT
AAGTAAAGTATTTTCAAATGACCAAAATGTAAATAAATTTGAAAAGGGCCCTTCTTTACGAAAAAAAGAT
CAAAAAAATGTTCACAAACTATTTCACGAAATCAAGACAAACATATAAAGGAGAAATACAGTGGAGATTA
GATAGTAATGTTCGTAATAC

>Positive Sample 249
CCCGGATTCATAAATCCACGTATTTTACCTGCATCTTTATTGAGTGCTTAGTAAAAGTCAGTTGGTGAAA
TTCCGCAGATAAAACAGCATATTAGTTGCCTGAAAATAGCCTTATGTGTTGGTTTGATACGTCTTTGTCA
TTATATACCCAAAGGTTTGTAGTTCATGTTGCGGGTTGGTATGAATAAATTTTTCGTATTTAGTGATTAT
AATACTTATTACCCATTTCGGCGGCTAATAAGGAATCTGCTAAGATAGGTAAATGTACAATACCATCAAT
GGCTTCTAAAAGTTTTCAAA

>Positive Sample 250
AACGGAGGTAATAAAATGAAGGATGAGGGAAAAAAAAAACCATAATTTTAGGCTGAAGCCCATATTGTGA
TATTTTTAATGTTTTCTTAGAAAAATTAAAAAACTTCTCAATTAAATGTAAAATTTTACTTGTTGGCGAT
ACCTTAGAGGATTCAAATTTAACTAGTGTTAATATACGAACTGGTAACACTAATGTTTAGATACCTGTAT
ATCATACAAACCATTCAAAGTGTATAGGAGTATGTTCACAAACCGTAGTATTATGTGCTACATGAATCAA
GTATAGATTTAAAATTTCTT

>Positive Sample 251
GCTTAATCGCATATCCCAGTTGGCCCGAAAGAGAATGCACATATACAAATATATATGTAAAGGGAAATAC
CTTGTGACTACACCTATGCCTAAGTTTCAGTATCTGAACTAGGATAATTTCGATTATAACTCTAACATTT
TTTACGTTTTCTCCATATTTCGAATTGTTCCTTCTTCAATCAAAATAATAAAAAAAAAAAATCACAAAAA
TGACTATAGTATTTTGAGCCCAACATGACTGCGAAATTGCTATTAACAATGATTAGTCAGTAGTATTGAA
TAACTTAGAATTTAAAACTA

>Positive Sample 252
ATTTGAAGATGCAGCCAAGGCTTACGACTACAACATTGCCCATGGTGGAGAGGTAGTCAAGACTATTATC
TTTGGTCCTGAATGAAAAGTGAATACTTTTCGGCACTGGTTCATGTCCATATATATAGACCAATTCAAAA
GCAGTAATACTTGAAAATAACACCGAAAAATAAAAATTTAAATAGTAGACACGTTTAATGACTTAAAAAC

TAACTTTTTCATATCTAATATTGTAAAATGGGCGGAAAAAGCTTAAGAATATGTTATTTTACAAATCAGA
GCGCTGACACATATAGAGAG

>Positive Sample 253
TCTAGTAGAAAATAGACGTAGCTGTCACAAATATTTACACAGGAAAGAGTCTGTCCTACGTATCTGACAT
GAAGATTCATAGAACCAAGTTACTTCCCAATGTGGACAACCTTTCGATACCATGTCAATAGCTTCCCCTT
ATGTTCGGTGTCACTGTAAAACGGATCAAAACAAAACCTAGAGGTACGGTGGTACTCAGATCATGCATGA
CCTCTGGGTAATTAGCACTTCTCCCGCTTGTTTTGAGATTCTGTATATAAATATTGCAAACAAAAGGATA
GAGCGCGGATGGCAGCCCTT

>Positive Sample 254
ACTCAATTGGTTAAATTAGTGACTAAGAAAGATATTCCGGCACATGTATCTACAATGATTCTCGAAATTT
GCGCAGATGACAAGGAAGGAGAGGATGTTGAAGTTCCTTTCATTACCATTCATCTATGAGTAACTAATTA
ACCAAAATAAAAAAATAAATTAAATATATACATTAAATATGTATATATATATGTATGACTTTTGACAACT
TTTTCTTTGGTAAAAACTTATTATTATTATTATTATTATTATTATTAATGTAGTCTCGAATATTTGAGTA
TGTTTTAGTTTTTTGTTTTA

>Positive Sample 255
GTTCTTTTCCTTTTCATTTTTCATCTTTATTATCTAGAGTTTGTATATAACATGTATCCTATATTATCTT
ATCCTATCATCATCACTTAACTTGTTATAATTCTCCCATATACTTATGTTTTCGCCTTTGATATTTACTA
GTCGAAGGAACTTTTTTCGCAGTTAGTAACGTCACGCTCGGAACTAAACATTAATGAATGAGATGCATTT
TTATACAACACTTTTACAGGCTATATACAACTAAGTGATTTTCAATACATTAAAACTACCAGAATGGACA
CCAACCGATATAATGTTGGC

>Positive Sample 256
TACGCCATTGTACACAAAAGTATTTTTTTATATTTTTTTTACAATTTTTTATAATTTTTTTTTATGTTTA
GTGTATATTTGCTAGAAAGAAACAAGATCTTTTTTGGTAGTGTAAATGCTTAAACTTTGTCTCTTTTTCC
TTTTTACTTTGGAAAAATGCCGACGCAAGAACATAGACATCTGCGAAAACCAAAAATAAAGTACCAATTG
TACATACAAAAACAGAGAAAAAGTTCATGAACAGAACGTTACTACGGTATAGCAAGATCTTTGAATACAT
CCATTACTTCGGATTGTTCA

>Positive Sample 257
CACTCTCATTCCCCACACATAAACTTGTTTTATGGCATCCTTTTCATTTAGCATGTCTTTATTTCCAAAC
CTTTCCTCGTTCTTTGCATTCATTTAGCGTTTGCTCGAGAAAGCATCACGTTTTCACACATTATCGTTCG
TCGCTATAATAAAAAATAGTTATAGAATTTACTCAGATTTACATGTCGTACCTTTTTAATTGTAAAAAAAA
AAATTTTATGATACATAATTACCTAAATATAATTCAGAATCAAACATACTTATAGCTATTTGTATGCTAT
TAGGTGGTCCTGCTATAAAA

>Positive Sample 258
GTTACTTAACGCTCTTCAGGCACTTCCTTATTTATTATGACACGTACAAAATAATAAATTCGTAGTTATA
AGATTATCTTATCATATTTAAAATATCTCTTCTTTCAGTTGAAATTGGGCCCAAAATTTTTACCCCATAG
CGTTAAAATTAAACAAAAACTGCTATTTCGGTGACAAATTGTAAAAATTCTGAAAACGGAAGAAACAAAA
ACGTTTAAAATGATTAAACTTTGGTAAAGCAATAATCTACAAATAATTGGAAAAGGAATAGTAATAAAGA
TGGGGAGAATTATGAAGAAG

>Positive Sample 259
GGTATTATGCAAAAGGAAAATTAAGGAGGGTTTACTGTGCTTTCTCTTTTTTTGAGAAAAATGTCTTGAG
TAAAGTGAGGATTTTACTGTCAAATCTTTAAAGCTTGTTTTGAACGATGTCCTGCATTAAAGTGGGAGTT
TTTCAAAACGGGAAAATACAAACAAAAAATGAAAAAAAAAAAAAAAAAAGGGACTTTAACTTTCTGCGAGA
GTATGTTTAAAAGTATATGTAATGCACTTTTGGTATGTTAAGAGGCATGCACGAAGAACCATTCTATAAG
AGACTAAACATCTATATATT

>Positive Sample 260
TGACTCTAAAACGGCATTTGTATATATTCCCAAATCGCGGAACGGATTTACACTAATTAACACATGGCCA
ATATAGGTATAAATAATTCCATTCTTAAATCTTTTTTTCAAATTTTCATTGATAGATTCATCAGATATTT
TTGACAAAAGTGTCAAATCAGAAATACCTACTTCTTTCTTTTTATTTGCATCAAATGTAGCCTTTTTGAT
TTTTGCAGATCTCTTCTTGGGCTCCTTAACGTCCTTTCTGCGTGCTCCCTTTTTTATGACAGCCATTGAC
GGTTATAAGTATTTCTCTTT

>Positive Sample 261
CAGGCGTCATTTAATGTTGATAATGGTGTACGGGAAGTGACTTGTAAAAGGTAGGGGAAACCGTCAGTCT
ATCACTAGTGGTGTATCTGATGGAAGTGCCCTTTTATATTTTTTGCCGTACACAGGTAAAGAAATGAAAG
TACTAATTATCCCCCATACTCTTTGTTTCTCTTGTGTTGTTTATGATCAATCCTTCTAATTTAATCATTC
AATAACTAAATATTTAAAAAGATGTAATTGCCGCAAAAAAAACTCTCAATTATTTACTATTTTGAAACAG
TCTCTCAGATAGTATGCGCC

>Positive Sample 262
TTAAGTGTGAGGAGGTTAATGAGTTTGAGAATCCCCTTTTCCTGGAATCTCTCGTAGTGAAAAACGTACT
AAGTAAAGTCGTGCCGCCCGTGTAAGTTTATTATGTATTTCGAGCATGCATCTGGAACCTATGTTTACAT
ACGTACAAACGGTATTTAAAAGTTTCTATTCTTAGCGTCAATTGCCCTTTATCGTTTTGTTAACCAATTG
TTGTTTTTATTCATTATGCCATGACCCGAAAAATGAGCAAAATCGGTCAAAGTAAATAGCTAAATATTTG
GTCTAAAAAGTAAGAGAGAA

>Positive Sample 263
CCTGACTTCGATGACAGCTTCTCATAACTTATGTCATCATCTTAACACCGTATATGATAATATATTGATA
GTATGACTATTAGTTGATAGACAATAGTGGATTTTTATTCCAACAGTTTCATACTTATTTAAATGTTTAG
GTTGATATATTGGTAATGATATGTTATCTTACTCTGAGATGCAACATATATGCTACATACTGAACTCATT
TATCCAATATTAAATTAAGAGATTGGTGAATTTTGAGGTATTTGTTGGCAGATCTAAACTTCATCATGCT
CTTCTTTTTCTATGTATCTA

>Positive Sample 264
ACCGCTTATACACTACATGATAATGAATATATCAAGTCGATCATCCTTTTTTATGTTTTAAAATACTTTA
CATACATAACTGTGTTTTTACTTTATCTAAAAAAAGAGTTGTTATGAATTTGTGATTATTTATATTTCCA
AATATTTATGATCTCAAAACGTAAAAAAACATAAAAAAAAAGTGTTAACCAAAGAAATTGAAGAAAGATA
GATAAATAAGTTCCAAAAGTTCTATGAATTTTCAGGTTGCATGGCAGATAATGTCTTCTGCTAGGCAAAT
GTCAAACATGAACTGAATTG

>Positive Sample 265
TTTTCGGACTCATACCATATAGAAAACTATTTTTATGCTTCTTCATTTACTTCTACTTGTTTATGTTTAT
TTGTAAAATTACATTATTTAATTTCTTGAAAGTAAAAAACTTGAATTTCTGAAATAAATAAAAACTAGAG
AAAAATTAACGATGAGGCATGCGACGGAAATCAATATTGAAACTTCTCAAAGCTGAACCACTGAAAGGGT
TCTTTAGGACAATTTTCATTCCCTTAATCAACAAAAAATGACTAAAACCTACATATTCTTCTTAGAAAAA
ATACATACGCAAAACAAGAT

>Positive Sample 266
TCAATCTTCATTTGTTTACGTAAAATGAAAACGTTTTGTGTTAGAATTGACAGATTGAAGTTTAATTTTA
GGTGTCGGGAGAAACATTTTAATCCCTTTTTATGAATATAATTTAAGTACTAAAAAAATCACCATAATTA
TACAAAGTAACTGCAATTTAAAAGTACGTTTATAATTAATTCACATACACATGTTATTGTATCTAACTAA
ATAGTCTCGAAATAGTTCAATTGAGTACGCAAACTAACACAGTCAGTACAGATCGTAAAGTTTTCCGTCA
ATAGGGCCCTGACGGAGAAA

>Positive Sample 267
TGTTGACGTAAACTAAGACATTCCTATTTATAGTTGAATAGTTTATTAATATAGGTTAATCAGTCATAAA
CAAATTTGTACCCTTTTTTTTGAATCAAAGTACTTTTTTTACGAAGCAGTAAAACGTTTACGAGATTCTC
TCTACATATGCTCTTTCGCGTGTAGCGACATTTACCATTAGGTGAGAATTTTGTGTTACAAGCTGGATG
TCAGGATGATACCTTTGAAGGCATAGTTAAATTGGCTGGGAAAGGAACGGAACCTTTTCCTTTCCGAGTT
TCGGCCAGATATTTAGGCTT

>Positive Sample 268
GGGAAGAGAAGATTGCATAACTTCAACAATTGTTTTGAGCGTTTCCTTTTAGCATTTTTTTGTTTGGCTT
TTGTTTGTTCTTTTGTGTACTTTTCATGTATTTGATCTTCCATGTGCAAAAATAAGTGAAAAAGAGCTGA
ACTAAATGTAGTATTAAAATCAAGAACAAAACATAAAAAGGAAAGGAGAAAATGGAACAATTGCTTAGTC
AAATAATGTGAAGTTATAAAACTGCCGGAAGCCTATAAAAAATATTTTCTTTACTGAGACCGTGATCAGG
AGAACGAATGTGTATTAATA

>Positive Sample 269
GATCCTTCGTTATAAGCTATGAGTTTCTTAGTTTTAATGTCATAAGTTTCGAAGCCACCAACAACTAAAG
CGGCTTTACCATTCTTGTCAAGTACTTGTAAAGGTTTAGCAAGTGTCTTTAGAGTTCCATTACTTGGCAT
TGTCGGCGTGCAGAGCTTAAAATATTGTTCTCCATGCAGTAACATTGCATAATTGAAGTTATCGACTAAA

TTGTCCATAGCTAGTGTGGCAGTAGCTTGCATAAATGGAATGACGGCGAACGTGGGCAAAACTTGGAAGT
CTGGATCATTCTCGTAGGTG

>Positive Sample 270
ACTATGAAAGAAAGTAAGGTATTTCTATTTTTTTACTTTTTGTTATTTTATTTATGTTTTTCTAGGAAAT
GTAAAAATAAATCTTTTAGTAGACAGCGAGATGAAAACTAATCTGTGTGAGCTTCAATCAATCAATATGA
TTTCCCCGCATTTTGTTTGATATTTTAATTGATTCCGATTAAATCCGCTGGTAGCAGTTTTGTTTCAAAC
TAATTCAGCCTCCTTACTCCCCATTTCCTTATTGAACTTTGGGGATTCGGAGTTCCTTTTTTTTTCCGCC
ACTCCGATAACTAAACTTTG

>Positive Sample 271
GAGATCCCACAAACTGAAAAAAAATAGCAACAAAGTTTTGGGGGAAGCATTAAAATCAAGTTTACCCCTCT
TTACCTACATAAGAGCCAACGTAATTTTCTCTCAGTATTATATGTATTATCTCCGAAGTTTATTATTCTT
TAACGAAATATAAAAGAAAAATAAGAAAGATTAAGACCCTAAAAGGTGTACCACTACGATTCAACAAGCA
CAACGAATCCTATCCGTAATATACTTTTGATTTCTGACTATTCTAGAAAAAATTTAGTTCATTTTTTTTC
CCTCTTTTTACTTATTGTAT

>Positive Sample 272
AACATGCAAATGACACTGTAAAATGATTCATTACCCTGATTATGGAGTGATTTTCTTTCCTTTTTTTTTT
TACATTTAGTTTCATTATTATGCAAATTAGAGGGTATACAGTTGAGATTTTAACACTTTGAATTAAAAAG
TGTTACAGAGGAAACCGACGCAAAAGGCTTGGTGACGCAAACTTTTCCATCTTTATTTCACCTCTTCAGA
CGGTCCTAAGACCTTTTGAACGTATCAATATAGTTTTATCATCTGTTCTCTGTTGTTCTCCGTTACTAAG
ATATTAGTCAGCTCTTGAAA

>Positive Sample 273
TATTGAAGATGTCCTCATCGGACTGAACTAATGATTCGGGTTTCGCATATATACATGGCTTTGGCTACTT
TTAGGTTTATAGTTCTTGCTGTTTATATCACTAATATTTAGTATATAGCTGCGAAGAAAGAAAAAGAAAA
TATTCACCTCTAGGAGCCAAAAACATAAAAAAAAAAAATAATTAAAAAAAACTTTTATTGAATGCTTAATT
ATTTAGTGACGCGATTACCTATATACCAGGTTATTTATTTTTTCTTTTTTCAAAGGAAAACAATACAAAA
TATTACTGTTACCATTTTTG

>Positive Sample 274
TTGATGATCCCAATTACTGTTTTATTGACTAACTTTCACTGAAATGTTTTCATTCTCTGATTTTATATTT
AGTTGGCGAACTAGCTCATCCTAATTATAATTAAACTTATATATAGCATAAACCGACAATAATGCGTGGG
TGGTCCTCCATGCCGAATTTGTGAACTGAATTATTATCTAAGCACTGGATCGATCACCTTAAGATGATAT
ATTATTTATAACTAAAACGCAGCAGCATTACACTCCAGAGTAACTCCTGAATTTCGTCATGGCATGTACT
TATACTATATAACCAACAAA

>Positive Sample 275
CTAGGTGCGACGACGACTGATTCTATTGGGTTATCCGACACATCATCAGAAGATTCGCATTATGGTAATG
CTAAGAAGGTAACATGAGAAAACAGACAAGAAAAAGAAACAAATAATATAGACTGATAGAAAAAAATACT
GCTTACTACCGCCGGTATAATATATATATATATATATATTTACATAGATGATTGCATAGTGTTTTAAAAA
GCTTTCCTAGGTTAAGCTATGAATCTTCATAACCTAACCAACTAAATATGAAAATACTGACCCATCGTCT
TAAGTAAGTTGACATGAACT

>Positive Sample 276
TGTGATAACTGTACTGTAAATTTTGAAGTTATCCATGCCTCAACCGTGTGTTTACTGCTGTTTTTCTTTT
TAAACAATTGTGTAGACTTAACCTCTGCGGGCTTGAATAGTGCTTCAAAAGGTTCAGGTAGTCAAGAGGT
TAGCTATATAAACAAAGATGACTTTTCATCCTTTATTTCTTTTATTTTGTTTTTGTTTTTTGCCTTTGTG
GAAAAAAAACAATCTACAAGCGAAAAGAAAAATTTTGGAGAAAAATATAATTTAGGATTACAAGAAAGTG
ATAGAAAAATGTACAATCTT

>Positive Sample 277
ACTAATGGAATTTGTTTAATTGAACTTGACACCCGAGCAAAAGAGCTCACATTTTAACGTTTAGTTCATC
TTGCTGTGAATAATCTAAAAGTAAACAATGGACAAAAAAGTCCACTGGACGTCAAATCTTTTTTTATTGT
ATTTGGGACATATGGCGTTGATAACTAAAGTTTAAAGGAAGTAATAAGTGAAGGTTAAATCTGGACCTCT
TTACTTTCATATTAGAAGTATAAAACACACGCGGATTGGTCCATTATTAGAATACGGGTACTACAATAAT
GGTTTTGATAGTTACTGTAC

>Positive Sample 278
TACCGCCTCTGTGGCAATTGACAACACTTATCCCTATCAAAGACAATACAAAGTATTTGTTACCGAGTGA
GATTATTTAGTACAATATGTGGGTAATAAAAACTAATCGGAGATATTTTTTTACGTTTTTGATGCAAAC
GCAATAAATTCTTGATTTTTGAAATTTTGCAGGACATCAAGATACGTTTCTTTTAAGCAGTTTGGATTGC
TTCTTTCGTTCCGAAAGTAAAACGTAATGAAAACCCCTATAGTATTGTGACGCGTTTTCCAACTCTGCCC
TTAGCTTGATTTGACGGCTT

>Positive Sample 279
ATAAACTTTTTAATAATATTTATTACACGTGATTTAATATATCCTGTTTTTTTTTCATCATTCTCTTTCT
TTCTTATGTTAACCTCGTACTACAAGTTTTCTCCTTATAAAAAGCTGACTAAAATTAGAGATTGATAATC
ATAAATAAATTTAGTAGCCATTTCCATTTTTACATTTTGATTTAATCGACACTCAAAGTTCTTATTTGAA
AGCTAGTTTAGAACTTATTTGTTTGCTCCTTGATGAATATAATGAGAAGAAATTCCACTCAGATTAAATA
TGAAGATTGTTAACGGCAAG

>Positive Sample 280
CATTGTTATATATGGAGTTTGCTGCGCGCCTAGGCGTTCTCGAAGTTTCTCCTTTGATTTTACTTTTTGG
AAGGGCTTTGGACAATTTAAAGAAGATAATTTGAAGTACCCCATCAAATGCTAGAATGAAATATTTAATA
TACTAAATTAAGGCTGGTATTTAAAAACGTAATCTTATGTGTAGAACCTCAGTGAAAAAGCGCTATTATT
AATATAACAGTATTTTCACGGAGCTTTTTTTATTCAAAAAGGTTTTCAAGACTTGGCAGAGGACGGCTGC
ATTCTCGAATGATAATATAG

>Positive Sample 281
ATTAATATATCAGTACGTGTCTCAACTGGAATCGTATAAGGTTCTTGTAAGTACAAATTGTATGCAGAAA
TTTAAATGCGAGAACAAAACTTGAATCTCCCCCCGCATTTAAAAAATATCTTTTTGAACACAGTGCAACT
GATTAAAAGTACCTGTAATACATATATATGTTTGTATACGTATGTATATCTAATTATTTCTTACCGTCAA
ATCATAATTATTTTTATCCGTTCGTAAAGCAATCCAGTGCTAAATACCAATTGTGAGGCCATCGGCTTAG
TTTGGAACAGCAGTGTAGAT

>Positive Sample 282
GTTTCTGTACCGCTGTTATTTTTAACATCCACTGCGTTTTATGTATCTCTAAAATTGTGCGTTGCCTTTC
TTTTGTTTATATTTTTGTTCAAAAAATGAGTTAATAATGATTTTTCATCTTCGGCTTACCGGTCTTGAAA
ATATTTGTTAAGACAACAGTGGATAAAATAAAACTAAACAACTCAAGCAAAAGTATTCCACTTCTTAAAC
AAAGCCCAACTGAAAGGTTGCGAAATACTATGTCATTTTGATTTAATCATCTAATGCTCGAGAAAATTTC
AGAGCCGTAATACTTAAGTA

>Positive Sample 283
TATCTGTATTTTTAATGCAATCATAGTATAAAAATTTTTAACCAAATTCAAAAATTATAATATTTTAAAA
GTTTATGCTTTTGTCCTTTTATATTCAAGTCATTTGGCCATTATTACGCTTTTTTTTTTATTAGGTAAGT
GGTTTCAATAATTAAATTCTTTAGCGGGAAGGCTGACTATCTATAGTATTGAAGATAAGTTAAATTAATG
TTTAGTTCTGATATTTCATTTTTATTATTAATTAAATTTGCACTATTCGCCATGACGGGTTCACCCGAGT
ATTTATTTTGCTTTCGTTAT

>Positive Sample 284
ACCAAGACATCAAGAAGGTCAAACTTCTGTAAGCTCTTGTGTCTTTGTTATATGGGTACAAGATATAAGA
AACATAACTATTACATACGAAAATGTGCATGTTATCTATATCCTTCTTTATATAGATGCTGTTAACTTCT
TTTTTTTTTTTTGGGAAAATCAACTGTTAAACGCGACAGTAAAAGCAGCAAAACATTAATTTTGCTTCCA
AGACGACAGTAATATGTCTCCTACAATACCAGTTTCGCTGCAGAAGGCACATCTATTACATTTACTGAGC
ATAACGGGCTGTACTAATCC

>Positive Sample 285
ATCTGCAGTCCTCTCCTCTAGTACTTCGCCTGTTATGGGCATCGAATCATCATTTATTACCCTTGTCGAC
CTTTTCCGTAGCGGTGTGGTAAAATTGTTAAAGTTGGCTTTGTTGAACCTTGGTGTTTTCCAGGGCGGCA
ATGACGGTAGTTGAAACAACTTGCCTTCGTTCTTATCACCATTTTGGTTCATCTTACTTTGTCACAGCTA
TTTCTTTCCTTCCTGAATTTGTCCTTCCAAACCTAGTTCCCTGTTCTTAATTTCCTTTTATTTAGGTTTT
TTTTTTTGTTTTGTCAAGAG

>Positive Sample 286
TCAAGAAAGACCTTTTTAAGACTCCAAGAACCGTCACTTATGGCGTATTGTTTGTTTATCAGCACTTCTA
TCTTCGATAAAGGTTTGTCTGTCTTATATTGTTTACATTTCAAGTCTAATTCTGTGCTTTTACCGAAGAG
GAATTTTCATAAATACGGAGAAAATATAAAAAAAAAGTAAATACAGAAAATAGAACAGTTGAAGCAGAAA

AAGAGAACTTGCTAAATAGCTGTCTCACCCAGACAAGCGTATACTTACCACATTTAGTCTTTTGCAATCT
CATTCTTGTTGAAGATAACT

>Positive Sample 287
TTTCGTATTCGGTTTTGTACCTTTAGCTATGATCTTAGCTAATTGAAGAGGGTGGTGTGATCTTTAACCA
TACCTTATTATCTTTCAGCTGCTTACCATTTTCTTATATTGATTTTTAGCGAAAGATTTTTATTCACAAG
CTTTTTTTATCCTTAATGCTCGAATACTACAACAAAACAAAAAACATTAAACAGTTTTTAATTTTGTGAA
CAAACTGAATTACAAGGCCTTACATCTTATTTAGAATATATTAAGAAACAGAGGCCAACATGCCTTCTTA
ATTATATTGATATGGACCTC

>Positive Sample 288
TTAGCGTGACGATCTTAAAGGCGTTTTTATGTTTATTCGCTAAAACCCTTCTTATTGTCATTTTCGTTAA
CATACTATCTAACGTATTTCTGTTTGTCAGTAATTTTCAGTTAATGGATGGTATCAGACGAAAATTAAAT
AAATCACCTTCTGCTTTATAAGCAAGATATAAAAAAAAATATGGATCGATTGATTCATTTCCATAATGAT
CAATATATAATAATAATAGGACTGCTAGCTAATATATACTCAATATGATTTGTTCATATTCTTCGCAGGT
ATATATAACCATCAAGTAAT

>Positive Sample 289
TACCACCATTCAAACTTTACTACTATCCTCCCTTCAGTTTCCCTTTTTCTGCCTTTTTCGGTGACGGAAA
TACGCTTCAGAGACCCTAAAGGGAAATCCATGCCATAACAGGAAAGTAACATCCCAATGCGGACTATACC
ACCCCACCACACTCCTACCAATAACGGTAACTATTCTATGTTTTCTTACTCCTATGTCTATTCATCTTTC
ATCTGACTACCTAATACTATGCAAAAATGTAAAATCATCACACAAAACATAAACAATCAAAATCAGCCAT
TTCCGCACCTTTTCCTCTGT

>Positive Sample 290
GAACTGAATCGCTTGAAACTGGACGACTTCCAGATCGGTGTTTATATGGAATTGGTGAAGGTTTTGTTCG
GCTAACCCAAACTTATATAATCATTATAAACATTTTATAAACTTATACACTTTTTTAATAATCTCACCAT
GTTTTTCTTATGTGTTTTGTTTGACAATGACATTACTCCCTACAAACATGTGCGCGTTTGTGACAAAAAC
ATAAATAAAACAAAAATAATAAAAAGTCAAGTGAAAAGCACTGAAATCTAAAAAATAAATACATATACAT
ATATATATATATATACCTAT

>Positive Sample 291
TATGATTTGGCGAGTAGTGTTAGTACGTTAAATGCTACGACTCGGCATATACTGTGCTCGTTTTTATCAG
TTTCATCCGAGGATTGTAGCCTTTTTTTTTCATTTGTTGATGTTTCTCTAGTATTTTCCGTCTTGTAGTG
GTTTCGGGTATGTTGAACATAAACATTAAAAAAGACGATTAAATAAACTTGAGTTTGTACAAAGGAAAGC
TGTAGTCATAGAGTATTTAGAAAAAAAAGCAGCCACAGTTTACATGAAGATGTGACCAATGAGGAGGCAT
CGGTTTGCCTTTGAATCTCA

>Positive Sample 292
AAGAACTATTTGCCGTTGTCAAAATATATCGCATTGAGTTCAATTCGCGAGTACGAAAGAGACAATTCTA
CGGTGAATTCTGCCAATGAGGAAGAGTCGTATGCATACCCTTACGCTGTGAGTGAATTAGAGGGTCCGAT
GTTGGATTGAAATCCCAAGGTGCTATTTCTATATTTATATATTTTGTTGTGAAAATGCCAAAATAAGATT
TTTGATACAAGTAATACTGGATATTTTTTTATTTGCTTATAACAATAAATAAGTTCCTGACTTCTGTAGT
AGTAGTATAAACTCCTGATG

>Positive Sample 293
TCTGTATAGCTGTTTCCAAATCCAAATTCGGCACCATAGGAAAGTCGCAATTGTTGCACGTATATGTCCC
TTGGATATTCCAATTAAATTTGCGAGGATTGTTCTTTCGGCCGTTCGTGGTTTGTTGTAGCCCTTATTAT
CCCTCTTTTTCATCCTTAATTGCAAATTTATGTATGAAAACTTTCATTTCTAATGTTATTAATTTTCCCG
ATTCTGCAAACTAATTGATAAAATAATTGATACTTGCCGTCACAGATGATATAGAGAACGTTCAGTTTTT
TTATTGTTCTATTGTAAAAA

>Positive Sample 294
TGAAGTTTCCAATTATTTATATGTATACATTATTTATTTGTCTATCATTAAAATTTCTGTCAAGTAATGG
CTTTTTATGTTTATTTCATGGATTACGAAATTTGCTGGCTTTTTAGAGTGACCGATGAGTTGCATGTACA
TATGTGCGAAATAAAACAATACGGTAGTAAAACATGAATACTTCGAGAAGTAAATTCAAGATTGCAGGCG
TAGCACAAGAATGTCCTTCCGCCTGCTAGTGTGTTGAGCAAAGCACGCCATCACTAATGGAACCCTTACA
AAATCTCGCATGGAGCAGCA

>Positive Sample 295
ATTGTTACTGCTGCTTCCTTTACCTTTCTTTTTTTTGGTCAGTGACAGGTTATTTTCTTTGTTTACTAAA
TGGTCGCAAGATTTTCGCGATGCGTTTAATTTTCATATTATTGCTTTCCAATTTACCCAAAGTGCAATTG
ACAAAGGCCATTAGTCTTTCTTTTTACAACAGTAAGAATATAAATATACAAGTGCAGTATTTAAGATACT
TAATTCGAAAAGTGAAAAAAAAAAAAAAAGAATCTGGAATTGGTTATTTTAATTTCTCATATGAATTGCTT
TCCAAATTCTTGCAATCAAT

>Positive Sample 296
AACCAATGTGGCACAATTTCTAACTAAGTCGCCCTCAGTCCGCTCATTTTAGCTGAATTTTCTAATGTTA
TTTTTCATCAGCAAAACTTAACAGAACGTTAATTTATCTACCCCTTTTAGTTCATTATCTCTTTTTTATC
CAACATTTTACAGAGATCTCTCACTTAAGTCTAAGTAAAGACATTATTTTATATGGTACACTTATAGAAT
ATACGATAATAATAATAAAAACTATGTAACATAACCTTCAGAATTTAATATTAGTTTCCTTTTTACCTCA
TTGCACTAATAAAAAAATTC

>Positive Sample 297
GGTAACATCTTGCTACCGCCATCTTCTGTTAGTTTACGCCTTATGACCAACATTCCTTATATTCTCGTTC
AAACTTTTGTCTCGATATTTTGCAGGTACCTGTAGAACTTAATGTGTTCTATTCAAAATAAGTATAAAAT
TTCTGGCCAGTAAAGGCAAACAAAACATAAAGATATTAATACGAAAAATTTCTTGAAGAAAACAAAAAAT
ACTTCTTATAATAAAGAAACAGCGAAGCCATGGCTATTGACAAGATAATCAGGAAGGTGAGATACCCTGG
ATAGAAGACTAAGAGAGAGC

>Positive Sample 298
GAGGTATAGTTCGACAGAAGAGTTCTATTAAAGTTTTCCTGTTATTTCATTTGGAATATAACCCAAGTTG
CTTTTCTTTGATCATAATTATTTCCACCTCTTTAATGGTTTGTTTATTGGTTGATTATTTCAGCCTTTCT
TTTAGGTTCCTTCGCATTACATTAAACCCCGTAACTAAACTTAAACTTTAAAAAAAAAATATGGACAACT
TCAGGAGATAGATACAATAAAGCATCTTTAAAATTTGAGCAATGCTTTGCTTATTAACCAGCAAATGAAG
ACTATGAATTCTAATTGCTA

>Positive Sample 299
TATATGGCTATTTAATGGCTTGCACCTGCGTAGAGTTACCAAATCTAAATTTCACAAGAGACAAATTAGT
TGAAATTTCATTCAGCCCTTTCTGTAATACTGTAGAGTTACTGAACATTCCTTTAACTAGCTAATATACT
GGAGTTGCATCGTTGATGTGTATTATCTATATTCAAAATAGTTATTTTTATGTTTAAGAAAGGATTAAGA
ACAATTGAAAAAAAATTACCATTAGCAACGAATATTAAATTGTAAATTATTTAGAGATATTTTTCTATAA
TATGCTCGCCTACCATTCTC

>Positive Sample 300
TGGTGTTAGTATCCACAGAATGAGAGACCCTGAGAGACAGAATAATTCCAATTTTTATAATTAATGTTTC
TTCATGTAATTATTCAAAATATTGTGTAATCGTCTATTTGAATTCGTTTTCACGGCAGCATATTTGAAAA
AAAAATTAATTTTTACATCTAAACATAAAAAACCTAATCGTTTTCTTTCCATGCTGTTATAGAGACATTT
ATTAGTACGAATATTTTCCTTTCTTTACGAGCACTATAGACAGTAATTTATATAACTAAGAAAATGGTAT
GCTCAAAAATCAACAATATG

>Positive Sample 301
CTTTAATTCTTCTTATTCATGCTCTAAATCTTAAATAAAAGGTTCCGTTTTTTATCTGCAATTCTGTGTA
TTTGTAAGTGAATTACCATGCACGTTGTCGGATCGTTTTTCTAGGCAGAAACTATCTTATTGAACCATTA
AAAGATTACTTAAATATTACAATTAATCTTACGAAGCATATTAATTAGACTGGAATTACAGCGCAACTAT
CACAGGTTAATAATTGGTTGAGAAAATATTATAGTTTTGTTAGTAATGTTGAACTTTTATTTTATACACT
TATGAAAGTAAATTAATTCA

>Positive Sample 302
TAATGGATCAGCCCAGGGTACAGTGAGTTATGATAAAGAGAGTGATGATCGCAAATGCTGCTTGTAGAAA
GACAAAAATGAATAGAGGAAAGCCGAGAGGTTTGTTTCAAAAATTGACGCCTTCCTCATTTTTTTCATAA
TTAGCTGGTAAAAGTTACTATTTGTAACGACTGCTAAATAACAAATAATTATTCGTGTAAAAATAAATAG
CTAATACCACCAGGTGTTCATATTCCATGACTGCATATTTGGGAAACGAGACGGTTTGAACATCCTTAGT
TGATGTAAAACTATAATAAA

>Positive Sample 303
CATCGCTACGTGAAGATTTTTACTTTTCTTCAACATGTGTATTTTTTATGTTTCATATTTTTATATTTTG
TGTTTGCTAAGGTCTAATCGTTCAGCTAAGTTAAAGCTAAGTATAAGAAGCAAAAAATACTAAATTATAC
TAAATTATAAATTTTCGTACATAAATAATCATGAGGTAGATGTTTAAATATGTAAATAGTTTCTTTTTTC

TTCTTTGTGATTGTCAAAATTTGTGTCTATCTATACAACCCTCTAGAGGTATATCTTAACTGTGTATTAT
CATCAAATTCGTTATTATTA

>Positive Sample 304
TTATGTTTTCTCGTTTCTTTTCTTTTTTTTGTTTTATATCTTACACATTTTTAATATACTAAAAGTAAAA
TAAAAAAATACGTACTGTTTCCCTTTGTTTTCTCCTCCTCTTTGCTTATATCTCCACTGCGCCATCGTCA
TCTTCAGAGGGCGTGCTGATTAGAGTCACTGTGTCTCCTCTGATGAACACCATTTCACATCGTCTTTCGG
ACTCACTCAACTCCTCGTTGTTTAATTGGTATATGGTCTCTACTGCATCACTCAGCACGATGTTGCAGTG
TGAGTCGAACGCTTGCAGTG

>Positive Sample 305
ATTCTTTCACCCATCATTCAAAATGCATATGCATACTATATTTGTCTAGTTTTTAATTATATAATAATAA
TAACAGAAACAAAAAATCGCATTCATCGTTTAATCTTATTTTTCTTTTTTCACCTTTTTCTTGTGAAATT
GCCAAGCGCTTAAAAAATCGTTATTTCAGAACTAAACATCATTAAGCAACGAAGAAGAGAAAAGCGGAAC
TATAACCATGAGTGATACTACGGAAGTCCCTAGGCAATCATCTGAGAACGACCAGGATAATAACTTGGAA
AGAACCAACTCATTAAAAAG

>Positive Sample 306
GAAATGGGTGCATAAATTTATGTGCTTGGTACATATGAGTTCAATAGATATGTAGTAGTTGGATAGTAGA
TAGTTAGGATATATAATCATGGAAGGGTTACTCACAACGAATCACATAATGAGTTATGATTTTCACTGTG
CTCATCTGTTGCCCCTACAAGGGGTGATCAATAACAAACAATTTCATTAAAAAAAAAAAAAAAACTAAGT
CACTATAACTAAACGTAATAACTATGTATATGAAAGAAAATAATATCTGTTCGTCATCTAGTTTAATACG
GCAACGAGTTTTTCACCAGA

>Positive Sample 307
CGGATTCTCTGTATTCTGTCTATAGTATTTATATACCAATACATTTTTCCGCACTTTGAAGTTTTGCATT
TTACTTAATGTAGTTTATTATTAGTTGCCAAAACGAAACATTTAACATGTGAATTAAAAATACAAAAAAA
AGCCTATTAACGATCTAAATTTATGACAAAAGACATATAATTATTTTAATGATGCCTTTGTCACAACCGA
ACACAATATCTTTGAAAAGTGATCCAGCAATCGAATTCAGAATTCGTTGTGCCATTTTTGCCTTACGTTT
GCGCCATTGTAATTTCTCTG

>Positive Sample 308
ACATGCAACTAGTAATAGTACAATTTTTAGTACCCTTTAATCTTTATTGTTCGCTCCCTAATAACGAAGC
CTTGAACTGTAACCAGATCCCAGGTTACCCCTTATCCTTAAGAGGTCTTTTATTTACTCTACATAATATA
TATTTTTCAATCCATTGAAGAGGATTAAAAGAAGAAAAATCAATACCGAACAAAAAAACAAAACGAAATA
AAACAAAACAAAAAATTAAATTAAATTAAATTAATATTAAATGTCATCATGATTCTTACTCTGATTGTCT
TCTCTTCTCTCTTTCCCTTG

>Positive Sample 309
AACCACTTGGGTTCTATGAAAGTTGATATTCCGGTTGATGTGAAAAAGTTACAAATTTAGACCATACAAC
ACTAACAGTAATCTCGCAGTTTTACAAATAATTGATAAAAGCAGGACTATTTTTAACCATTTAATATAC
CTATTACTATTAATATCATTAATATTATTATATTATGTACAGTCACTTATTTCTTTTTTTTTTTTTCTTT
CTTTCTTTCGTTCTATTTCCCCCTAGTATATACGAATTCTGCACATCACAGCAAAATATATGGCTATCAC
ATGGCACAGGTATTACTAAA

>Positive Sample 310
GGTTAAACGAACTTTTCACGATCATTTCAAGAATCATTGAAGATTAAATTTTACCATTTTAAAATTTTAA
TTTTCTTGGGTATGAACTTTTATTTTCAACTGCTTATTATATATCAATTCTATAAATTTTTTTCTTCTCT
CTAACGACCAATTATAAAATTCATCCTCTTATTTATTACAGCATCTTATACATTATGTATATGGGTAGCT
ATTATTCATTTTTGCTTCGTAAGGTCTTTTTTTGTCAACTTTTTCATCCTAAGCGGCTAAAAGTGATTGG
AGAGGAATGTCCAGGCGACC

>Positive Sample 311
CGTTGTCATTGAGTGAAATCAATTGAAAGTATGTACATTTGGAAGAGCATATGTTAAATATTTTATTTAT
AGATTTTTCGTTGTGAAATATTATTTTTTTTTTTTTGAAAAAAATGTCGGACTTTATTCCTCCTAATTAT
TAATAAAATACGAATATATATCTAAATATAATTAATGCTTATTTACATGAAAAATCATCAATCGTAAACA
GTTGATTAAAAAACAAAAACTTTATCGGTTCCATTACCTTTGTGAAACCCATTACTCCACCCCAACTCCT
TTGCATAATTCAAAACCATT

>Positive Sample 312
TGAAATTGGTTTTGTTTTGCAAAATATTTATGTTTTGTTAATCTCCTCTACCCTTTCAATGCTTGAAAAA
TACTTTCAACTTTTCGATTGGGTGATGAAAAAAAGACAAATAGTGTAAAGGGTTCAAAAATAAATAACAA
GCAAGAGAAAGGGACTTTGCTTTTCTCATTTAGTCACCAGTAAGTTATGTCATGGTGTAGAATAACGAAT
TACAGAAAACTAATATAACTGATGAAAGACCAGGGAGTAAAATGGCTTTGACTCAGTTTGAAAATGATTT
GGAAATATTAAGAGATATGT

>Positive Sample 313
TGTCAACAAGAGTGTCAAGACCGGCTACTTATTCCCCAAGGATCACGTTCCTTCTGCCAACATCATTGCC
GTCGAACGTCGCGGCGGTCTTTCTGACATTGGTAAGAATACTTCCAACTAAGAGCATGCTTCTCTTTTTT
TTTGTAGGCCAATGATAGGAAAGAACAATAGATTATAAATACGTCAGAATATAGTAGATATGTTTTTATG
TTTAGACCTCGTACATAGGAATAATTGACGTTTTTTTTTGGCCAACATTTGAAATTTTTTTTTGTTACCT
CGCGCTGAGCCCAAACGGGC

>Positive Sample 314
AAAGACTATTAATGATTGTTTCTATCGTCAGGATATTTTGAAAACGTCCAAATATGTAAAACAACTTCTA
AACATTAAATATTTTTGGTGAATTAAAAAAAAAATATGCGCAAGCCCGGAATCGAACCGGGGGCCCAACGA
TGGCAACGTTGGATTTTACCACTAAACCACTTGCGCGATCTTTATTGGTATTTGTTGGAACATAGAGTTA
ACTAACCATATATAACAACTTCTTAAATGCACAGTACAGTCTATTCAAAACTGTAGCTATGCGCTGCATC
TTGTGGCAAACTCGAATGGA

>Positive Sample 315
AAGCTGAGTGGTACTAATGCACCGAGATGCAAAGTCCGAACCACCCCCACCCCCCTCTTTACCTATTTTT
TATATTTTGTGATAAAGGGATAACTCGTCTTGCCTTCACTGCGCTTCGATTCTACTTTAGCTAAACTAAG
CGTTACTTTGTTTGAGAATCTCTCAGCCGTTAAATCAACGTACTTGTATACTAAACAGTATATTCACGTG
ATAACTTTGCCTTACACCTATGATATAGATAAATATATACATATATATGAGTGTGTGTGCATTGCGTCAC
TAAACTATGTTTAATATGAC

>Positive Sample 316
GGTATTCGTATCGCTTGTATACAATGATAATCGAGGTAAAACTCATGTACAGTAGTGCGGCATATTTTTC
TTATTTAGTTTTTGTAGGCGGCATAGTATTACTTGCAGATTTTTTTAAAGTCCCTGAGCCCCAAAGTTCC
AAAGCAACAAAAAAACATAAATCCTTTGCAGGAAAAGTAACATTTGGCAAAATTTCAACAAATTATGCCC
CCCGAATTTAGCAGTGATCAATGTTAATTTGCCTACATGCTGATGCAGAGTTGTAGAAACAAAGAATTTT
ACCCTGTATGAAAAATTTTC

>Positive Sample 317
ATATTGGTAGCAATCCTTGGTGACGCTGACTTTTTTTTTTTGTAATTTTATTGTTTAGTTCATGATAAAA
AACTTCAAATCACTTTTAATCTGGTAGACAGAGAAAACAAATCGAAACGAAAATAGAGAACTACGAATAA
AAAAATATAAGTGGAGAAGATCGTCACTACGCATTAAACAATATTGATCGCTCAATGCCAGTACTGCGCG
TAAAAGTTTAGTAACTTAACGATTTAGGCACAATTTGAGAAAAATTTCGCCCTGCAGTAAGTATGTTATT
CAGTACGATATAAAGCTGAG

>Positive Sample 318
CGGAGCGACAATTTTGTTATCATAATGTCATATTATAATGTGATACATCGTCTGAAGAAGAGAATGCTTT
CGAAAAATTCACCTTAACGCTATGATCGAGGTTTGCTAACTCAGGGTAAATAAATATTACATTTGTGATA
TTTCGGCGGTTGTGCCTCTTTTACTACTTTACAATTAGTTACAATTTCTTTTTCTTGAGTTACTTTTCCT
CAAAAAGAAAAAAAAAAGAAGAAAGGCAAACCTAAACTAACTTATGATAATATAGAAATATTCAAAGCTA
AATAAAAGTAATTCTGGTCT

>Positive Sample 319
CGATGACCTAGGTTTAACAAGATCATGAAATATTCATTAACGGAGTTTTTTGTTTTCGCTTTTTTTATTT
TTTGTTCCTAATTTTTTTTTTTTTTTACATTTATTAGCTATACACTTGGCGCAAGGATATTTTATATACC
GGATATAATAGATAATTAAAAGACTACTAATTAAAAATGATGTAATATGGTAATAGGTATGGAAGAATCA
GGGAGTTTTCAGAATGTTCTTTGCCCTCTCTTGGGCTTTCTAGTTTTGCACTTTTACTTATTCATCTCCT
GTAACCGAAACTGAAATGGG

>Positive Sample 320
AACAATGGAGTTCACAATTATCAAAATATTCGCACAATATTCACGTAGAATAGTGGATGACAGAGTATAT
AAACAAAGAAAGAAGCACTCAGGATTTTCTATAAATAGTGAAGTTTTCTATGGACGCCAAAAAATCTTAA
GGGTATCTTGAGTGTAATCATCATTGGAAGGATTATTATCTTTCACGAGTAAAATACTATTAATAACTAG

TAATTTCCGTGAAGTCATTAAAATGAACAAAACGATAAAACGGTTTCTTATTGCTAATGTGCTGATTTTT
TGCCAAAAGGTAAAATCTAA

>Positive Sample 321
TGAGCCTTATAATTCTATTCGCTTACTTTCTAAATTTTAAGTATAAGGCAGTCCTCTGGTCTACAGAGTT
GTTTTGTTTTTTTATCCAAGTTCTTTGTTTCAATCTTATTGTAGTTTTTTTCCATTAAAATATTAAAGCC
TAGGATAATCTATATCCTAAACTAAATATAAATTAAGAGTATAAAAGATGAAAGTAAATAGCATCTAGCA
TTCAGAAAAAATAAGAAGTCTCTTTTCAAATGATTGATCGAGCACAACGAATGTTTTCAAACCACTGTTA
ATTTTTTTATAGATGTAATG

>Positive Sample 322
AAAGATACTAGATAATGTTTGTTCTTGATGGCAAGAAATACACAATGTCAACACCCGCGCTTTCTCTTCG
ATTCATTAGTTGATATCATTAACATTATATTATACATACTTTATTTCTCTTGGAAATTAAATATTTGATG
ATGGTTTATCTAAATATTGTCTCACTCATTTTCCCTTTTTTTTAAGATAGAATATCAGTTTGAGGCAACA
AATGATAAATTATGATTATGTATATAAAACAATTTTAATTTTATTAGCATAAAAAAGGGACAAGGCCTCT
GCTAAAATATCGATAAAAAA

>Positive Sample 323
GCAATTAACCAAGTCCTTTTATTTTTTTAATGGTTTATTTATGAATTGTTTATGTTTATGTTCTTTATAC
TCAAAATCAATAAGTGCTTATTTTAGCGAATTTTCTTCTACGAATATATATAAGTCATATCTAAGACTTC
CATCCGTGCTTTATTACGTTTGTATGATGCATACAATGATGCCAAATCAGCTTGATGCTGTTGGTATTCA
GTCGTCCGAAAGCCTGTTGATGTGACACTCAAATCTTCGGCATGTGTGAACGGTGGCCGAGCATAGTTCG
TGAACATCATCACTTAAAAT

>Positive Sample 324
TTTCGCCTTCATGTTTTATATATTATATAAATTTGTTTACTTATTTTTACTATTTGTAATAATGATTCTG
CTTTACGCGCCTTTAAAAAAGTTGGTCTTAATGTAAACATGAACCATAGCACACAATTTAAGACATCAGC
TGTCAAATGACCATAAAATTGTCCTACCAGCTGTCCTTAAAGTTTAGTGTACACTTACGAATGACTTATT
TACTCACCTTTGCAACTTTGTTCAATAAATTCATTGATCTTTTCAAGTTCCTGACATCTTTTGTAACTGG
CATAGAGCTTCAGACTTGAC

>Positive Sample 325
CCCTTGAATGATACATGACTAAATATAATGATAAATTTATTTCTTGATTTTTGATTATTTCATATTTTGT
TGACATTTGAGAGCTTCAATTGTATAAAGTTGCAAGATAATATATGAGGTTAGGGAAGCCGGATAAAGAA
AATAGAGTACTTTAAAACTGTTCATGGACTAAGAATTCAGTGACGATGAAGTAGTAAAGGAGAGTGTTTA
GGATATAAATAAGACCGAGAAATTGTTAGTAAGCATTACTTCTAGCAGAAATAATGAACCACAATCGTGA
CAATATATACTCGAGTTAGC

>Positive Sample 326
GACGAAATTACTGTAAAGGGCTTAAAACCTGACTTGGAAAATGTTCAACTCCCAGATCCTAATTGCGAAC
CCATAGCCACAACCTTTCGCGCGAATTAGTTTGTAAATAAGTCAACTTTTTAGATGTGTAACCTGATAAT
TTTTTTTTTTGATGTTCGACATCATTATTTCTGAATGAAAACGTGAAAAGCTTCGCCAAACTTAAAATGG
AAAATTTCGAATGGAACAAAATATAATCGATGCGATGAATTAAAACCTTGGAAGAAGAGGGTTAATTGTT
AAAATTACATATATAATTGG

>Positive Sample 327
CTAAAGGCAAGAGAGAACTGAATCCGCTAGACGTTATTACCACAACGGTCACCGAGACCAAGTACAAGAC
AGTCACCGTCACTGCCAAAACTTAGCGCGGTTTTTTCTACCTTCCCTTTTTTCATCTTTTTGTTATATAT
ATATACAGAGACACTCATGCGAATTTTCGCAAAACGAAGAAAAAAACATAAAATTTTCGTTTGTATCTAC
ATGACTATCCTTTAATTATTACCTTTCTGTCAAATGTTTTATGGGAATATATATGTTAATATGAGCCAAT
AACTCACTAAAAACTCACTG

>Positive Sample 328
GTTTAGTTAGGTTATGTGAAGGCACGGGTTTTGTCTTTTTTTTTTTTTTTTTTTACTATTACTTTCTTTT
TCAAGCTTTTAAGCGCCGAAATGATATTTAAGGGAAGATGACTAAAGGGACAGCGACGAGGATTCAGCCT
GGACAGTGATAGAAAAGTTATGCGGGAATACGTATATATAGTTGTATAAATTGTGGTTATAGAACATCGC
AGCGCCTTTAAATATATTGTCTTTTATTTCAATCTTATTCCATCTCTCTCTTGCAACCACGGCAAAGCTG
GAGCTATGTCTGAAAAATCA

>Positive Sample 329
TGTATCCGTAAAAGACATTCTTAAAAGTTTGAATTTATGATCTTTTTATTTTTGTATTTAGTCTTTGTTT
TCGAACTTTTATTAATATTAAATTTCAGTAAGTAAATTAAAAGAACATCTTTAAAAGAGTATATTTATTG
TTACATATGTTGATCTTAAAAGGAGATGAATATTTCTTAATGGAAAAAGGTAAAAAAAAGATTTTCTCAT
TCATATGTTCCCATTAGGCAGTTCTATGAGGCAGTCGTCCAACTCGTAATCAGAGATCTCATTCTCATCC
TCTAAGTAGCCCTTTACCTG

>Positive Sample 330
GTCAGTAGTCAAAGCATCAATTTATTTCGTTTTATTTTTCTTGACAAAGAAAATTCTATGTTTTGTTCCT
GAAATCTCGATATTTTAGTAACTTAAAATAAAGGTAATAAAATCATCAAAGGTTTTCCATCCAGTGGTCC
AAAAAAAAAAAAAATACAGAAGCAACAAAAGAGCACCAGAAAATGCATTTTAAAAGAATTTTATTAATAAT
AGGGAATACAAAGGACGCGATAAATACCTTCATCTATAAGCAAACCATACATATATCTATATCTATAAGA
AAAGTAACGAGAATTTTTTC

>Positive Sample 331
TGTGCTGTTGATTGTGGATTCATTCTGAGACAATAATTTGTCCCAGGCATTACAATTATCCATTAATACA
TACAATTCTTTTATCTTCTTTTCTTTTTGTACAAGTTGCGGATTACTTCTCTTTGTTTACCTTTTTGCTT
TTATTTTTTTTTTTAATACGTGCATATATATATATATATTTAGTTTAGGTCAGAATACAAAGATAATTAT
GTATAAATGGGCGCATTTGGAAACTATTTACATACAACTGAACAAGTTCGGAACAATCTATCAATAATC
TGCACAATTATATAATCCGA

>Positive Sample 332
TCATCCGGATAGATTGAGATCGCATTCTTCCTATGATAGACCAATGATCATTAAAAACTGATACGTCTTA
CGACTATGTTTAGCTTTGATGTTTTCTCCATTTTTTTTATGTTTTTTTATGATAATCTCTCAGCCGCCAT
CACTTCCACACAGGCAATGCTCTACAGTTTTATTTTGCTTTTAGGTAATGCCGCATATATATATATATAT
AAGTTAGAACACAAACTATAGAGCAGCGAGGACCCGTAGCTAAAAGGAAGACAAATTGAAAAATCTTTCA
CAAATGAGTATAGAATATAT

>Positive Sample 333
ATTAGCAAGGTGTCGAATCAAGTTATACTAGATAGGAGGATTTCATAATGAATAATGCTTTAAAATGGTT
CTCACCTTTATTTTATCATTTTCTTCTTTTCTTCTGCTATTGATTTGGTAGTGTTCGGGTTAATTCATTT
TTTTGTTCAATAACAAGAACCAAACATAAAAAAAAAATACTTTAAGAATAAACAAGACTGATGAAGCATAG
ATCATATTATTAGAGCGCGTAAGGTATGTAGAGCACAGATTGGTCCTTCGAGTGTGATTATATAATTTTA
TATACGCTCCTTAATTTAAA

>Positive Sample 334
CATTAGACAAAATTGGAAAAGTTAGATAATTAGCGTAAAAGCCTTCATTAATGATAATAGCGTGGGAGGT
ATAAGATGCGCTTAATAACGCTGAAGAATATAAAGTTTCTTATACATTCAATGAAACAAGGAAATTTTCA
ATTTTAAATTTAAGTTGAAATAGCCTTTAGCACCTAAAAATAAAAAAATATTAAAACACCAACATGTTGT
TGAACAAGTTTTCCAGAGAATTGACCGAATATTTAAATTTCTATTTAAGAGTCATATAAATTTATTGAAA
ATCAAGTTGTATATCTTTAG

>Positive Sample 335
ACGCCAATGTCATTATATGTACCAATATAGACGCATTATACAATCCTACAATTTATATCATTGTAAAATT
ATTTACTACCACTTATTCTTCAATGGTGTTTTTAATGTTTTGTAGAGGAAAACTTAAAAGTTGTCATTTT
TATTCAGATCGCAATCCATGGTTTGACACATTCTATCTTAAAAAAACAAAATCTATTTATTTTGGTTTCG
GCTATATTGATGATGATGAATGTACATATAAAATGGCAAAATTTAAATAAATAAATTATTTAAAATCAAT
TTTGTTTGAGTTAAACGATA

>Positive Sample 336
CTCATCCAATCAATCATGTATTTACATGCCTGGAGTGGCCTATTCTATGTATTAACAGCCTTTGCAAGAC
GCGACATTGTTCTTTCCGATATGGCTCGGAGTAGACTAAATATGCTACTTATAGTTTTTCTTGCGTATAC
CATTGTCTTATAGTTTTTGCACACATTAATGTTTAAAATTGAAACTACGAAGCAAAACAATAAAAAAAAC
AAACAAAACTTAAAAATAAACAAAAGACGCTCGAAACCAAGTGCATTTATTGAATGTTTTTGGGAATGCA
AGTAGCAATACTATTGTAAA

>Positive Sample 337
GTTCTTTGGAAGCGAGTGCAGCCGATGAAAGCGATGAAGATGAAGAGGCCATCCGCCTTTTTGGCAAAAA
GTCTAGAGTTGTTTTGAGCCAAGGTGATAGTGATGATTGAGAATGAATATGTAGTAATTAATCCCTTTAA
TACGTGTAATTTTAGAACCACTTCTTTACTCTGTTTTTAAGTTCAAAGTATTGACATAATATTAAAACAA

TACATGCGATTCATTCTTTATCATTACCTATATTTTTCTAAGTTGTTGAACATCTTAAGGAACTCAAATG
TCCATTAGGACACATAAAAT

>Positive Sample 338
TTCGATAGATAGCCAAATGCAGTTTATATATATGTATGTAGACTTGAAGGTATATTAACAGTGCCCATGT
TTCTTTGGAAAGTGTCAAATATCGCAAAGCATAACCTTGAAGGTTAACATTATTTTCTAGAAATACACCT
TTCCCACTGGATATGATGACGGAAGAAACAAAACGTTAAATTATTGTACCGTTGTATAAAACAGTAACCG
AGCATGGACAATTCATTTTTACACCCCAACGACGCCACTAACCATGTATAGGTGTCTTGCCTAACTTATC
ATTATTCATAGTAGAATTAA

>Positive Sample 339
ACGAGCAAATCAATGATAAGTACAAGTCCAATCGGACTGATTCGTAAAAATTTTTGCAGACATTTTTGAT
ATATATTCAATTCATATTTTATCTCTTTTTTTTTTTTTTTTTTTCAGACGTGGAATGGTGGGAAAGCCAAA
ATGTCACTGACCGCGGCTAAAAGTTAAAAAATACATGAGACGACATTCTTTTATGTATATATATTTACAC
ATGTATACAAACAAATACACACGCAAGTAGAGAGGAGTTGGTGGGGGGAAGATGCCATTATAGAGGAGAA
AAGAACAAAGAGAGTGAGCT

>Positive Sample 340
CCTGGACTACTCAGTAACTTGTAGTAAAACCATTCTTCTATACTTCTTGGAGGCATGCTTAAACTGATGT
GATAAACATATTCTTGTTGGTAACAGAGTGTTCGTTCAATCTTTTTAAGATATTTTGTTTCCTAATTTCT
TCACATTTTTGAATTACTGTTTTTCTTAATTTCATAACGGGTTCTTTTCAAAAAACCGTAAAAATTTGAG
GTCACACCAACTAAATACAAATTGTTTCATCACGGTGACTATATCAAGAACTTCGTAAGGAAACATTTAG
AAAACTCAATATAGTAAAGT

>Positive Sample 341
AAAGGTTACATTTCGCTAAGTAGATTGATTACCCCTTTGTTATAAATAAGTATATGTGTTTACTATCATA
TAATAATTAATTAGTTGTAAGTATGGTATTTTTCAAAGAAACATTTAGTATTATTTTTAAAGGGTGCCTT
TACCATAAAACCAAAAAAAAATGGCTTCTGAGAGAAAAACATAAATAATTGATAAAACTGAAACTCACAA
CACCAACTCTTCCGCTCTAAACACCCAAAGCTCAAAAAACGACATGTGCACATCTATATCCATAAACTTT
CAAAAAAGTGGTTCATTCGC

>Positive Sample 342
TGTTATTGTTCCGTTTCATGTTATTACAATATAATTGACTATCTGAACATTTTGTCTTCATTTTTTTTTT
TTTTTATTATCGTTTTGTTTACGTTTAGGCGAAATACTCACCTCTTTACTATTAAAATAATAGAATTTAT
CAGAAAAAAAGTCAGCAACATCAAAAATAACAGATGGATGGCTCTAGGATAAAGGATTCTTATTCTCTTA
CGATTGTCTATGTTCTTTTGGTATTTATTTTAACTTTATATATCTGTCTTAATTTATAAATATTTATAAC
AACCCTTGGCGTAATGTAAT

>Positive Sample 343
CGCCTGTGTTATCGCTTAATTTTTGACGACAACCAAGAGGTCAAATCAATATCTACCCTTTCATTTATTA
CGTGTTGCTGGCAAACTAATTTATTCCAATTCTCTCATCATTAGCTCACCCTTTCCATACTTTTTCATAC
TTTTATACATATGTATTTGTAGTTATCACTTTAACAGATTTTTTCATGTTTTTCTTTTCTGATACGCCTT
TCCCTCCGCAGGAAATGAAAGATGAACCACCCATTACATTCGATTTTTTTTTTAATTATATTTTTGACTA
TTATTTAATCATTAAAAACA

>Positive Sample 344
CATCAAGGTTGACCAATACGATTGCTGATTCGACAACTTCTACATTACATAGGCATATACATATCAGAAA
AACGTGCTTTATAGGTACATTTTTTAATTCCTTAAACATAACTTGCTTCTATTTTGGGATAGTTCCTTTT
CTTTGAACTACGTTTTTTGGGCAGAAATATAAAAAAAAAACCGAAAAAAACGGCACGCAAGAAAGCTTATT
CAAAAAGACGCAACAATATCATAGATTATTCTAAGTACCAACAACGTGATAAACTTTGTTCTAATAAAGC
TACATCTTATCTCTTCTGTA

>Positive Sample 345
GCAGAATTAGGGAGAAGTATTCAAACTAAATACACTGTTATTTATATACATCGGGAGGGAGTTTAAAAAT
TGTATACACTAAAGTGACCTTATGAATTGGAGCCATTTTCAACGTTTTAGAGTGATGTTGCATCCTTTTC
ATCAGATCGGGAAATTAAAAAAACGGTTATAGAAGGGCTAAATGTAAATATGTAAATTTTATGAAGTCAA
GAGTAAAGAAAATCTTCGGTTGCCGATCAAATACACTCTTACAAAATACCCTAAACTCAGTTTATCTTTC
CATTCCAACTGATTATCGCG

>Positive Sample 346
TTTTTACTTTCTGCTATTGGACCCTTTTGGACCAGCCGAAGGAAGTTTTTTTTCTTTATGTTTAGCTCTT
TTTTCATCACTTCTAATTCGCAAATAGAAGGGGAAGGAGGACAGCAATTATGTAAAAATTAATTAGTATG
TCAGTAACAAAAAGAAGAGATAACAGAGGTAGTTCCTCTAGACATTTTTAAGACGACTATTGATGCCAGG
CAAATTTTGGATTTACTGCTCCTCTTTTAAGAAGACAAGTGTGTGATATCGTAGCGGTAGGAACCAATTT
TGCAATCGATTTACTTACAG

>Positive Sample 347
CTTTCTCCGTCAACATTAGTATTTAATTGTATACAGACACCATTCATAATTGCAGTTAGCTTCTCTGTTC
TTTCTCATTCCTAGGATTTACAATGCAGTCTTACCTTATTTGTTTATGTTTTTTTCGTTTATCAAGCGTG
CAATCAATTACTGAAGATTCACGTGCCAAAACGTAAAAATAATAATAACATAACCCGAGAGAGGCAAGAT
TAGAATCAATTCATCTTTTCAAACAAATGGTTATATCGACTAAGCGAAGTGATTAGATGGTGTACGTCGC
AGATATTCAACAGTGACGAA

>Positive Sample 348
GGCTTCACAGGAATAAACTCCTATAATTTAAAGGAACTTGTTAGTGTTAGCACTATAAGTAAGAAAAAGT
AAATTCTGGCAGTGTTGCTTTTAATCGCAATAATATTTGATTTACATTCAAACAGTTTCATATACCTCTT
TGATAGAACATATTTGCATATGTGTTTATTTGTTTTTTATTTTTTTTTGATTACTTCTTTTCAAAAACTA
AATGTGAACAAAGAAGAAAAAGGAGTATAAAAGAATAAATAATAGCGGTATTACGTGTGTTGGAAAAAGA
ATCATAGATCGTCAATGGAC

>Positive Sample 349
CGAGTACATCTCTTTCAGTGAGTAACTTATATATTTTTGAAGTTAACATCCTTATTCTTTTTTTATATTT
TTATTTTTTTTATGTTTATTTTCTATTACTGATTTTCACAAAGAAAGGTAAAAAAAAACATACTTCAGCG
GCTATACAACCATATTTAAGTAAATGCACTCAGAGAAAGTATACAAGTAAAAAACCTAAGTAAAGACATA
AATATCCCAAAATAATGCAAAAACTCCTCTTTGTCTTTTCTGTGTTATTGACAGTAGTACTAGCAACAGC
ACCATTTCAGGTACAATGTC

>Positive Sample 350
ACATGTAAGCAAAGTCTTCCAAGAATCTGGTTTTACTCGTAGCCTTTTAACGCTGTTTTTTTTTTTTGCA
CACCTTTAAAACCTATAGAAACTCCTTCATATAAAATGTACTAGTTATAAGATGACTTTTTATATTCAAA
ATTAGAAGAAAACTTAACTAAAATTAAAAAAATAGCGGCTATTAAAAGGTATTAAAAAGTGAGAAAAAAA
GAAAAAAATTATAAACTTGAGAAATCAAAGGAGACTAAATGCATAAGCAAAGAGAAGAGGATCTCCTCGG
ATGATAGATTTATACAATTA

>Positive Sample 351
GTGTGCATATATAACATACAACATATATAATATATACAATAACCCTTTAGAAAAAAATAATAACAACCAC
AGTGGATATTTCCCTTTTGGACCTATGGCTTCCGCTAAGAGAAAAGCATGCTTTTAAGTTTTCTTTTCTT
TCAAGGAGATTATTTGGTCTTAAGCGAAGTGAGAGATTGAAACAAAATGTTAATTACAGAGTTTACGAAG
AATCAGTTTGCAAGACCACCTTATTCGTATGGCATTATCAAGCTTTGTGTGTAGACTATCCACACAAACA
TGATTCAACATACATAGCAG

>Positive Sample 352
TGACTCATCATATGCGTGAAATCTTAGTTATACTGGTAGTTGGTATTAGGGCCTAGCGTCTAATAGTTAG
TTGGCGTTTTATTTTTTTGCACCTTTCCTAATAGTATTAATCTCTAAAAGTGAAAAACTCCTAAGAGTCT
TTACGAGCTCATTTAATACGTTCTGATCAGAAGCAAGACTATAAAATGCAATAGCCAATACTTTTTAGAC
TAAAGGTAGACGACTTACTAGCGGTTCAAGCTTCAGGAACTACACTCTCAATTACGTTCTAGTCCATTTT
GTCCCTCTTTCCGGTTTTTT

>Positive Sample 353
CAGTATGAGTACGGTGAATTTCCTATAAGTTTTTAAACTTTTAGATTTTTCTATAAAGCATGTTAAACCC
TTCTTGACATCGTTTTCAAACGGCATCTTTCCTACGGAACTAGGCTGTTTTTGAGATATCCTTATTTAAG
TTCCTCTTGGCACAATGTTATTCCCGCATTTATATTTAGTTAACATTATGTGAAATTCGCTCATAAAAAG
AGCCTTAGCTCTTTCTTCATTTTGCCTCAAGAAAACTTTAAAATAAAGTAGGAATGCCAACAATACTTTT
CTTCAGTAAAAGACGAATAT

>Positive Sample 354
TTTTGCTATTTGATTTTTCTTGATAGTTTGACAATTCAATAAGTTTATAGTTTGATTTATCCTTTCAATT
TTACACTTGTTAATGACATACTGAGAGCTCATTGGATGGAAGAAAGCATAAACGTAAATCAGGGTCCTGA
GTGTCTGGGTGCTGTCTACGTTAGTACTATTCGGCTGCATTATATTCCCAATTCACAAATAAATTAGGCT

CATAACCGTAATTTTATTCGAGACATTTTTGGTTACTTCAAAATATTGTTATTATATAAAGATCATATAA
AGTTCTTGGACAAGATTGGA

>Positive Sample 355
AGTCTGAAACAATTTAATGACAAGTTTATCATAATTGTGTTATTATTATATTTATATTTAGAGGCCTATA
TTGCGCATCGAACTTTTCAAAATCCGCGGAAAATTATAGAATTCATCATATATAATGAAGGAACTGTGTT
CCTGAAAAGAACAAAAAAAGGATTAACTACAAAATGAAACATAAATATGTCTTTTATCAATTAAATTAAT
GATAATTTATTCTTACCGAACGTTTTCTTCTACCAAGTTTTCGCCTACGCACCTACATTTCCTCTTTTG
AATTTCATGGCAATAAATAG

>Positive Sample 356
GAATTCTGCCTCTATTTCCGGCCCATTGTTACCTCTTGGGTTGTGTTTGCTTTTTTTTACTTTTAGTTTA
TTTTTTTAGACTTTAATGTCGTTCTCCCTTTTTAAAGAGTAAATACATATTTAAAAAAGTGACTATGGCT
ATTGCTAAACGTGATAAAAATCAGAGCCTATAACACTCTCTGAAATAACGCTATGCAGGAATTTCCAGTT
AAGTTCTTCTTGGGGTGACTTCTTTACTCGGTATGATATGTGTTTTATATGCACAGTACGAGTCCATTAG
GGTAAATTAGTGGCCGAGAA

>Positive Sample 357
GAGCTGATAAGGTACGTAGAATCCGATGTTTCTTTAGAGAACGTTGCTATGTTAGTTTATAAGAAATAGA
GAGCTTTTAATTTTTATTTTTTTTCACTTGTATTTTTGACGGGCTATTAATTTTAAATGAAACTTTTCTA
CATGATGGAACATAAACATAAATATAGGTATAGATAAATTGTTCTTCTTTTTTTTTTTTTTTCTTTAAGA
GAAAGAGTAAGTAAATATATCTTAAAATTACAAAGGGCTTATGCATCATTTCAGTTAAAATTATTGAAGT
ACCGCTCCCTGAAATTATCT

>Positive Sample 358
GAGATCATCGGTTCAAATCCGATTGGAAGCATTTTTTTTTAATATTTTAAAATTGTTTATATTTTTGTAC
CACAAACATCATTAGAAGCTGCAAATGAAGTGAATCAGAAAATTTAAAAATGAAACTAAATACTAAATAT
TATTGTTCTTTTTCTAACAGTTGAAGTGCCAAAAGCAGTAAATTGTTTAAATATTAGGGTATAAAACATG
ATATTATAAAGTTAATGATTTAGAATGGCTCCGTCCAGCAGAACCAGTTGATTACCATTAATGTTGAGAG
TTTATGCGAGTTTTTCAGGC

>Positive Sample 359
CTTTTAGTTTTCTTTAAGGCGCTATTGGCATTCATCTTCAAAGCTTCCGCAACACAGAAATTATATATTC
ACATTTCTGAGGCAGAGAATAGTTTTGACAACGAAACTGTTAATATTTTTACTCCAGTTACCGCCTTTGA
AGTCTGATATTGGTGTACAAAGGTACTTAGGGGTATTTAAGAACAAGAAACTACATAAAATAGTTCGAAA
AGGGAAAACAAAAGTAACATCTTGATGAACCGAGAAGCCACTAACTAGTTTTTAAAAAGCAAAAGAAATT
AAATCTCTCCTTTTTTTTTT

>Positive Sample 360
TTTTAATGAAGAATGTGTGCATAACTTGATGGTACCTATTCAATATAAAATTTGATAAATAAATAATATT
ATAAAAATGTCTATGTAATTATTATAAAGCTCTAGAGTATGTATGAGTTGTTACACATAGCATTCCAATG
AGTACTTGCTTTTTTCTTCGTTCTTTAGACATTTCAGAAGAATATTCTTTATCTTGTCATTGTTACTGCA
ACTACTATTATAGTATTATTAGTATTATTGTTGGTATTGTTACTATATATTTACGCTTAAAAACTCCAAA
TTCAGACCCCAAAATATAAT

>Positive Sample 361
TTTCTTTGGCTATAGTACTTATTTAACTTTTATTTATGTTTTCGTTATATTTTCAACATTTCCGGAAAAT
AAAAAAGCGACTTTTTGAGGATATTATCCTATCATCTGTTGAGAAACCTAAGACAGTTAGTTTCTATGAT
AATACCATCTGAAACGTAAACAGTTTTAAATTTAGCAATAAAAGCTAGGTAAGAAGCCGTGAAATTTCAG
TCAACATACAGAAACGGTATATGTGGCTCCAATCAGAAAGTTCCAATACTATTAAGATGCTGACACAATT
AATGTTGGTTTTAAAATAGC

>Positive Sample 362
GGCTTTAAAGTAATTAATGCAGATGGTTCATGGCATACAAAAAATACTTTGTTTCGTTTGTTTTTTTTTT
CTTTTTGTTATGATTTTATTAGCCTAATAATGTCCTAGAGTTAACAAATGTAAATTGTAAGAGGATACAT
TACATCTACAAAAATTAAAACCAGTTAACTCTCTATCCTCATCCTTTTAATTTTGTTGAATAAGAGGCAA
CCATAAAGGTTAAGAAATAGCATCAGATGTAATGGAAGCTGAAAAATGCAATGATTGATTATTCAATAAT
AGTGAATAACGGCGTGTAAA

>Positive Sample 363
TCCACAAAAATCAAGCCTATTTCTGTATGAGGATTATAAAAAATTTATTTATCAACAACTAGATATGTTT
AGTTAATTACTTACGAAAAGAGAGGCTATTGTTTATTGTTGGCCATTTTATTTATTATTATTTTTTTCCA
CCTTAGCATAAGGAAACACGATTTGGATTTCAAAAGTAGAGCTTTAATAACAAAAGATAAACAACCACAG
TGTCAAGAACGGTTACAACGAGAAGGATAGGCTGATGATTTATCAAACATCTTCTTATTGGTTCCTAGTA
ATTTCCCTATTTTTGAGCAA

>Positive Sample 364
GGCGGATAATGAAGACATGATAGCTACTATCAAGGGTATTGGTTTCACAAACACAAGCAAAAAGATTGCA
TAGCATGTTCTAATATTATTCAATAGATATTAGACCACTGTATATAAGTTATATATATATATATGATTAT
TTAGGTGTAACTTCACGGACAACTGTCTAGAGTATAAGATTACTGGAGGATCATTGATGATTTCCTTTAA
CTCTTTTGGGTCATCTAAAATATCATCAAAGAGCGTATGCGAAAAGCGCGTTAAGAATAGATTACTTCAT
GACAAAATATAATTTTCCAG

>Positive Sample 365
CATTTGAAATCTGGACTAGGCGCAAAGTTTTGGGGCTCGGGTATAGAATCTAGTGCTGATATCATACCTA
AATAATCCGTTACTATTTCCTTCAGTTCTAGATTTTTATTTTAGTATTTATTTTCCACATATTAACATAT
GTTTAAAGGATTTTTTCATAGTTAGATTTGTTTAAAGATTTAATATGGTGACAAAAGGTAAAAAGAGAAA
AATAACAAAAAAAAAGGAAAAAAGAAGAAAATAAGGGATCGAAAACGATCTTAACTCTAGTACTGCACAAA
CAACGTAAGTGATGAAACCG

>Positive Sample 366
TGGCTATACTAGGAAAGAAAGTTTTTAAAGATGATTTGTTTTTTCTATCTTAAATCTCATATATTTATTT
TTATTCACTTCTGAGAAATACAAGTTGCATGTCTCAAAACTTTTACTTTTCAACAAAATATTAATCAAAA
AAAATAAATGAAAAAAGAAGACCATATTGAAGAACATTTAAAATCCTTTGTTTGAAATAGAGATCAAATA
TCTTGTATTAGCTGGTTTTAAATCAGTAGTATATTGCTATGTATGGTTTGATAAGATGATACAGATTGTT
AGACGAAATTATTATCATAA

>Positive Sample 367
AGTCAAAGAGCTCACTAAGAAAGTGGAAAACTTGACAGCTGTAATTACCGATCTACTCGAAAAATTAGAC
ATAAAGGATAAGAAAGAGTAAACGACATGTGTTCAAATACTGCTTAATTAATTTCTCAGAAATCCATATA
TATATATATATATTGCTCCTCATTGCAACTTTGTAAATTTAATGTTCCTTTAATTTATCATTATTTACAA
TATTAACTAATCCATCTCCCGATTTTCGGCATAAAATAAGTTTATATTTTGGTCTACATGAAAACAAAAG
TTTAACTAAACTAACGTGTA

>Positive Sample 368
AATGAAAACAAAACTTCATGCTTTTTCTTCTGTTTATACAATTTCACTTTTGATTGTTATTCAAAAAAAT
AACATTTAGTCGCAGCTTCTCAGTGTTACTAATCTTTTTCTTCTTTTTTCGAACATAAGATTTTCTGATG
GAACGAAAAAAAGGTGATCTTTTAATTATTTGTGAATCATAAATATCCTTTTCATTAATTTTTAAGTGGTA
TAGCGTATTTACATATAAAACATACATGATTATTTCATGTTTATAATCAAAAATTATAAATAATTATTTA
ATCTCTAATATAGATTAATT

>Positive Sample 369
AGATAAGGACAGAGCGTGGATTAATAAAAATGGCAGAATTTCTCGGCAAAAAAAGAAATCATGCATAATT
ACCTCGATGTGTACGTTATTGCGGGCACGATCCTGTCGCTACGGCCTGATAGTAGCGGCTATTCTGCTGG
CGAAAATTTTCAGCAGTTTGAAATCTTACTGTGGCGTCCCGGTCCAGCCGAGATGACATCTAACTAAGAA
ATGTCCGCTGCGGCACACCCACGCCGTTGCAGCTAAAGTCGCGCACCAAGCACAGACCTGCTGGAGAATG
AGGAACAAGGTTGCGTTAAA

>Positive Sample 370
ATCAATCATGACGTAAGAAATGTATCATAATTAAAAGTTGTTAAAGATGTCAGTGTTATGTTGGTGTTAC
AAAATTCTCGGCTTCTCACTAATATTTAATATCTCTTAAATTTTATCTGTCTTTGATTCTTTTAAGAAAA
GTTATGTATTATTCAAGAAAAAGTCAATTCCGCATCAAAAGGTAAAATTTATATAAACTGCTTTAAAATT
TCATGAAACTAGGCAACTTTTCGAAATGATCTTTTTCGAGCATGAAGTTTCTTTTATAATAACCTGGTCA
AAAGCTTTCAATATATAATA

>Positive Sample 371
CCGTAAGAAATTTTTATTTTGAATTTCATCAATTGGTGACAACTCATAAAAAGAAAGCTAATGCTCAAAT
GCATCGTTGTACTAACTGCCATAGTGTAAAATCCGTTTAAGCAGATCGAGTAAAACAGCTGTTGTCGATC
TTATACTTTTCTATGGAAAACACTATTTAAGCGCTATGTGCCCTTACTTTAGAATGATGACTTTACTGTA

ATATGTCAACATTTTTATTCTCAGCAAATCTTTGGCAGCGTATTTACATAGTCGTCTTTTTTATAATATT
AATGACACAAATATAAGGCT

>Positive Sample 372
TTGTAATGGAAGACAGTCGAGAGCAAGCCATTGGCTTGGACTTGCGCTTGGACCTATCTTTCGGACCAAC
TATTTAATGATTAGTAAGACAAAAATGACATTTAAGCATCTATAAAATAAGAAAAATAAAAAAATGAAGG
GAAAAAGCTAAACACTAACTTCTTGACCAAACAAACAAGGAAAAGCCAGTAATTGCGTGAACAAAAGGCA
CCTGCAATTGTTCTGATGGATGTACTTTGTCCAACCTATTCTGCAAGAAAAAGAAATCAACTCCAAAGAA
GTATGGAGCTTACTGGCACT

>Positive Sample 373
CGCCAGCAGGAAGAAAAAAGGAATCTAGTAAAAAATAAAAATTAGGTTTATAAAGTAGTAAGTGAAGTGC
AAGAGGGAGCGTTATTGGACGATCATATTGTGATCGGATCCCGTTTATTGGTCTTTGATTCAATTTAAAA
GAAAAGAAATGTACAGGTTAAACTTCAACATACCAAAAAAAAATTACAATGTGGCAATTAAACAGAGAAG
AACTAATTCTTTAAAAATAGACTTTCAGAACTATATATTAAAAATAAAAGAAATTCAAAAAAAAATTTT
TAGATTGATGTGAGAAGAAG

>Positive Sample 374
ATTGTGCTCACATGTGCTAACATGTGAGCACTCCAAGCTTTCTCTTGTCAGCTGGATATTTTTATATTTT
GGCCGGTTTATGGATAGAAAACACGTCAAACGTTCGATGGATCTTATAAAAAGTAATGATGTAAAGCAAG
GTACAAAAATGAAAGAAGTACGGTTTACTGTTATATAATTTTCGAAATCGTTCAACTCTCTTAAAGTTCC
TCACGAAATAGATATGATTGCTAACATAACGCAAAATTTAGTACAAGATATATGATTTCTTACTGCTGGA
AGATATCTTCCAATCTTAAT

>Positive Sample 375
TCTTTGTATAAATATCCCATCAATAATGTATTCTTAGGAATCTATATATGTATGTACGTTTTATGTGTAT
ATGTATTTTACGTTTTTTCATAATCATATACTTTCTGCCGAAATTTTTTGCCTTCATTATGCACACTAAT
GTTATTAATCAGATAAAAATACAGAAATTTAAAATAAATACTGAGAATAATATAATTAACTGTTCGAGAA
TTGTCGCAAATTAATAGCTTCACACTACTCCCACAAGGATTCCTAATTCACGTGCAACAGCAACCGGATC
TTTTTGAAAATGCTTTCGAA

>Positive Sample 376
GATGTTTGCATTGCTTTTTTTAAGGGAAAATGCAAGCGTTTTTATTTTTAACTTTTGGTTTTGAAGCTCG
TAATTCAACAAAAAAGAATTAAATAATCTTCAAGTCCGATAACAAGATGTAGAAAAAACATCCCAATGAA
GTTACAAGTCAAACCATTCACTGAGAATTTTTGTAACTCACCACCGATTTTTTGGATAAAATGTATTCCT
GCAACTTTTTTTTTTGAAGAGATAAAAAGAATTGAATAGAATATGCAGTAAAAAAAGAATCTCGAAAAAA
AAAGGACAAGAAATCTTAAC

>Positive Sample 377
AAAGACATATACTCAAGGAAAAGAAGTAGTAGATATATCTTCTTTATATATATTGCCCTGTGTTGCTAGC
AGGGTGTTGGAACAAATAATTTGTTCCGTATTGGAAAATTTATAATTAGGTCCAAGAACAAGTTCACTAT
GATGACGACATATTACTTTGTTCAAAGAGATGGTTTAAAATAAATATGTATAGCAGAATTATAAGAGGCG
AAATCGATCAATTAGTTGAACAATTAACTTAAAATCTAATATATATAAATATATATATATATATATATAG
GAAATATACGGCACGTTATA

>Positive Sample 378
AGTTTTCCGTTTAGCATTTATACCCATATAGTAAAAAGTTTCGCAGATAGTGCGATAGTTGTATTCTGAT
TGCCTTATTATTATTTCACTAATTCTTTATTCACGTAGAGCATGACACTTTTTTTATGTGTTTCCTTTTG
ATTGTGAAATAAAGGCGAATCCTAACAAATATTAAAATGTTTCAATACATTGATAAAAAATTGTAAACATT
ATTTTTTTGTAGTATAAAAATTCGTTCCTCATCATTAAAGAAACAAAAAGCATACAGATAATAATAAAAA
AGGCACTTTAGACAGAATGA

>Positive Sample 379
TAAATAAGCGGGCTAAACTTTGTTTATGTTTAGGTTAACGATAGACATATTTATTTTTTTTTCTAGGAAT
AAAAATGAAGAACGGGAAGAAAAGGAAAAATGAAAATGAAGAATATGAAGAGTAAACCTACTTAAAAAAA
AAAACCATACTTTAGTATTTAATGTAGCTACCACGCGTATTCGATACACTATTATTTTTCTTGACTTAGT
ATGTCAATTGTAGGTAGCCTCATGATGCCCTCTTGATCTAAGCGCGGTGAAAGTCCGCAGGCAATTGCTA
TAATACTGACAAAATTTATA

>Positive Sample 380
CGCAAATTACTCATCACATTTATTGACTACGAACTTGCTGATGTCCTTTTTTTATTTATATTTTTCTTCA
GTGAAGCGATTTTTTTTTTACACAGACCAAGACGGAAAAAAGTAGCTAAGGAAGAAAACAAAATCATGAA
AAAAATGTGAAGTGATCATGCACATCGCATCAACTTAAACATTGGCTTAGAGATATATAGAGTTAGAGTT
TACGGCAACCTTTAAGCACCAATACCTTTTGGCATAGTCTAAAGACCTGGTTCTTAATTTTAAACAAATT
TAACTAAAGATTTCCCTATC

>Positive Sample 381
AGCAATAAAAGAATGCCGTTGTGACACTTAAATAGTCATATCACATGCGGATACACTTCTAATACACTAG
TCTTCTTTCACTGCCCTTTATTTATAAGACTCATGTCCAGTCTATTTTTTTTTAAAAAAAAAGACTTTC
AAGCTTTTTACAACTTGATATTCTAATATGAATAATGAATAATGAATAATTTAATTAATTGTTTTGTTTA
AATTTTGTTCGGTCTCGGCTATATTTGGAAGAAAAATAGCCAGTACATACTTTCTACCACTTTCGCTCAA
TTATCAATAAAAACATTTAA

>Positive Sample 382
CTGCCATCTGTCATGGACCGCTCCTTTTCGATGGATTAATAGATATCAAAACAACAAGACCATTAATCGA
AGGCAAAGCTATAACAGGTTTCCCACTCGAGGGTGAAATCGCCCTGGGAGTTGACGACATCTTGAGGAGC
AGAAAATTGACAACGGTTGAACGCGTTGCAAACAAGAATGGAGCCAAGTACTTGGCGCCAATCCATCCCT
GGGATGACTACTCTATTACAGATGGAAAGCTAGTTACGGGTGTTAACGCAAATTCTTCCTATTCGACCAC
AATTAGAGCTATAAACGCAT

>Positive Sample 383
CAATCGTTATCAACATTTTTTATTTCCAGTCCTACCACAGCATTATTGCATAATTAATTTACAGGAAGAT
TTTCTTGAAAAAATAGAAAGAAAAATACCCATTTTTTCAAGGAAAATAAAATTTATATATTACATACATT
TCTATAAAACAGTTTAAATTCAAAAATATAAACAAAAACATAAAAATAAGAAAAGTTCAAGTCTTCGTTA
TTTTCTGTTTGTGGAGTAATCGATCTTATTAACATATATGTGGAGACTTTTCAAATATAGCCACCTGCAA
CATGGAACCATAGTGTACAG

>Positive Sample 384
CGAGGAGAATGTGTGTGTATGTGTGTGTTGTTAGGGCATTTCATATTCTATATTCATTGTTTTTTTGTTT
TTTTTGTTTTTTCAGTTCTTTGTTTACCTATTTTTTCTTTGCCGCAATTGCAAATATTATTAACTTAATT
GCGATTATTGAGACTAAACAATAAAGACCTGGCAGTGGAGGCATGACAGAGGATATCGCGAAACGATTGT
TTAAGAGTATATTATAAAGTTGACTTGTTTCCTTATAATTATAATTATAAATATATATATATAGATTATT
CACAGGAGAAATTGGGGGCA

>Positive Sample 385
AAGTTTCAAATATTCCGTAGAGTATAAATATCTTACAACTAATATAATTCTTAATATGCGCAAACAAATT
ATGGTGCGCATCTTTTTTTATTTTTATGTTTTGTTCCATACTAAATGCAGTTTATAAAATTTTTCAAATG
AGTCAAGCTATGTATAATCAGTGCTACAAAAATGATAAGATGCAATTGCAAGAAAAAATCCCTTGCATAA
AGGGCTTTCTGACCTCTTGCGTTGATCAACGAGAAATAACCGCAAAACTTAATTTTAAACTCCCGCGTCT
GCTTTACACAGCCCACAGTA

>Positive Sample 386
AAGAAGCATTTATTGCTAGTATCCAAATTATTAGTATATCATCATGTTTTCAAAAAAAAATGATGTAAAA
ATTGAGAAACAGTCACCAGTCATCATGAAAAGTGCACTGCAAGGATTAATAACGTAATAAAACAAGAACG
ACAGTACATAAACGAAAGAAGAGATAACTATATTATTGTATAGAATTATCGATTCTCTTTTAGAATTCTT
TTATTCTCCAGGAAAACTACTTGTACATTCTGTTTAGCTAATATTGTAGCCTTTAACCACAATAAAATGC
ACTCCAAGTATAACCCAAAA

>Positive Sample 387
GTAGATCCTTGCCGTTTATATATACATGTAGAGCAATTCTCAAGCTAATAATTAATGTTTACTTCAGACA
ATAAAACATTAAAAACAAATTTTTAGAAAAACAAAAGCTGAAGTAAAGACTAAATATTACACAATTTATT
CATACATTTCCGCCCCAAACTCAAACAGCCTAAATAATATAAAAATTAACTTTTGGCAGCTCCAACCTCT
CTACTGCCATCTAAAACTTTTCCAGTAAACGGCTTATATACAGTTAACAACCCGTCACTATCAACTGTAG
CAACTAGATCCTCAATGTAT

>Positive Sample 388
AGAAGTGGTGGAAGATGGTGATACCAATGCATATATAATGTTGTCTTGAACCCACTAAAGAAGTGACGCA
TATATTACTTTTTTGTATTTAGTAACACAATTTTTCTATTTTTATATTTCCTTGTATAGTTAGTAATTTT
AATTTTTTTCCTTCTTTATCTCAGCTCTTCGACCATTTGGTCAAGAAGTTAGTTTTTTTCTCTTATTTA

TTTGACAATATACTAATTTTATTATATGGTATTGTAAATAACATTTGAGGAATAAAAAGACAGTCACTCT
ATAAGCTTTATCAGCCTGAC

>Positive Sample 389
CTTACACTTCGTTCCTTCCCTTCTGTTCACCTTTTTTATTATGCCTGACTATTCTAAAATTTATGTTTA
TGTGGCGTAAAATATCTTTCTTCTGAAAACACATGCAGCAGTTACTCCCATCATAAAAGGAATTAATAAA
TGAATAGTATAAACAATTATTTACTTAAATATATTTAAATTTCTGCTTGGAAAACCGGCCATGTCGGCGG
CACATAAAAGTTCTATTTACCTTTAAATTCTTTTCGACATGTATGGACCGTCTAGTTCATATCCTAGTTT
ACCATAGTAATTTCTTACAC

>Positive Sample 390
GATCCTAATCACTCCAAGCCCAACGGACAAGCCCAAACACATCAAAGGGGTCCGAAAGGACAATCACATG
CACAGGTTCAAAGTACTAATAGCCCTGCGCTAGCGCCAATTAACATGTTCCCGAGCTTAAGTAATTCTAT
ACAGCCGATGCTTGGCACCTTGGCTCCGCAACCGCAAGATATAGTACAGAAGAGGAAGCTACCGTTACCA
GGTTCAATAGCCTCTGCAGCAACAGGCAGTCCTTTTTCTCCATCACCCGTTGGTGAGTCTCCCTATAGCA
AACGCTTTAAACTAGACGAT

>Positive Sample 391
AAAGAAAAACTAACTTCGGTTCCCTTATGATTTGATATTTTTCTTTCTTATTTACAATTAGTTTTCTCAG
AACGATATTCAATACAGTTCTCCGGATTTTTGCTTACTATATTGGCCCCAAAAAAATGAAATAAAAAAAG
AAAACAAAATAATATACTTGGGTGAAATAAAAAAAATAAATCAATAGCACCCAGTTAACCTAGATTCAAA
GTTTCTTCTTCAATCTCAATTTATATACACGTTTGAGTTAATGGGAAAGCAAAGTCCAGCTATAAAACAC
TTGACCGTTAGCGCCATACC

>Positive Sample 392
GAATTTAAGGTATCGTACAGGCCCTGCAATTCAAGAAGAATCAGATAATGAAATTGAAGATTTACTTATT
CACAATTTCAATTCCAGATACTGTCATGAATGAAGAGCCGCCCCATCCATAGTTTTTCCTAGTTCAAGCA
TTAATATACCGGACCACTCTACATCTTCCGAAAGCCGGAAAATAAGAACAAAAAATAATAAAAAAAAGCA
CGTTTAATATAAAAAACATTTCATTGTATGAGTTGGATTCTTTTAATTTTATTTTAAAATACGGTTGCAT
TTTGCATAGTAATATCCAAC

>Positive Sample 393
TCCGATGATGGTACTAAAAATTTTATATTTAAAAATAAATAGTTAAGTAGTTTGTAAAGATGGTATATTC
AGCGGATTCTTCCTCTATTTTTATGTTTTTTGAGATATTTCGAGCTTTCTATTTTTTTAAAATGCCTGAA
TTTCCATCTTCAAAAAAAACATAAAAAAGGTACTTTACCACCAAATAAATGATAACCACAATTTACTTTA
CTTATCCTCCACTATCAAGTATCTTCCTACCAGGAAAAATGAATTTCAAATGATTCGAAATTTACGGAGG
CTCAATATAGAGATAGTGTG

>Positive Sample 394
CTCCTCATATTCTCCCATCTGGAAGAACTCTATTATTCCTTTGCCATTTCTCGTAGTCTTTTATATTTAG
TCCGCTTTACACCGAAAAGGTCATTTTTAAAAAGGCAAGAGAACTAGGAAAAGTAAAACGTGCCCCCTTC
AAAGCGAAGAGATGAATATATTTAGAAGTTAAGAATTTATTTATTTATATTTACCAAGTTTTTCAGTTAG
TTCAGGGACTATTTTGTAAAGATCCCCTTGCAACCCATAGTCAGCGACATTAAATATAGGGGCATCAGGA
TCATTGTTAATGGCAACGAT

>Positive Sample 395
CAATTAGCTGCTTTACCGTTAATGGTAGTAAATTATATCACTAGATTTTGTTTGTTGATTATTTTAATTG
TTTTTAATTTTTAGTTAGTAGATTTTCGTTCGAGGCCCTAAAAATGTACCGAAAAGTCATAATAAACGGG
CGGAGTCACATCGTAAGAGTAACATATACAATATGCGAGCAGACTTTGTCCATTAAGCCAAGTAACAACA
AAAGAACCAAAAAAAGGAAAGTATCTCGTCTCTGTTGAGTAATTCTTATATCTTGCAAGATTTAATTCTC
ACTACTAATACGGAACTCAA

>Positive Sample 396
TGTTAGCGAAATAACGATTAAAAAATTTGAAAGTCAATACTATGAAACAAGGAAACGAGCAACTTGGTAT
ACTTCCCTCACCTTTGTTAAATTCACTTGGCCTTACTGGCAATCATAATCATTTTGACGAACTGCGGACA
TCGAGAGTACCCTGCAAATATATCTAGAAAAAGAGGTAAAAAAATATTGAAGGGTCAACCTGAAAAAAAT
TGAAAAGTGAAAATAGATCTGCTGATAAATAATGCACGGCTTGCATCAAGTGATCACATGGAACTTTCCC
AATACCTCAACTACGCATTT

>Positive Sample 397
GAAAGGTCGTCTTTTCTTTATTCTTTAGCATTTCAATACCGAACTAAATATCCGTTTTTATAGTTTAGTT
GGTTTTGTTTAATTTCTAGAATCCTGTTCGGCGCTTTTGTTAAAAGTAAAAAATGAAAATTCAAACGAAA
TGAACCTAATCACGTTAGAATTTAAATCTTGGAATATGTTAAATATAAAACAAGAAGAGCAAGCAATACT
ATTTCTGACTAATTCTCTATACACATAAGAGCAAAACAAAATGTTCGGTAGCAAACGTCTGAAAAATGTT
AAGGAAGCATTTAAGTCCCT

>Positive Sample 398
TCATATTACAGTACATACACCTTTTTTTTACTTTTTGTTTCTTTATTAATTTTTTAGTTTTTTTCATATT
CTATTTTATTATAAGTAGTAATATGAAGAAATTTCCCAAGCAAAGCTCAATAAAATTATTTAGCTAAACA
AACATATGCATATATATGTATATTCTCCCTAATTTAATTCCTTGGCTGCCCAGGTTGGTAGAACAAAGGA
AGCTTCGTGAATTTTCTTGTTATAGTATCTGTATAATTCAGCCTCCTTCTCATCAGAGATTTCACGTAGT
GGCTTCTTGACATTGCAAGT

>Positive Sample 399
GCTTTGTAAGCACATCTTGTTTTAATGTGTATGAATTTTTATATTTGGTTCGAAAATTATGACATTCGGC
CAAGAAACTTTCAGAGGAGCTGTGTATTAGCAGATGGTAAGCGCACGTTGAAAATGCTGAATATTATTAA
TTTAGACATACCTTGATGGCTGAACAGTAGTCAGAAAATTAAAAAAAAAAAAAAAGCGCTTATTACGGCG
TATACACGTAACTATATAGACGTAGATCATTGAATATTGATATGATATAATATTTTCCCCGGGATTGCAG
AATACTGACACGCCTTTTAT

>Positive Sample 400
ATTATGAAACCAAGGCAACATGTAATTTTTAAGTTTAGTAATCTTTTTCGGGATGAGATGTTTTGAAGAG
TTTTATGCTATTAAAGTAGTAATATGATTTATATATTCAAATAAAATGTAAATTATGAAAGAAAAATACG
ATAAGCTGATAGGGTAAACTAGAAACCTTCGTTTTCTTCATCATCCACATCCAGTTTCTTTCTACCGCCC
CCATTTCTTAAGGCAGCCATCTCATTAGTGGCAACTTGCTTTCTAACCTTCTTTGTCATCCTGGCTATCT
CTTCTTCAGCGTCAACTGCA

>Positive Sample 401
TGTGTTAGTTTTTCTTTCTTTCTTTCTTTGTCTTGACGTGATTTGGACTTCTGTCTTGCATTCGCGTCCA
TTCATCTGACCCAATATTCCTTTTGGTTTTGTTATCCTTATAAAAGAAAGGAAGCTTCTTAGAGGGAAA
AAAATGATGAAGAGTAATGCCAAAATATAAATAAATAAATAAATATGAAAATCATTTTCTATTTTTAATA
GAATAAGAAGAGCATCTTAAGATTACAATTTCAAGAAATAGTTTACACAGTATATCCAATAACTCCAATA
AACTACTTTCCTATACAAAT

>Positive Sample 402
CCCTATATCTTCCCTCCTCCTTGCAATTGAATTTTGTTTGCATTCTATATTATCATATAGTTTCATATCA
ATTGCAAAAACTTTTGCAGCCAATGTTACAAAATTGTTTTCAATCAAAGAAATACAGGTGTGGTGTCCTT
CACCATCGATATATTAATATTAATATATCATGAATAAATATAAAAATTCAAGGTAATGTACTTGTGCACT
TCTTTTAGAATATAGCAATTTCCCTTAGCTCTTTTTCAAATTGTAATTCTCTACAATACATAAAAAGAAA
TTGATCTTTGGTTCAACTTT

>Positive Sample 403
GAATTTTTCACCAATTGAAGAACTTTATGACAACAGAATGTTCTGATTGTAATATAATGACAAATTTTAA
TCTTTAGTAATATCTAATATAATAGTTAATGATAAATAGTTAATTCTAATGTTTAATGATCTGCAAATAC
TTTTTATGTTTTTCAACATAGTTTTTAATGTGTTTCTACACTTGATCGCAACTAAATCTAAATAAACACA
CAAGCACAAAATATGATAAAAAAGCAATTTGGCTAGAAGCACAGGAACCAAATATGACGTTCAATTCAAA
TAGACCCTTATTTCTGGTCT

>Positive Sample 404
TTGTCAGTTTCATAACAGTTGTTTCATACGATTTCCACATGTGTCTCATATATATTTTATGTTTAGGTTA
ATAACTTTGGTAATGCTAAATTATCTTAATTTGACGTACACAAATTCTTCCTACTGTTATATATGTTCGA
ATTATCTTTTAAATTGTTGCTAAAAAAGTTAGAGTTATCACCAAATCAGTTGAGCAGATCACCATTATTC
TTATGTAAATACTTGTTAAAAGAAGTAGCTTTCGCAAATACACCAATGCATTCGTTGATAATTTACGCAG
TAACCTCTTACAGAACGCCT

>Positive Sample 405
CTATACAAAGCCCATGTTGGCTACATTAGTCTTTGAATCCTTTAGATTGCCTGAATCAATAGCTCATTCA
AAATATTTTGGACAATCGATAGTTAACTGTAACATGTGCTGCTATATAGTGGCAATCTAGATGCATGTAT
CTTATATGATTAAGTATAAAAGAACCACAGCAAAGCTAAACGTAAATAAGTAGTTTAGAAAGTACTGGAT

ATGAGTTAAGAATCTGTTAATTGAGCATGACATGAAAATTTTGTATTAAAGCAATATGAGTCGTCACATT
AAATATATGAAACTACGGGA

II. Negative subset contains 406 replication origins with each having 300 bp.

>Negative Sample 1
CCACACACACACATCCTAACACTACCCTAACACAGCCCTAATCTAACCCTGGCCAACCTGTCTCTCAACT
TACCCTCCATTACCCTGCCTCCACTCGTTACCCTGTCCCATTCAACCATACCACTCCGAACCACCATCCA
TCCCTCTACTTACTACCACTCACCCACCGTTACCCTCCAATTACCCATATCCAACCCACTGCCACTTACC
CTACCATTACCCTACCATCCACCATGACCTACTCACCATACTGTTCTTCTACCCACCATATTGAAACGCT
AACAAATGATCGTAAATAAC

>Negative Sample 2
CCCTGTTTTACTTCTTTTATAGAACCTTATTTTTGACGCAGGGAGGCGACATTTATCCAAATTAAGTTTT
GACATGGCGCATCAGGGAATAAAAAAAACTTTATTATGTGGCCGAATCAACATTAATCAAATGCACTAAT
ATTGTAACGTTCTTACAAAGGGCAGACAACTTGAGAACTTTCATGCGTGCAACAGTATTAATATTTTACT
GTCTTGATATCGTTATCCTCATCGTAACGTGAATTTTTTTGTCTCATACGTTAAGGTAAATTTTGATGAC
CCCCGTTGTCCTTGTTTGCC

>Negative Sample 3
AGCAGAACATGCAGTGAAAGTAAACCCCTTTAAATACCTGGGAGCTTCTTCTGTTTTCCAAACCAAAACA
CTTATCCATGCGGTAGATGATTGAGCCATAATATTCATTGTAACTAAAGTAATAGCTCTAGTTTGAGCAT
CTCGGCGACAAATATCGTTTTGCCAAGAGTATAAAACAGGAGCCATAGCCCAACCAAAACATTGCAGCAT
AAATGCAAACCATTTGGCTCCTTCTGCGACGTCCCAAGCGGCTAATATGGAGTTACCAATGATATTGAAA
ACCTGAGTAAAAATAATCGC

>Negative Sample 4
CTATGGACGTGCGGTTATATGATCTTCTAATAAAAATCTCTTAGACTACGGTTCATGGAATACTTCTTGA
CTTCTTGGCGAAGGAAAGGCGCATAAATGTTGTGATCGAATGACCAGTAATACGTCGTTTGTTCTCTTGC
TGAACAGCGAGGAAGAGATATTCATCCAAATGCATGAACGCAGAATCCTGTAAAAGTCGTAAAAATGTTA
ATCGCAATGTTGTAAAAACCGTCAAGGCATTTATCGTTCAAAATGGCGATCATTGTTACTACTAAACACT
TACTGTTAAATTAAAAAGCT

>Negative Sample 5
TAGAATTAAATGGAAACATTTACATATTTGGGGGATTGATGCCATGCTACAGCTATGAGGAGGATGCGCC
GATGCTGAACGATTTTTTTGTAGACGGAATAAAGAACTTACCTCCCCCTTTACTACCTCAAGTGATTAAT
AATCCATCAATGGTCAATAATCCTCATCTTTATGTCGCTTCTATACCATCATGCCGGTTTAGCAAACCTA
AAATGGGGGGTTATATACCGCCTCCATTGCTATGTGTTCAAGGATCCAAATTAACGGACCGACATATTTT
CTTTTATGGCGGATTTGAAA

>Negative Sample 6
TCCCGATGAAAAGAAAAAAGGGAAAAAGTCGATAAAAAGAGGTAAGCGAAAAGAAAAAGAAAAAATAGAA
AATTTGGGTGGGGGGGGCGGAAGATCCCACGCCGCGCAAGAGATATTTCAATATTACTACTACATAGTAT
ATGCGGCGCTACCATACGTACAACTTTTTTTTCTTTTTTTTTTTTTTGCCTTCTAAATTTGTAATTCGG
TCACACTTTTGTCGCAGTGTTGCAAACGTCTTGAAAGAATTGTAGGTGTTGTAAACCACAACTTGCTCCC
TTGAAAGCGTTGCTGATTAT

>Negative Sample 7
ATTACTATTATATCGAAACTCCCGTACATGGTGAAACAGGATGTATAGATCTTTTCGATCTAATTGAATT
TAAAACCAACATGACCGAATTTGAAGCAAAATTGATATTCAAGCAGGTTGTAGCGGGAATAAAACATCTA
CACGACCAGGGTATTGTTCACAGAGATATCAAGGATGAGAATGTTATCGTAGATTCTAAAGGCTTTGTTA
AGATTATTGATTTTGGATCTGCTGCGTATGTCAAAAGCGGACCATTTGATGTTTTTGTTGGGACAATAGA
TTATGCTGCCCCTGAAGTCT

>Negative Sample 8
TTTAATGTGAAGCTAGTTAAAGTACAGTCTACGTGGGACGAGAAATACACAGATTCAGTAAGATTTGTGA
GTCGAACAATTGCTTATTGTAATATTTATTGTTTGAAAAAATTTCTGTGGCTTAAAGACGATAATGCCAT
TGTGAAATTTTGGAAAAGTGTCACGATACAATCCCAACCGAGGATCGGAGCTGTGGATGTGAAATTAGTC
CTCAACCCCAGAGCATTTAGTGCAGAGATTAGAGAAGGATGGGAGATTTATAGAGACGAGTTTTGGGCCA
GGGAAGGTGCTAGAAGACGC

>Negative Sample 9
TGTCTAACCAAGCCCTCGACTGCGTTGTTAATACCATATGCTCCTCTAGGGTTCAAGGTATATATGAAAT
TTCTCAAATAGCTCTGGAGTCGCTTTTACCCTATTATCATTCAAGAACAACAGAAAATTTTATACTGGAA
CTAAAAGAAAAAAATTTCAATAAAGTTCTTTTCCATATCTATAAAAGTGAAAATAAGTACGCCAGTGCGC
TTTCACTTATTTTAGAAACTAAGGACATCGAAAAAGAATATAACACGGACATTGTATCCATAACCGACTA
CATACTCAAAAAATGCCCAC

>Negative Sample 10
TCAATGATAAAACCACAGCTGAAGAATTGCAACGGCTTGATAAATCTAGCAATAATGACCAGCTAATTGA
ATCTTCTTTGTTCATTGACATTGCTAATACCTCTATGATTTTAAGAGACATACGGAGTGATTCAGACAGC
TTATCCCGCGATGATATCAGTGATTTGTTATTTAAGCAGTACAGACAGGCAGGAAAAATGCAGACCTATT
TCTTATACAAGGCATTGAGAGATCAAGGGTACGTTTTGTCCCCAGGTGGACGTTTTGGTGGGAAGTTTAT
AGCATACCCTGGTGATCCTC

>Negative Sample 11
TTCTAATATGATGCAAAGCCATCAAAAATCATGCATAGTTATGAAATACCTGATGAAACGCTTCGAGTTC
GTGCTCAAGAAATTACTGAAAGGTTACCGAGAAGAAAAATATCTATGAGACACGATAAGGCCCCTTCTGA
ATCCATTGTCCTGGGCTTGTTCATTCTATTTACCACTTAAAATTGATCCTTTCAAAGGAATTTTTTTCTA
TTTCCAATAGTATATTTGTACAAAAACTACAAAAATGGATAAAAAATAACAGTAATTTGTGACTACTGTA
AATATCACTGATTTGGATTT

>Negative Sample 12
GCTGGTGTAAAAGATGGTGTTATTACTTAAGACTGATTTGGTTGCTCAGGTATTTCATTCAATAAAATTG
TGAAAGAGAACCTGGAATATAGAATGGAAATATATATCCTGCTACTAACCCCAATGGAAGGTGACGATCA
CTTCTTGTGCGTTCCAATCCCAGTTTTTGAATGTGCGAGTGGAAAAATTCTAGAGGAACAAATTGATATT
TTCAAATCAGAATTCATCAAATATATGTACTCTGTAAAAACCAAGGATGTGATGAAGTTGAGATATTGTC
TTTATGGGAAATTGACTTCT

>Negative Sample 13
CAGCATCTGGTTCAATCGCTTCTACTGCTTCCACCGCTTCCACTACTTCTACTGCATCCGCTGCATCCGC
CACCAGCTTCACCTCAGGTTCCGCTTCTGTCTACACTACTACATTAACTTACTTGAATGCCACAAGTACA
GTCGTGGTTTCCTGTTCAGAAACAACCGACGCTAGCGGTAACATTTACACCATTACCACAACTGTCCCAT
GCTCATCTACCACTGCCACCATCACATCTTGTGACGAAAACGGATGCCATGTTCCAGCACCAACTGCTAC
CGACGCAACTGCAACCGTTT

>Negative Sample 14
CAGGTACCATACCCCTCGGAAAGTTATCTGACATTGACAAAATCGGAACTCAAACGGAAATTTTCCCATT
TTTGGGTGGTTCTGGGCCATACTACTCTTTCCCTGGTGATTATGGTATTTCTCGTGATTTGCCGGAAAGT
TGTGAAATGAAGCAAGTGCAAATGGTTGGTAGACACGGTGAAAGATACCCCACTGTCAGCAAAGCCAAAA
GTATCATGACAACATGGTACAAATTGAGTAACTATACCGGTCAATTCAGCGGAGCATTGTCTTTCTTGAA
CGATGACTACGAATTTTTCA

>Negative Sample 15
ACGACCTCGCGCTTCGAAGTAGAGGAGCCCTTTTTGGCGTACCTACATATGGCGCGTCAGACAGACAAAC
TTCCCCCAAAAATGTATTACCCCGCCGAATAAGAAAACAGACCCATTCACCCACGACGTATCAAGTTACT
TCCTTGGTGCAATGTCCCACTATAAAAAAATTCCTTGACGCTAGATCGTTGGACTAAAATCTGCGTCACA
ATCGCCTAAACAGGAAATATTGCCTATTTTCGTACAAGGTTACTTCCTAGATGCTATATGTCCCTACGGC
CTTGTCTAACACCATCCAGC

>Negative Sample 16
CTATTTAAAAGAGTCACCTTTCCGATGGACCTTCAAAAATATAAACGTGGCTTATCTCAGAGCTAAGACA
GACCTTCCGAGCAATAATAATGAGTCGTTCTATTTTTTTTTCTCGTCCCTTTTTTTGTCCCCTCTTAAC
AAAATTAGAAATATAGGCGGGAAAGTGGAGAACCCTTGGAAGAGTCAGATCAGAGATTGGAAAAATTAAG
ACCAATGGATATCTACAGATCCTGTGAATCGAGTGTGAGTGATATACCGCAAGATCGGTAGAAAACTGTA
TATAAAGCAAAGAACTGATG

>Negative Sample 17
GGTCGTGAAGCCTACCCTGGTGATGTCTTTTACTTGCATCCAAGATTGCTAGAAAGAGCCGCTAAGCTTT
CTGAAAAGGAAGGTTCTGGTTCTTTAACTGCTTTGCCTGTTATTGAAACCCAAGGTGGTGATGTCTCCGC

TTATATTCCAACCAATGTTATTTCCATTACCGATGGTCAAATATTCTTGGAAGCTGAATTATTCTACAAG
GGTATCAGACCTGCCATTAACGTTGGTTTGTCCGTTTCTCGTGTCGGTTCCGCTGCTCAAGTTAAGGCTT
TGAAGCAAGTCGCTGGTTCC

>Negative Sample 18
TTAAATGGCATCTTAATATTTTGTGAGCGGTATTATACCTTTCTTGGGTACCTAATACTTCTAAGCAACC
TGAATGAAATTAGAAGGTGTTCACTCCTTCATTAGCAAGTGAGCTTTGCCGTTGTCACCGTACTTGGTCA
TCTTAAGAGATACAAAGGGGGGGGACCCTGAGAAAACTTCTCCCGAAAAAAAAAAAAGAAATATACCCAC
GCGTTCTTTACCCGGAAATAAATATCTTGATTTAGCCGCCGAGATTGTTATATATGCATCCAAGACCTCT
GAATGGTGGCTAATTAAGAC

>Negative Sample 19
TCATGTCATCACAATGGATCTCCATGCCTCCCAAATTCAAGGGTTTTTCCATATCCCGGTTGACAACCTA
TATGCAGAACCAAGTGTTTTAAATTATATTAGAGCCCGGAAAACAGATTTCGACAATGCTATTTTGGTGT
CGCCTGATGCAGGTGGTGCTAAGAGAGTAGCTGCTTTGGCTGACAAGTTAGATTTAAATTTTGCTTTGAT
TCACAAAGAGAGGCAAAAAGCTAACGAGGTTTCAAAAATGGTGCTTGTTGGTGATGTTACCAATAAATCA
TGTTTATTAGTTGATGATAT

>Negative Sample 20
GAACAGTTAGACTTCTAGTCTTCCCACCCTTAGATAGCATGTACAATTGAGGTAGAATAGCCACACTCTC
CAACCATACAGAAAAACTCCATGCTAATTCAAGAAAAGTGAACTTGTGATGGAAAAAAACACTCATTAGA
GCACTCCCAATTAGTAAATGCTGGATCTTAAAGGTATCATGCATAAGCATTTCATTATACGCAATGGTGT
TGGTTCTTTTAGACCCTTGTAATAGCACTACAATGTAAGCGGTAGATACAATGAAAAATATTTTCATTAG
AGCATTGTATAGGGATACCC

>Negative Sample 21
AGAAACTAAGGTGAAGGAGACTGTTCGTAGAATAAATGAATATTGATTAAAATTTTCTGAAAAAAAGAGT
AATGAAGTCGTAGTCATCGCTAATGTACCGTAAGTGAGTTCTGAAAGGAGGCGGGTTCTTGTTTCAACGT
AGCTTGTCTAGTCCTTTATGGCATCGTGTAAAATTGGTATATATTCAGAACACCCAGGAGTGAATTGATT
TGCTGCTATAGGGGGAAATAAGATGTAACGATATTTTTGTACTGAATTTCTTTGATGATACTGCCAGGA
TTGAGAGTTTGGTTGCCACA

>Negative Sample 22
CTCAGAGATTCTAAAGTAAGTTCTTCTCTTAATGGGTCAATGTCCATGTCACTCAATAGATCGTCATCAC
TATTCTCTAAGTCCTCATACTGCCTTCTCCTTCTTCGTCTTTGCACAGGAAGGCCCATTTCATCAAGTTG
CGCTGCACCTTCTTGTTCTTCGTCCTCGTCATCTATGTAGGCAACATTTCTCAATAGCCTATCTCTTTCA
TTTAGTTGAGCATCAATGCGGCGACGTTCGCTTAAAGATAGTTCTTGTTGTTCCCTGTCGTCAACTTGAT
CTGGATCATATCTATCTCTA

>Negative Sample 23
CGCAAGCCCGGAATCGAACCGGGGGCCCAACGATGGCAACGTTGGATTTTACCACTAAACCACTTGCGCT
TATTTCTTGGAAGTGTTGTATCTCAAAATGAGATACGTCAGTATGACAATACGTCATCCTGAACGTTCAT
AAAACACATATGAAACAACCTTATAACAAAACGAACAACATGAGATAAAACCCGGCCTTCCCTAGCTGAA
CTACCCAAAAGTATAAATGCCTGAACAATTAGTTTAGATCCGAGATTCCGCGCTTCCACCACTTAGTATG
ATTCATATTTTATATAATAT

>Negative Sample 24
AAAATGACCAAAACTTGATACTGTACTTATTTTTCAAATACCAGGGAACTCCTCTATCAAGGTGCTCTAA
CATTGATTACTCTCAAGCATTGTCTATTTTCTGGCAGTGCAGCAGTATTTTATATGTTGCTGAATCCAAA
TTTCAGCTCGAACACAGGAATCTAACTTTGGACCATATTTTGATAGACTCTAAAGGGAATGTTACTTTAA
TTGATATGAAGTGCTGTCGTTTCTTGAATATAGACAACAATAAGGCTTCCTATACAAGATTAGACCATCA
TTATTTCTTCCAAGGCCGGG

>Negative Sample 25
CAGTTATTATTAAATTGTTTCTTAAACTCTCCTTAAAGATATACCCTATTTAGGTTCACTACACGAGCAT
TCATTGAGAAATTTTTTTTAAAACAACATCGATCTTATCTTGAGCAGTTTGGTGCTTATCAGTTCTAGTC
CCAACACGAATATCAGTGTGTACTCTAACATATCCTTTCTCATGACCGTATTCATGGATTTCGCCAATCA
AACCCATCACATCATCCCAAGGCCCTTCAATTGTTGTTCCTGCACTGTGTAAAGTGCTCTTTAATGGGCT
TTCTCTGATTTTTTTTTCAA

>Negative Sample 26
CTTTCCTGTTCCCCTTTTACCTGACTAATTATCCCGCCCATTAGTCAGATGATTGATACCAATTTTACTC
AAATGTGTACTATCATGTCCCTTTTCGCACTGTTATTGCTCCTTACCTAAGCAAATATCCTTTTTTTTT
CTTTGCATTTGTCTTCTTTCATTTGAAATCTTCATAATCTTCCGGAAAAACTAGTGATGCGCAATATTGT
AGAGAAAAGCGCAGGGCATTCCTATACTTAGTAATCACCATCATAACGTATTTAAAATGAAGAAGCGAA
CCTCATAGAATTTTCTATAC

>Negative Sample 27
TTCAAAAAACCCTAACTCCGCTTTATGGTCCCAATTAGTGTGCATTCCATTTTTGTTTTCCATCACTTGT
TTAATTGGAATTCTAGTCACCGCAGCTGGTTATGAAATATACGGTATTAATTACTGGTCGCCACTCGATG
TGCTAGAAAAATTTTTACAGACTACTTATAATAAGGGCACAAGAGCTGGTGTTTTCTTAATCTCTTTTGT
TTTCGCCGTAGCTCAGTTAGGTACTAATATTTCTGCAAACTCATTATCGTGTGGAACTGATATGTCCGCT
ATTTTCCCCAAGTTTATCAA

>Negative Sample 28
ACATCGTTTTGACTTTTCACTATAATCTTGTTCTTCGCAAGAAAATCTCTGATATATCGTAATCCGGCCT
TGATCCCATTGGTCACTTCTTTTTCGGGATACTTGAAATAGTGCTCCAGTAAAGAGCATGTCAAAGATGT
TGAAGTTGCTGTTAAGTTGTCCTGAATGCAATCCATGAATAATTGTAGACCAACCCTCCCCAGCTTTCGA
GGCCTGTTTGATTGAAGTCGTGTGTTGGTAGCTTCATCTACTTGTAGGGAATCTAGTAACTCACGCATTA
CATTGTGAAGTCTCATGGTA

>Negative Sample 29
CAGTTAGCCATTGAATGGTGTAAACCACAGAAACTGCAAAACCCAACGAAGGGTCGACTAAAATAGAAGG
ATATCTGGTGTAATTGCCGGTTAGTCCTGCATAACAGAGACCTAACTCGCCTGCCGCTTGGATGATACAG
TAAAGCATGATCGATGCTATTCCGTAACCAAGGACTAACCCGGCAGGACCAGCTGTTCCCAGCACCTGAC
CATTACCGACCAGTAAACCAGTACCTATACCGGTACCGAGACTGATCATGACTAAGTGACGAGATTTGAT
CGACTTTGTCAGGTTGTTTT

>Negative Sample 30
CAAGGTTCATGTTTTACATAATAACCGAAACTCAACAGAGAGATCTCCTTACGAACCAATAGTTGCAGAG
TTACTTTTGGAGGTGAAGGAGTTCAGGGCTTGCACTTTAACTTTTGAAAGTAGACATCAGGCGAAGTCAC
CTGTCAGAGAAATCACGGTATATCAAAAGAAAAATTCGGCCCCACAGAAAGCTAATATCACGAAGACGAT
CAAGTCTGCTTCTGGCATAAACGAACTGCAATTGCATAAACTTGATTTCGATCAGGAAACTTACCATATC
GGAGTCCAGTGGTTTCCAAA

>Negative Sample 31
TAGATTTATTCTCTGGATTATTTATTAAGATGAAAAATAAGAATAAAGAAAAAGAAAGAAGGATGAAGAA
AAAAGAAAAATACAGGCCAAGGCCTTTCAAGGTGAGCTGTTGCCAGGTTTTTCCGCAGCGGGAAAAATCT
CGCAGACCATGTGACCGCCGGTACTCCTTTTCCTGTTGCTCGGCCATTTGATCCTCGCTTCGAGAAAAAC
TATAACAATTGATTAGTGCGGAGAGGAGGATAGGCGGTAAGATCAGTTTTGTTTATTGTATAAATGAGAT
ACTTTGGAATAAGGACATTA

>Negative Sample 32
CACAATGATGAAGGTTCTTTGTTAAGTGCATTCCCCCCCTTTTAAGTACCTTGGCTATCAATTTTATACC
CATTTTTCGTATTTACCATTTAATTAACGATCCAATTATTAGCGTTAATTACGGAAGCGCCAATGAATTT
TTAGAAATCAGAACCATAGTTTTGAGCTCAACGGAACTATCCAGGATAATTTCTCAAAGCTTTATCAAGA
TGAGCATAATTTTCCTTTCGGGGTGTAGACCTTATAAGAGCTTCCGCGTTTTTGTTCTACATTCCATTGA
GAAATTTGTTGCCAAAGTTT

>Negative Sample 33
TTTACTCTTCTTCTTTAACTTATGACTGCTCCTCTACCGTAACTCAGATAACCATGATTCCTAACTTTGA
CGCGTTTGCCGTTTCCAGTAAAGATGGACAAATAATTGTATTAAAGGTTAATCATTACCAACAAGAAAGT
GAAGTCAAATTTTTGAATTGCGAATGCATCAGGAAAATTAACTTGAAGAATTTTGGTAAAAATGAATACG
CAGTGAGAATGAGAGCATTTGTGAATGAGGAAAAATCTCTACTAGTAGCATTGACGAATTTGTCAAGGGT
TATTATATTTGATATTAGAA

>Negative Sample 34
TGACAAAACTTTTGAAATTATTCCATTCCAGAGAGCCAAAGCTACTTTTGAACTCTCTTGCAGAGGGGAC
ATTATCCAGTTTGATTTGAAAATCTGACATTTCTCTAAATTTTATAGTGTTATCTACTATGGAAACAGGA
GTGACTCTCAATTTATTCGAATCTTTTAAGGATTTGATAGAATCTTTGACGCTATTATTATTTTTACTTA

AAGTTCCCTTAGGCATTGTAACCTTTAATATGACCGACTCATCTCTGAAGGGAACTCTTTTACCAATTAC
AGGATGCTCATTAAAATAGC

>Negative Sample 35
TTTTCCTGGAATTGTTTTTTGAAGCTTTTCCTCATTTACGCTCCAACGATTTCCACATTGCAGGCGAATC
CTATGCAGGACATTATATCCCTCAAATTGCACATGAGATCGTTGTCAAGAACCCTGAAAGAACGTTCAAT
TTAACTTCAGTTATGATTGGTAATGGTATCACAGACCCTTTGATTCAAGCAGATTATTATGAACCAATGG
CATGCGGGAAAGGGGGCTATCACCCTGTTCTCTCATCAGAAGAATGTGAGAAAATGAGTAAAGCTGCAGG
TCGTTGTCGTAGGTTGAACA

>Negative Sample 36
TGTTTCAGACACCAAACACATTTACATCTCAGCCTAATATAACAGTAGAAAATGATTTTCACAAAGGTGG
GAGACATGTGATTGACTATCTGAACAAAAAATTGGCTACTATGAACATTGATATTGATTTGACTTCAGGG
GGGAAACAAAACGTATCTTGGGAGGAAGAGCTCGACCAATTGAGCGACCACGTTATAGAAAGTATTACTA
ACCACATTTCAAAGGGTAGAATGCACGCACAAGAGAAGCAAGATGAGTTGGAAAAGTTAAAATTGGAGAA
TTTGAACTTGTCAACATTAA

>Negative Sample 37
CTCCTGTTCTCCAGTTACTATGCGTTCAGCGTCACGTTTTCGTATTACCTTGAACATGACTACCGTTTTA
CTATGTTAGAAATTGGTGCTGCTTATGTCTGCCCAGGTGTAGCTATGTTACTAGGATCTCAATCTGGTGG
GCACCTCTCAGATTATCTTCGTTCACGCTGGATCAAAAGTCATCCTAAAAAGAAATTCCCGGCAGAGTTT
CGTTTATTACTGAACCTAATTGGAATTTTACTAACAATATGTGGCACAATAGGATACGGATGGGCAATCT
TCTTTCATTATCATTTTGTG

>Negative Sample 38
ATGTCGTTCACACCGAGCATATGACCACCATGGGTAAATATAGTTTCTTCGCAGGAGGTATCCCATAGCT
TGACTAACCCATCTTCTTGGCCAGCCGTTGCCAATACAGTATCGAAATTTGGACTCCATTCTAAAGTTGA
AACGGAAGTACCGTGCTCCATGGTAGCGATTGGGCTTTTGTTCATGTTTCTAATATCCCATAAATTTAGC
CTCCCATTTGAATCTGCAGATGCTAGAATTAAAGAGTTCTTATAGTTAAATCTACAGGAGTTTACTCCAC
CATCGTGTTTTTCACGGTTA

>Negative Sample 39
TGAATAATGGTTTCGACAGAGAATTTGAAAGAGACGAAAATCAAAACAATATTAAAACATTACAAGAAAA
CGATACAGCAACCTTTGCAACAGCATCTCAAACTTCTAATTTTGCCTCAACTAACCAAGACAACACTTTA
ACTGGCGAAGACTTGAAGTTAAACAAGAAACTCTCAGATTTATCTTTATCGGGCTCCAAGCAGGCTCCAG
CCCAATCAACTTCTGGATTGAAAACTACTAAAATTAAATTTTATTACAAAGACGATATTTTTGCTTTAAT
GCTGAAGGGTGACACAACCT

>Negative Sample 40
GAAAACAACTCTTATGAAAGACACTTATTCAGTTTCCGGGACTCTGGCTTGGATAGAAATTTTTCCTGGG
CTTGTAATGCCAAATTGCATGAATTTGTCGCATTGATGGTTATGGATCATCTTTCAAATTTAGATGATTT
AGGATTAGAAGAGCTTTTTAATATCAAGTCCGAAAAGCTATATATAAAAAACTATTGTTGCCGAGACCCG
GATATTCTCGAAATATCAAACTTATTTGATATTAGATATGGGGCCGGATCTGAGGCTGATGCTACTTCTG
ATTCCAATCTCGAAGCAGAA

>Negative Sample 41
CATTTTACGTTGGATCAACTCCTAACCCAGTACGACGTTTGAGGCAGCATAATGGGAAATTGGCTGTTGG
AGGGGCATATCGAACCAAACGTGACGGTTCAAGGCCCTGGGAAATGATAATGATTGTGCGGGGCTTCCCA
AGTAAGATAGCAGCTTTACAGTTTGAACATGCATGGCAACATGGGTACCAAACGCATTATATTGCCGAAA
AAGATCGTGTTGTAAAGCATAAAGCAGGTGGAAGAACTTTACATCACAAAGTTGCATTAATGAAGTTACT
TTTAAAGCATGAATTTTTTC

>Negative Sample 42
CGCCCATGCTCACGTACTTTCTTCCGTATCGGTTGGCCCAGTTACCGGCGTAGTACGACCCAAACAGTCC
GCCTATGCTGAATATAGACGTAATTGCGCCGTATTGAGAATCGGTTAACGCAATGCATTGCTTGAGTCCG
TGTTGCCCAACCCAAGTGTCATCGTATGAGATGTTTTCGTCTGGTGCCTCAAATCTGGAGCACGATAAGA
ATTCTTGAGGCGCATTCAACTCTGCGATGTGGTATCCGTACTGGATCGACCCCAAACAAGCAACTATCGT
GCCCAAGATTAAATGGCCCG

>Negative Sample 43
CCAGGTTTACTCAAGTCCTTACCGAATAGGCCTATTTTGATGAAGGATTTGCCCACACGTGGAATCAACA
TATCTGTAGCTAAATAGCCTGCTATACCAAATCCCACGGCCGCAACAAGAGCAGATGGGCCCTGATTTTT
GGAATAGTAGATTAAGCATGTGATAAGTGCCAGTGAAAAAAGTCGCAACATATAGTCTACTTTTTGTTAC
CGTTCTCCGCCTTGCTCTACTTGATAAAGTTTTTATTTACATATTTCACAGTTATATACACATGTTAACA
ATTTAATTTTCGCGCTGTTT

>Negative Sample 44
TGAAATTGGTATGCCTTTCCAAATGATATTATCAATTACCAAAACAGATTCTTCCATTGAATTACAGGAA
GCTTCTTTGGCAGTTGCACAGAGAATGGCAATACCAAGTATTGATTTGAAGACCAAGAAAATACTACGAG
AGCCATACATTAAAAAGTCCGAGTATTTACTAAGAACTGTAGAAAGCCAAAGTTTCGATTCCGACAAGAC
CATTTTCGGATTTTGCTTTGATGACGTTGTCATCCCAACCTATGCGGATGGTCTACCGAGCTGGTTTAAA
ACATTCTACTGTGAACCAAG

>Negative Sample 45
CTCGATAAGAGAAGTGCCAAGTTCGTCAATAACCAACCACACTACAGAGACCAACTTCTGCAGTAATTCT
TTTTGCACTACTTTTTTTAGTATTTTGTCGCATATATACTGCTTCACCATTTGTACATTAGATAATTCTT
CACTACCCCCTCTATAGCAATGTTATAGTCATTGAATCATGCTTTGAAATGCCTGTTCTAATATTCTCTT
CATATAATAAATGCGTGATGCCGGGTATTTGTGCGCCCTTGTTTGGCGCAAAAATCTCGAAAGTGAAAAA
ACTAATAGGCTTCTTAAAGC

>Negative Sample 46
TGTATCTTCTGGATATAAATTCTTGTTAATAGCTGATAAATCAAATTGTTTATTCTCGCAATCACTTGAA
GACGCTAGCTTTCGCCTCTTTGGCGTTGGCGATAGATCGCTTTGTATATGCATATGAGCTTGTGGAGATG
GAGGTGGCAAGTTAGTTTTCATCCACGGAGAGCTTCCATTGGAATAATTATCGTGTTGAGACCTACGGAG
GATATGCTGATCAATTCGATCTATCGTATGTTTCTTTTTATCTGAAAAATCTTTCGACATTGCAAATTGA
CCACAAAAACGATTTTGCAA

>Negative Sample 47
TAGCGGCTAGTATATACCGTTTACATTCAGCAGATTTATGCGAACTTCTAAGAAACAGTGTCATAACCAG
TGGATTTTCAAGTACCCTAAAGAACAAATGGCTCGGCGTTTCCTTAGCCAGCCATGACTACTTCGTGGAA
AACACTGGGTTTGTAGACAAGTGGTATGATTGTAAACCAAATACAAGTTTAGAGCATAACGTTCCGATCA
TCAGACGTCAATATCGCAGTAGCACTCTCGCAGGGGAGTGGCGACTCATAATTGCGTGAATTCGCTCATG
TTTCTTCGAGGGGCGCATAT

>Negative Sample 48
CCATGGATCAAAAGAAGTCTTTTGAGTGTTAAATTCGGAACTACAAATGAGGAATATAGTGACGACTCTA
TTATCGCTGGCCATTCTAGTTTTCACGTTGCAAAAGTTTTGAAGGATGATGTGGATTATACTGATGAAGA
CAGCCGTATAAGCACATCTTACAGTAACTATGAAACATTATGTGCTGCAACTGGGACAAATTTACCGTTT
TTTCATATCGATATACCCGATTTTTCTAAATGGGACGTTTAGTGTAGTAGATAGTAAGTACTTTAATTAC
CCCCCCTGTTTTTAGTTATA

>Negative Sample 49
AGGTGTTTCTGCTCAAAATGTTTCTAATACAAAAGCTGTGACTGGTAAGCGGGTAGAAGGAACATCATAC
TCTGGTTTGAAGTTACAAGGAGGGAACTGTCGTTGGCTTGGTCATAACAATGATCCTGACGTTCGAAAAG
CCCTTGAACAAGGATATTCTGTATTCTGTTTTAGTGTTAATGGTTCAGTCACTGCAGTTTATGCATTAGA
GGACTCTTTACGGGCAGATGCTGTCTCCACTATTAACTTGTTACGCCAAAGAGGGATTTCACTACACATT
TTATCAGGGGATGACGATGG

>Negative Sample 50
TTTGATGCTTTGGCTACGGAGTCTACTAGCGGTTCTTGCACTGAAGACTCACTGAAAAAAATATGGAAAG
AGCTTCACACAGCATCCCTTGAAATAGAACCATGGTCTTATGGCTATGTGGACATTTCATTTTCTCGACA
TTGGATTAGGGCGCTGGCTTGGAAGCTAGTGTTTCAGATGAATGGTACCAAGTTTTTCTCAAACGCCAAT
AATGCTCACATATTGGTCGAAATTGCAAAGGATATGCTGGACGACATATTCTTAACTCCAAACAACCTGT
ATGATGTACATGGTCCTGGA

>Negative Sample 51
CACCCACACACCACACCCACACACACCACACCCACACACACCCACACCACACCCACACACCACACCCACA
CACCCACACCCACACACACCACACCCACACCACACCCACACCCACACACCCACACCCTAACACTACCCTA
ACACTACCCTATTCTAACCCTGATTTACCTGTCTCCCAACTTACTCTCCATTACCCTACCTCTCCACTC

GTTACCCTGTCTCATTCAACCGTACCACTCCCAACCACCATCCATCTCTCTACTTACTACCACCAACCCA
CCGTCCACCATAACCGTTAC

>Negative Sample 52
AATATTTGGAGGCGTTCTTGGGGTAATTGGAGCAGGGCTTATGACACTTATGACAAATACGTCCACGAAG
TCAACTCAAATTGGTGTTTTGCTATTACCGGGGTTTTCCCTTGGATTTGCTCTACAAGCATCGCTCATGA
GTGCACAGCTTCAAATTACCAAAGATCGTCCAGAAGCTGCTATGGACTTTATTGAAGTAACAGCTTTCAA
TACATTCATGAAGTCATTAGGTACAACTCTTGGTGGTGTGCTTTCAACCACTGTTTTTCCGCCTCCTTT
CACAACAAAGTATCACGAGC

>Negative Sample 53
CCAAAGATCGTCCAGAAGCTGCTATGGACTTTATTGAAGTAACAGCTTTCAATACATTCATGAAGTCATT
AGGTACAACTCTTGGTGGTGTGCTTTCAACCACTGTTTTTCCGCCTCCTTTCACAACAAAGTATCACGA
GCTCATCTAGAGCCTTACGAAGGAAAAACGGTTGATGACATGATTTTGTATCGTCTTCAAAACTACGACG
GTTCTCATTCGACTATTGGAAACATTTTAAGCGACTCCATTAAGAACGTATTTGGATGGATCTAGGGTT
TTATGCCTTAGGATTTTTGT

>Negative Sample 54
TTTGTATAAAATATGATATTACTCAGACTCAAGCAAACAATCAAAGAAATCTTTCACTGCTCTTTTCTGT
GTTCCATTTAGTTTTTAGTACGATTGCATTGTCTATATACTGTATTTACCAAATCTTAATTTTAGTCAAA
CATTAATGTTTACTTTCGTATTTAAGGATGTGTATACTAAGCCTTGGGAAAATGCGTGTTCCTTGTGCCA
TCCGAAAGCTACTTCATAAAAAAAGATACTGTTCATTTTCAAGCCCGCATAGTTGGATCAAATATTAACTA
TATATCAACATGGTCCAAAG

>Negative Sample 55
AAAGATCGTAAGTTTGGTCTAATGTTGTTTTAGCAAAAAAAAAATACTATACCTTTCATTAACGTTTATTG
AATTTCTTGAAAAAAAAAACCCTAAATTATTATAAAATTATAGCAGGATAGTTCTTAGTGGAAGATATTAA
GCAAACTATTTTCTGAATTGCTATGCGAAATTTCTCTAATGCCAGCTGAGTAACTAACTCTCATGGTACA
TAAAAATACGCATCTTAAAAAATTTGATATTTCATGATTTATTTATAGAAATAACGATGCTTATTGTGCT
TTGTTGGGTGTTTGACTTCT

>Negative Sample 56
ATCCTCTTTTTCACCCGATTATTTTCATTTATTAGTAAGTGGGGTTCTGGGTGAAAAAATCAAAACAAAA
ATATGAATTTGTAATGATTTTTATATTTTCGCCGGAGGTGCTGGAAATGGCAAACGAAAATACTATGACA
TAAAAGCTGGGCACACTATGTACGTTCTTTTTAATTTTTTATCAACATGAGAAAAATTCATGAACACTGT
ACCTTATATTATCTAATAAATGAAGTAAGCTTTGCATCTCCGTAACAGCTGCATATATATAATTAAGCGG
GAGCTTTCTTTCGATCTCTT

>Negative Sample 57
TTAAGGACTCTTTGATGGTGAACGAATTACTACTTTGCAGCAGGTAATTCTCTAAAACTCAGAAACAAGG
CATGTATTTTTCACTAGGAAAAAGAAGGAAAATGGGACTACAGTATTAGGGAAACAGGATTAAAATTCGT
AGGTAATAATAACCCATACTTTCATTGTAGCAGTACTTTCGTTAATAAAATGGATATTGTTGTACTTCGG
TAGATGTTATATTCTAAAGAGTTAAATTATCCAATTCCAAATTCTAGGGACGGTTTCCAAAGATTTGTAT
TGTCCAGTGACATAATTTTC

>Negative Sample 58
CACCAAATTCTGGGATAACCGTTAATTCTTCCTCAGGTTTGCCTAGTGGATCCTCTCCTTCTGGAGTTTG
GCCACGCTCTGGCTTTTCGATCAGACTTGGCATGTGACTAATCAAGTATGGCATGCTGGTTTTTGGGTCC
TTTGTTTTCGTTGTTTCAGTCTGGATAAATTTTAAGTTACCATTATCGAAGGCACTTTTGTACTTGTCAC
TAATTAAAGATGCAATGTCAGCGGGGATACTCATTTTTATTTTAATTTTTACTTTTCTGTTTGTTCTAAA
ATCTATCTAAACTGGCTTTC

>Negative Sample 59
TTTCTTTAATGATGGTTTGGACGACGGATTGGATGTCGTTGATAGTAATTTCACCAGGTAACTCCGGTTT
CAAAGCGTAAATAGTACGACGAGCAGTTAAAGTTTTCAAATAAGTTGCAACAGCAGACATGATATTGGAT
TGTTTTTTTGTATATATGTTGATATAGGATACTTCAGTCTACGAAAAAAGTACAAATTATGTAGTCAGTT
CCTTCAGTATGGTGTCCTTATATACTGTAGTTTGGACAAGGTGCAAATGCCAAGACCCTAGCCCGAAAAG
CTCGAGGCACCCCAGGATCT

>Negative Sample 60
GCTGAGCCGTTTAGTTGCACAAACAAGCACATTAGACTTCTATGTGGTTTTGACGTTGTTTCAATATTTT
GCTGTGCTTTGTGCTTTTGGCAGCATCATAGGACTCATCTTTGGATTTATATTGGGTGTGTTCCACTCAA
TCTGCGGGGTACCCAGTGTATACATAAGTCTAGAATGGAAACGGTGGTTTGCTCCGATACGTACGGTCCT
TGAACGTGCTTCCACTAGTATTGTCAACATTATGCGAGGACAAACTATTGCGCCAATACCCATGCCTAAG
CCCAATCCCACGCATATATC

>Negative Sample 61
AATACTCTATGATAGTGCATAACACTAAAAAGTTGAAAGAATCCCGTATCATGGACGATTTCCTTGAACA
TTTGAGCAAAGACGATAACAAAGCATGGTATGGCGCGGAAGAAACCGAGAGAGCTGCAAAATTAGATGCA
ATAGAAACACTACTTATTACAGATAGTGTACTAAAAAGGAACGACGTGAAAAAACGTGAAAAATACCTAG
ACCTAATAGAGAATAGTGGAAACAACAATGGCAAAATATTCGTACTCAGTACTTCAAAAATCACAGTGAG
CAACTTGACAAACCAACAGA

>Negative Sample 62
ACCTTTGGCTTCCTTTTGTAATGCCTCACCTGCTCTTTGAAGAACTTTATAACGACGTTCTTCCATGTCA
TACTCTTTATCAATGGTCTTGTCGACATTCTTAATTATCACACTGTGACCAGCTCTGTTGATAGCTTTCT
TAAAACCTTCCCAACTCATCTTGTATCAGCTTTCTTCTGGCTTCTTATAATAGGATTTGTATAGTTTTCT
ATATCACAGCGTTTCCTTTTCTTCTCTCACGTCTGCCTGTTATTAAATTTGTTTTCCCTTTCTTTTAATT
CGGATTCTCATAATTGTTCA

>Negative Sample 63
CGATAGCACTCTCGTGGAGTGATGGGGTTTTGCTATGTTAATCATCAGAATTAAAAAAATGGCAAACACC
AGCGAGCCAAACAATGTGCGAAATATTATTTTAAAAGGCACTTTGGAATGAAATCCCGTTCCAAAAGAAA
GTTCATTCTCATCAACAAGTTCAGTATCAATGGGTTGACCATTCGCGTCTAATTCATATAGCGGTATACC
CCTTCTAAAGTTAGAACCATCCCTACTATCTTTCGCGTCGCCAATCACTATGGGATTGTTTGTCCACTTC
AGTCTATTCTTATAAAACCA

>Negative Sample 64
CTATTTATGACCCGAAACAGTTGTGTGAAATTTTTCCAGAGGGCAAGGAGGAAGAAGAGTATGTTGTTTT
TCAACACGAAATTCCTGATCCTCTTTCCATTATTAAATGGATACATGCGAACAGGCCGAATTTTCAGATC
GTATATAACCCCTCACCTTTCAAGGCCATGCCTAAGAAAGATTGGGAGTTGGTAGACCTTTTGGTCGTTA
ATGAAATTGAGGGTCTTCAAATCGTGGAAAGTGTATTTGATAATGAACTTGTTGAAGAAATAAGGGAGAA
GATAAAGGACGACTTTTTAG

>Negative Sample 65
ATTTCTTACGTATAAAATTCTTATTGAAAGAGGTGGTTTTCCCTTGAGGAGATAACTCGTCTAACTCAAT
TGTTTCCTCGTTAATATTGTTCCCATCTATGGCCTTTTCGTCGGAGTCTCCCCTTTTGCTTCCTGAAGGT
GTTTTAGAACCAAAGAGCTTACGCTTGAGTTTATGTGTAAATCTTCTTTTCGATGAAGATGGTTCTCCTG
CAGCTATGTTGCTTTCTATATCAGCGATTGGACCTGGAGTCTGAGAGTCAGCGTCATCGTTTAAGTCACC
GGTTGGCGTTTCTTGTTTCA

>Negative Sample 66
TCATCTTGAACTTGCCTTATACCCAGTTCTATAACCATCCTCTTCCTATTAAATCTTTGGGTGACACGTA
ATATGGGTACCCCAGACCCATATACCCATTCGTTGAAAAAATTCTCTAATTTACTTTTATTAACTCTTTC
GCAAACATGTTGAAAATGCGATGAAGTCAACGAATTATTCGGTAAATCACCAGACATAGCTTGAAGGAAA
ATTTTAGGTAATACTCGAGACATACCGAAAGATCGTTCTGTTTTAGTCATCCTTCTGTCAAGTATGTGTA
GTATCATCGGTGCCTTCAAT

>Negative Sample 67
CGTGATCGTCATCCATTGAGCTTTTCTTTCCTCTCTCTTTTTTTTTTCTTGTTACATATTCCTATATATA
TGTATATATATCTATATATATATATATCCCAGCGTATATGACTGCACAAGACGCAATTTTCTTGAACGGT
TTTTTCTTTTCAGCACTGCGAAAGAGAGAGCAACGGTAAAGAGCATGAGCCAATTTGAAAAGCAGAAGGA
ACAGGGCAATTCTTTGTTCAAACAGGGCCTGTATCGCGAGGCTGTGCACTGTTATGACCAACTAATTACT
GCTCAACCGCAGAACCCGGT

>Negative Sample 68
CAACTTCATTTAATTTCCCGTATATATTATATGTACGTATATATGCTATAAACAGACACTCTTTTTACTT
ACCTTTAATAATGCTTGATGAGAAATCAGTCATTGTTACTTCTTCCCCAAGGTTGTCATCATAATCATAC
CATTCATTATCCTCTAGTTGGCATGACATTACTTTCCCCGAACTTAGAGAAACTTCAAACGTTATGGTGT

CGGGATAAAATTTGATTAATTCACAACCTCTGCAATCAAGGCTTAAAGGAATGAAAGAATCTTCTTTGAC
CTTCGATAAACCATGCTTCT

>Negative Sample 69
TGATAACCCAAAAGCATAATACGAGTAATGTTTCAGTATTGCTATTATATGTTTACACAAGGAAAACATA
TAATAACAAACCTCTAATCCGGTAGTACTTAAGAAACTATAGTTTCTATGTACAAAAAGGTAACTATGTA
ATTCTTACATTTACATAACATATAGAAGGGTCCAATAAACTTACTAAACTTACTACCTTGTTGTATATAG
GCTAGATCGTAATCCACTACGTCAACATAAAAAAAACTTAAGGAGTTTGAATTTTATGTACAAACAGATT
GTTAAAATATAATATAAGAT

>Negative Sample 70
TCCTTATATCAAAGAAAATCAAGAAGGACAACATGGATGATATTTGTAGTATGGCGGAAAACATAAACAG
AACTCTGTTTAACATTCTAGGTACTGAGATTGATGAAATCAATCTCAATACTAATAATCTTTATAATGTA
TGTTTTCATTTCAAGGATAGCCTTTGAATCAATTTACTAACAATACTTCAGTTTATAATGGAAAGTAATT
TGACTAAAGTAGAGCAACATACATTACACAAAAATATTTCTAACAATAGGTTAGAAATATACCACCACAT
TAAAAAAGAGAAGAGCCCAA

>Negative Sample 71
TACTGCTATTGCTATTGCCTATGTTCGCTCTAAGGCGAAAAATTTTTTTCCGTCGGTTGAAGGAGGAAAA
ATTGAGGATCTCAAAGAGAACATTAAGGCTCTCAGTATCGATCTAACGCCAGACAATATAAAATACTTAG
AAAGTATAGTTCCTTTTGACATCGGATTTCCTAATAATTTTATCGTGTTAAATTCCTTGACTCAAAAATA
TGGTACGAATAATGTTTAGATAATTTTTCAGTAATCAACTACGCAAGTAAAGCAGTAAATACGTTACTGC
TGGTATTAATGTCATGTATT

>Negative Sample 72
CAAGTACCTTTTCAATATAAATAATTTTCAGTTAATTAACAGAGTAGGAAGAGGAAGGAACAAAAACTAT
CGCTTATCTTCTTGCATAGGGGTAGTGGAGTAAAGATACAAGTATTCGAGAAATAGATATACATTAAAAT
AGCATTACTATCCCGGGAATCTTGTTGCCATAAGGTGCTTTTATGATATCAAACCAGGGGGGAGAATATT
TGGGAAGTAATTGGGAAGTAATTGTATGTATTTCTAAAATTTTTGGATACTCAACTTTTAAATTCAAAAT
AGTTATATTTGTTATCGAAA

>Negative Sample 73
AAGTGCGGTAGCAAGGGCTATATTAATCAAACCTATACGCCGACAGAAATTTATAATTGTGGGGTAGCTG
AAGGAAAGAAAACTGCTAAAGAAAAGAACCCAACTTATTCAATTTTTTATGATACATTTCTCACTGGGCA
GCCTGCTGAAAGTCCTGAAACGTTTACGTGTGGTTCGCATGGCTTCACAAATGCGAGTTATGTGGCAAGC
GACTTTTATGCATGTGGTTTTCTGCAAGGGAAAGGTACAGAAACCAATGCGGGAATACATAATACAAGGC
CTTCTCATTCTCTCGCCAAA

>Negative Sample 74
ACTATCTCTAACGCTGTTGGTAGATTGTCTTCTGAAGAAATTGAAAAGATGGTTAACCAAGCTGAAGAGT
TCAAGGCTGCCGATGAAGCTTTTGCCAAGAAGCACGAAGCTAGACAAAGATTGGAATCCTACGTTGCCTC
CATCGAACAAACTGTCACTGACCCAGTCTTGTCTTCTAAATTGAAGAGAGGTTCCAAGTCCAAGATTGAA
GCTGCTTTGTCCGATGCTTTGGCTGCTTTGCAAATCGAAGACCCATCTGCTGATGAATTGAGAAAGGCTG
AAGTTGGTTTGAAGAGAGTT

>Negative Sample 75
TCGTTAGGAAAAAAATGGGCCATTGCATTTATGTCATTGATCGCATTCTGTCAATTTCTAATGGGTGCTT
CCATTACCCACAGCTGTTTCTAGACAAGTTTGGGCATTTTCCCGTGATAACGGTTTGCCCCTATCAAAGTA
TATTAAAAGAGTGGATTCTAAATACTCGGTCCCTTTTTTCGCTATTTTGGCTGCCTGTGTAGGTTCCTTG
ATTTTAGGATTGTTATGTTTGATTGATGATGCCGCCACTGATGCATTATTTAGTCTGGCTGTTGCAGGAA
ACAATTTGGCATGGAGTACC

>Negative Sample 76
CCAGGATATGGATTGGTCCAAATTGTACCCTTACTACAAAAACGCGGAAAATGGACAAATGACAAAGAAG
GTGACGATTGCTGATATTGGCTGTGGATTCGGTGGGTTGATGATAGATTTATCACCAGCCTTCCCTGAAG
ATCTTATCTTAGGGATGGAAATTCGTGTGCAGGTTACAAATTACGTGGAGGATAGAATTATTGCCTTAAG
GAACAATACAGCTTCAAAACATGGATTTCAAAACATTAATGTCTTGAGAGGTAACGCTATGAAATTCTTG
CCCAACTTTTTTGAAAAGGG

>Negative Sample 77
AAGAAGAGGAACAAACAATTACAAAGATGCCTATAGAGGCCAAAGTCGTCGGAATAAAGAAAACGGCGGT
TACCAATCGGGATATTCTTCGCCATATCTTGTGTATCCTCCTCCCCAAATGGGTGGCAATAGTCTGCCAA
CCTACCCTCTAATGTATAACCCCGCTGGTCCTGCCCCCGGTCCTGCACCTTCACCCATGGTAATGGGCAA
TAACACAGTCTTCATGAACCCTTACATGTACAATATGAATCCCCAAGGGTCCTGTTCGTTTGGCACCCCA
ATCCCTATGTATCCCCCATA

>Negative Sample 78
GAAAATACACAAAATACTACTAAAAGCTTCACACCGATAAAGAGATTTATTGCATTTAGCACGGCATACC
AGTTGGTCACCGGTTTATTGTTAATGATATTGGTAGTTGGTAGTAATATCATGTTGACAATTGGGGAATC
ATTGATTCTGTCGATGTATTTCGTCTATGTTCGCGGCCATGAAGGATTATTTACTCCCGTGGTGAACTAC
TTCGGCTCAAGAACCATTTCAAATTTCATCTTATGCGTAATAATTCCTTTCATATCTCTAAACTTCCTCA
TCAACACTTCGATATATTTA

>Negative Sample 79
TTCAATTCCGTTGTTAAGTACTGGCTGATTGTGAATTTTATATATCATGATATTTTGAATTTCAATACAA
CGTCCTTCCCGATCGAACAATACGAGAAATTCTTCCAAAGGGATCAGAACTCTTTACCCAGCTCAGCCAA
CTTTATTGAGTCAATAGACTCGCCAATTGAGGAAATAGACCCTTTAATTGGCATCAACAAGCCTATTTTA
CTACTATTGGGACAAGTCACAAATTTGACGAGGTTTTTGCAGACTATGGAACAGGAAGAAATGCTAGAGC
ATGGCGATAAAATTTTGAGT

>Negative Sample 80
TATATACACATGACCATTTCTTTCAGCAATTGGCCGTGAAATTCCATCGTCCTTTTTGGTCTTCCGTTTT
TCGATATTTGGCGGATATACTCGATGAACAGGATTGCAATCGCTATGTTTTCGTATTTTGGACTCACTCT
TCTTGCCGGACTCTTTCAATAGTCCTTTTAAGGCACCATGGAGATTTTTCAGGCTTCTTCTTTTAGAAAT
TTTCTTATTGGTTTTCATCCTTCTGTTAATTTGTAGTGGAGGCGTCGATATGCTGTATCCCACTTTTTGT
TTATATATCATCTGATCCAT

>Negative Sample 81
AGACCACTTCTTGAACGACCTTTAAAGCGTAAACTGTCATTGCCAGGACTAGCGCAAAGACCATTATCCA
CTGGCGCTAGGCTAGAAGGAGGATATGGAGGTGTATCGCCGAACTCATGGAAAACCAAAGTTCCAAAACT
TCCTCTTCCTGCTTCCCGGCCGTCTTTGAATCTTTCTCCCCAAAAAGTCCCAACGGGTACTGATAAAGTT
GAGGAAGATACCAAAATTGACACATTGGAATTAGTAGAGAACAATAAACCGCATCCAAGGATGAGAAGAA
GGAGTGACAATCCAGCAACA

>Negative Sample 82
CTATTAGAATATTTTGATTGGGACGTCACCATTTCCACAGATGATTTAATTACATGTCTCTCGCCATTCT
TGAAGCCTATCAAAGAGGAGCAGTTGTACAAATCGCAAAGAGACTGTCGCACGTTGAAAAATTTTTCTGC
TCAAGAAAAAGACATAGTAAACAAAACATCCATTTCCCATTCGCGCTCATCGTCCAATATGTCTATCCCA
TCTTTAGCTTCCACTTCGACCCTCTCCACGTTGGAATCTAGAAGATCGAATTTATCCAATTATAGTAACA
GAATACGAACTTTGCCCGAA

>Negative Sample 83
GCGTAAAGGCTCTCGAACAATTTATGGAGAGAAATAATATTGGAGAAATATGCAAGAATTATGAATTAGA
GAAAATAGCCGATAACATATCAAAGGATGAAAATGAAGATCAGTTTTTGGAAGAAATAACTCAATATGAG
CATTTGATAAAGGGCATAAGAGAATATGAAGAATGGCAAAAATCCGTTAGCTTGTTAAGCTCTGAATCGA
ACATTCCTACTTTAATAGAGAAATTGCAAGGTTTTTCAAAGGATACATTTGAATTGATCAAGACATTTTT
AGTAGATTTAACTTCCTCTA

>Negative Sample 84
AACAAATCATCTCTAGTTAAACCGGGCTTTCTGGGAACACCAGCCGGTATTAGCACCACCTGAGCATTTG
ACAAGGTGTTCTCAATACTATCCTTATCATAACCGACACAACTTGAGTTGGTGTTGATGTGAGATAAATC
CTTACCAATGCCTTCCGCAGCTCGGATATCGTACAACGCCAGCTCGGAAACGTAAGGGCTTAATTTTAGC
AGTAATGATAGCGGTTGTCCCACGCCACCAGAAGCGCCAAGAATTGCGACTTTGACCATGTTTATGATTG
TTTGTCTCTTTTTATTTTCT

>Negative Sample 85
AGTATCAAGCTCAATAGGAGGGAGAATATATTGATAGGTCCACCAGAGTTGAGATTTTCTAATGAAATCA
AGGAACACAATAGTATTGCTACAATATTCAAACTGGTATTTATTCAAACCAATAGAAGAGTCGTCGAAAA
AATAGATTTGGCGAACATGAAATGGGGGACAATCCACGGAGGCAGTAAGTACATGAAGCCTTTACCCGTC

CCGAAAGATTTGGTTGCGAGTGTTGCTAAGAACGAAAGTGAGACGAGAAACTTAGCTACCTTATGCGGTA
ATGGTCTAGATTTTGAGATA

>Negative Sample 86
AATGGAAACCATAAGGAAATCAGATGAAAAATTAGAACAGTCGAAAAAAAGTGCTGAGGAGGATATTAAA
AACTTGCAACATGAGAAGTCAGATTTGATATCCCGAATCAACGAATCTGAAAAGGATATTGAAGAACTGA
AAAGTAAACTGAGGATCGAAGCAAAATCTGGCTCTGAACTAGAAACCGTAAAACAAGAGCTAAATAACGC
ACAAGAAAAAATAAGGATCAACGCAGAAGAAAATACAGTTTTGAAATCTAAATTAGAAGATATAGAACGT
GAACTCAAAGACAAGCAAGC

>Negative Sample 87
AGTAGCTTTTCCCCTTATAGATGTACGCCGTTCGATATTACCCGAACTGCAAGAAAAGCTTCTCCAAATA
TCTACTGGCCTCCATTCAAGAAGGCAATTAAAGAGACTATGTGTACATGAATGGGAACTTGGTATGATGG
TAGTAATGCTTTATTAGTCATTCCCCTACCCCCTTGCGCAACCTATCATTTTACTTGCCAGATGTGCTCT
CCACTGTTAGAGCCTCTTCGCTGACAAAGAATGGACGAGTACGCGAGCGGTGAAACGAGCACTGCAGGCA
AGAGAAAGAAAAATCATGCA

>Negative Sample 88
CCATTGTTGCTAAAGGCTATAATATTGGTATACAGAATATACTAGAAGTTCTCCTCGAGGATATAGGAAT
CCACAAAAGGGAATCGATAGTTCTACATAATGTTATTATTTTATCTTCTTTCCTTTTATGCGTTGTCATT
CATTATCCTATTACATTATCAATCCTTGCATTTCAGCTTCCATTAGATCGGATGACTGTTTCTCAATCTT
TATGTCATCTTCTTACACCGCATGTGATAATATAATATTAAATAGATGATATTAGAGTTCAATTCCAACA
ATTTTCATTCTAGATAGCGG

>Negative Sample 89
CTTGGAGTATGTCTGTTATTAATTTCACAGGTAGTTCTGGTCCATTGGTGAAAGTTTGCGGCTTGCAGAG
CACAGAGGCCGCAGAATGTGCTCTAGATTCCGATGCTGACTTGCTGGGTATTATATGTGTGCCCAATAGA
AAGAGAACAATTGACCCGGTTATTGCAAGGAAAATTTCAAGTCTTGTAAAAGCATATAAAAATAGTTAAG
GCACTCCGAAATACTTGGTTGGCGTGTTTCGTAATCAACCTAAGGAGGATGTTTTGGCTCTGGTCAATGA
TTACGGCATTGATATCGTCC

>Negative Sample 90
TTGTCTTGGAATAACAACTCTTATAATTTGTAAGGGGCCCCTTGCACCTCGCGTTTAAGTCCATAGAAAC
CAAACTATTTGTTAAAGCTTTCTCATCCTTTTGCCAGTTCCTACTTAAAAATATAAACTTAAAATTTCGC
ATGCCAAATCCCAGTAATGGTATGTACTGCAGAGCTTTCTTCAGGATGATATAAACGTTACCACCCAAAT
TTGAAACAAAGGAAAGCCACCAGAGATAAATCCAGTCTGCATACATTTGATGATTTGCAATTATTATAGC
CCTGTCTTTAAATCTAAAGC

>Negative Sample 91
GAATATATTATTCGACGATATTTAATTATATGAGAAAAACAGTAAAAAGAATAAGGCCAAATGCATTGAT
CGTTTTGCCGCTCGTTGACGTTTATATATATTTTATTGTTGTCCAGAGAGTAACTGTACTCAATTGTGGG
AAAGGGGTTGCTAACGAAGCCAAGGCTTGCCAATACACTGCGCAGGTGCAGCCCCGCAGAATTATTAATT
TTTTACTAAGACAGTCAAGAGACGGGTAAGCGATCGCAATGAGCGGCAATAGGAAAAATTAATAATGAAT
CAGTCAGCGTCGCAAAAGCC

>Negative Sample 92
ACTTTCGGTTAAAGCCTGAGAGTAAATATTCCTTAACCCCTACCATGTTACATTATGCATCGCAGCAAGG
GATGAAACAGATGGCACTGATTTTGCTCTCAAATATTAAATGCGATCCAACGATCAAGAACAGACTAGGA
AGGACTGCGTGGGATTTGAATAGAAACGACGACGTGAGACACGCGTTTCAAATTGCAAGATACAATCTTG
GAGAGTCTTTCACTAATTGGGACGAAACTCATATCGGTCAGCCATTGAGCAGAGAACAAGTTGATGAAAT
CAATGAAAAGAAAAAAGCAA

>Negative Sample 93
TGCCATAAACAAAATGCTTAGTTTGAGATTAGTATCGTTACCTAATGGACATATTCTCCAGCCTGGTGAC
TCATGTGTTTGGTTGGCGGAAGTGGTTGATATGAAAGATCGGTTTCAAACCACTTTATCGTTGAACATAC
TTAATTCACAGAGAGCAGAGATATTTTTCAACAAGACGTTTACATTTAATGAAGATAATGGAAACTTCCT
ATCATACAAAATTGGGGATCATGGCGAGTCAACTGAACTTGGTCAAATAACCCACTCAAACAAAGCAGAT
ATAAATACCGCAGAAATTCG

>Negative Sample 94
TTTGGTTTACATTTATCAGCGCTAACGATAGCGATACGACTGTTTTTATCACTCATTATGGTCTGTCGTG
TTTTCTTAAGAACTTTTGCAGAAGGCTAGGTGGAATTCGCGCTGAAACAGATCACAATGAAGATCTAAGA
ATACAGAAACAATAAAGGTTAATGCCCAGTTTTACATTTTTTTTTTTTCACTTTCACTTTGCTAGCGAGC
GATGCGATTGAGAAAAAAAAATAGAAAATTTTTTCAGTTGCAAAACTACCCGGCTAGAAATCTGGTTCGAT
GCACACGAGTAACAAGAGAT

>Negative Sample 95
GGTGTAAGCAATCCACTCATGTATTCTTCCACAATCAACGATTATTCAAACGGTACTGGAATTCGCCAAA
ATAGTAATAATATTAATCCCTTAGATGCAGGTCCATCTTTTTCTCCTCTCCACAAAAAACCCAAAATACT
CAACGGTAATGATAATAGCAATCTGGACAGTAATAATTTTGATTACAGTTTCACGGGTAACAAGCAAGAA
TCTAATCCATCAATCTTGAACAATAATACTAATAATAATGATAACTATCGTACATCTTCAATGAACAATA
ACGGCAACAATTATCAAGCG

>Negative Sample 96
GAGTATAAATTTCGGATGTGCTTGGACAGACCTGGATAATTTCTGAAAAAAGACATTGAAGATGCGGCAT
AGCCATGTATCAGCAATGTTGGTGTTCGAACAGTACTTGTGTTTTCATTTTGGAAATGCCATTGATTTAT
TTCGTTGAATAACTTATCATTCTTTTTAATACCTTCAACGTGTACCTCTTCCATAATACGTCTCTGTAAT
TCCTCCAGCCGCATATAAGTTTTGTGTGGGTTATTCAAATGGCTTGACCATAGCTTGAATCCAGATTTTT
TGACAGCATTTTCCTCCTTG

>Negative Sample 97
TCGGCAATATTCAAACAAACAGAGCCTGCCATACCCATATCGGAAAATATATCTACCAAAACACCAGCAC
CGGTAGCTTATAGATCCAACAGACCTACAATAACTGGAGGTTCTGCTATGAATGCCAGTGCTTTGAATAC
ACCAGCAACAACTAAATTACCACCCTATGAAATGGATACTCAGAGAGTTATGTCAAAGCGTAAATTAAGA
GAGTTAGTGAAGACTGTCGGAATTGATGAGGGTGACGGTGAAACTGTCATTGACGGTGATGTTGAGGAAT
TACTATTGGATCTTGCCGAC

>Negative Sample 98
TAAAGATAGTATGATAAATTCTGGGCTAAAAATGGTATTTTGCCATAACGACTTACAGCATGGTAATTTA
CTTTTTAAAAGTAAGGGTAAGGATGACATCTCAGTGGGCGATTTAACAATTATTGACTTTGAGTACGCAG
GCCCTAACCCCGTTGTATTTGATTTATCAAATCATTTGAATGAATGGATGCAAGACTATAATGATGTGCA
GTCTTTCAAGTCTCATATTGATAAGTATCCAAAAGAAGAAGATATTTTAGTTTTTGCACAAAGTTATATA
AATCACATGAATGAGAACCA

>Negative Sample 99
AAATGGTATAACTCCATGCTATACAACCACAGCGAATTCCCGAGCACATGGAGTCGCTGAACGGCTGAAC
CGTACCTTATTAGATGACTGCCGTACTCAACTGCAATGTAGTGGTTTACCGAACCATTTATGGTTCTCTG
CAATCGAATTTTCTACTATTGTGAGAAATTCACTAGCTTCACCTAAAAGCAAAAAATCTCAAAATTCCCA
ATATTCACATAGTCTAAAGTACCGATAGCAACCAACATATATAAACAGTAGTATTTTACGAAGCTGAATT
GCAAGATTAGTGAGAGGAGA

>Negative Sample 100
GATTGATTATTCGTCGTTCCGAATGCGCTACTGCCCACGTTATTGTTGTTGTTAGCTTTGGAGGTTGTAG
TACCAAACGGCGAAGAACCAGCGCCTGCTCCGCTCTGTAGATTACTAAAAGGTGATTTGTTATTACTATT
AGTTCCAAATGTGCCAAATGGGGATACTCCTGTCCCACTGCTTGTACTTGTATTTTGAATAGTACCAAAT
GGCGACTGGGTATTAGTAGCTGCGCCGAAAGTCGGTTTGCCAAACGCTGACGATGTAGATGATGCATTTT
GTGATGCGTTTTGTTGCAGT

>Negative Sample 101
CTCTCGTCAAGTAATCCCAGAGATTTTTGAAATATCTCACTTGTATTCATAAACTTGACAGGATTTGGAT
TTGCTTTGGGACTTCCTTCGATTATGTTTGTGCTATTTACCTTGTTTTCCAACGGATCAACGGGAACATT
CATTTCGTTCATGTCGCCATCAGCCAAGGGAGAATGCAGTGCTTCCTTTATAGCGCTTTGGGTGACTCGA
TCTGGAGAGCTTTTCCATTGAACTAACTGGCCTGACATAGCCCTGATGTTGAAGATTTTCTCACTATGCT
CTTTTCTATGGCGTTTCTCT

>Negative Sample 102
TTGGCCATAAAGCTTGCGAAATATTGGATTCTAAAAGAATTGGAATAAAAGTACCAAAGTTATACGTTTG
GTCTTTAAGTGATAAACCGTTTAGTGTTATCGATGGGTTGTGCACAAAATCGCTGTACCCATTGAGTGAT
GACATAAATACTCCTGAAAGTTTGAGCTCATGCTCAAGCTCAGTAAGTTCACGGGAAAATCAAAAAGGAG

ATGCAACATTTGATAATGATGCTATGATAGCCGACCTTCTAAACATAGGCGGACTCGAAGTAGAGAAGGC
ATCCAATGGCCATATTGAGC

>Negative Sample 103
GGAAGCACGCATCAGAAAACATCTCACTCTCTTTCTCAAGACCTGCGTCCTACGGCTATTTTTCTGTTGG
AAAAAGGGTTCCAATCGTTGAAGATCGTCGAGTGAAACAACTCGACGATATAACAGACAGTAATACAACA
GAAATTTTAACTAGTGTTGACGTTTTAGGAACACATTCACAAACTGGTACTCAACAATCAAATATGTACA
CATCAACCCAAAAAACAGAACTTGAAATTGATAATAAGGATAGTGTCACCGAATGTTCGAAGGACATGAA
AGAAGATGGTCTTTCCTTTG

>Negative Sample 104
ATCACTCCCAAGGTGCTCATTTGATTTATATGCAAAATCAAAATGTGCAGCTTTAAAACCACTAGATTCG
TCTTTAGAAACTTGCTGAAGGCTTTTGGTGGATTTACCACTAGCCGATGTACTAAAGGAAAATTTATCAT
CCCCATCATCCAGATTCGAAGAAAATTCTTTTAGATGATCTGATTGTACTAAATTTCCGTTACTTGCTTT
ATTTACTTCTTCATTATCCACAATAAGTGGTTTTACGTAGCTTAAGGAACTCTCCGCGCTGACATTTCTC
AAAATGGGAGCTTCCATGAC

>Negative Sample 105
ATATTCCATGTCTACGGTAGTTTCACTGGAGAAATGGACTCGTATTTTCTCTCACGATGAATTTTTCCAC
AAAATTATAAGATGAGGAAATGTGTGGGGCAGTCATCCTAACAAAACTCGCACGGACTCCATTTCCCCGG
ACCTTTTTCCCGCTTGTTCTAGGGAATAATTTCGGAATAATTTTAGGGACTTCATAGTATGACTGGGTTT
CAAGGAACGGAAAAACTGGTTAGTACCGCGTTATTCTGCCATTTGCTGATCGTTTTAGTGGAGGAGCGGA
GGAGGGGGCGGGGTGATAAC

>Negative Sample 106
CGACCAAACATCTTCGCTAAGCTGAGAGTCAGAGATGTAGATTCAGATTCTTCCGCTAATACTGTTGTCG
AGAATTCATCCAACTGTTTAGACGTGGGTTCTTCTATAGAGGGAGATGATACCTTCAAAAAACCCCACAA
AACTTCTACCGAACAAGAACTGATTACAAGTATGAGCCTTAGTCAGAGAAACCATGGTTATTCTGATGAT
ATGGAAATAGGTTCACCAAAAAAAACAACATCAACAGACCAATACAACAGAATTTTAAAAAATGATGTTG
CCGCAATTGAAAATGATACT

>Negative Sample 107
GACTCACAGAGAGCAAATCATTCTTTTTACCTTTGCCACCATGAAGACTGTCGATGACCACTGTGGGTAT
GCTTTGCCACTTGACCCATTCCAATGGCTTTTCCCTAATAACGCTGTCTATCACGATATCCACCACCAGC
AATTTGGTATCAAGACGAACTTTGCTCAACCATTTTTCACTTTCTGGGACAATTTGTTCCAAACTAACTT
TAAAGGGTTTGAAGAATATCAAAAGAAGCAAAGACGTGTCACCATCGACAAGTACAAAGAGTTTTTGCAA
GAGAGAGAATTGGAAAAGAA

>Negative Sample 108
TGGATTCTTTTAATGTTAGACAAGGCTATTTAGGAAATTTTGTCAACAATGAAATTCACTACTATTATCC
TCCTGTGAAACCGCAAGGTTGCCACAAATTCAAACTGAGAGTGGACGGTAAGCATTTTAAATTACCAGAG
GTTTGCATTTTATATGCTCACCAAGCCTTTCCGCCAGCTATAGTCAACTTAGTGGCAGATAAGTATGATG
GTATTGTTCTTGCTACCATGGGTGCTGGTTCATTGCCGGAGGAGGTCAATGAAACCTGCATGAAATTGAG
TTTGCCGATCGTATATTCCA

>Negative Sample 109
CCAAGATGCTGAAGAGATTGCTTCTGAAGATATTGCAGATGCATTCCAGCAAATGTACGAAAAGAAAAGA
TACAATGAAATCTTCTTTATGATAGATACATGTCAGGCAAACACAATGTATTCCAAGTTTTATTCTCCAA
ATATTCTGGCTGTAGGCTCTAGTGAAATGGATGAAAGCTCGTACTCGCATCACTCGGACGTAGAAATAGG
GGTTGCTGTTATTGATAGATTCACCTATTACTGTTTGGATTTCCTAGAACAAATCGATAAAAAATTCCACG
TTAACTTTGCAGGACCTCTT

>Negative Sample 110
CTACTGAAGCCACTCTTAAAGCATGATTTTTTTCCTTCCTGCCATCTAAAGGTTACTTACGGCAACCTTT
CCATAAATTGTTGCTGAAATAACATATAATAGTACCAAATGGGCCTATCAATAAGTTTACAGCCTACAAG
ATTTTCTTCCACTAAAATATATTCGAATAGGACTTTGGCCAAAAACTGTTCATTAAGGGGCAGACTGTTC
ATGTTTTCTCGCAAATTACCTTTTTAACAAGGTGAAATTATTTTTTTGGGGAATGCTAACTGTTTATCA
TCTGCCAGGAAAGAAAACTT

>Negative Sample 111
GCTGTCTGCGATGACGAATAGGAAAGAAAAAATTTTCCTTAATTTTGCTTGCTTAATTCTTATACGAATA
ATTCTTCTTAATCAACATCGGGCTTCATATTTACCAACACGCATTATTGATTAACAATACTTGCCTTAGG
TTGAAATTGGACGCCGGAAGAGAATTTATAGCTAGTCAGTGTTCTTCGGCGTAATTTTATTAATGGACAT
ACAGTGTACTTTTCAAACTACACCGCCATTTGATATACCGGTGTAATTTTTGATGAAGTGTCAGAATTAG
CCGCACAGTTAATTGAACAC

>Negative Sample 112
TTCAACTTCCTCTTCGTCTGCTACTTCCATCTGCGGCAGCTTCAACACATCTTCAATTAATCTCTCTATC
GATGTACTCTGAGGTTCACCCTCAACTCTATTTCTTAGTTCACTTCCAAGAGAGTACTTTGGTATGTCTC
CAACAAGTTCCATTATCTGCTGCGATTGACTGGCTCTCAATTCAGACTCGTTATCGTATATCTCTTTGCT
CAGTAAGTCTTGTTTTCCCCTGCTCATGGCATCCTTTCTCTTTTCAATTACTTGAGCCTTTAGTAATCTG
CACACTTCATTTGGTAATTC

>Negative Sample 113
AATCTGTCCTAAGTCCGATTTGTCGAGACAGATTTTGATGAAAGGCTGTGATACAGATGCAGGTCGTTTG
AGAAAGCAGATTGCAATAGAAGGACATCCAAGATTGCATCTGTATAGGGGCTTTTCACGTTTTATGATGG
AAGATTTATCTCTACTGGAACATTTCCAAAGTTATTCCAGAAAAAAATTCAAGAAAATCATTTCAATGAT
CGCTTTTAACATGATTAAGAGAAATGACGCGTATTCGAACTTAGTGGAATTGATATTCCCTCATCATTTG
AGAATTTCTATTCATGCGCA

>Negative Sample 114
GCTTCCGTTACTAAGGAAATTTTGGGTACTGCTCAATCTGTCGGTTGTCGTGTTGATTTCAAGAACCCTC
ATGACATCATTGAAGGTATTAACGCTGGTGAAATTGAAATTCCAGAAAACTAAGGTTGTTTATAACATCA
AAAATCCATTCCATATATTATTCTGTACTTTTAATTTAAAAGGCTTTAATCTAACATATAAACCAAAATA
TTTTACTTCACGTTATATTCTTTTTTTTTACTGTTGCAGCAAATTGTAGTCCGGCTGTGTTCAATCAACC
GTCTACATGTTGAAATAGAT

>Negative Sample 115
ACATACCTAATATTATAGCCTTAATCACAATGGAATCCCAACAATTACATCAAAATCCACATTCTCTACA
TTACTAGTATATTATCATATGCGGTTTAAGAAAATGGTATAAAGATTAAGAAACAGTCATGAAAATTTAG
TGAAGCTGAAATGCCAGTATTGATAATACGATAGAATAATGAATGACAAAGTATATAAGGAAGATGAAGT
AACATTATTATGGAGAACTATCGACTCCCTTTTGTGAATTTCTATATCCTCAAGGAGAATTGCTTGTATA
CTATGTATATATAATATTAT

>Negative Sample 116
CAAAGATGCTGTACCGTTCACGCCGTTTAACGGTGATAGAGAAGCACACCCAAGGTTTACGTTGAAAGGT
TCAGTATACAATGACCCATTCATCAAAGATCTTGAGCACAGGAAAGAATTTATTGCGTCTGGGTTCAACA
CTAATTATGCGTACGAAAGGGTGTTGACAGAGGCATTTATGGGCTTAGGATGTGTTATATCCGAGGAGCT
TTAAAACATCAGGATAGTGTGCAACGTGGCATAAGCTATGTAATCAACTACTTTTTATTTTCTATGTACG
CATATACATGCATTCACGAT

>Negative Sample 117
ATGAATCTTCTTCAAAGTTATCAAATGCGTCAAATTCCCCGGGCATGTTGGCTAACTCCAGCACAGCGAC
TTCTGCATCTGCCAATGAACGACTCCAAGAAAAGCAAACAGAATCATTGTCTAACTACATTACAAAGATG
AGGCGCCGTGATCTGAAAATTCTCGATATACTGCAGTTTATCCACGGTACCCTATGGTCATATCTTTTTA
ATCACGTAAGTGATGATTTGGTTAAATCTTCAGAACGTGACAATGAATATATGATAGTAGATAACTTCCC
CACTTTAACCCAATTTATCC

>Negative Sample 118
CCACTGTAATTTTAATTCGGGGTATTTGTAGGTCATTTCCACAATACTACATTTCAGTACCGATTCGAAA
TCTGAAACTTTGCTCTCTATAATATTCACCAAATCCTCAAATTCAATCTCTTCTTCTTCATCATTGTGTT
CGTTGGCAATGGCTGCAAAACTGTCCTTATTATCATGCTTGCTTTCTCCGCTTTCTTTTCTGCGGGAGAT
TGCACTTAATTTTTGCAAATTATTTAATAATGCATCTTGCAAAACTTCCAGGTCCTGCAAAAATGTCACG
ACCATAATCTTTAATGGCTT

>Negative Sample 119
AGCTGTTGAACAGTAATCCTTCCAAACATCACTGTCTTTACTAGCTTCTTCTTCTGAACATATCCAGTTA
GTCAATTTCAAGTTAAGAGTGTCACCGGATTTAACTCGAATTTCTGGGCCAAATGTAGCGCTGTAGCCAT
TTATGGAGATAACTCTTCTCTTATCAAGCGTGTATTCACTGCTCGCATTGAATTCTAAGACATGTATTTT

GCCTTCCTTAGCGGCCATAATAAATTTTAATAGTATTAAGAGTAGCATTGGCAACTGAACCGGCTTAAGG
AGAATGACCTTCATTCTAAA

>Negative Sample 120
AAGGGACCTTGTAATTTTGGAGGCTTCCTTTTTGGTAAGTTAGTCATAACTTTCATAGGATCATATTTAG
ATAATGGGGGTAAGCCCTTGAGTAACTCATAGGTGGTGATACCCAAGGACCATATATCTGCTTTCTCATT
ATACCCATCAACTTCGCAGCAAACAACTTCCGGAGCCATCCAATACGGCGTTCCTACAAAAGTGTCCCTT
TTCAAAGTGGAACGAATGTGGCCACTTACTCCGAAATCACCCAACTTAACCATACCTTCTTCATTTAGTA
AAATATTAGCTGCTTTGATA

>Negative Sample 121
CCTTCTTTTCGGAATGTGTTGAAGGGATGTAAGGCTTCTACAACAAAAACACCGGTCTTGGCGCCGTCAC
TGTAGAATACATCGTTATATGAGGTAAGAGCGAGTAAAACTTTTTTTGGGGCCATTATAAATTTGTTTGA
GTTTTATCTGTGTAATTAGCCAGTGGGAAGTACTTAAAAATGCCGTTAATAAAATTTAGCGGATGATAAA
GAAATGAATGGTTCTCCTTATGGCTGTTTATATATCTGATTTAAAGGGTAACGGAAACCGGAAGCAATAA
TGGAACCGTGAAGCCATCCC

>Negative Sample 122
CTGCAACGAGGATATCGTTTGTACTCTTGGGCTCTCGGGGCCTTTTTCGCGTTACTCTTGAGATTTTTTG
GTGATGATCTGAACTCGCCGCCTCACCTGCCACTACGTCTTCCGCATTCCGAGTACTAGAGAATGAACAA
TGGTAGTCGCTTTCAAGGCCTAACCATTGAATCCAGCGCTCGGAAGCTCGTTCCTGCAATAAAGTGGCGG
TCGTCCCACTGACACTAGTTAACGAAAAGGAATCCACACCGTAAATATTTCGCCCTGTTTGAACGGAATG
ATTGGCCATGAGTTCACTCG

>Negative Sample 123
CTGCGCTATGCAGGTGCAGCAAAAGCAAACAAAATCTCACTAAGATAAGCGACTGTCTGTCCCCTTCAAG
CACAACATAGAAAACAGAAGGATGTCTCATTCATCGCTTGATTTCCGGCCTGCAAAAATAAAGTAGTCGG
TACGTACGTTCGTTTTCAATTTCCATGGTGCACAGTATCTTAACTATCTGCTTAGTCGAGGAGAACCAGG
ATTCTGTTCGTTGCTCAGCCGCTTCGTGGATATTCTGTTGGATACTTTAAACATGGACCTACGTTCCGCT
CTCGAAAAGACCAATATAAT

>Negative Sample 124
TACTGATATCATTTATGTATTAACATGTCTGAACAATAAGTATCTTTTTAACTGTATAAGAAAGCATGAG
AATTGTTTCAAAAACCTCTCATTTATATATGGAAGGAAATTAGAGAAATATTGTTCGTAGTGTATTCCTT
AAATTTACGGCAGGAAGAGTTAAAAAGAAAATAATTCAAGATTCTCTACTTAAATTAATGCATCCTTTCC
ATTAATAGTTTATAACTGGCGCGATGCAATACACTGGAAAAAATTAATAATAGTTATAAGTACGAGAAAT
ATCTACTCCTAAACCAAATA

>Negative Sample 125
CAACAACATTTTAAAAAGTAGATTTGTCTGCCTCAGCTATGAGATGCGCATGTCCCTAGCATCTCATATC
TGGTTATATTATTTTTTCCACTTGGTGAATGTTGAAAAAAACACCACTCGTCCAATTTATCAGTTTGCAG
GTCTAATGTCCTTCCCTGTTATTTAAACTGTATATTGTAAGCATGTCTTATCGAAACAACTTACTCAGTT
GTCCGAAAACAAAACTGCAAATTCTGTGTGTATTCACGTACTAGAATCCTGTCAAATTGGATCTTGATTT
AAGCTTTTATAGCAACGAAC

>Negative Sample 126
GTGGTGTGTATTTGTCTCAATTTGAGTCCTACATCGACCCTGAAATTAATGATTTCAGATTAACTGGTTT
AGGTAGAGGTGGTCAACAGGTTCAACGAGCCAAGGAAATCTATTCAAGAGCCGTTGAAACTTTAGTTGAA
TTAGCCTCTTTACAGACTGCATTTATTATTTTGGATGAAGTGATTAAAGTGACGAACAGAAGGGTTAATG
CTATCGAACACGTTATTATCCCAAGAACTGAAAACACAATTGCTTACATTAACAGTGAGTTGGATGAGTT
GGACAGAGAAGAATTTTATA

>Negative Sample 127
TTTAGAAAAACTATCCAAGCAAAGGCCACAGTCCGGAATTTTCAATGTTTACATTATGGGATCTATTTTG
TCTCAATTTGCCGTTCATATTGCCACTTTAGTTTACATCACTACAGAGATTTATAAATTGGAACCCAGAG
AGCCACAAGTTGATCTTGAAAAGGAATTTGCTCCATCATTATTGAATACGGGTATTTTCATCATTCAGTT
GGTACAACAAGTCTCTACATTTGCTGTCAATTATCAAGGTGAGCCATTCAGAGAAAACATTAGGAGTAAC
AAAGGTATGTATTATGGTTT

>Negative Sample 128
CCCTGCTGGGGATGCTGAACGTTGCTATACAGTTTCCTTTATATGAAAATTTAAAAATAAGGTTCGGATA
TTCAGAATCGACTGACGTATCGACAGATGTGACGAGCTCAAACTTTCAAAAATTAATATTGGCCTCTATG
CTATCTAAAATGGTAGCATCTACCGTGACTTATCCTCACGAAATACTTCGAACTCGAATGCAACTGAAAT
CCGATCTTCCAAATACTGTTCAACGCCATCTCCTTCCATTGATTAAAATTACGTATAGGCAAGAGGGCTT
CGCCGGCTTTTACTCTGGGT

>Negative Sample 129
CCCCCGTACATCGCTTGCGTAATTTTAATAAATGGGTGGAACTGACTTTGCAAATGGGGCTCCAGTGCGA
GGACGAGTATTGAGTTGGGAGGGACAGAGTCTATATAGTTCACGGCAGGTCTTGGATCATCAATGGGTGC
AAACAGTACTGTGTATGCAGTGCCTACTATGGAGCTATTTTGCGGTGGAGAAATTGCTGTTAGATTAGGG
AAATAACCACCCGTTGGAATGTTATAAACATTCAACAATCCGTCCGAAATATCACAGGTTGAGAACCTCT
GTAACTTTTGAAGATCTGAC

>Negative Sample 130
GAACCAATTTATTCCTCATCTAAATCATAGGCCCAGAATAAACTGAACAGATGCGTATAAAGGCGTTATA
AAGATGCCAGAAGCATATCGCGGGCCACGACTAAAAATGCCGCGAGGTCGCCCTGGACGGTAAGCACGTG
CTCAAGTGTTGCCCGACTCTTGGGGTCCAGCGCCGGGTGAGAGTTTCCGATCACGTGGTCTCCGTGCATC
GCGACTTCGATCCAGCAGCGTGAATCATCGCCGAATACGAAACGTACGTCATGCACATCGCTGGCTCCGG
GTAGTACACGAAGCTGCAAC

>Negative Sample 131
TTATGTTCAAGTCTGTGGAAACCTCGTGAACAAATTTCACATCTAAAGGGTCTTTGGTCATTATCCGGAG
GAAATCTATCTCGCAGGTAATTCATATATGGAATTTAAGAAAAGGATCGCCGATGTGCACCTACTAAGAG
CAAAAGGAAATACTATGAAGCACACCGACTCATGGCAAGAAAGGGTCTCTGTTTTTAAACTTTATCTACT
AGGATTAAGGAAAAAAAGAAGAAAAATAATGAAGCAACAAAAAAAAACTGGAACTGCTTTGCCTTCCAAG
TATTACTGATTGAAGAACAG

>Negative Sample 132
AGCTATCGGCGATCCAGCCTGGAAGACGTATTATGACGAAAATTCCATAGGTGGTTTAACTTTTGCCGTT
CTTGTCCCTAATTCTGTTCACGGATTCGGTCAGTTCTGTTGCGTGTTATTGTCTCTGTCCACTATCGCTA
ATAATGTTCCCAACATGTATACTATTGCTTTATCGGTGCAAGCCACGTGGGAACCTCTTGCGAAAGTCCC
AAGAGTTATTTGGACTTTATTAGGCAATGCGGCCGCACTGGGTATTGCCATTCCTGCCTGCTACTATTTT
TCTACCTTCATGAATTACTT

>Negative Sample 133
GTAGTAGCCGGTCTAGCGTTCCCTCTATTTTTCACCATGATTCTTGGTGCTGCTTGCGGTATGGCGGCCC
TTAATGACCCAACCTGGAAGTCATATTATGATAAAAACGCCATGGGTGGTGTCATATATGCTATCCTGGT
CCCTAACTCTCTAAACGGATTCGGTCAATTCTGCTGTGTTTTGTTGGCTCTTTCAACTGTTGCTAATAAT
GTCCCTGGAATGTACACTGTCGCTTTATCCGCTCAAGCTTTGTGGGCACCTTTGGCTAAAATACCAAGAG
TAGTCTGGACAATGGCAGGT

>Negative Sample 134
GATAGGCATTTTTCGTTGACTTTTTGTTATGTTATGCTAAGAGTTTTCTCTCAATTGTTAGGTATTTGGG
TGTGAAAAGAAAAAAAGGATAAGATGAAATTAGCAAGCCAAGACAAATAATAAGAATTTTGCCAGTGAAG
CAGCTCCTTTATATAATAAAGAAACGTTTTGTGAATTCCGATGTGCCTGGTTAAGGGCGGACTTTGGACT
GACTTATGCTTTTCTCTTTTGCACCAATATTGAGTCACATACTGGGATGCTAGTCCGGACTATGGATATT
TTCTTCTGTAAAGGGTAGTG

>Negative Sample 135
TACCAAACGTTCCACAAGAATTGAAGGACTTATACAAGACTGTTTGGGAAATTTCACAAAAGACTATCAT
TAACATGGCAGCCGATCGTTCTGTCTATATTGATCAATCTCATTCTTTGAATTTGTTCTTACGTGCCCCA
ACTATGGGTAAACTAACAAGTATGCATTTTTACGGATGGAAGAAGGGATTGAAGACCGGTATGTACTATT
TGAGAACCCAAGCTGCATCTGCTGCAATTCAATTTACTATTGATCAGAAGATTGCGGATCAAGCTACAGA
AAACGTTGCTGATATTTCCA

>Negative Sample 136
TGATGGGTCTTCCAGATGATATTCTAACCAAAATGGATGGTTATAGATGGTTGCGAAGGGCTACATCTAT
GGCTAGCAGCTTTGTTTGTGGTGCTCCTTTTGAATTAGCCAATATTTATATGACGGGATATAAAGATCTT
ATCATTTCGGATCCAGATTATGCTATGGCATTGTATGAGAAGGCGGCGGCTTTAGGGCATACTGAATCGG

CAAGAATACTAGAAGACGCTCGCAGAAGTGGAGGGTTTGTTTCGCGAGGACATCCTCCTTCGGCACAGAA
ATATCATAAAACCTCCCATG

>Negative Sample 137
ATCAGTCATACACAAAATTCACCATTTGTACCATATTCAAGTTCTAAAGCTAACCCTAAAAGAAGTAATA
ATGAGCACAACTTACCAAACCACTACACAAACCTTGCAAATAAGAATATCAATTATCAAAGTCAACGAAA
TTACGAACAAGAAAATGATGCTTATTCTGATGACGAGAATGATACATTTTGTTCTAAAATATACAAATAT
TGTTGTTGCTGTTTTTGTTGCTGTTGATAAAGCGATTTTTATACTTTTCTCTTTTTCCTTTTTTTTTTG
ATTGGCTGTTTCCTTATGCC

>Negative Sample 138
AGTCCATGTCATGCTTGAAAGATTTTAATCAACCTTGCATTTGTGGTCGTTAAACTCAAAGGGGATATTC
AAACGACAATCGACAACTAACTTCGAATAAACTAAGATTCACTTTATTGGGATATAATCAATTACCCTTC
ATTAAGTGGTGTATATGTTCCTGACGTATTGTCATTTTCGGGATAAGAGGTTTGATGACTATTTCTCGAT
TTTCATGTCATCCTCTTTACACCGTACGTGATAATACACTAATTACACGATATAACTGAGCAATGATATT
TTAGAGCTTCATCTCCACAA

>Negative Sample 139
ACATTCCTTGTTATAACAACCCTCGGTATTCTTTTAACAAACTTACGATGACTATATTGTATTAGTGTCA
ACCTCATCGCCAGGAACTTTTCATTGAAAATCTTCTGACAAATTTTCTGTGTAAAGGAAAGAAAATGGAG
TATAAAATCGAAAGTTTATTCCAATTGTGCTAGGCTGTAATGGCTTTCTGGTGGGATGGGATACGTTGAG
AATTCTGGCCGAGGAACAAATCCTTCCTCGCGGCTAGACACGGATTGCACGCCCTTTGGGCAAGGGATAG
TTCTCTATTCCGCACCGTGC

>Negative Sample 140
CCAACCAGATGGATTGGCTTGGTTTTGGGTCATCATGCACTGCTGTGGGTACGGCCCATTCTGTGGAGGT
GGTACTGAAGCAGGTTGAGGAGAGGCATGATGGGGGTTCTCTGGAACAGCTGATGAAGCAGGTGTTGTTG
TCTGTTGAGAGTTAGCCTTAGTGGAAGCCTTATCATATTCTTGAATTTTGGAAGCTGAAACGTCTAACGG
ATCTTGATTTGTGTGGACTTCCTTAGAAGTAACCGAAGCACAGGCGCTACCATGAGATATATGTGGGTAA
TTAGATAATTGTTGGGATTC

>Negative Sample 141
ACGAACTCAATATCCACTACAACAAGCAACGCGACCGGAAACTACTTTCAAGATCAAGATAAGTATACAT
TGGTAAATACGGGATTGGGATTGAGTGATGCAAACCTCGATCATTTTATTAGATCTCAATGGAAACACGC
TTCTCGATCAGAATCCAATAATAATACCGGAAATCGCGTTTCTTACAGTGGCTCAACACCAAACAATGTT
GATACAACAAAGACTAATTTGCAAGTGTATACCGAGTTCGATTTTGAAAACCCAGAGTCGTTTTTCCATG
AGCAATCGAAGCTACTAGGT

>Negative Sample 142
AGCATATATTGATACAGAAGGCACTTTCAGGCCCGAGAGGATTAAGCAAATTGCAGAAGGTTATGAATTG
GATCCCGAGTCATGTTTGGCAAACGTTTCATATGCTAGAGCCTTGAATAGTGAACATCAAATGGAACTTG
TTGAACAATTGGGTGAAGAACTTAGTTCTGGAGATTATCGCCTTATCGTGGTAGATTCTATAATGGCAAA
CTTCAGAGTAGACTACTGCGGTAGAGGTGAACTAAGCGAAAGACAGCAAAAGCTAAATCAACATCTTTTC
AAATTGAATAGATTGGCAGA

>Negative Sample 143
ACAGACCATACTATCCAGGTGAGTTTGTCATGGGAAGACGTTCAGATGCACTTGGAGTGACTGGTTTTGA
CCAAAAGGACTGCGCAGGTGAGGGTTTTTACGACGAACAAACTGCAGCAACGTCATGTCAAAATATAGGC
TCCACTCAGTACGCCAAGTCGGTCAGGTCTTATAACTATGGATGCTGCGGCGGGGCGGTCTGGATCAGGA
TTTGGCCGCATCACAATTGTTCAAAAGGTCATGATCACCATTTCAAGATAAGGCCAGGCCAGATGCTATG
CTGGAATGTCAACCCATATT

>Negative Sample 144
AACCCTACCCTAATCCAACCCTTCCATCCTGTCTCTCAACTTACCCTCCATTACCCTGCCTCCCCACTCG
TTACCCTGCCTCCCCACCACCAACCCACAAATCCACCATAACAGTTACCCTCCAATTACCCATATCCAAC
CTCACTGCCACTTACCCTGCCATTACCCTACCATCCACCATGTCCTACTCACTGTACTGTTGTTCTACCC
TCCATATTGAAACGTTAACAAATGATCGTAAACAATGCACACATACTTACCCTGCCAATACCCTACCATG
CCCCATCTGTCATACTCACC

>Negative Sample 145
TTTCAATTCCGGTGATGGCAAAGGTCCGTATTGGACACTTCGTGGAGGCACAGATCCTGGAAGAGCTGCA
AGTAGACTACATTGACGAAAGTGAGGTTTTGACTCCAGCTGATTGGACACATCACATTGAGAAGCATAAC
TTCAAGGTGCCATTTGTTTGCGGTGCCAAGGATCTAGGTGAGGCTTTGAGAAGAATAAACGAAGGTGCTG
CAATGATCCGTACCAAAGGTGAAGCAGGTACCGGTGACGTTTCCGAGGCCGTCAAGCACATCACCAAGAT
TAAGGCGGAGATCCAGCAGT

>Negative Sample 146
CACATGAAGGCCTAGCTCCAAAAATTCTGGCTTGGACCGATCGAAGAGGCGTTCCCATCCCCGCCATCAC
TGTTTTCAACGCCTTGGGCCTAATATCATTGATGAATGTGAGCGTTGGAGCTGCAAATGCGTACTCTTAT
ATCGTTAATCTTTCTGGTGTTGGCGTCTTTATTGTCTGGGGTGTAATAAGTTATACGCACCTGAGAATAA
GGAAGGCGTGGGTTGCTCAAGGAAGATCCATAGAAGAGCTACCTTATGAAGCGCTATTTTATCCGTGGAC
GCCAGTACTTAGTCTGGCCG

>Negative Sample 147
AGTCGTTTATGAAAGAGAAAAGAAAGAAGAAGAAAAATCAAATTTACGGCAAAAACAATAAATCAATGTT
AAAGCACTAGTAATTGCCGGATGAAAAGGTTCAGAAATTGGTTGCGGAACGTTTTTACGCCGGCCAGGGT
TTTTCATAGACCATTTATGAGATATTATACAGCAATATTCTAGCACAAAAGATAATCAACCCGAAATATC
AATTTCATAGGGTATAAACCCAAGAACCTCTTGACCGGTGACTTTTTCTCCGAGTTATATTCTAGACCAA
GCACCTCTTTTCACCTAGCA

>Negative Sample 148
AGAAAAAGCAGTTGCTTAATTTCCCTCTTTTTCATATTCTCTACGCTAGCACTGTAAGGAAAAACGCAGA
AAGCAACATACAACGTTTGTTAGGATGTTCGTACTTCACAATAAGCAGACAATAACCATTTCAAGTGTAA
AAAAAATTTTTTTTCTGTTAGTTTGATGCAGAAGACTTATACTTTTAACTTTTTTGGTTAACTTACACAA
ACCAAGCGCACAACGCCTGCGCTTTTTAGTATTAGATGGCATGATTTGAAAAGCATAAAAGTCTGGATAT
TGGAATTTAGGGAGGCGGCG

>Negative Sample 149
TGGTTAACTTACACAAACCAAGCGCACAACGCCTGCGCTTTTTAGTATTAGATGGCATGATTTGAAAAGC
ATAAAAGTCTGGATATTGGAATTTAGGGAGGCGGCGGGATAGAGCGATAGTTTCTCCCCATATAAAGTCT
ACAAAATGTGTGGGTTTTTGATATCGATCGAAATTTCACAAGGTGTTCTATAATGACTAACATTTTTGTG
AGCAGTTGACTCTCCTTAGTGCACATTGTGGAATATGTATGCCATGTCTCTTAAAGGGGTAAAAAGAGGT
ATTCTGCATACTACCAGTGG

>Negative Sample 150
TGGTATTGCTGTTTTAAGTGAGGAGTTTGTCGAGATAGAGGAGATGATGAACCCAGAAGAAGTGAAAAGA
AAAAGGAAATTAATAAATGATGTCGATGCAGCCCAAAAAAAACTAAGTGAAAGAAAAAAGCATAACAGTT
GGGTGCCGAAGTGGTTGAAACCGAAGAAATCCAAATGGAAGGTCATGGTCGAAGAAGCTGTCGAAGAAGG
AAGAGATATGCAAGACCTACCAGAAAATGACGTCAATAACAATGAAAACGAAAATCCAGATGAACATGAA
GGGATAGCAAGGCAAAAACG

>Negative Sample 151
ACCAGCTAGGCTTGCAGACCGTCATTAGGGGCAACTCCATAATATCCCTAGAGGCTCTAGATGCCATATA
AAATTATATATATATATATATATATGTCGACCTCTATGTACTATACAGCAATACTATTTTCTAATTTGCATT
TCCACGCCTGCGTTGCTAAACCCACATTTTTCATAGAATTTGACATTTTTCTCATCGCAATCTAAAATAA
TCTTATAACAACCGTAGTCAAAGCCGATAGTTACCAATTGATCAATCAAGAGCTTGCCCAAACCTTGGCC
CTGATACTTGGAGTTTACTG

>Negative Sample 152
TACAGCTTTGGTCGGTTTATTAAGATTATCCGACAAATTTTTGGTCAGTGCCGCTGCAGACGGTTCAATA
AGGGGTTGGGACGCAAACGACTACTCTAGAAAATTTTCCTACCATCATACCAATTTGAGTGCAATTACCA
CATTTTATGTATCGGATAATATTTTGGTGAGTGGATCGGAAAATCAGTTCAACATCTATAATCTACGGAG
TGGGAAATTGGTCCACGCAAATATTCTAAAAGATGCTGATCAGATTTGGTCGGTTAATTTTAAGGGCAAA
ACACTTGTTGCAGCAGTTGA

>Negative Sample 153
CCCAAATGCCTTCATTGACGTTGATTTCCTAGAAAACGTTCAATCTCTCTCACACGTAAATGAGCCCTTC
ACGAGAACCAATTTTGCAATCAGAGCAAACAGTTTGCACCAGAGTAGAGTTCTATTACATTCAACAAATA
GAAAAGCTTCCAAGCTGGAAAACCTATTGTTAGTTGACATCATACAGTTAGCGACATCCCTTTATCCTGA

TATTTATAAACCAGCACAGGGAACTTTGGTACACTGTATGAAACAATTAGTTGGGTCATATGGCGTAGTC
ATCAATAAAATTATTCCATC

>Negative Sample 154
GTATTGTGTAATTATTTTCTTTCAAATTATATGTGAATGAACAAACCAAATCCAGGATAGCCACTGCTTC
TGCAATCATAAATAGTTCAGAAATATGGGTTGCAATTTTATCGAGCAGTTCATCAACTGTCTCTTCACTT
AAAAGTAAAATCTCTTCCATAACTTCCTTCAACCTCGCATTTTTTTTTATTATATTTAGGGTTGTACATT
CAATATAATTTTTTTTTATTGTTCTTGAAATGAATACGTCTGGAAGTGTAGCGACGTCATCGGTAAATTC
CTGTCTTTTTATTCTGAGAT

>Negative Sample 155
GTCTGATAATAGTGAATTGTTCAAAGTATCTTCAAACTTCTTTAACGTTTCAACTTCTAGGTTAGATAGC
CTTTGTGAAACCTTCTTCTCAATGAACTTTTCTGCATTTTGCTTGGTGACATAGTCTGATTCCTTCGATG
AATCCAATTGAGAAACCAAAGACTGTGAAAAATCGATCTGTGTTAGCGGAGTATAGACTTGTTGTTTTTC
TGCACCAGCACCCACTTTGGAAGTGTTTGAACCCATGATTGACTCTGTATTAGTCGATATACCACCTTGT
TTTTGTTCTTGAATTTTTTT

>Negative Sample 156
TTCTATACCAACTAGCTTTCATTCCTCTAGTTTTATGAGTAGCACTGCTTCCCCTATTTCCATAATTAAT
GGCTCTAGAAGTAGTTCTAGTGGGGTATCTCTTAATACACTTAATGAGTTAACTTCGAAAACTTCGAATA
ACCCATCCAGTAATAGTATGAAAAGGTCACCAACAAGACGGAGGGCTACTTCTTTAGCTGGGTTTATGGG
AGGTTTTCTATCAAAGGGTAACAAACGATAGTCGCCGATATTGTTTCGGCTCGTGCATTAAGCTTGTATA
GTTTCATATAGAAAGTGATG

>Negative Sample 157
GAGAAGAGAATACTCAATTGCCTCCTCTCAGAAAGTTCATCCAAATGAAGTTCATTTATTGATCGTTGTT
GTTGATTGGGTGGATAATAAAGGAAGAAAAAGGTACGGTCAAGCTTCTAAGTATATCTCAGACCTTGCTG
TCGGTTCAGAATTGGTCGTTAGCGTTAAACCATCTGTTATGAAATTACCACCATCTCCAAAGCAACCAGT
TATTATGAGTGGTTTAGGTACTGGTTTGGCACCATTCAAGGCCATTGTTGAAGAGAAATTATGGCAAAAG
CAGCAAGGTTATGAGATTGG

>Negative Sample 158
TGACAGCTTCGAGAATTACTTCAACCAATTGAAGCCTTACTACTTTAGCAACAATCATAAATTATCTGAA
TCTGACAAGAAATCGAAGCTGATAAGTCTGTATTTGTTGAACTTATTGTCTCAGAATAACACAACCAAGT
TTCACTCGGAATTGCAGTATCTAGATAAACATATCAAGAACTTGGAAGACGATTCACTTTTGTCTTACCC
TATCAAACTAGACAGATGGCTCATGGAAGGGTCGTACCAGAAAGCATGGGATCTTCTGCAATCTGGGTCG
CAGAATATATCAGAATTCGA

>Negative Sample 159
ACGTACTCCTTAAAAGCGTCTAAATCTTCATTTTTCCGGTTTATTTCCAACCGGGAAATAAATTATTCCT
AATAAAATTTCCGGGGTTTGCAGGATGCGGGGTAAAAGTAAAAAAATGAAAAAGATGTAAAAAGAAAAGT
TCGAGGTAAGACCAGGTGCAAGGAGAATACTGGGCGCCAATGCTTTTACTGCAGCGAAGAAAAATTCTAA
CCGTCTGTTTCGGTTGCTAATTTTTGTCTTACTTTCGATGGTTAGGCTTCGTGGGGTAAAAGTCGAGCTT
GTTTTCTGAAGCGGAAATTA

>Negative Sample 160
AAGGTCAGAGAATAAAACGCTTTTCCGTTAAAAAGATCCACCATATGGAAGAAAAATACGAGAGTTAAAA
TGCTATTTAAAAGTGCTAAAGGAATCCCCAGAGACCTCAGCTGAGATAAGTAATATCGTTGATGAATTAC
AAGGGAACAATGAGCAGAGGGAAAATGGACAGATCCTTTCGCATTCCTACTTGTTTGATGAGTTCAAATG
TGAAATAGTAGCAACAAAAGGTCATAAATCTTCAATGATATTTGGACCTACTAGTGTCTATAGTAAGTGC
TCGGCCAAGTCAAGCGGTAT

>Negative Sample 161
TATATATTTAATAATAAATACATTATTGGAATGGCTAGTAAATTTTGACATAAACATGATTTCCGAACGA
ATTAAAATACAGTTGATTTAGGAGAAAGTATCAGTACATTGCTAAAAATTTTAAGCAAATGTTAAGTGAA
AAAGGCTTATCATTCAGATGAGCTTAAGAATATACTTTTTGCGTTATTCAAGAGCGATGATTATTACTGA
ACTACTGTGACGATATCAGCAGCATGCCTGATAAATAAATCTGTACATTCGTGGAAAGTAGTATGTACTT
TTCAAATATTTCTAAGAGCT

>Negative Sample 162
TTTTCATTCTATTTATATAGAAAAGAATACGCTTTTTTATTATTTATGAAAGGAACATGAAAAAACTAC
GAATGTACAAAAGCAGAGATTGCTAGAGAATACATTGGTCAAGAATTTTCGCATATGGAACGCATTGCTC
TTTTAGATGGGCTGTTAAATAATCGTAATTTAGGTTAGTTATTGCCCTTTGCGTCTTCCGTGGAACAATG
TTGGTCCAATAAATTATAAAGAAAAGAAATTGGATAGCACAAATAAAAGAGCAAGCGATAATGCAAAACA
TTAAAAGTCTGTTTTCCATT

>Negative Sample 163
TAGATGATAGTTGATTTTTATTCCAACAGTTTATCAACTAGTAGTCACACTACCAATATATTATCATATA
CGGTGTTAGAAGATGACATAAGGTATGAGAAGCTTTCATCGAAGGTAGAGGAAGCAGAAATACAAGGATT
TATAATGTAATAGGGTAATGGAACATATAAGGGAGGAGAAATGATGGTGATATTAATATGCAGAAATATC
GATTCCATTTTTTCAAAAAAATAGTGCCAAACTATTAAGAAGTAGCGAAACAGCAAAGGCATATTTCCAC
ATTTCGCTTTTAGCAATATA

>Negative Sample 164
AGTTCTCCAAAGCAGGACCATGACGAATTTTTGAAGTCCTTGGCAAACACTTTCCTTTCCTTGGATACAG
AAGGCCGTGTTATTAGAATGGATTCCTTTTCAAAAGTTTTGGCCCCAGGGACAAGATTGGGTTGGATTAC
TGGTTCATCCAAAATCTTGAAGCCTTACTTGAGTTTGCATGAAATGACGATTCAAGCCCCAGCAGGTTTT
ACACAAGTTTTGGTCAACGCTACGCTATCCAGGTGGGGTCAAAAGGGTTACTTGGACTGGTTGCTTGGCC
TGCGTCATGAATACACTTTG

>Negative Sample 165
ACGAATGCCTATGGTGACTACCCTTTTAAAACTAACATACATCTAAAAAGTAACGACGTTGAAGATAAAG
AGAAGTATCATAGTGTGTTGGATGAAAATGACAGAATAATCATAAGAAAGTATATTGATAGTATTGCAAA
CAGGTTGAAAATATTGAGGCAGAAGATGAACCACCAAAATTAAGTGATGCATAATATGGTTTTCTTATTA
AGTACAAATGACCTGCTATATTTTCAAGTATCTTCATGATTATGTTATGCTACTCTCGAAATGCTTTACG
AGTTCGTCATTAAAGTATTC

>Negative Sample 166
TGCTCCTTCAGCGAATGCGTCTACTAGGCCATCTCCTACTTTAGCGTCTCATGTTTATCATGATCTAAAG
GATAAAATTCCCATAATTCTTGATGGCGGAGCGTGCAAGGTTGGTGTAGAAAGTACTGTTGTTGATGGGT
TATGTAATCCTCCTACGCTACTGCGACCTGGCGGTTTTACATATGAAGAAATTGTTAAATTGGGTGGTGA
AGCTTGGTCTCTTTGTAAAGTTGAGAATAAGAAAACAGTTGAAAAGGGCGAAAAGGTAAGAACGCCTGGT
ATGAAGTATAGACACTATTC

>Negative Sample 167
TATTTGGAAGTAATATAAAGATAGAGCTTAATCAGTATCAAATAAACGACATCAGTTCAGAAGGTAATGG
CGCTGTTTCTAACAGTGATATTATCTTTGGTTGTCTACCTACTCTAGAGCCCAACTTGTTTTTGAGGCAG
TTGCTAAACAGTAAAGCTTCTGTGGAGCAGAAACATACTTATATCTCATTAATTGGAAGTTACAAACCAG
TGATGCACGAATGCGATAAGGAGTTGATTGATAAGTTCAAATCTGATAATGAAAGCGCTTGCATTTTGGT
AGATTCAAGGGAACATACTT

>Negative Sample 168
TAATTTCCGATGAAGTATGGGAGAAATGGATCAACTATGAAGTTGAAATGGCCAATAGGCAATTCATCAA
TATAACTAAAAATCCGGAAGATTTCCCAAAATCTTCTCAGAATTTTGTCAAATTAATCAATAAAATTTAC
GATTATTTGGAACCGTTCTACGATTTGGATTTTGATCTATTAGTAAGATACAAACTAATGACTTGTTCAT
TAATTTTTATGAACTTGACTTCATCCTACTTGGATTACATTTTAACTGTCGATTCATTGAATGAAACAAG
AACTAAAGAGCAGGAATTGT

>Negative Sample 169
TTATTGAATTACTCGAACGGGTTTTTATCTTCTTTTTCTGATCTGGGAAGCTATGATTCGGTGTTGTATA
ATAATTTGATCAAATTATTGAACATGACCACTGACGAGATCAAGTCTTTAGATTTAACCTTTGAAATAGA
TGAGCCTGAAAGTTCCGCAAAAGTTGTTGACTTAATTCCGAATGGTTCAAAAACGTACGTGACGAAGGAT
AATGTGTTGCTCTACGTTACTAAAGTAACGGATTACAAATTAAACAAAAGATGCTTCAAACCAGTTTCGG
CATTCCATGGAGGGCTCAGT

>Negative Sample 170
TATGACACGTATGGAGGGAACTGAAATAATGAAATTGCCCAAGGCATCACAAGAAACCAAAAATTTTATT
TCGGAGAATATCATGAGACTATGTCTGGAAGAAATTGCCACTTTCAAGTATATGCAAACGGATCCTAATT
GGGCAAATTTCCTCTATAACGGTAGGACAAAGAAAATTGAATTATTGGATTTCGGTGCCTCCAGACCTTT

TGCAGAAGATTTTATTCTAAAATATAGAAAACTTTTAACTTACGCCACATTGAGAGATAGGAAAGGGGCG
TACGAAATGTCTGTACAACT

>Negative Sample 171
GATTTTCGGGCGAACTAGCTTGTAACCCAGCATAGAACATCAAGGGAACCACGACCAGAAATGCAATGTA
GTAGAATTTGAACTCAGTAGTTTTCCATAGTGATGGCTTAGTGGTAGTAGAGGCATCCTTTTTTGGTGAA
GGTTTGATTCTTGAATCTAAGCCCTCGGAAGTAATTAGGGGAGACAGGATGCTGATCAGCGACATTTCTT
GCAATTGTCTGTTAACAAATGCTGATTCACGCTTTGTTTTTGCCTTTGTATGATTAACATCTATATATCG
AAAGCAATATGCTAAGTTAT

>Negative Sample 172
ATCAGACGTTTTAAGGAACAAGAGAAGAAAGTTGACTTGTCGTCCAGGCCTGGAACTAGAGCCATTTGAT
CTCGAAAAAATTAGAGAAGACTTGCAGAATAGATTTGGTGATGTTGATGAGTGCGACGTTGCTTCTTATA
ACATGTACCCAAGAGTTTATGAAGACTTCCAAAAGATGAGAGAAACGTATGGTGATTTATCTGTATTGCC
AACAAGAAGCTTTTTGTCTCCACTAGAGACTGACGAAGAAATTGAAGTTGTAATCGAACAAGGTAAAACG
CTAATTATCAAGCTACAGGC

>Negative Sample 173
TATAACCACCTGCTAGAACAGATGAGATTTCTGTTGGTTCTGTTTCCAAAAATTCAGCTGTATGCATGGT
TTTGTCAATTTTCTAGGTATGAATTACTGGAGTAAGCAATAATATGAGTAAGCAGTAAAACTTTTTTTA
TTTGGCTGATTGGGCAAGAAAATCAAAGACAATAGAGTGGCGCATAGATGTTGCTAGCATTTATTAGGTT
GCCTTACTCTTATATAAATCAAGCGGGAAGCTTGCCTCAAGCCAATCACCGCACCGAACAGGGATTGCTC
GTTTACCCGTATTTTGATTT

>Negative Sample 174
TGGTTGCCATCTCATCGCATCCAATGCTGTCTGTAATCATTTTATTGGACTTTTCTTTGTTTGCTAATCC
CCCTTTTCTTTTTCCCGTTCTTTTCTTGTCCTTGTTTTTCATTTTAACTATATTTCTGAAATTAAGGAAA
AATAAGGCAAAAAGTGGGTGGATGCATCTGCGACTTCTCTAGTTTTGCGCCTACTTGGGCGCGCTAATAA
TAAGGGTTTAGCTTCTTAAAATAAAAATGTCAGTAACAGTTGTACGATAAGGTCTTTACTTTTTTTCTCT
CGAGAAATGTCTTTTTCTAA

>Negative Sample 175
TTCAAACTTTTGTTCATGCGATTGCAAATTTGAGCCTCTTCTATCTTTCTTGAACACTGGGGGTATTTCA
CCATTATCAATTAAGTCATCAATCTCATCGCCTGGCTTACCCCAATTACCCTTGCCGGAACCTTGCTTCT
TGATGTGATTTGGAGATTCACCGTAGTTACCAGTGTGCGAAAAGTACTTTGGATCAGCCTTTCCTTCACG
TTCGGTCCACTTGTTTGTTCTCGTCATTTTTTCTGATTGGTTGTACACTTGAGCGGTTTGTTACTGTTGA
TTGTTCGTTTATTTGTATAA

>Negative Sample 176
ATATATAATTAAATATTTCGAGATTTACATACTAGTGGTTACTTAAAAGTGTAATCGCAAATTATATCAG
AAATGAACAAAGATACGCAGTCCAAATTATTGACAACAACCAGCATATGTACTTAGTAATATTTTATGAA
GTTACTAAATCAAGATTAGAAACGTAAAAATAGAATAACATGATCAAGTTCAAGTCTGAATTTCCTTTAA
TATTTGGATAAGTAAATTCACTACGTGACATGATTGGTTGGTGCCAACATAACACAATTTTAACATTATG
TAATCTACAGGATAAGTAGC

>Negative Sample 177
AACGGATAGTGTGGGTCCGGTTGATAATACGCAGGTGACGTTTGATAATGTGAACACGTCATCATAGATG
GTTGTTGGTAATGTGCCCAGTTAGAGGCCATAGCTTTGTTTGGGGTCATCATGCCGTGCTGTTGGTACTG
TCCATTCTGTGGAGGTGGTACTGAAGCAGGTTGAGGAGAGACATGATGATGGTTCTCTGGAACAGCTGAT
GTCCCAGGTGTTGTCTCTTGTTGAGAATTAACCTTAGTGGAATCTCTATCAAATTCCGGTAAATTGGAAG
CTGAAACGGCTAACGGATCT

>Negative Sample 178
CTGCTTCGTTCGATAGCAGAGAAGATGTACGAGACATGCAAGTGGCCACACAGCTATTTCATAACCATGA
TGTAAAGGGCAAAAACCGACTGACAGCTGAGGAACTACAGAACTTACTACAAAACGACGACAACTCCCAT
TTTTGTATATCATCAGTAGATGCGCTGATAAATTTATTTGGTGCTTCCAGGTTTGGCACTGTCAACCAGG
CAGAATTCATCGCCCTATACAAAAGAGTGAAAAGTTGGAGAAAAGTTTATGTGGACAATGATATCAACGG
ATCGCTCACCATTTCTGTAA

>Negative Sample 179
ACGTGGGTTTTTATTTATACTGTATATAAAAGAGGACTGCAATAGCACAAGATTAAGATAGAATGGCTT
CAAACAGCCGCCTTTTATACATATTGGTAAAAGCTCGCGAATCGCACCATATCCCTTATCCTGTAATCAA
ATCGATCTAGGTGCAGATACAGATCAATTCATAAAAAGAAATTGAAGCACCAGTTTATCACTACTACACT
ATCTTTTTCTTTTTTTTTTTTTTTTGCGAAGTTTCGCCCTTTGTTCAATATCACTTGATAAGTTGTGGGC
TTTTTCTGTCACTCATTCGG

>Negative Sample 180
TTTTGAGACAAGAAATTGATTATTTTAAAGGCAGGATTTCACAGAACGCAACACATGACGAAAAGAGCAC
TTTAAAAGTCGAAAATTTGAAGGTTGCCCTACAGCAAAGTGAGACTGCGGTAAATAAGTTAGAGATGCAA
TTGAAAACTCTCACTGAAAAAGAGCTTGAATTAGAAGAGGAATACTTGAAAAAGAAAGATCTACACTTGA
AAAATCAATTAGAGTTCAGTAAGTTAGAAGAAAGCTTATCGAAAGACCTGAAAAACTCAGAAGGGAGGTT
CCAAAAGGTCAATCAAGAAT

>Negative Sample 181
GATCCAGAACAGTCAAAACGGTTTGAAGCCTTGCATAGATACGCATATGCTATTTTCAATAAACGTGGTT
CAGGTGAATACTTATTAAGCTTTGCTTTGAAATGTGGTGGTGAACCAAGACTATCATTGGAGCAGCAGCT
ATTCGATGGCAAAAAGTCTGATATTTTAAAGAATAGTAATTGTGACTGGCTTTGGCTTTATGGCGATGAC
GATTGGATGGATGTGAATGGTGGACTTAGAGTATCAAGATTCTTGAAAGAGAAGTTGAAACAAAAAAGTA
ACGTCATCATTGTTCCTCAT

>Negative Sample 182
GACTACCCGACATATGATTATATCCAAAGCCATCTAAATGCATATTATAACGCCAATTTAACTGATTGGG
AACAGGCTGGATATACTTTCCCAAAAAATATTCTAACAGGAGGTTGTAGTAGTTCAAAATTCTCATTATC
CTGAGGTTGATTTTTTTTTTTCAACTGGTTAAAACCTACTATCTAAGTGCGCTTTAATTCTAAGGAGGCA
AAATAGTCTGCGGTCACAGTCGTTGAAAGTTCTAGATTTGGGTACGGAAGCAGCCATGTAAATGGTCTTC
CTTTGTAATAATATCATCAC

>Negative Sample 183
ATTGTGAAGAAATCTATTAAAAATGGCCTATGGTCAAAAAGCACACGGAATGGTAACGAACTCCTGTCCA
GTTTGGCAAATAATACAATAATCGCTAAACACACAAGAATTTGCTGGAATTTTATTAATGTACCCTGGGA
AAAATTTTTTTTTTTCAACCATTGTGTTAATTTTGCGATCTTTAATTTGGAGGCTATAGCTTCCACAAGA
AAGTGTGCGATAATCCACGAAGGTACCTCACCCTTGGTAAGCATTAACGATATTCCTACAATGGAAGACC
AGTGAATATAATGATTTTTT

>Negative Sample 184
TTTAGGTCCTGGTTAATGGCGAGACATTTCAGTTGTTGGGTCGAAAGATCAAACCCAAGATAGTATGAGT
CTAAAGACATTGTGTTGGAAACCTCTCTTGTCTGTCTCTGAATTACTGAACACAACATTAAAGTACTAAT
CTCATCCTCCTTTTGTTTTTCTCGAGAGGGCCCCCTTATTCGTCCGCCTGAGATTGCATTGGCCGAATTG
AAAAGTGACAGTTATGCACCTCAGCGGCTATTCCCTGCGTCGCTTGCTGAAGATTGAGGATTATAGGAAG
TAGTAAGCTGGAAATGGTAA

>Negative Sample 185
CCACCGACATCGAAAACTTTAACAACGAGGTGCAAACATTCAGAGCTTCTTGTCCATCGTGTACCCAAGA
GTGTGAAACTCATATGAAGCCAGTAAATATCCCACACTTTAAAGAAGTCATTATCATGTCGACGGTCTGC
GATCATTGTGGTTATAAGTCTAATGAGGTGAAGACCGGTGGTGCCATCCCTGACAAAGGAAGAAGGATTA
CTTTATACTGTGACGATGCAGCTGACTTGTCCCGTGATATTTTGAAATCTGAGACCTGTAGTATGGTAAT
TCCTGAATTACATCTTGATA

>Negative Sample 186
AAAACTAAGGCCATCATATATATATATGCGGCTGCGTGCGTGTATTCTCCCGGATAATATGGTGCGTTGC
AATTGGAGTATTGGAGAAAATTTTCTTTTCCCTTTCATTACGGCGGAAATACTTCATATAAAAAAAAAGA
ATACAATCAGTCTTTAAGACTATACGCATAAGCATTCAAGACACATAGAAACACAAACCTATATTTTTAA
TGTCAGCATCAGCTTTTAATTTTGCCTTTAGAAGATTTTGGAATAGTGAAACAGGCCCTAAAACAGTACA
CTTCTGGGCCCCAACTTTGA

>Negative Sample 187
TGCGTTACTGCAGCAGTACGCGCATGTTCAATCATGGAACGAGCATCTGCTGTAAGCCCACTCATTGCAC
AACCAATATGACGATCAATCTCCACGATTTTCTCAATAGAATCAGATTCTAATAGTGGTGAGGTGGCACG
TTTCTCCACACCTAGTACAACACCTTCTTTCGTGGCAATACCAATTGCTGTAGATCCTAATTTGATGGCC

TCCAAAGAATACTCAACTTGGAATAACCTCCCTTCTGGGGAAAATGTGCTTACACCACGATCATATTCAC
TTCTAGTTAAGAACATCCTT

>Negative Sample 188
ACATTCAAACTGCTGAAGAAGCTTTGGACTTGATTGTTGACGCTATCAAGGCTGCTGGTCACGACGGTAA
GATCAAGATCGGTTTGGACTGTGCTTCCTCTGAATTCTTCAAGGACGGTAAGTACGACTTGGACTTCAAG
AATCCAAACTCTGACAAATCCAAGTGGTTGACTGGTCCTCAATTGGCTGACTTGTACCACTCCTTGATGA
AGAGATACCCAATTGTCTCCATCGAAGATCCATTTGCTGAAGATGACTGGGAAGCTTGGTCTCACTTCTT
CAAGACCGCTGGTATTCAAA

>Negative Sample 189
ATCACATGAGTCGTACTGAGCTCTCTTGTTGGAATCAGTCAAAGTTTCAAAGGCCTTTTGAATAATCTTG
AAAAAGCCATCTTGGTCCAAACTACCACCAGCAGCAGATTGCTTGTCTGGATGGTACTTGACAACTTGTT
TTCTGTGAGCCTTGATGATTTGACTTTCAGTAGCTCTGAAACGCAACTTAGACAAACCCATAGCAGCATA
CAAATCGGCAGTTTTCCAGTCTCTAGCATCATGAGTCAGTAAATCTTCATCGGCCAATTCAGTGTCGAAT
AACAACTCATCTGGGTCGAC

>Negative Sample 190
AACGACTTCCCAATCATGAGGATCCGAACTTGGTGTGTTTTTAGTAATCTCTTTGCAAAACTGAGTGAGT
TGTTTCGATAAGGAACGTTTACGAGAAAGTCTAAAAATAACAAGAACAAATACTGGTCCCCAAAACAGGA
CAAGAAGTGAAACCATAAGCGGGTAAATCCAATTGTTGGAAAGTTTCCACCAGACGCTAAGTGGTAGCCA
TAATAGCATCCAGATGCGAAACACTAAGGACTTGTAAATTTCATAACAAAACCAGGTAAAGCTGCTTCTG
AAAAGATCTTGAGGTAGAAC

>Negative Sample 191
CGAAATTAACAATAGTATGTTGATTGTTATGCTTTTTGACAAGAAATCCATCAATATAAACAAAAGATTG
TCCAGTTTCCGTCTTATCATCATCTAAGAATGTAATAATTAAGCTACTCTATTGAGAAGAAGCAGAATCT
GCGCTATGCAGGTGCAAATCTCCAGCAGCAGCAAAGAAAATTCAGTTAATAAGAAATCTCACTAAGATAA
GCGACTGTCTGTCCCAACATAGAAAACAGAAGGATGTCTCATTCATCGCTTGATTTCCGGCCTGCAAAAA
TAAAGCAGTCGGTACGTGCT

>Negative Sample 192
TCCGTTCCTTATATCGAAGAGGCCTTGCAAGTGTATGCTGCAGGATTTGACAAAGAGTGGAAGCTGTTTA
ATACTGAGAAGGAAGAGAGTCCTTTCGACCTGGAAGACATTCAGCTCCCCAAAGAAGCTTACCGATTTAA
GCTTACCTGGATTCTTAAAAGGATTTTCAACCTGCGGTGTTTACCACTCTTCCTTTATTATTTTTTAATT
GTCTACACGTCTGGAAATGCTGACCTTATATCACGTTTCTTGTTTCCGGTAGTAATGTTTTTTATCATGA
CGAGAGATTTCCAAAATATG

>Negative Sample 193
TCATGTAATGTACACTGAAGTTTCGAGAAAAAGTATGAAATATGTTAAGAAGATACTACCGCTTTACGCG
AGATATATCAATGTGAATCCACAAGAAGAGACGATCACCTTCTTAATTGAAAAAGCCAAAGAAATGATTC
GATTGTTGGGCAGCGAATCTGATGATAGTGAAGGTTCCATTAAGCAGACTTCCGATGAATCCACAATAAA
AAGAATTAAGCCTAACACTGAAATTACACAAAAGTCATCCAAAGAGAACATTGAGAACGAAGAGTATGTG
ATCAACTTGTTAAAAGTTAG

>Negative Sample 194
TTTAAGGATTTTCTTGGAGAGAGCTTGAAATTAAAGCGTATAACGTCCAGCCACTGGACTACGTTTGCTT
GTCTTTGAAACAATTCCACGTTGGAGTAGAAGATATCTCTCACTGTAGTGTTTTTACCTAGCGGTAATTT
TTCCATAACGACTTTCAATAAGTTTAAAAGGATTGCGCATCTTTTCAACTTGAACTGATGAGTGCATAGA
TGCGGGCCATTCAAAGGGAAGTCCAATGTTGTGTGAATGTCAGGACTGCTTGTATCGCCTTTTTTTTTTT
TGTTTTGAAAGACGATTGAA

>Negative Sample 195
CACGAAATCATCAAATCATAGATAGTTACTGCTTTTCTCAGAACTCCAACGGAGAAAAATAGGATATTAG
AGTTGAAGAGGTCAGCACCCATCATTACTACATAGAATAGTCCCATGGCGAAATTAACACCAGTAATAAG
ATTCACAATACCCGGGTTTCGTGCGACTATGTCAGGATCTTCGGAATATACCGCTACCAATAGGAACGAA
CCACTACTAAACAGAACGCCACCAAGTATGGAATTTATTAGCAATGTATCTAGTTGCAGTCTTGCTTTCT
TCATTGCAGTGGCCACCACC

>Negative Sample 196
TGATTTCGTGGCAACACCCATGGAAGAAATAACATCTATTTTATTGTGATGGGCGTACTCTAATAGGTCT
ACCTTAGATTCTAGATTATCTAGACAATCCACGATAAAAGTTGGGGACTCACCGTCTGCAAAAATTAAAT
CGTGTGAGTTTTCTTTAGTCCAGGCCTTGGCCCTTGCTTTTATTTCAGACCAAGGAGCAATTTTAGACAA
GTGTTCTTTCAAGCATTGAACTTTGGGCTTTCCAATATCTGATAAAACTGCGCAACAGTGGGTATTCAAA
GAGTCGATAGATATGTTTTC

>Negative Sample 197
CGGTCTCGATTTCTAATAACGGAGATTTTGTTGGCCAAGAGACACCAAGGGCAGCACCAAAGTTAAGTAA
AAAGAGACTTTCAAATACATTGGATGTTAATTGTTCCGATTATGAGTCTAGTGGACAAAATGCAACTTAT
AATGATAGTGAGTCTTCCCTTAATTAAAAAGATTAGTTTACTTTTTCTGTAGAATGTTAATTAGTTTATT
GCATAGAAAATCTTTAATACTGAATGACAAAATTTCCACAATTTTTTTAAAAAAATTATCGACAACTGC
AGGACTCGAACCTGCGCGGG

>Negative Sample 198
TCACACACGTCTTTCCCTGAAACGCTTGAGAGGAAGATTAGCCAAGGTATATAGCAGCATTATTTCGTTT
TTTTGCCGGTCATCTTCTAAATCATCCAAATAATATCCTTGATTAGATAACAATTGCTTATCTACGTATA
TATATACATAGCACAATCTCGAAAATATAATACTAATAACAAAATAAGTATTTAAAGAAGGGTTGATGAA
GTCCTAGAAGTACCAGACCTGATGAAATTCTTGCGCATAACGTCGCCATCTGCTAATTTAATTGGGATTT
CAAATTATCCAATTGCTTCA

>Negative Sample 199
TTTTGATGGCGGCTTCCTTTTTAGGGCCCGCTAGGATAATTCCCACATGGTCATTCTGTTTTGTCTGGTG
TGCAAGCATAGCAAAATTTGGGTTTGCATTGAAGCAAGTTTCTGCATCTTCTACTAGCGCCACGATCCAC
TCTGGCCCAGTATGTAAAAGAGCAGGAGGCTTTATGAATTTCAAACCAATCGCTTTTTCATAATCAGCAA
TCATCTCACTCGATATACTTTCGTAATCAGCCATCGGAGCTTTGAAGCTAATTAGTCCCTCATTAATTGT
TATTGGAACAGCGCCTATTT

>Negative Sample 200
TACCGCAGGGGCATTTGTCGTCGCTGTTACACCCCGTTGGGCAGCTACATGATTTTTGGCATTGTTCATT
ATTTTTGCAGCTACCACATTGGCATTGGCACTCATGACCTTCATTTTGGAAGTTAATTAATTCGCTGAAC
ATTTTATGTGATGATTGATTGATTGATTGTACAGTTTGTTTTTCTTAATATCTATTTCGATGACTTCTAT
ATGATATTGCACTAACAAGAAGATATTATAATGCAATTGATACAAGACAAGGAGTTATTTGCTTCTCTTT
TATATGATTCTGACAATCCA

>Negative Sample 201
GATTTAAGGTTTCGATAATCCCACCACCTTCGAGCAATCGCAAGAAGGAAACTCTAGCTGGTAAATGGAC
ACAGAGTTTAGCTAATGAAACTACACATGAAACTATATGGGAAGTAGGTGATTTAGTTAGCAATCCGAAG
AAGAAATATGGCTTTACTAAATTCACCGCAAATTTGAATGAGATAACTGAAATTGAAAAGGGCAATTTAC
CACCTACGGACTCAAGATTAAGACCAGATATTAGAGCTTACGAGGAGGGAAATGTTGATAAGGCGGAAGA
GTGGAAGCTGAAATTGGAAC

>Negative Sample 202
ATGGGAAATGTAACTTGAGACGGGTTTAGCTTGTGCCAAATTATCGTTGTAACCGGCGGTACCAGGAGCA
GTCGAGTTTCCTGACTTCTCGTTGTATGAGTTAGAATTTGTGCTCACAGTAGCAGACCCTTCCAAGGGGC
CTTGATGAGCGTTTTCAAGTTCCGACATTCTTTCTTTACCTCTAATATATTCTTTTTTCTAGAAAAATAA
TATTTTTTGCTGGTTTTAACAAAGCTATGAAGTAAAATTGCAAAGTGAAAGCGAATCTTGTTTGCGTATA
TTATATATAGAAAAATTATT

>Negative Sample 203
GCAATAATGATTGGTAGTTGCCAAACATTTAATACGATCCTCTGTAATATTTCTATGAATAATTATCACA
GCAACGTTCAATTATCTTCAATTCGGCTTCAGTACTGTATGAAATACTCGCTAACATTTTCTTTATTCTA
TAATAGCACAGTGACACTTAAACGTCAAACGTGGCGCACATTAATATACGATGTATGCATTTGGCTTGAC
ACTGCTGTAATAAGGAACATCATCATCCGTCAACTAGTAGTCACATTAGTAGTGTATTATCACATGGTGT
GTAAGAACATTACGTAAAGA

>Negative Sample 204
TAAAGAAAAAAAGCCTTCTGCCAAAGAGGTTAAGAAACAAACTGTAAAAGCCCCAAAGAAACAGACAGCT
TCTCCTTTGTCTTCTTCGACAGAAGAGCCCAAGAAGAAGAAAACTGGTTTCTTCGGCAAATTGAAGAAGC
TATTTAAGTGAATTATCTAAGGATTGGTCTCATGTTGCCGAATCATAGAAATATAAGAAAAAAATTAAAA

AAAAAAAGAAAGAAAGTATAATACTAATATTGGCAATAATGCTAACACATTACTAATAATAGCAATAATA
TTAATAATAATAATAATAAT

>Negative Sample 205
AAAGAACTGGCTACTAAAGCGGAAAAGATATTAGCTAGATTTAATGAACTACCAAATTATGACCTAAAAG
CGGTTTGCACCGGTTGTTTCCATGATGGATTTAATGAGGTTGACATCGAAATATTGAACCAACTAGGTAT
AAAAATTTTTGACAATATTAAAGAAACGGATAAATTGAATTGTATTTTTGCTCCAAAGATTTTGAGAACC
GAGAAATTTTTAAAAAGCTTAAGTTTTGAACCGTTAAAATTCGCATTGAAACCTGAATTTATTATTGATT
TACTGAAACAAATTCATTCT

>Negative Sample 206
GTGGATTCTCGAGAAAGCAGATGGCTTCCGTCGTTATTGAGCAACATCGAAAACAAAACCGTTATTAATG
CTGCTCTGGGGTTTGATAGCTACTTAGTTATGAGGCATGGTAATAGAGATGAACAGTCTTCAAAACAACT
GGGCTGTTATTTTTGCCATGATGTGGTAGCACCAACTGACAGTTTAACTGACAGGACTTTGGATCAAATG
TGCACAGTAACTAGACCCGGCGTTGCTATGATGGCCTCTTCTTTAGCAGTTGAATTGATGACTTCCTTAC
TACAGACCAAATACTCTGGT

>Negative Sample 207
CTATTTATGAATTTGGCGTTTGAGTACTACGAAAATTGTTCAGATATGAAGGATATTTCGAGAAAATACC
TACAAGAATCTTTGTCCACTTTACAAACTATCGACACATTGAGTTTTTCGGAAGAATTAGAAAAAATTAG
AAAGAGAAGATTTGATAAGTTTTGGGCAAAAACTAATCCTGATCCAACAAACAATATTCAAAAGTTTGTT
AATATGAGAACAGGAGTAGCAGGTTTTAATGATTCCTTAATGAATCACTTGTACGGGAAACTAAGTTTCG
GGGTAGCGCCAGTTGAAGAA

>Negative Sample 208
ATCGGTGTTTTGAACTTTTTGATTATTTGTTTCAAGTGGCTTTAGTTAATTATGTACACTTCGTTTCTTT
TTTCTACTCTTAAAAGTGAAAAAAATGAAAAATCAGCGGCATGTATAGCATACAGATAATAGGAAAAAGG
TTGTGAGTTATTCTTCGAGACTATTTCGACTCGTATTCTAAATTAGAATTCAAGTAGGTGGATAATGTGA
CAAAATGAATGAAGGTTAGAGCTTTACACAGAGTATTATCCTCGGCGTTTTCTATCAAAGCTTCTGGTTA
TTCCATACTTTTTTCAAGGA

>Negative Sample 209
CAATGGAAGAATCCAGAATGCCTAAAACTAGTTATTTAAACAAAAATTTTGAATCTGCTCACTATAATAA
CGTACGTCCCTCTTACCCTTTATCTTTAGTCAATGAGATAATGAAATTTCACAAAGGCACACGCAAAAGT
TTGGTTGATATTGGATGTGGCACAGGAAAAGCAACTTTTGTCGTTGAACCCTATTTTAAGGAAGTGATTG
GGATTGATCCTTCTTCTGCTATGCTTTCGATTGCTGAGAAAGAAACAAATGAACGTAGATTAGATAAAAA
GATTAGATTTATTAATGCGC

>Negative Sample 210
TATAGCGGGCATTATGCGTAGATCAGGACTTAAATTTTTCATTGCAGAAGTCCAATTTCAGACTCAGTAT
GGTTTGTTGTAGTGCTGGTGTAAAAGATGGTGTTATTACTTAAGACTGATTTGGTTGCTCAGGTATTTCA
TTCAATAAAATTGTGAAAGAGAACCTGGAATATAGAATGGAAATATATATCCTGCTACTAACCCCAATGG
AAGGTGACGATCACTTCTTGTGCGTTCCAATCCCAGTTTTTGAATGTGCGAGTGGAAAAATTCTAGAGGA
ACAAATTGATATTTTCAAAT

>Negative Sample 211
TATTACCCAAGAAGTTATGGCTTGTGGTAGGCCACAAGGTACAGCCGTCTACAATGTGTGTGAATTTGCT
AACCAATTCGGTGTTCCATGTATGGCTGATGGTGGTGTTCAAAACATTGGTCATATTACCAAAGCTTTGG
CTCTTGGTTCTTCTACTGTTATGATGGGTGGTATGTTGGCCGGTACTACCGAATCACCAGGTGAATATTT
CTATCAAGATGGTAAAAGATTGAAGGCGTATCGTGGTATGGGCTCCATTGACGCCATGCAAAAGACTGGT
ACCAAAGGTAATGCATCTAC

>Negative Sample 212
GCAATAAAGTGTGACAAATTGGTAGCATTGATAGTTATTCCCAATTGACCCATAGGCTGTTGAAATGAAA
CCATCAGTATATGTTGAAACCTCAAGTGAGGCGTCAACCTCTAAGTGAGGCTTCAAGCTTCGCCAACTAT
TATATATGCCACATGACTGCAGGGTCCTTATTTTAAGGAGCTAGCTGCAACGACTTTGTGCTGTAAAGGG
GTGCAGCAGCAAATGTAGCCTATTTGCTCGAGGTTTTCTTTCTTACGAGAATGCTAGCAATTCAAGATA
GTACGTTGTAATATGATGCA

>Negative Sample 213
GCCAAATAGTACGCCTGGTAATATAAGTAGTTTAGAGTTTGGTCAATCTGTGATGACTTTAACTGAGCGG
CAATGTACAGTCTATCGGACCTTAAATTTTTATAGTTCGAGGTAAAATAGACCCCTGTCGTTACAGCCAG
AATAATCAGTGAGCCCAAAGCCACTATACTAACTAGTGCCGTTAGTTGAGTTCGGATGCCTATCCTAAAC
GGAGGAGTGAGTTCCAATTTTGATGGCAGGCCAAATCGCATTTGTATTGTTAAATCAAAACAAAGTAGAT
TATACCCAACGTGTTCTTAT

>Negative Sample 214
TGAGGCAATACGATTCGTCGATACAAAATTTAACCAGATCGCGTAGTCAATTGGGTCATAATGTTAATGA
TTCATCTCTAAAAATTAACATTTCAGAGAACTTATTAGACAGAGATTTTCATGAAGGTATCTCCTACGAG
CAATTGTTTCCAAAGGGATCTGGAATTAACGAGTCTATTAAAAAATCTATCTTAAAATTAAACGATGAAA
TTCAAGAAAGAATAAAAACCATTGAGAAAGACAACATAACATTAGAAAAAGATATCAAAAACTTAAAGCA
TGACATAAATGAGAAAACTC

>Negative Sample 215
AATAACTCCAACCCAGATACCATTAACAACAACTACAATAATGTTAGTGGCAAAAACAATAATAACAATA
ATATTACTAACAATAGTAATAATAATCACAACAACAACAACAACGACAACAACAACAACAATAATAATAA
TAATAATAATAATAATAACAATAACAATAGTGGTAATAGTAGTAATAATAATAACAATAATAACAATAAT
AAAAATAATAATGACTTCGGCATTAAGATTGATAACAATTCACCGTCTTATGAAGGGTTTCCCCAGTTAC
AAATACCGCTTTCACAAGAC

>Negative Sample 216
ATCTCTGCTGGTCGGTACTTAAATTGGAGCTACAGAACAAGAAAGGTTAAATACAACAGATGGATTAAAG
AACAAGAATTACAGCTGATGGAGAAATATAAAGGTGATAAAAACAAGGTAGCAGAACTTATTCACTCTAA
TTCTCATTATGCATTCAATTTAGTGGAGGCAAGGTTGCATCCAGCATTTGTGACACTTCTTCTCAGTAGC
ATTGGCTTTACTGCCTTTGGATGGTGTATTTCGGTGAAAACACCGCTTGCTGCTGTATTATGCACAAGTG
CATTTGCAAGTTTATTTTCA

>Negative Sample 217
TTGAAAATTCTTTCTGTTAATGCACCGGAAACCGAGTTCGACAAGTTCCCATCTTTGTTAGATGTTCAGC
CATTAGACATGTCGAAATATGGGTTTTCTAAAAATGTTCAAGGTATCAAATCACCACTTTATGGTCGCAG
CGACGGTGGTAATGCTGTCGGCGTCGTCGCTTTTGATGTTAGATTAGTCACTGCTGTGAGAATATACCAT
GGCTATGCATTAGATGGTGTGAGGTTTTATTACAAGGAAAAACCAACTGGAACAAAGGATGCTCCCGCAT
CCAAGCCCTCTGTACCACCA

>Negative Sample 218
CTTCATGAAATAAGTAACCCTAAATTTTTATCACAACATTCTAATGTTCTTCATTTTCTGTAAAGAAAGA
GCAGGACTGTGGCAGAATAGTACCATTTTCATCAGCAAATGTGAGGCTATGCCATTATTTGCGAATGCTA
TGAACCTACTGCGTTGCATTCTCCAAGGTTCCCGCTGGGTGGGTCGCCACTAACTCTTCCGTGTGGCCGT
CTATTGCTGATGCATTCAAACCATAGTCACCTGCGATAGGGTTAGTTGTGTAAGAGGTAAAACAGTGACC
AGGTGTTAATTCTTCCCAGT

>Negative Sample 219
AATCTGCCTTAAAGAAATGGGGTGGTAACTGGGAGATCAATGCTGGTGATGGTGCTTTCTACGGTCCAAA
GATTGACATTATGATTTCTGACGCTTTAAGAAGATGGCATCAATGTGCCACCATCCAATTAGATTTCCAA
TTGCCAAACAGGTTCGAATTGGAATTTAAATCTAAAGATCAAGATAGCGAGAGTTACGAAAGACCGGTCA
TGATCCATCGTGCCATTTTAGGGTCTGTTGAAAGAATGACTGCCATTTTGACCGAGCATTTTGCTGGTAA
ATGGCCATTTTGGTTATCAC

>Negative Sample 220
AAACTATGAAGGCGTAGCCTTTACTCTTCTGGGTTATCTTGTCCTTGACTATCCTAATTTTTTCGATCTC
GCCAAACTTAACAAAATACTTTTGCAGTTCAATTTCGTCAAGATCGTATGGTAGCCTCCCAATAAATATC
GTTCTGTAGGGATCTGTGTCCTTGATATGAGGGTCAACGTTAGGATTCCAATTTTGTAGTCTCCGGTCTA
ACAATTGAGCATTTTTGATCTTGGAAAGTTTGATGTCTTCGTATCTTTGGAGATGGTTGTTTGGAGATCC
TTCAGGAAACTCCTCCATAT

>Negative Sample 221
TTTGCTGCTATATATACGACTTGGCTGTAGAGAGATCTACGTAGGATGTGTCCACTATCAAGGGGGGTTT
ACCAACGTTATCTTCTATTTAAGTGCTCAAATGAAGGTTGCATTATATCCCTGGCAGTTTTCCCGGCAA
TTTGCCGGCCGCTTATAATAGTCATGGTGAAAGGATTGTTGCTTGTTGTGGTTGCAACATGACAGTTGC

CTACTAACTAAATAATAATCAAAGTCAGTTTCTCAACAATGGAAAACCTGTTGTGACCTTATCGAAGTAA
CAACATCGTATAATAATTAA

>Negative Sample 222
CCAGAGTCAAGAAGCTTATAAACCCCAGAAATATGGCTCCTCCAACCATTGTGACATCAGTTACGCCGCT
GAGACTTTCAGGTATCACGTGTAATAAAATATCACCCAGGAGTGTTCCTAGAGAAAAGGAAACTAGTAAG
GACAGTGTCAAGCTGGCACGATCGTTCTTGCGCAGTCCAGGAACAAACAGTACGAAAAGACACGGCATAA
GTTGTATGATTAGGATAGCCACAATCGCATTGTAACGGGGTCCCAGGACAAAGAGGTGGTTCTGCAGAAA
GCAAACTAGAGAGTAAAAAA

>Negative Sample 223
CAGCCAAATCTCAAGGTTTGGTTGGTATTGAACTATTATGTGGTATTGTTTTCTTTGATGAAGAATGGAC
ACCTGAAAATGGCTTTGTCACATCTGCTCAAAAATTAAAGAGAAGAGAAATCTTAGCCGCTGTTAAATCA
GAAGTCGAAAGGGTTTACAAAGAAAATTCTTAGAGAGAAGCTTATTACCTTCCCCTTCCATCTATGCACG
GTGTCTCGTACTTCCATAGAGCACCGATTAGTGAATACTAAAACTATGTGCGTGATAAATGGTTCTCTTT
TACAGATGCGGTAAATCAAT

>Negative Sample 224
CAAATTCAATATAGCCGTAGCCTTTTGGTGTTCCCGTATTCCTATCATAAAGAAGTGTTATTCTTTTTAT
CTGGCCACAGTCTTTGAAATGATCTTCTATTTGCTCTGGGGTGACATCCGGAGTGATGTTACCGACAAAG
ATAGAACGCGAGTCAGCTTCAAGTTGATGAGCGTGTTTTTCCTCCTTGCTTAGCTTTTGCGACACCTGTG
GAGTGCCCTCGATAGATAGTTTACCCACAAGTTCATCCAGCTCGAGCTCTTGCTTGTCGTTCTTTTTATT
GTTCGAAATTTCAGTCTTTA

>Negative Sample 225
TGAGAAATTTGAGTGTGGTCCTTCGCAAAGGCATCTTTTCGTTAGAGGCAACGGCCTTACTAAAGAAAGG
TTTACTCACATCAAGCTGAAGATGTACCCCGATGGAGGAATCGCCAGATTTAGATTATACGGAAGAGTTG
TTCCTCCAGAACTCAAAACGAAGGATCATATAATTGACTTGGCTTATGTTTGCAATGGTGCTGTCGCTTT
AAAGTATTCTGACCAACACTTCGGTTCAGTTGATAATTTGCTACTGCCAGGTCGAGGTCATGATATGTCG
GACGGCTGGGAAACCAAAAG

>Negative Sample 226
ATCTTGACAACATACACTCCTATTCCATTTATCACTGTGGTGTTAAGAACCTCATTTCTTTTGAAGACGG
TAGAAACATCCTGTGTGCATATATTTATAATATCAGCCAACTTTTGAACAGGAAACTTAACATAGGAACT
TGCGGAAGACCCTAAAACGGTCAATGAACACATCAATGATGACAACAAAGAGAGGATGGAAATCAATTGC
ATATTGATAGCTTTGCGTTGTCTTCAAATATCCTTATTTTCGAAAGAGACAAAATTTAACAACTGATCAA
CCAATGAAAAAAGAGAACAA

>Negative Sample 227
ACGCTAGATCTTTGGGCTAAAATCTGCGTCAAACAGGAAATATTGCCTATTTTCGTACAAGGTTACTTCC
TAGATGCTATATGTCCCTTTACATAATAAATTAAAAAATTTTTTTTATAAATTATAATAATTTCTTTTTA
TTTCTAATAGTATCTTGGGATTAAATAAATCACTTACAATATTTATTTTATTATATTGCTTTGTTCAATT
AAATTTTAATACGAAATTATTTAATTGTTTTGTATTTAAAATATTTTGAATAATAAAATAATATAGAGTT
TTATTATAAATTTTTCATTG

>Negative Sample 228
CACCACATCCTTGTCCGGGAAAAGAAAAGGCATTCAGGGAAAAATACTCTATAAACACCGGCGCTTATGC
GCTGGTTTTTGTTACAATTCTTCTCGTTATTTTCTTTGTCGCATGGTTTGTATACGATAGAGGTATCAGG
AGAAATGGGGGATTTTCAAGATTTGAAGAAATCAGATTAGGAGACGATGGGCTGATAGAAAACAATAGGA
CCGATAGAGTTGTCAACATCATTGTAAGACTAGGATTATGCATTTCTTTAATCACCAAGTCTGCGTTTCA
ACGCGCGAAGGCAGGTACAG

>Negative Sample 229
TTAACCTGACCTAATATTAACAAAAGTCCAAGTGACGGCTTCTCCATCAAACAAGATAAATTACCAAACA
CAATGGTCGAATCTAAGAATACTGAATTATCGCAAGGAACATGGCTCAATAAGCCAAAATCTGTTTTTCA
AGAGGCAGGAAAAGTCACTCTGGAAACAGATGAAAAAACTGACTTTTGGCGTGAAACATTTTACGGATTT
ACCCGTGATAGCGGTCATTTTTTGGGAGTAGAGACAGGTAGTGCTTTTACTGCACAAGTCCGTGTTCAGG
GAAGCTACGAGAGTCTTTAT

>Negative Sample 230
GATAAAATTGATGCCACGGTTAATTTCCCCATTACAGATTTGGATCTGTCGAGGTACGTTGTCTATAAAG
ATGATCCCAGAGGTTTAATCTATGACCTGTATGCAGTAGATAACCACTATGGTGGTTTGGGTGGTGGGCA
CTATACCGCGTACGTAAAGAATTTTGCCGACAATAAATGGTACTATTTTGATGATTCTCGAGTAACTGAA
ACTGCGCCAGAAAATAGTATAGCTGGATCGGCTTATTTGCTATTTTACATTCGCCGTCATAAAGATGGCA
ATGGATTAGGCAGCTCTAAA

>Negative Sample 231
TTGACATGTGCTGCTGAACATTCTAAATCTTTAAGTAAGGATGTGAAGAAGACAATTTTGGGCGCACCAT
TCTGTCCTAGAAGGAAGGACAAGCTTGTTGAGTACATTTCCAACCAATCACATTTGCGCAGCTTAATAAG
AACAACACAAGCTGTTGATATCATCAATGGTGGTGTTAAAGCTAATGCTCTGCCCGAAACTACCAGATTC
TTGATCAATCACAGAATTAATTTACATTCTTCTGTGGCTGAAGTCTTTGAAAGAAACATAGAATATGCGA
AAAAGATTGCTGAGAAGTAT

>Negative Sample 232
ACAACTCATCATTATTCCTATTGTCACCATTCTGTGCTCAGTTTTCACTATATCTGAAACGTAACTATTT
CCATTCGCCACTAGCGCAAACAAGCCACCGAATGATGGTACGATACACGCAGTAAGAACAATAGCCCATT
TGTGATACTTCATTTTCGATGATAGGGTAAAGACGTGCGTAAGAAGCCCTATGACCCTGATGCCGCTCAT
GTATTTGAAAACACGCACTCTTCCTATTCTATCGGATAATTCTCCCCACTTACCAGCCATGAAAATACTG
ATTGCCCCTGCTATCATCAT

>Negative Sample 233
TTACCAATCTGAAGGTTTGGCAGCATTTTATTATTCTTACCCAACCACCCTAGTAATGAACATCCCATTT
GCAGCATTTAATTTCGTCATATATGAATCATCCACAAAATTTTTAAACCCATCAAATGAGTACAACCCCC
TCATACATTGTCTGTGTGGCAGTATCAGCGGATCGACATGTGCGGCGATCACAACACCTTTAGACTGCAT
AAAGACAGTACTGCAGATAAGGGGCAGTCAAACAGTTTCGTTGGAAATTATGAGAAAGGCGGATACTTTT
AGTAAAGCAGCCAGTGCCAT

>Negative Sample 234
GAGTTGCTATGGTAACAATCTAATGCTTACATCGTATATTAATGTACAACTCGTATACGTTTAAGTGTGA
TTGCGCCTATTGCAGAAGGAATGTTAAACGAGAAGCTCAGACAATACTGAAGCTGTGTTAAAGACCTATT
AGTTGAACATGTTATGGTAGGTACATATATGAGGAATATGAGTCGTCACATCAATGTATAGTAACTACCG
GAATCACTATTATATTGGTCATAATTAATATGACCAATCGGCGTGTGTTTTATATACCTCTCTTATTTAG
TATAAGAAGATCAGTACTCA

>Negative Sample 235
GCACCGTACACGAAAATGAAATTAAAAAAAAAATGTTGGAGCACAAGCAAGGCAGCATCAGAACGTACCG
TCAGTTGGTACCAAGACAAGTCCATCCCCGTAGGCACTACAAATTATGGGAATGAGAAAGTATACAAAAT
ATAAACTGTCTTATCATGAAGAAGAGTCGCTTAGGAATTTAAACTTTCAAATCGCATTACGCAATTCTCT
AAAAACGCGAACCTTTCCCTGATATGTAATCAGGGAATTTCCTCAGAATAATATTCGATCTAAGAAACCG
GAAACGAAATGAAAATTGTT

>Negative Sample 236
GTATCCTAGCATATTTAGAAGACATCACAAAAGTAGTTCATCTGAGTCGTCATTATTAAATTCCCTTTTT
GGTAGTGGAATAGGCGAGGAAGCTCCAACAAAGCCTAATCCACAAGGTCATAGTCTGTCTAGTGAAAATT
TAGCTAAAGGAAAATCTAAACACTATGAAACTAATGTGTCTTCACCTTTAAAACAATCTTCACTACCCAC
TTCGGATGATAAAGGTAATTTATGGAATAAATTCAAAAGAAAGAGCCAAATAGGGGTTCCTAGCCCAAAT
ACGGTAGCTTATGTAACGTC

>Negative Sample 237
AAACGATGATGATTCTAGCTCCTCAGGTTCTTCCACAGAAGACGAGTCTAGTTCATCTTCCTCGTCTTCT
GATGAGGAAACTTCAACATCCAGGAAGGCTAGAAGAGTTGTTGTAAATACACCAAGAGAACCAGTTAGAT
CTTCATCTAAGATTGAAGCCCCCTCTCCATCTGTTAACAAAAAAATCAATGCCACCCCTGATAAAATTCC
AGTTACACAGCTAATGGACATGTCGTCTCCTCCGTCAGTTAAATCCAAGACCACCTCAAATCCGTCTAGT
ATACTTCATGACCTTCCAAG

>Negative Sample 238
TGAATATGCCAAATCCCAGTACAGCTTAAAAGAAGTCATTGTGGGGCGTATATATTTCCTTTTAACGAGA
CTAAGGATAAAACATATGGAATTAAGCTTGATCACAAGAGAATCTTCCGGCCTACAAACTTCTAATGTAA
TGACAGATTCCACTGCTATCCGATACGAAATAATGGATGGGTCTTCAGTGAAAGGTGAAACCATACCCAT

AAGATTATTCTTGAGCGGCTATGACCTGACGCCCAATATGAGCTGCAACTACTTTAACGTCAAGAATTAT
TTGAGCTTGGTTATTATCGA

>Negative Sample 239
TAACAACACGGTGCTGTGAATGCTATCACTTGATTATACGCCAATAAACTTCATATTCTTTGAAGAGCCG
CTGCTATTGCATTTAATGCTAAGGAAGGTTAACAAAGCATTAATGAAGAATAACTACAGGAGTAGCCAAA
AAAATCTCCGCGACGGGGAATTGAACCCCGATCTGGCACGCGACAAGCGCCCATTCTGACCATTAAACTA
TCACGGAAGAAACAAAGCACTCACGATGGGGGTCGAACCCATAATCTTCTGATTAGAAGTCAGACGCGTT
GCCATTACGCCACGCGAGCT

>Negative Sample 240
ACTGTTCAAGTTATTGAATAGCATTATATATTAGTGCCAACCAAAATATGAAACCGATGCTTCCAACTGT
CACCCCAAGAAGACACGCAAAAGGTGCTACTGCACATAAATCGGCCACTCCTTGAGCTAGACCCAATTCT
AACAGACTTACAGTTAGGATTCTCTTTATTCTGTCTAGTCGTTTCTTTTTTTCCCTCGCTTCTTTTTCTT
TTTCGTCTTCTCTACCACCATTTATAGTAATTCGATTCTGTTCTCTACTGAGGTTTTCTAAAGCATTAAC
TACCTTGCCCCTGGAGACTT

>Negative Sample 241
GAAATAGTCATCAGACTTAATAAAAGCCGAAATTCAAGGTCGATAATGTAATAGGATAGGGTGTTGAATG
ACAATGTATAAAAGGAACGAAGACAGAGTAATACTATTATGTAAGAAATACCGATTCCCTTGTGAGTATA
TAAACATAACCATTTCATTAAAATAATTTATTCAATGGTATGTATGATATTATAATAACATACTTATACT
TGTCTCACTCTGTTCATAAACGCAACAATACAATTACTTTTGAATATTATCTTTAATAAATACACTTAGT
AGTTACCAATAAATAATAAA

>Negative Sample 242
TAAATAAGGATAGGTTCCACGAGCTCTGAAGGCAAATTATAATTTGAAGAAAAATGGTTGATTAAATAAA
GACATCTAACGAGGTCTTGATAAGTCAGACTCTTTTTTCCATCAACCTTTTCTGCCTTGTCAAACTTGAT
AATTTTTTCTAAGACCACTTCCATCAGTTCATCAGGAACATTTTCCATTGAGGCCTTGTATTGTGTAAAA
AGTTCCCAAGAGCTTTGTACTCTTCCTGGATTATTCCTAATAATTTGTTTGATGGCGGGAAGTGAAGGTT
TTAAATCAAGATCACGCAAA

>Negative Sample 243
GACTCTGAAAGCCATACCGGTCAACTTACCTTGCAATTCTGGCAAGACCTTACCGACAGCCTTAGCAGCA
CCGGTAGAGGATGGGATGATGTTACCGGAAGCGGTTCTACCACCTCTCCAGTCCTTGTGGGATGGACCGT
CAACAGTCTTTTGGGTGGCGGTCATGGAGTGAACAGTGGTCATCAAACCTTCTTCAATACCGAAAGCATC
GTTGATAACCTTGGCCAATGGAGCCAAACAGTTGGTGGTACAAGAAGCGTTGGAAACAATCTTCAAGTCA
GAAGTGTATTTTTCTTCGTT

>Negative Sample 244
TAGCTTTTCTATATATATTAATCAGGCCCTTATCCTTAGAGAGCTCGTTAAATAGTTTACAATTCAAAAG
ATCTAACTGAAACTTCCAAATGGGTAACAATTCATTCTGTAACACATTAACTAAGATCTTCATCTGTAAA
TCGTTCATCTGTGACTGTTGTTTACTCATAGGGATCTTGTTCAAAAATTTGTAGAGATTAACGAATCCTG
ATAAACAATTTTGCATTTGTAATATAAATTCATCTTTAAAATCGACATTTAATCTGTTTAATTCCTGGAA
AATATACCTGTTCACTCCAT

>Negative Sample 245
GTCATTTAAAATCCAGTTGTTATTTCGATACGTAGTCTCAAATGTTAAAGAAAAGACGTCTTCGAAATTA
TCCTCTGTGTAATTGAGCATTTTTATTAAGTTCCTACTTGTCTCCGGAAACAATTCCGAGTAATCTTCAA
AACTCAGAGGCTCTGAGCATAGTTTTTTATAAAGCGCCTTTGGAAATTGTAAATCTAATATGGTACTGTT
GAAAATCGCCAACCCCATGACAACTCCAAACAAGTAATATAATTCCAGTTGTGAGTTTTTTCCTTTTGAT
TTGTCAAAATTTGGAGGGTC

>Negative Sample 246
AGTGCGGGAAATAGACTATGCAGTGATTTAGTGTGCAAGTTTGCCCACTTCACATTCATACCAAATATTT
CTTCCCACTCATTTTTATCGTATTAGCACTACATACGTGACTTTTTAGCTCAGCTTTCGGACTGGATAGC
TTTACTGACTTACTTTCTACAAGCGATATCATTCGTCACAGTACTCCAAAGAAGTCTGGGCATTTAATTT
TCTTCTTTATCAAGTCCGCCATTATGAAGGAATGTATTGGGTAATAGTTGACATACCGATGTTCTGCTGG
TAGCATCGATGAACGAAAAT

>Negative Sample 247
TGTGGTAAGAAGACTGTTAAGAGAGGTGCAGCTGGTATTTGGACTTGTTCCTGTTGTAAGAAGACCGTTG
CCGGTGGTGCTTATACTGTTTCTACTGCAGCTGCCGCTACTGTTAGATCTACCATCAGAAGATTAAGAGA
AATGGTTGAAGCTTAAACTTCTTTCATTCATTTTCTCTTGGCTTTCACTAGGTATATCTATTCCATAACG
ACTATGTTTTGTATTTGTTAATTTACATAAAACCATATCAGTACATCAACGAACTGTAAAAAAGAAACTT
TAGCATAATTATTGCGGATA

>Negative Sample 248
AATTGTAAATGGATGCGATGACTTCGATGTGACGTTACTAGTCTTACCATTGTAAAAACCACTATCGGTG
CCAAAAGATAAGCGCAATCAACTAAGAAATTTACCACGCTCTTTGTATTGTATTTATCTCCAATTTAATC
TTTCTTTTGGTGTGAAAATTTAGCGAAAATGTCATTGGTGAATTCGTTAACACACTACGAAATTTTAAGA
ATTCCATCGGATGCAACACAAGATGAAATCAAAAAGGCATATAGGAATCGGTTACTAAATACGCACCCCG
ATAAACTTTCTAAAAGCATA

>Negative Sample 249
TCCAATTGTTCCAACCAAGGCGTGAATAGCATAAATCTCTGGTCTTGCATTATGAGGACTCAGATGTGCA
ATCATGGCCTCTTCTATAGATTTGAAAGGCCCGACTTCATCACTTGAAGGCGATATAACACCGAAGATAG
CAAATACTAGCAAGAGGGTGAAATTTTCGCTAAAACTGAAAACCAACCCACTTAACAACATCATTGCACA
ACCATAAACTAGGACTCTTCTTCGGCCCCAGGAATCCGCATACCAAGTAAGAATGTAAGAGCAAATTACA
TCCCCTGCCAGTGTTAATGA

>Negative Sample 250
GGATGGATTTGACCAAAGTAGTTTCATCTGTAAAAACGGTAGTTAAAATGTCCTTAGAGAGTTGATCTTT
GATGGTATTCAATGGATCTTGATTGTTCAATAGATGAGTAGCATCTGTGGCAACAGATTCTAGCCCTTTG
AAGGATGAATGACTATTTTTTGTGCTAGTAGTGTAGAACACTTTATTTTGGGGAGCACTAGAGGAATATT
GCGCCAACAATTGTGGGAGCGTCAAGAGGTCAGAAGCAGTCATTCGCCAATCTTTTGTTTTTCTCTGGTT
CCCGTTCCCTACTGTTACTT

>Negative Sample 251
AGCTCCAGGCTAGGAAACAAGATACTACTGCGAAACTAGCGGAAGAAGTTACGTTAAAGATCTATTAATT
CAATGTTAATTCAAATTTAATATACGTAGGGATGCGAGTTATTACATTGGAAATATAGCAAATCACTGAA
ATACCATCATTTTGAATACATTCGTCATCGCCGCATCAGTTATACGTTTCTGCTATACCGTATAAGAATA
CTTGCTTCTATTCTTAGTTATACTGAATTTTAGACATTAATAAACTACTAACGGTTGCTACTACACTACA
CTATTCTTCTACCAATGTCG

>Negative Sample 252
GCCTTGGCAGCCAAGGTGTGGATCATGGCTTTCAAACCCATGTCGACCTTACCAGATCCAATTAACTCAG
TGACATCCGAAGGATTGGTTGGTTCTAGGATGGCAATGTCTAGACCTTGCTCCTTGAAGTAACCTTTGGT
TTGAGCCAAGAAAATTGGAATATGGTATGGGGTTGGTTGCCAGTTCAACAAAAATGTGATCTTGTCTGTA
GACATAGCTTGGAAGTATGTGTATTGTTGTTGTGGTATTTTTATATAATTGAAAAGGTAAAAGAACGAAA
GAATGGACAAGAATATTGTT

>Negative Sample 253
CTATTTATCTCCAGAAGATTTCCTTGTGAAATTGCCAGAAGGCGTCAGTTATGAAGAGGGCGCTTGTGTC
GAACCCTTATCAGTCGGTGTACACTCTAATAAATTGGCTGGGGTCCGCTTTGGTACCAAAGTTGTTGTAT
TTGGTGCAGGTCCTGTGGGGCTTTTAACTGGCGCAGTCGCCCGCGCTTTTGGTGCCACCGACGTCATTTT
CGTCGATGTATTCGACAACAAGCTACAGAGAGCAAAAGATTTCGGAGCCACAAACACTTTCAATTCTTCC
CAGTTTTCCACCGATAAAGC

>Negative Sample 254
GTTTCCACCAGACGCTAAGTGGTAGCCATAATAGCATCCAGATCGGAAACGCTAAGGACTTGTAAATTTC
ATAACAAAACCAGGTAAAGCTGCTTCTGAAAAGATCTTGAGGTAGAACAATCTTTTGGGATTCGAGCTGT
TTGAAGGATAATACATCTACACTCTTCTCATTTTTAAGTTCATTCTCTTTCATTTTCGGTAGTGAGATGG
CAGTTCGAGGGGTTTTTTATTCGAGATAGTAACTTCTGACTTTTCGCTTTTATACAGCACAGCAGAAAAA
AAAAGCCGCCGAGGCGCGCG

>Negative Sample 255
GATGATCCGGATCCAAATGCCAACGCGGCTAATGGCTCAGATGAAATTGATCAGCTGGTATCATCCCTAC
CAGATCCTTCTACTTTGGCTGGCTTTAAACTGGAGCCTGTAGATTTCGAGAAAGACGATGACACTAATCA
TCATATTGAATTTATAACGGCTTGTTCTAACTGTAGAGCTCAAAACTACTTCATCGAAACTGCTGATCGC

CAAAAGACCAAATTCATAGCTGGCCGTATTATTCCTGCCATCGCAACAACCACTTCTTTGGTTACTGGTT
TGGTCAATTTGGAATTATAT

>Negative Sample 256
CCCCAGGACTAATTGCTCATCAGGGTCCACAATTTCCTGTGATGGGTGTACCACCAAATTTTCCACAACG
CATGATGCCACCGCCACCAGGTCTCGTTCAATTCCAGAAAGATTCTAAAGATGTAAATAAGAAGGAAGAC
AGGCAATTAAGGCAAAATAAAAATCCAAACGGAACAAGAAATAGCAAGGGAAAACAAGAGGAAACAGCAA
CGCCAGATCTGCCTCAACAGCAATACATGCCACCACCCCCACCTCCAGGGTTTTTCCAATGCATCCTAA
CTTTCCTAACGGCCCAATGC

>Negative Sample 257
AGCTACAGCGTTCACTTTGGCATCTCATGTGATTGCGAAGAGAAAACTGTCTGAGAATGAAGCAATGATT
TACACTAACGATGGTGTATACGGGAACATGAATTGTATTTATTCGATCATCAAGAGCCCCATCCAAGAA
CCCTTTATCATAATTTGGAATTTCATTACGACGATTTTGAATCCACTACTGCGGTCCTCGACTCTATCAA
CAAAACAAGATCTGAGTATCCATATAAAGTTTCCATCTGGGGACCCACATGTGATGGTTTGGATTGTATT
GCCAAAGAGTATTACATGAA

>Negative Sample 258
TACTGCCGCTGCCGTTTCTCAAATAACTGATGGTCAAGTTCAAGCTGCCAAGTCTACTGCTGCCGCTGCC
TCTCAGATTTCTGACGGCCAAGTTCAGGCCACTACCTCTACTAAGGCTGCTGCATCCCAAATTACAGATG
GGCAGATACAAGCATCTAAAACTACCAGTGGCGCTAGTCAAGTAAGTGATGGCCAAGTCCAGGCTACTGC
TGAAGTGAAAGACGCTAACGATCCAGTCGATGTTGTTTCCTGTAATAACAATAGTACCTTGTCAATGAGT
TTAAGCAAGGGTATCTTAAC

>Negative Sample 259
TCTTCTGCACGTAATTGCCGTTTTTAGGATTTTTTGGAGTTGTCACGGTATAGATATGTCTTATTGCGTG
ATTTCACGGTGTCTTGCACACAACATTTCCAGGTCACAATATTTGGTTCGAGGTTCCCAGCAAGAATTCA
TTATCCTTTTCAATGTGTCGCATTAAATCATTATCCAAAAACAAAAGAACTTAAGAAGTACAAAAGACAC
TTGAAGTATGCTAGCGTAAGAAATACGGTAACCAATATTTATATGTAACATGCTTATTCTGTCATGATTT
CACAGTTCAAACTACATTCC

>Negative Sample 260
CAAGAAGATATTATCAGTTTCTCCGATATTCGTTCCGTCCTCATCTGCGCATTTAATGGCAAATTGAACA
ACTTCTAAATCTAGCAGGTTGCTGCCTATACGCAAAGACTCAATCATCCATTGAGAAAATTTCACGAAAT
GATTGTAGGTATCGTCAATGGCTAATATGGACTCTGCTGTTAGCGTTAGGTCCTCGTTCAAAGTTTTAGA
TAAAATTTCCTTTTGTAATGACTGCGTTAGATAATAGTTCAGTAAGTCAAACGTTTCAACACATTTAACA
AACATTGAAGCTATTGGTAA

>Negative Sample 261
AATTTGGTCTTGTTCTTCCTGGACTAAACCAATGGTTCTCATAGCCTCTAACGTGCCTTCGTAATCTTTA
CGCATCAATTGATCTGCAGATGTACAGCCAGCCGCAGCAGTGTAAATATATTGCTCAGGCATTTGCACAC
CGAACATCTGTTTATATGTATCAGATGCTCCCTTAGTGAATTGGTAAAAAATATGAAAGTTTCTTTCGTT
CTTAATTTGACCAACAACTCTTTGTTTCTCTAGCAAATAATTTGTGATATTACCTGCGCAAGGTTCAAAT
TGTGAGTTGAATTTAATTTC

>Negative Sample 262
TAGTTCAAGGATAAATGCGAGTTATGAGTTCTAAAGGACACTTTGGTGTCTGTCTTCTGTGTTGGTGGTT
TATACGATATGGAGAAGAGCGTTTGAGATGGATTCACCTCAAAAAGGCGAACACTGTTCGTGATGTAGTT
GTCAATGGGTTTCACAGACATGTTTTCCAGTTCAATTAGGTCTGTTAATTTGCTCGCGGAGGGAAGCTCT
TTTATTTGGGTCAATCGAAGACAGCACAATTGAAACTTTTAGCATTGTTCTCGCGAAAATAGTCAGTTCT
TAACTTTTCAAGAGTGGTGC

>Negative Sample 263
TTCATTGGAATAATCCCGTGCAGCCTTCAGGCGCCGCTAAGGATGGGGATGAAGCAGGTAAACCAGCTCC
CAAAACTCGCATCTTCCTGCCCACGTCTTTCCGTATGGGCGCTTGGCTCAAACACAAATTCTGGTTTGCA
TGTGCACCCCCATATTTGGATTTTGAAAGTTCAACTGAGTCTAGCATTAATACAAGAGCCAATAATAGCA
TTGGTATGGCTGAGGAAGAGAAGCAGGAGCCAGAGAGCAAGAGGTCGATCATATTAAATGAAGAGGCAAA
TCTGAACGATGTATTTGTGG

>Negative Sample 264
CAGATCTATTAAATCAGACAATGCATAAGATACACTAATTTCCTGGAGAGCCATATACTTATAGATATTA
TAAAAATTCGTAATTTTAAGAATTCTGCCATTTGAGGGATGCATATTTAATTTCCTGGGAGAACTGTATA
TTTATGGTTGATCTTTGGGGACAGTTAGCTATTTCTTCTTTCACATTATAGATGTCACATAGACTTCCT
TCCTTTTTACGGAATAAGCCCACTGAAATTTTTAAGGAAGCTCCAGCTCATTATGCTCTCGTTCTTCTGT
TCTGAGTTTCGAGAAATACT

>Negative Sample 265
GGTTGTAGCAGTGTGTTGTCTCTTTTGCAACACTCTTGGCCTACCAGTAACATACCTTGTTGCGTTCCCA
AGAGCTGCTGCTCCCACATTCCCTTTAAGTCGACTGTGTACGGAGAATGATTTAATTGCTGGAGATCTTT
TTACTATGACACTAGTAGATCTAGGTATAACAAAGGCATCATCGTCGTATTCCTCTTCTGTATCTGGGTT
GTAAATTTTTAATTGGAAATCTGTGCCGTCACCTAGTTTGTTCTCTTGAATAATTTCCCTTTTCAAATCA
AATACTGTCAGGCCGGTACC

>Negative Sample 266
CCCTTACCAGATGAGGAATATGAAAGAGGAAAGGTCGACGAGCTGAGCGCCCTGAAGGACTTCCGTGTAA
GAATCCTACCCGTTTTAGGTACTATGCCAAGTTTGTTTGGATTGACCATCACTACGTGGATTTTATCTAA
TATATCCGATAAACCTCTAGAACCTGTTGAGGGTAAGAATAGAATTAAGGTATACGATGGTATATATCAA
TCGTTGGCAGGTCAAATGAGCAGAGTCGGTATACCGAGCCAAAGAATCCCATTGGCTTTGAAGGATGTTA
GTTACCTTGTCGAAGAGGTG

>Negative Sample 267
TATCAATCTAATTAGACCGCTCCCAATAGTCACTGTACTAGTATCAGATGACAAAACTCGTAAGGTTTTA
GTCAGTAATGAGGTAGTTTTCGAGAATTTCCCATGGTTATTTACTTGAGCATAATATGACCAAGTCTGTA
CCAATTGAGCACCAAATCCTCTTTGGAAGAACACTATTAGAGGTTGGTAATCTTTGGAGAGAGTTTCATC
TTCGACTGCACTGATTATACTTTCTAGTGTTTCTAATGTACCGTCTTCGAACTCTTTCCCTTGGGTGTAC
TTCTCCCTTCTCTGGTCGCG

>Negative Sample 268
TACTGCATATACAAGGGGCAGTGACGAGCAGCATTTAAAATGGAGTGGTCTTGTTGAAAGTAAGGTAAGG
CTTCTAGTTATGAAACTGGAGGTGTTAGCTGGAATAAAAATTGCACATCCTTTCACCAAACCCTTTGAAA
GTAGTTATTGTTGTCCAACCGAGGATGACTATGAAATGATTCAAGACAAATACGGTAGTCATAAAACTGA
GACAGCACTGAACGCCCTTAAACTGGTAACAGATGAAAATAAAGAGGAAGAAAGTATTAAAGATGCACCA
AAGGCATATTTAAGCACCAT

>Negative Sample 269
ATAAAATATCTGGAAGAACCAGTGTATTAGCATCAAGAGATAAAACGAATAGTTTTCGGTATAATATCCA
AAGAGATGACGACTTGTTGATACGATTGTTTGATGAACAAAGAAGCCGTTGAATCAGTCTTACTTCTTTG
ACCAATAAATCCGGTATTACTAAAACACTGTTGTCCTGGAAGTATCTCTTGAGTAAATCTTCGTGTACAT
TATATACCGTTTTGTTTTCAGGTGTTGTAAGTAGAAATCCAACAGTCATGTAATAAATATTCTCATTAGA
AATGCCGCCTAATTTACATC

>Negative Sample 270
GTCCGTAAAGAAAAAGTACTTAAAATGGGTAGGTTAGTTATGTAGGGAAAGATGTATGTAAGGATATGGC
TTAACAAAGCTGTTAAATGAAAAGAATATAATCGCTATTTTTTATATTATTCAAATCTTTTTTTGTATTA
TAGTTTAGATTTTGCCTGCGATAGTTTTACAGCGGCGTTATCCAATACAATGACGTTTCTGGTCGTATCA
ATTGTTTGAAAAACGACAACCGAGCCTTGCTTCCAAGCTTTAACCTTTAGAGTATCACCTGGGAAAACAA
CATTGGTAAATCTCACTTTC

>Negative Sample 271
TTACTAGCCTTGTCTGGGTTGTCATCTCTATTCACATGGGGTGGTATCTGTATTTGTCACATTCGTTTCA
GAAAGGCATTGGCCGCCCAAGGAAGAGGCTTGGATGAATTGTCTTTCAAGTCTCCTACCGGTGTTTGGGG
TTCCTACTGGGGGTTATTTATGGTTATTATTATGTTCATTGCCCAATTCTACGTTGCTGTATTCCCCGTG
GGAGATTCTCCAAGTGCGGAAGGTTTCTTCGAAGCTTATCTATCCTTCCCACTTGTTATGGTTATGTACA
TCGGACACAAGATCTATAAG

>Negative Sample 272
GCTGGCGATTTCCATTAGAAGAACGAATGTTTATGAGGAGCAATCGTTGGGACTATACGATAGTGGACAA
GATGACGAAAATATCACCCACGAAGACGATATAAAGGAAACAGACCATGATGGCGAATCCGAAGAGCGAG
ACACTGTATCTACAAAGTCCAAGCCGAAGAAACAGTCCCCAAAATCGTTTGTTGGTGCTCATTTGAGGAG

GCAACTCTCTTTTGATTTATGGTACCTATTCCTTGGATTATTTATAATATGCATATGCGAGGGCAGAAAA
ATCGAAGACGTTAATAAACC

>Negative Sample 273
CTACGGCATTTCTGTTGAAAGTTGCCGGAAAAACCCCAAAAAGCATAAAAAAAAAGAGCGGGGGGAAGAA
AAAAAGATTCCTTTCTATCTTTTCCTTCCCCGGCCTATTCGGGTACTCGCTTCCGGGTCCGCTGTCGTTT
ATAATGATTAGTGCAGAGAGCTTTCTTCTCTTAGTTTTTCATGTGGTAAAGGCAATGCCATTTAGAAGGA
TACTACGTGGGGTTCAAAACCGTGAATACGTTGTCTATCGTGTTATGTATGTTCTGAGTTTCTTTTTGTC
ATTCTACTAGTTTATGGACT

>Negative Sample 274
CGACGCCGGCAAGGAAATATTTCAAAACTTCAATAAGTGAAGCGCAAGATTCTCCTACATCTGCCCCTTC
GCCCGATGGCAATGAGGATCCCACATATCAATACAACGTACAATTTCATTTTCCAGGACCAATAACACCT
ACAACACCCAGGTCTAAGAATGCAGAAATGTTTCCATCCCCCACGCCCCCATTGGTTTCTCCCACAGCGG
TTATCGAGGAAGAGAATGACGATTCCGTACGGGAGTTTTCACGCACGCTAAAATCAAGACTCAACTGCGC
AATGGTTAAGCTATCAAAGG

>Negative Sample 275
CTTATTTAAATTTTGTAAACAAATAAAGAAAAAATTCCTACGAGGTGCGGACTTCAAGTTACATACATTA
CCTACAGAAGCAAATTTAAAGTATGAGCCGGAGCGGATGACAGTTTTGTGTTCCTGTGTCCCTATTCTTT
TGGATGACCAAACTGTCCAATATCTGTATGATGACAGCATTATTCCTGAATTTGAAGCAACATCTTCATA
TGCAACAAAGCAGTCAAAGTGTGGGCGGAAAATGTCTTTGCAAATGGAGCCTGACCTCCTTTTTCAAGAG
GCCATTAGACGGATGCGACA

>Negative Sample 276
TTTGCGCTGACCGATCCTTATACGTGTTTGTCCTCTGATGTTTTCCCATTGATGATGTTTATTTTGATTT
TCATTATCAATGCTTCCATGGCTTATCAGACAGGTACAGCAATGAATTTGGCTCGTGATCTGGGCCCACG
TCTTGCACTATATGCAGTTGGATTTGATCATAAAATGCTTTGGGTGCATCATCATCATTTCTTTTGGGTT
CCCATGGTAGGCCCATTTATTGGTGCGTTAATGGGGGGGTTGGTTTACGATGTCTGTATTTATCAGGGTC
ATGAATCTCCAGTCAACTGG

>Negative Sample 277
GGATATATGTTTGAAGTTTTCCATATCTACACCCATATATAAGGGAAGCACAGAGTATGAGTTCAGTTCC
ATAGGTTCGATAATGTAGTGTTGTGGAGGCAAAGTCATACCATTCTTCTTTATTAAATCGACAAATGCCA
ATAACTGGGGTTCGCATTCCATTAAATTAGATCGGAATCCTAATGCATGCACGTAAATACATAATTCCTT
GCTCTCGCCTGTCTGTTCTGGTGTGAAGTAAGCTGGTTCTTTGGTGATGATTAGTTCTAAATTGAATTTT
TCACTCAAGTCCAATAGTAT

>Negative Sample 278
ATTTATCAATTCTCAACAAGGATCAAAGATCCAAGAATCTACCAAGAATTTGGTCATGGGTGCTGTTAGG
CAACATTTCAGACCAGAATTTTTGAACAGAATTTCTAGTATAGTCATTTTCAACAAGCTATCTAGAAAAG
CTATTCATAAGATCGTGGATATTCGTTTGAAGGAAATTGAAGAGAGATTCGAGCAAAATGATAAACATTA
CAAGTTGAATTTAACTCAAGAGGCCAAGGACTTCTTGGCCAAATATGGTTATTCCGATGATATGGGTGCA
CGTCCACTGAACAGGTTAAT

>Negative Sample 279
ATGATTCTCGCTTTGGGCGACTTCCTGACTAAACAGGAAGACAAAGCATGCGAGAGGCCCTGGGTTCAAT
TCCCAGCTCGCCCCTTTATTTTCTTTTTTTGGACCCAAAGCACTAGCTTAGGCTGTCCATCTTTGTTCTT
GATTCTAAAGATCACAGTAGAATAATGGTTTATTTCTTAGAGTTCTACAAGTTTTAGCGTACTACTGAAC
AATGAATGTCCTGATTTTTTAAAACTCGCGCATATTTGGAGGCCATATCGTGCCACCAACTTTACTACAG
TCAAACACCTGTGCCCAATT

>Negative Sample 280
AAATATTAGCAATCGCAAAAGTATTAACTAAGCTAGAGAACCTTCACTAGAGAAGCTCTACCTAAAGGTA
TAGAACAGGAAAAAGTGTTTTTATTTTGGCGGACTTCGTGGAAGATTGCCTTCCATCAATAATAAGCGTA
GTCCATAGGTACGATCATTTCCTTTTTAACCGTTAAGCAAGCGACAAGATGTATTTTGTTTACCAGCGAA
TGCTCTTATTTATCTTCTGCGCCTTTCCAATAATCTAATTATCAATGCTACGAATGATTATAGTTTTAAC
TAGATGAACGAAATTTCTAG

>Negative Sample 281
CTTTGGTTTTTCTTGGGCTTTATGCAATTTTCTTTCAACTTTACCGTTAACTTTTATAGGCAGGCCTTCA
ACAAGATTTGATGTATCAGACCGATTCTTCCGAGGAACCATTTCGTAATCCTGTTCTTCTTCATCCCAGG
ATGAGCCTTTTGCGCTATATATGGTATTTTCATCCATATCTTCCGGTGCATTTTGGAAAACATTGCCTTC
TAACAAGGAATCTTCGTGCTTTCTTTTCTTTGCAGTCCTTTCCTGAATGCGAAATTGAGATCTATTTCTC
TTAGCCATTATTACTATGAT

>Negative Sample 282
TCTGGCAGCACAGCTGGGACATACTACCTTCGAACATCTCTTTGGCTTTGTTTTATTTTCATTTTTAAAA
TGACCGTTCTTTCGATATAAAAAGAAAACTATAATAACGAGGGAAATTGTTACCGCCCCCAGAATCATAT
CTATGATCTTAATGATGATTGCTTTTGGGTACTTTTCAATCAAATTATCTACTGATGGCACCATAAAACA
ATTTATAAAATAAAGGCTACAAACGGCCTTTGTTAATTTTTCTATAATTAATAAGGAATTAGACTTTGGG
ATCTCATCTTGGCTTTCTGT

>Negative Sample 283
ATACAAAATGTTTGTTGACGGTTTGTTTATAGTGTTTTCAAAACACCACGAAAAGACTTCATTCTTGCAT
TCTTTTTTCTACAAGATGAGTCTATACATCTGAAACTGCGACGCCATTTTTTCCGCTGTTCGGGGCATTC
TTGGCCAAATCCTCGAAAGAAATAAAACGTCAAAAGTAAAAAAGGTGGGCTAGACGAAACGTTATTATAC
GTCATAATTGTAGCCGTGTATGAGTGGGTGGAACTTGTATAAATAAACAAGTTCTTACTTATCATATTCT
CGCATGCAATGAAATTCTCC

>Negative Sample 284
CCGTTCTTTCAAGGAACCAAGATTGGTTATTGTTACCGACCCAAGATTAGACGCTCAGGCCATTAAGGAA
GCTTCTTACGTTAACATTCCAGTCATTGCTTTGACTGATTTGGACTCCCCATCTGAATTTGTTGATGTCG
CCATCCCATGTAACAACAGAGGTAAGCACTCCATCGGTTTAATCTGGTACTTGTTGGCTAGAGAAGTTTT
GAGACTAAGAGGTGCTTTGGTCGACAGAACTCAACCATGGTCCATCATGCCAGATTTGTACTTCTACAGA
AACCCAGAAGAAGTTGAGCA

>Negative Sample 285
AAGTTAAATTTATGGGCAGCAGCCCATTTAGGATTGGAACAAGTGCTATCAACGATGCATCCAAAGAATC
GTTTGAAATCTTGAAAATGAAGCTTTATGACGGAGTTATAGAGGATTCGCTAATTCCTAACGTGAATCCT
ATGGGACAACCCAGAGTGGTTACTAAAGTGATCAATTCTCAAACTGGTGAAGAGAGTTTCAGGGAAAAGA
TGCCATTTTCTGATAAGGAAGAAAGTATAACGAGTAACGAGCTTTTCGAAAAGATGAACAAGTTGGAGGG
GAAAATCATGGCAAATGATA

>Negative Sample 286
AAACGAACTATCCTCGTTGCTATAGTGTCTTCTGCACTTATCGCGTAGACTTTCTATTGGCACTCTGTCA
TGGCATTTAATCTTGTACACATTCTTTAATTCTTCTTGCAAAAGAAGGTACTCTTCATGCAAATGATGAC
TTTGGAGTGAATTGACTAATCCTGAGTCGTTCCAGAAACTTTTTAAGGCGTTCGTTTCATGATTTATTAT
GTCATCCAACCGTCTCATATTAGGTTTCGAATTTGTTACAAATTCTCGCCTTGATAACCTCTCCAGGTCT
TCTTTGTTGCATCCAGGTAC

>Negative Sample 287
AGTAGCCGATGCCAAGTTCGATTGGCACTTTAATGACGCTGATCTGCCTGTCGATACCTGGCGTGTTATG
ATGTACTCAGAAATCCTAGACTTCCATAAGATTGGTGGCAGTGATGGACAGATTGATATATCTGCCACGT
TTGATGACCAAGTTGCTGCAGCCACCGCTGCCGCGGCGCAGGCACAGGCTCAGGCTCAGGCTCAAGTTCA
GTTAAACATGGCTGCGCATTCGCATAATGGCGCTGGCACTACTGGAAATGATCACTCAGATATAGCTGGT
GGAAACAAAGTCAGCGATCA

>Negative Sample 288
TTGACTGTTCAAGAAATCTCTACCATGATTAGATGGGGTTTGAAGCCATACATTTTTGTCTTGAATAACA
ACGGTTACACCATTGAAAAATTGATTCACGGTCCTCATGCCGAATATAATGAAATTCAAGGTTGGGACCA
CTTGGCCTTATTGCCAACTTTTGGTGCTAGAAACTACGAAACCCACAGAGTTGCTACCACTGGTGAATGG
GAAAAGTTGACTCAAGACAAGGACTTCCAAGACAACTCTAAGATTAGAATGATTGAAGTTATGTTGCCAG
TCTTTGATGCTCCACAAAAC

>Negative Sample 289
TATACAGCTGTAAACTTCTTTCCTCGTCTACATCATGGTAACGATTGTTCAATCTTTACTTCGTGTCTTT
TTTTTTTTCTATGTACTTTCTATTCCAACCTATGTGAAGACTAAAATTCACCTTAGTAAACGTAAAGACA
ATGACGATAGGTGCCTAATCCAATGAAAAGAGCACAAACAGTTTCCATATAATTAGGAGCAGTCTACAA

TCAAGCAGTAAATTTTGGTATCATAAACGACCCGCTGAGACCTTTCCATTGGGTCAGGTCGAGAACTGTA
GTTACTCGAAAATCAGATCA

>Negative Sample 290
TCTGGGAATTTCTTCCCAAATTGTATCTCTCAATACGCATCAACCCATGTCAATTAAACACGCTGTATAG
AGACTAGGCAGATCTGACGATCACCTAGCGACTCTCTCCACCGTTTGACGAGGCCATTTACAAAAACATA
ACGAACGACAAGCCTACTCGAATTCGTTTCCAAACTCTTTTCGAACTTGTCTTCAACTGCTTTCGCATGA
AGTACCTCCCAACTACTTTTCCTCACACTTGTACTCCATGACTAAACCCCCCCTCCCATTACAAACTAAA
ATCTTACTTTTATTTTCTTT

>Negative Sample 291
GATATAAATTTGGCACTATTTATAAATTTTTTGAAGAAAAAAAATTCACTTTGATTCTCTAATCACCAATA
GTCAACAGCATTTCCCCAATGACCAGTTTATCAAGACTCTGGTTGATACAACCAATGGTAAGCCGGTCGC
TCATGAGTTGAATCCGAAGTTATATGATATCATTGAACTAACGCAATTGATCATCCCATCAAAGCAGGTT
CTGAGATTTGCCACCCCGTGGGAATCCTCCACGAATTTACACAACAAATTCCAGGATGAGCTAAAGGCTC
TTCACATTGCACAGCTAAAA

>Negative Sample 292
GGTCTTGGACTTGTTATGAAACTTCCGTTTCCCTGTCGAAGTCTTGCTCACTTTCTTGCCCCCAACCTCA
ATCAGTTCAGCATCCTTCTCTCTTTCTTGTCCAGCATTCCCATCATCGGATGTCATGTTATGTTAGATGG
AGATCTATGGATAAACGAAATATAAACCTTAAATGAAAAAAACCGTCGACTTAACTCCTCTATATTATGT
AAAGGCGAAATGTTATTTGTGCCTTTGAAAAGTGGAGGTCTTGGTTGCTTCTCCTTCGACGATAATCGAT
TTCGGTGACTAGTTAGTAGA

>Negative Sample 293
ATTTTTGTCAACCTTTTCTTAGTTTTCACATTGGCAGGTACAGCATCTAATTACTGGGCGTACCTCAGCG
ATACCACCAAAATTGCTTATCAACTTGCTACATCTGTGAAGGAGTTCTCCTTATTCTATGTCGATTTGAT
TATATTGCAAGGTATTGGTATGTTCCCGTTTAAGTTGTTATTAGTTGGTAGTTTGATCGGCTTTCCTCTA
GTGAAAATCAAGGCTAAGACACCTAGGCAACGGAATGAACTTTACAATCCACCGATATTTAACTTTGGAC
TACAATTACCACAGCCAATT

>Negative Sample 294
ATCGTTAGCGTTCTTTCTACTTGTAAATATATACGTAAAATTATATATAACAATTTTATATGTGTCATTA
AACGGAAGACGTTGTTGCTATCACAGTGGATTGTGTTATTTGGGTATTTGGAATATTACAGTGACTTCTT
GCAGCGTTGTTAGTTTTTGCCATGTTCTGCAAGTACAAGGTTGAAGATGTGGTCGAGACTTTGATAGTAT
CCTTAAACAACTTCATTTTTTTATTTTGACTGGGTAACGACCTGGTGTTCCTTCTAGTCTCACCGCTAGT
GGCTTCTTCTTTGCTGACTC

>Negative Sample 295
GTGCTTCCAAATCTAATTCACTGATTACATCAACTGATCCTGTCGAAGATCACATCAGTAAGTACTCTTC
ATCAGGCACCCCTGAAAATATTACAGGTGAGGCAGACGACGAAGACGAAGACATAATCAGAAACTCTTAC
GGGCAAATGATCAAAAACAATTCCAACAGGCCACATTTGGCGAAGGGTGAATCGTACCAGTCCGCAGAAC
AGGAAATAGATCATACGGCGCCTGAAAAATCAGAGAAAAGACAAGAAAGAAGCGGTAGATCCTTTGATAG
ACAAAAATCTTCGGCTGAGT

>Negative Sample 296
GTCTGAAATATCTTAGATAAGTTAATCTCCAGTAGCTTTGTTATAGATTCAAGCAAGTTTTCCATTTGAT
TACAGTCTCTTTTAAACAGCTCGACGTTTGCACTATCATTCTTACTGTTTTTGGCAGAACGGTTTAGATT
GGTAGACTGCGAGGAGACCTTATGTAACTCATCTTGTAAAAACGTCAAAAGTACCTGAATAAGGTAGCCA
TACTCTTCTAAATGCCTTTTCCACTGCGGTATCAGGCCACCAATTTCAGTAAAGTTAACATTTGATGAGA
GATTAGCCTTAATATCTTTT

>Negative Sample 297
GATAAAATGAAGAATGACTACTTGGCACCTGCTTACGAATACTTGAGAAACAACATCAAGAGTGACCAAG
TTATCATCATCCATGACGCTTTCCAACCATACAATTATTGGGATGACTTCATGACTGAAAACGATGGCTA
CTGGGGTGTCACTATCGACCATCATCACTACCAAGTCTTTGCTTCTGATCAATTGGAAAGATCCATTGAT
GAACATATTAAAGTAGCTTGTGAATGGGGTACCGGAGTTTTGAATGAATCCCACTGGACTGTTTGTGGTG
AGTTTGCTGCCGCTTTGACT

>Negative Sample 298
TCTATTCAGCCTAATTTATCGGCAGCAAGAATTCGTTTGAAAAGGGACCTTGATTCGCTGGACCTCCCAC
CAACAGTGACTTTGAATGTAATAACATCACCAGATAGTGCGGATAGATCACAATCGCCGAAATTGGAGGT
AATTGTTAGGCCTGATGAAGGATATTATAATTATGGATCTATTAATTTCAATTTGGATTTTAACGAAGTT
TATCCGATTGAACCACCAAAAGTTGTATGTTTGAAGAAGATCTTTCATCCAAATATCGATTTAAAGGGGA
ACGTTTGCCTAAATATTCTT

>Negative Sample 299
ACTCAACATATATAATTGTCAATACTTTAGGAAGCTACTATTTAAGATCAGGTACGTAGAAGCAGACGAA
CAGATTCGAGTTTCGATCTCCTTTCTGTATTGTATTGGTTAGGTTACATCTCGGTGGAAGGCTGTCGTAT
CTCAGAATGAGATACGTCAGTATGACAATACATCATCCTAAAAGTTCATAAAACACACATGAAACAACCT
TATAAGAAAACGAACAGAGTGAGTAACATGAGATAAAACTCCGCCTTCCTTGGCTGACCCACCCAAACAT
ATAAATGCCTGAACAATTAA

>Negative Sample 300
TAATTGTGAAAGATAACATACATGGTTATTTGGTCAACAAAATAGTATTTTTCTTAATGAATTTGAGGCA
GAAAAAGGGGTGTGTATATTTTGCTCGCTGTGGCACCAGCGATAAAGACAACTACATACATGATGAGGAA
CTAAACGAGGAGGGAATCCATTACTCTCAAGTTCTCAAGGATTTTGTCCTCCAACGAATTAAACAGAAGA
GACAGGCCAAAAAAAATTCGGATTCCTTAGTCGAAGTAATAGATGGAAGTCATGATGAAGATTTGAAGAC
TTCCTTGATAGTTTGGACAG

>Negative Sample 301
AGATACACAAGAGGAGATTAAGCGTGAATTGGATAAAGTCTCGAGAATGATTCAAAAAGCTGAAAAAAGC
CTAGGATTATCGCAAGAGGAAGTCATCGCATTATTTGAGAAGTGTAGGAATAAATATAAAGAAGGTCAAA
AGAAGTACATGGAGATAGACGAAGCACTGAACAGGTTACATAATTCCCTCAAAGCTCGTGATCAAAACTA
TAAAAATGCGGAAAAAGGGACTTGTTTTGATGCAGATATGGATTTTCGCGCATCATTGAAAGTAAGGAAG
TTCTCGGGAAACCTTTCCTT

>Negative Sample 302
TGAAAACCCTGACGGTACATTTAGCGTAATAGATCCAGATTCGACAGATTCTTTTGGTGATGGAATGGGC
TCTGCAAAGGATAAACCATACCGTTGTGAAGTTTGTGGTAAGAGATATAAGAACTTGAACGGTTTAAAAT
ATCACAGGGGCCACTCCACTCACTAAGATATAATTGTTTCTTTACTGAATTGATTTAGTATGCTATTTAG
ATACTAATTCTACCGTTACCGACTCATGCATCGAGCGGCCGCTCTTTCTCTTGTTGTTGGGCGCAATTTA
TGTGATCATCTTTGAACGAA

>Negative Sample 303
GTTATGGAATTGGTATACCATTGACACCTGCTTCATTGCCAGATCTTGGAGAAATGACACCAAAGGAAAA
TTTGCTGGTTCTTGCATCGGCTGTTTTGCGCTTGTGGTGGTAGCTCAATGGCTTACTCGTTTTTCAAGGC
AATTTGACGTAGAGCTATTAAAAAGACAGAAAATAAAGCATTTAGCTAGTTACTCGCCAGAGGAGTACGT
TGTCAAATGTGGAGAAGAGGATGCTAAATCTGATATAGAAGAACTACAAGGCTTTTACAACGAACCAAGC
TGGAAAACAACTTTAATTTC

>Negative Sample 304
GTAGTAGACCTTTTCAAAAATGAAAAAGTTGTTGACGCCGCTTTAATCGAAGAAACGTTACTTTATGCAA
TGATAGATGAATTTGAAACTAATGTTGAAGACGACTCGGCTTTACCGATTGCCGTGGAGGTCATCAACAT
ATATAACGACTGTTTCAATTTAAATTATAATAAGGTAGAAAAATTGTATTTGGAATGGCAAGAAAAGCAG
AGAACTAAAAAATCAAAAAGAGTTGTGCATATTGAGGGTGATGATGATGAAGACGATGAAGATGTAGAGG
ACTATGATGACGAAGATGAA

>Negative Sample 305
CGTAAGCACAACGTCCTATTGATCGTTGACGAAATTCAAACCGGTATCGGTAGAACCGGTGAGTTGCTTT
GCTACGACCACTACAAGGCAGAGGCCAAGCCTGATATTGTTTTGTTAGGTAAGGCTCTCTCAGGTGGTGT
TCTTCCCGTCTCATGTGTTCTGTCTTCTCACGACATCATGTCTTGCTTTACCCCAGGATCTCACGGTTCT
ACTTTCGGCGGTAATCCTTTGGCTTCCCGCGTTGCCATCGCCGCCCTCGAGGTCATCCGCGACGAGAAGC
TGTGCCAAAGAGCCGCCCAA

>Negative Sample 306
TCGCTAGTTGTTTTCATTGGGGGCCTGATAAGTAGCATAAGCCTTGCCTGGGTTAACTACACTATTCAGT
CTAGAATTAATACAGAATTAGAGGCCTTCGGGTTTTCTTACCATCTTGGAGTTACATGGTTTGCGCTTCT
GTGGTGTTTTGCAGGTTTGATTTCTGTGTCATGCTTAGCTTGGTCTGGCTTAGAATGGTGTATTTCCGAT

AATGGCACGTCCTATGGAGGTGGAATCGATGATAAATTCTTAGGCTACCAAGCCGGTGTATTTACTGATG
CAGATCTAGACGATGAAACT

>Negative Sample 307
TAGAATAGCACGGCATAAATATATATTTTGTGCTGTCTTTTTGGGAAGGGTAGTTTGACAACCCATGTAC
ATTAACATAAAAAGAGCTTTATCATTAACGGACGAAGAAAGAATGTGTATCGCGTATTTTTAGACTATCG
ATAATCTTTTTTAAAGTTATGGTAGATAGAAGATGGAATCAGTTACGAAGTGATGAGTTCCCATACATTC
CAGCCTGTTTACATTAGCTAATTTAAAAAAAAAAAACATAATTAGAATATTACGTGATGACTCAGTTTAA
ACGGTACTCTGTAACACATC

>Negative Sample 308
TTTGTATGGTACAAGAAGTAGCACTAGTGTGGCACCTAAGTTGACGAATTCTCCCACGAAATCAAAAACA
AATTTCAACATCAAGAAGTGCATCTTACCTAGGTCCGTCGTTACCACATATAAATTACCATCTCCAGTGC
ACGAAACGATAGACGATATATCCAAAAAAATCATAATCCTGCTAATTTCGTTGAAGTTTGAGAAAAACTA
TCACTTCTTGCAACCTATTCAATTGTCCACAAATAGTAAGACACGGATCTCAAAATCACTAGACGAATTA
TGCGGAGTGCAGCTGACATC

>Negative Sample 309
TGTTTTTAATTTGGGGTATTATTGCCGCCTTACTGGTAGGATTTACTATCAAGAATGTGTCTCCATCAGT
GTTTTTGTTCTTTTCTATGGTAGCATTCAATGTGGGCTCAATAATGGCAAGTGTTACACCGGTTCACGAG
ACATACTTTCGTACTCAGTTAGGAACGATGATAATTTTAAGTTTTGGGATGGATCTTTCATTTCCTGCTT
CTTCCATTATCTTTAGTGATAATTTACCGATGGAGTACCAAGGCATGGCTGGGTCATTGGTGAATACTGT
TGTCAATTACTCCATGTCCT

>Negative Sample 310
AAGTCTGATAATTCGATTCCTATCAAATTCTGCACTGAACTGTTGATGAATGAGAATAGATTAAACAAAC
TAAAGACAAAATTTTTGACGTATCAGAAAAAAGTTCATGAATATCGTAAAAAATTTGAAGAAAACCATGC
GAAGCTAAACGAGCTTTATAATAGAAATAGAGATCATTTCACTCCAAAAGAGCTTTTGTTCACAAATTTC
ATATCTGATCAAATAAACAATGATATTGACAGTTTAGCTGGATTAAAAGTTAACATTATTGATTTACATG
ATATCTTCAAAAAGCAAATA

>Negative Sample 311
GTGCATACGTTAGTCTGAACGGTGTATGGTACAACATTGTTGATCAAGTCGATTGGCGTAACGATGTAGC
CAAAATTCTCGAGGACAAAGTGGAGAGATTACCTGGCTCGTACTACAAGATAAATGAGTCCATGATCAAG
TTCCACACTGAAAATGCGGAAGATCAAGATCGTGTAGCTAGTGTTATCGGTGATGCCATCACACATATCA
ATACTGTTTTTGACCACAGAGGTATTCATGCCTACGTTTACAAAAACGTTGTTTCCGTACAACAAGTGGG
ACTTTCCTTATCGGCAGCTC

>Negative Sample 312
CCACTAAAGGTAAAAACACAGTAAGCAATAAGTGGAATGAAACTTTGAACACGGAGCTGCAATATTATGA
TGAAGATGAAGACCTTAGAAGAAAAAGACAGTCAGAAATATCAAGATTGATCGATGCGTATGGTGGAGAC
CTTGAACTTTCAGGCGACACCGATGAAGAAAACGACATTCAGTTGAGAATTGCACTCTTAGAATCACAGG
AGGCGCAAGCACGGAATCAAGCTGAAGCGGGGGAGCCTGTCGGAGATGATGAAGATGAGCAGTTAAGAAG
GGCGCTGGAGGAATCTCAAC

>Negative Sample 313
CGCTTTCTTCTTTTTAACTAAGCCAACTTTGTACATACCATTCAGTGATGTTAGCATGGTAAACATTTCA
AGAGCAGGACAAACTTCTACGTCATCGAGGACGTTTGATTTGGAAGTGGTACTGCGTTCAAATAGAGGTT
CTACCACTTTTGCCAACATCAGTAAGGAAGAGCAGCAATTATTGGAACAATTCCTAAAGTCTAAAAACCT
AAGGGTGAAGAATGAAGATAGAGAGGTACAAGAAAGGTTACAAACCGCTTTAGGTTCAGACAGTGACGAA
GAGGATATTAATATGGGTTC

>Negative Sample 314
TCATTGAACGCATGCAAAAACTAGAGCCAAGCCTTTCTATTCTTTTCTTGTTTATTTTATATTCGTTAGA
GTTTTGTGGTGTTTCTTTAACTTAATAAACTTTCGAGAAAAGTGTCAAGTTTCCCAGATTTTTAAGATCC
TTATAACCTGTAAAAAAAAAAAAAAAAAAAAAAAAAAGAAGGGTTTAAATAAAAATCGGACTTACTCAAAGG
GTTGAAAAGCACTTTAATATAGGTTTTTAGTTTCGGGTAAGAAGATGTGTCAAAGGTCTCGAAAAGGAAA
CATTAGGGCAAATACGTAAA

>Negative Sample 315
ATCGGCATAACGACAGAAAGACCGCAGATATCCAACAGTATAATTGAGTGCAATTCAGGAACTTTGATTC
AAAAGCCCATCAGTTTGCCTAATTTTAGAAACTTGGAGAAAATTAATCAGGTCAAGATTAATGATCTATA
CACTGAAGAATTTCTTAAGGAAGGTAAATGAAATTAATCATAAATCATAAATTATATTTAAAATAATAAT
TTACTCGGCCGGACCCCGAAGGAGTACCCAAATATTTACTTGTTTATAATTTATTATTGAAAATGTTTTA
TTCTATATTGGTAGAGTTAA

>Negative Sample 316
TTATTTTTGGGTATGTCGTCTTTTGGAAAGCTTAAAAAGTACAAGCTAAATTTTGCTTCTTCAAAATCCT
TTCTGCTTAATAGCTTCATACCGAAGTGTTCGGTGTAGAACTTAACGGTCCTTGCTGGATCCTTGACTCT
TAAACATGTGTGATTAAGCAGAAGGGTTGGATCATTCGAGGCTTTCTCAATCTGAATTGGATAGCGTGTA
CTATCAGTGGACATTGTTAGTTTGTAGCTGGTGGCGTATCTGGTCTGAAGTGTCAAAAAAAAGGCATGGA
AATGTTATTCCGATTTTTTT

>Negative Sample 317
ATGTACGAATTAGATCTTTTTAATACCTTACAAAAGGATGTCGAAAAGGCCTTGAAACAGGATAGTAATG
ATACGTCAGATTCACCTCAAGATGACCAAGTGGGTAAATCTCAGGCACAGGCGGTAACGCTATAGAAATT
TTCAGGTTTGCAAATAAAAAAAGTTGATAGAGAGGTTTTTTTATATTTTAGATATATGCATTCATAATTC
GATATATACATGCATGCATATCTCGAGTACAATATTACCATTGTTGTAATTACTTGTTAAATATCGCCTC
TTCTATTCATGTTTGAAAGG

>Negative Sample 318
GTCACTCTCACTTGCTTCCGCATCGATCAAACCGTCAACGGATAAAAGCTTGTCCTCATCTTCCTCGACA
TCAGATTCTTCGCTAGCTGCTCTCTTTTTCGACATCTTTGCCTCGGTAGTTTTGTTGTTCTTAGCCATCA
TTGACCTAGTTATGTTAGAACAGATTTAATACAGTACCTTTCTTCGCTAGGATCTATATGCGAATATATC
ACATATGTAAATTATAAGCTCATCGCAAAACCAAAAAAAAAAAAATTTTCAATAATTTTTCACTAATCTT
CAAAAACAAATGGGGTAACC

>Negative Sample 319
AGATCGTCTCACGTACATCGTATCTGCTCGAGTGAAATCACTGTATGGTAAAAATGTATTATGCATCTCT
TGTTAGCATTAAATGAGCGTAAAGCCGCATTTGTGTAGGTGGACTATCATTGATAAGGAATTCAACTTGA
TTTGAGTCATATTCTTCCGAATGATTTCTGGTTTAGTTTTAAGTTTTCCCTTTTGCCCCGCTAGGATCTA
GTTTGTCTATTCAAATTACAGTGCATGAAAGTTAACTTCGAATTGATAGGGATGCGTCGCTACGCTCACG
GGACAGCTCATAAACACAAG

>Negative Sample 320
GGCAAAGACAAGGAAAGAATTAATTTAAGGAAGAAAATATCTGATAACTTTGAAAGGCGCTGGAGCTACG
ATAAAATGAAAAAGGAATTAACAAATATTGTTTACAAAAACAAGGTACTAACAAATGTTGTAAACATCTT
CCCCTTAGTCGAAAAATATACAGTGAGCGCAGAAAATACACATGATGTTATATATTGGAGTTCTGTCATC
ATGAGAAATTCATGTCGAAAAAATGAGATCTTAGGTGTGCGTCAGTGTGCCAATTTTTCGTGCGGAAAAT
GGGAAGATTTTCCAAGACAA

>Negative Sample 321
TCTGAATATTTCTGAGCTCCACTTATCAGCTATCACAGTCGCATTGAGTGTGATTAATTTTTCGGTATAT
TGATACTATTTCATTTTTATTTGCCATTATCTAAAATCTTCGTCAATTTTTTTATGAGCAACGTATCCCT
AATCCCTTTTTTACAAAAGTCCAAAATAGAAAAAAAACTATTACGGATGCAGTGATCTCTAATTAGGGAT
ACTTTTCCTTGTAATTTGGTTTATCGTACGGTAAGAATGACCGAATGAGTGATGGTTTTGATCCGAACCG
TAAAGAAATACTGGTAGAAG

>Negative Sample 322
TTCTTCTTTTGGTTTTTTTTCGCTACTTTCCTTAGCTTTTCTTCTTCAATAAGATTTGATCTATTCGACT
CAATATAGTTTACAAAAAAATCACCATTTTTGGTCTTCTTTGCTTCGTTAAGTGTAGCTTCCACATTATT
AAATAGTTCTACCCTTGTCTTGGCCAGTCTGTTTAGTATTTTGTTATCTCGTGCTCCGCATTCCATTTTT
CTACATTGTCTTAGAGACTTAAATGATACTTCACCGGGATTCATATACCTGTTTTTTCTCAAGTTATGCC
ATGGTGTTATCAAGATGGTG

>Negative Sample 323
TCTTACTTCAAATGAAAATAATATTCAGTCGAGGCATGAAGTGGAAGATAATCTCCAGAAACAGCAATTT
GAAAGGATGAAGTGTCAGTTTCTATTGGAACGTCAAAGTTTGAAGGATCAATTGCGAAAGCGAGAGAATA
AAATTGTTAAATATAAGCAGAAAATTATTGAAAAGAACAAGAAATTAAACAATCTAGCAAAAGTACTTAA

TCAACATGCAATATCTGATACCTCTCAAATAGATAGTTTCAGTAGCTCGGTAAAGAAAACGCCTTCGTCG
ACTACAACGCCCCAAGAAAT

>Negative Sample 324
TTCTGTATTTACTTGCATGTTACGTTGAGTCTCATTGGAGGTTTGCATCATATGTTTAGGTTTTTTTGGA
AACGTGGACGGCTCATAGTGATTGGTAAATGGGAGTTACGAATAAACGTATCTTAAAGGGAGCGGTATGT
AAAATGGATAGATGATCATGAATACAGTACGAGGTGTAAAGAATGATGGGACTGAGAGGGCAATTATCAT
CCCTCAGAATCAACATCACAAACATATATAAAGCTCCCAATTCTGCCCCAAAGTTTTGTCCCTAGGCATT
TTTAATCTTTGTATCTGTGC

>Negative Sample 325
TGAAGGAATTCGAGGGTAAAACTTTGGTTGACATTACTAAAGATTTCGAATTGGAAGAAACAGACGAAGA
AAAAGCTGAAAGAGAGAAGGAGATCAAAGAATACGAACCATTGACCAAGGCCTTGAAGGATATCTTGGGT
GACCAAGTGGAGAAGGTTGTTGTTTCTTACAAATTGCTAGATGCTCCAGCTGCCATCAGAACTGGTCAAT
TCGGCTGGTCTGCTAACATGGAAAGAATCATGAAGGCTCAAGCCTTGAGAGACTCTTCCATGTCCTCCTA
CATGTCTTCCAAGAAGACTT

>Negative Sample 326
AATGAATGTATGGATAAATTACTACAAGACTCTATGATGAAAGTAGCAATAACCCCAAAGACTATGAAGA
AATATTCTTCTGAATATGAGGAAATTCACAGATTGGACAATGAAAAAGAAGTGCAATATAAGTCTATCAC
GGAAAAAAATTTACATTTACAAAAGCATGTTCGTAAATTAGAAAACGACTACACATCTTTAAATAGAGAG
CATGTGACAATTGCGAACGAGTTAGTGAAAAATCGGCTTAATATCGAGTCTGTATTAAATGAAAATAATG
GTTACAAACTTCAAATTTTA

>Negative Sample 327
AGGAAAAGATTTCCGAATAGAAAGTTTTATATGATGGGATTTTCGTTAGGCGCATCTATAATGACAAATT
ACTTGGGAGAAGAGTCAGATCGTACTAAAATCGAATGTGCTATTTCCGTGAGTAATCCATTTGACCTGTA
CAACTCTGCATATTTTATTAACAGTACACCAATGGGGTCACGATTTTATTCACCTGCTTTGGGTCACAAC
TTACTACGCATGGTTCGAAACCATCTCTCTACTCTGGAAGAAAACCCTGATTTCAAAGATGTTATCGAGA
AGCATTTAAAAAAGATTCGC

>Negative Sample 328
AGCCAAACAGTGATAAAGACTATTGTTATGATGGGAAGGGAACCGTAATAGCGAAAAACAACGCTAACAG
CGGTGACGTTGCATTTTGCCAGACCGTGCTTCCGGGCAACGAAGCTATGCTGATCCCAACCTTAGTCGGC
TCTGGGTCAAAGCAAACGCTGGCTGTGCCTGGTACAGACTACTGGGCCTCCAGCGCGTCGCATTACTACG
TAAATGCTCCCGGTGTAAGCGTAGAGGATGCATGCCAGTGGGGTAGTAGTGCAAATCCACAGGGGAACTG
GGCCCCATTTGTAGCTGGTT

>Negative Sample 329
AATTGTTAGCAGTATCCATGAAAGCATTGAGAACGATTCCGCCCCTAAGTCAGTTAAAGACTATTGGGAA
CACCCCCAGGCTTACAAACCAGGTGTACCGCTGGTAGCCTTCAAATTGTCCAAGAAATTCCACGAAGAAT
ATCCAGAAGTGCCACAAGCAATCCTTTCCTCTTTACTGAGAGGTAGGGGTTGGATAATACCAAATTACCC
ACTACCAAAGGCAACGGATGGATCCGATGAGAAGGAGGTATTAAGAGTGGTTTTCAGATCGGAGATGAAG
TTGGATTTAGCACAGTTGTT

>Negative Sample 330
GTGGTTTGAAAAGACCCATACTGGTAAAGCACAAGAGAGATAAGAAGGGATTGGGTAATGCTCCTGGCGG
GAATGATGGCGAAGCATGGTGGGAAAGGCTATTTGATGGACATCTGAAGAACCTGGATGTAAGCACTGAT
TCGAATAATGGCAGTATTAAATTTACTCCAAAATGAGGCAGTTGCTACTGCTGTATCGAAAAGTAGCTCA
CCTCTATACAGGTGGTTTGTAAAGGGAGAAGGGCTGAAAGGAACCATTACTAATCTTGGTAAAAAGGAGG
AAGCCAGCTTTGTTGTATCT

>Negative Sample 331
CAAAAAAAAGCGATGGTTGCCAAAATAACTGTTGATAGCTTAGTTGCGAATGCGGTATTAGGTTTAAGTA
CGTAGTCAAAAGCATTGACGTGTAAAATATTTCCTCAGTTATTGGTGCAAATATAAAATTCCTGAAACTC
CAAATATTCAGGAATTCATGGTAAAAATCTTCAAGTATAGAGCTCTTTGGATTTAATAAATGATATAATA
CAAAATCTAAAACGGGTCCACAATATAAGGTCAATAACATCGCAACACATTTCGTTAAGTCTTTCACGAA
CTGGCTGAATTGCCAAGGGT

>Negative Sample 332
CATTTCAATATGATCTTTCATATCTCTATCTTCACAAAAATGCTTTAGAAGCTCTTGGTATTTAAGCCTT
TGTGCTTCCTCTCGTTTCTCTTCGGTATGGTCTTCGCATATATAGAATAGTGAGCTATCATTAAAATAGC
AAAACGATCTTAACGTTTGCAAAATGAAGTCAATATGTACTGACTGCCATACTGTAGTGTGTCTTTGTAA
GTTTAATATTTCGCCACTGTTTAAGCACCATACAGAAAATTCATTATCGTCATTCAGTTGCTTAATTTTA
TTGAATGCGTAGGATAGTTC

>Negative Sample 333
ATTTCTTGAAAAAGTGCAACACAAGATCGGCTTTACCGATGAAGAAAAGGAGAACATCTTACTCTGGACC
AACACAAATTTCCAGTTTCAGGGCTTATTATCGGACCAAAACACCTTCAAAGATGTGAGTAAACATTCTT
TACTCTTTGGTAGAATTTTACCCGAGGAGTCTAAATTGTTCAAAGAATTAAATCGTCTAGAAAACGTACA
AACATCTTCATTGGAGGATTTTATGGATGATGAAAACGCGACTGACAGACCAATGGACGATGAGCAAGAT
CTAGGTATGGCGATAGAACA

>Negative Sample 334
CTACTCTGTAGTATTCCCCGTGAGGGTCCACAGCACCTTGGTAGGGGGTCTTGGGCTCGTCTATGTGAAT
ATCTTGGAACTGCTTTTTTGTTATTTCATTTTCTGCTAAATTTCTTTGGTTCCATTGAACTCGCTCTTCT
GGTGTCATCTTGGCCAACATGTCTTGCTCATGTTTCAGTGACAGTGTGTCCTTTGGAAGGAAGGTACTCG
ATGTTCCAATTATCTCCCCCTCCTCTTTTGTATTATCTAGTCCCGGTATATTCCTTTTATGTGATGTCAA
TTTGGCATTTTTCTGCGTAT

>Negative Sample 335
CGCGTATAATGTTGTGTACATTAAGAATTGAATAAAAAATTTTTGATTCCTAAAACCAGTGCATTCAGCG
AACCAAGGGCAATGATGATCCATTTTCAATATGCAAACATCGCATGATGAACAATGATGACAGCGATCGG
GCTTCCAAACATGGCATACTTGGCACACCCGAAATCTTCCATCATGTTTTAAGGTCAAACACCTTTTTGA
CATATACTCCGGAGGAAGTTCAAGTCCATTTTCCGCTGCCTTTAAATCGTGGACTAACAAATCAGGGAAA
TCTAGAGGAGAACCGGGACC

>Negative Sample 336
GTACCAAAACGCAATCATTCCATCACTGTACAAGAAGACTAATGCGGTGATATTAACTTACGATATAACA
AATGCGAAGTCCTTCCAAAGCTGTATGGAGCGGTGGATAGTTCAGGCGTTGGAAAACTTTTCTTCCCAGG
ATTTGTTAAAAGCAAGATTTTTTCTGGTGGGTAACAAGATTGACCTTTATAAGGAAAGGCAGGTAACCCA
TTATGATGTGGTGCAAATGGTTCAGGAAATGCAACTGAAACATGGTATTAAAATTTCGGGAAACTTTGAA
GTAAGCTGTAAATGGGTCAA

>Negative Sample 337
AACTTCCATTCGGGTGATACCTGCAATATGAGGAAGAAAAAAGATAAATTTTTTCTTCTTAAAGTATAAA
AGGCGGACATATTTGATTTAAATGCAGAAGTTTTCTCTCAGTGGTTTATGTTTTACATATGATAGAAAAA
ATGAAGCAATAGCAAAGTATCGTAGTGCGAACCGGTCTCGTAACTTTGATAAGTTTATCAAAGGCGTTAA
CATTTTGTTTCGTAATAGTACCTTAACACATGACGAAGATGAAAATTAAACATGAATTCAAGGAAAAATG
GTAAATGACGAGAAAAAAGC

>Negative Sample 338
CCAAATTCAGTAAAGGGATTGCGAGATCCAAAAAAAAAAGATAAGAGGAAGCGTAGGAAAGGAGAAGCCAA
GACTAATTTGCCAATGTTTGGCGACCAGGATGATGAACGACCTCAGACCGTTAGAGAACGCCATGGCGTA
TTCAGTAAAGAGTTTATTAGTGATTCAGAAGATGACGAGGATTTGATGAACCCTATATTTTTTGAAAACG
AAACATATATGAGATGGTTACTAGATAAAAACAATGGTCAGTTGACCGAGGATAGATATATCCAGTTTGC
CAAATTTGCCGCGGAAAGGA

>Negative Sample 339
GGCTTGTACTTCTGAATTTGGAAATGACCTCGAAAGTTTATCAAATGTGGCCACAGTGGAAAGCTTTTTT
ACTTCAATCTCATCTTCTGTGATACTGTCTATTTTGCCGCGCTTTTTTGAGGGGGAAAAGGCATCATGGC
CTGATAAGTCGTCACTTTCTCTATCATGTATGTCATGTTTTCCTTGTCTTTTCATTAACAGTAAGTTATC
ACCACTTCCCTTTTTTGTGGAATAAACACCTTTTCTCCTCACTCGATTAATTCCCAGACCAGGCCCAACC
ATCTCTGTCTACGCAGGTCA

>Negative Sample 340
GGAGTCTGAAAAAGGGTGGCAATGTCTTCTTGAAAACGACAACGGGAGATTTTGTGAAATTGGGAGTTAA
ATTAACTTCAGGGACACAGGCGATATTGGAGAACACCGAGGAACTATTCGGTGGTGTTGGTTCCAATGGC
AGGGTGTACGATGCATCGAAATTTGGCTCCGCTGATGGTGCAGACAGTGATACTGCAGCTGTATTAGACT

TGGATACTTTATTCGAGGAGGATCAGTTAGTTGGTAGTAAATATTCCAGGATAAGAGATCATGAGCCCAC
GGCAGTAGTCATCGATATGT

>Negative Sample 341
GTTGTTGCTGATAATTTTGAATTTGGTTCAGACAAAAAGGTTATCGTTGCCTTGAAAGACGATGTCTCTC
TATCGAAAGTGAAATCCTTTAAACAGCCAAGCAAAGGAGTCTTGATGCCAAGTGCTTCCCTCCAAGATTT
TTATGGCTAATTCTTATTTTCAAGGTTTTAAAGTTGTATTCCGAGTGTATTATATAGTTGTTTCCCTTCC
GGAAGTTGTGTAATATATAGTTTTGAATATATATAATGGTGTTAAATAGAAATGCCAAATAGTACGTAGT
AATATTATTTAAATTGCATG

>Negative Sample 342
AATCAGTTCAATTATCTATTTGCAGCCATGGCATATGTTGACAAGTTTTATTCAGTATATTTTATTGAGT
CCTTCTTACATCAATGTTTTGAATATCTATGCATTTTGTAATGTCCACGACTTATCATGGGGTACAAAGG
GTGCAATGGCAAATCCGCTGGGTAAGATTAATACTACAGAAGATGGTACGTTCAAAATGGAAGTTCTGGT
CTCTAGTTCAGAGATTCAAGCAAACTACGATAAATATTTGAAAGTTTTAAATGACTTCGATCCAAAATCA
GAATCTCGGCCTACTGAGCC

>Negative Sample 343
AAACAGTACTTGGTTGTCTCGAGCGTTCCTCGCTTGACATAATGATTAAGCAGGGTCTAGAGACTGGGCA
GGTGTATTTGGAATGCAGAAATTGAAATGAAAAGAATGAAATAAAAATAAAAATCCACGAATTGATTAAG
GAACTTATCAAATAATAGAGATCGGTAAAAAGATAGGAATCTAATAAGTGGAAAACTCACTTGAATAATA
AAAGAGTTTTTCTCGATAAGAAAACACAATTCACTTTTACGTTGAACCCCTTTGGTAGTAACTTTTCTGA
GCTTAAAACCACTCCTGAGC

>Negative Sample 344
CTCGGAAAACTTGGTGGATTTTGATGATGTTAACATGGTCGATAAGACCAGACTGTTTATTTTTTTATTT
TTCAGTTTCATCATTACTATACCGTTTATGGTATAAATTAGAAAAGTTAAAGCAGCATGTTTTCATTTGA
AACAAATACTAATGCAATAATAGGTACACCATCTACTACATAATTAATTGATAGTTTCCTTTTCCGTTAA
GTGCATGCATAGGACGCCCATAATTTTTTAAGTTAAAAAAGCATGTATTTACCTATTCGGGAAATTTACA
TGACATGGATGCCATAAGGA

>Negative Sample 345
CTATGATCGAGATGAATATTAAAGTCAAGTCGTTAAAACTTGCGTACCTGTTACTGAATCCGAAAAGAAT
ATTGATATTGAAAAAGAAATCTGAAATCAAAGTGGTGACTAGCCAGATGGTGTTAATTAGAATTAGGATA
TTTAATATCCATTTGGAGTTATGAATAAAGATTCTATAACCTTTGCGAATTTGAAAGAGACTATCTTTGA
TGAACCCATCGGATTCAATGTCACGACCTTCATTAACTGCTGGGCCATTCGCGGATCCTGCACCAAGGGT
CCGGTGGTTGTTGTTCAGCA

>Negative Sample 346
TTCAGCCGATTGCAAATATCTTAAAGGATTTTCCCAAAAAAAAATTTATTTTGGTCGGTGATTCTGGTGA
ACATGACTTAGAAGCGTATACCACTACGGCTTTACAATTTCCGAACCAGATTCTGGCTATTTATATAAGA
TGTTGCTCTAATTCCATGAGTGATGTCCCATCGCATGATGAAGAAGTTATGAATGAAGTGAACAATATCA
TCGAATTACAACAACGGCCCATGCAAATGACCAAGTCTACCGTACGTACCAGGAGAAGACCACCACCACC
TCCCATTCCTTCCACTCAAA

>Negative Sample 347
CTTATGCGAGGGAATCGGGAAGATTGGCTTTGAAGTGTTTTTAGCCTTTTGTTCCTTAGTATGATCTTGA
TTATTGAGCGGTTCAATTCAGTGGAAAAGGACTGCTCTATAGTGTTTTAAGTTTTACAGAAATATAGTGA
TCCTATGAATGTCACTTTTTTGTTTACCTTTAGAAATGCGGAACCATTGGCATTTTTTTTCTTTATTTAC
TAATTTACCTAGAAAATCCAAATGGCACAGGGCGAGCGAATTTTTTTTCCCTCTTTTAATGAGCGAAATC
GAAGATTTTCGGTAATGTTG

>Negative Sample 348
GCAAGTTTCACGTTTCACTCAAAACTTCTCCATGGAAAATATTAAGAGCGAGTTCCAATCCTTGCAATCT
AAATTAGCAACCCTTCGCACCCCGCAAGAATTTTTCAATTTCAAGAAAATTTCCAAACCTCAAAACTTTG
GGGAAGTTCAATCCAGAGTGGCGTATAACCTGAAGTACTTTTCCAGTAACTATGGTTTGATTATTGGTTG
TTTGAGCATTTACACATTATTAACAAACTTGTTGCTACTCTTTGTTATTGTTCTAGTCGTAGCTGGTATT
GTTGGTATAAACAAACTAAA

>Negative Sample 349
TTCATTGAAATAGAAAGACATTTACAAAGTAGACGGGGATATTTAGCCCCACTCATATAAATTATGCATT
ATAACTTTTATAAAAAAAAAGAAATCACACTTAAATAAATGTCTTAATTGCACATTCCATGTTCAGATAT
ACACTATCTTACCTTTTGAATAGGAATGATGGAAAAAAAATTATGCTTCCAATCGGATTTGAACCGATGA
TCTCCACATTACTAGTGTGGCGCCTTACCAACTTGGCCATAGAAGCCCGTTATTACGGTCTGTGATCCAG
CAAATGTCAGATATAACGAA

>Negative Sample 350
CCATTCTAAAATGGTTGCTGAAATTGATATGAAGGAAGCTTTGGCTTCTGGTCAATTCAGGGCTTTAACA
AGAAAAGAGATTGAATTACATTGCGCCATGTTGGGGTTGGATTCAGAATTAGTCTCACATTCAAGAATTA
GAGGTTTATCGGGTGGGCAAAAGGTTAAATTGGTACTCGCTGCCTGCACGTGGCAAAGACCCCATTTGAT
TGTTTTAGATGAACCTACGAATTACTTGGACAGAGATTCATTGGGTGCTCTTTCAAAAGCATTAAAAGCT
TTTGAAGGTGGTGTCATTAT

>Negative Sample 351
CAGATTCTAAGACACCTTGAAATTTTAATTCGTCGTTTTGTTTTACTAATTTGATCAAGTTCTTGTCTAG
CAATTCCTGTACAATGGACATCAAGTCTGTTAACGACCCGATTCCCATTTGTTTTTGGAGTTCTTGCTGT
GTAAATAATGCGCCTATTCCTTTCGACATCATCTGGCTATGTAAGGTTTTAGCATTGTCCGATAGCTGTA
ACCCATTTTCTATCATTCCACTCATATTCAGGATATTGGCGCTGTAAATTCTTCACTTGATTAAGATTTT
CTGTAATGATCTCATCTCAC

>Negative Sample 352
AGAGATTCTGTGAAACTACCGATGTTTGATCTGTTTCTGCTTGGCTGCGCTCCTGACGGTCACATAGCCT
CGTTATTCCCCAATTTTCAAGAGAATCTACGTGAAAATCTGGCATGGGTCATACCTGTAGAAAATGCACC
CAGCGGACCTTCGAATAGGATTTCACTGACCATCCCAGTGATTTGTCACTCTCATAGAGTCACCTTTGTT
GTAGAGGGCGCCACAAAGGCGCCTGTAATCAAGACAATAATGGAAAGGCCTGAAAAGGGATTGCCTAGTA
GTATTGTCAATGAGGGCGCC

>Negative Sample 353
CGTCTCTTTAGTCTGTCCTTCATTGCCATTACTCCACTACTTGCGTCTGGTGGCGGCGTTGGCGTGCTGG
AGGTGTTGAAACTGTTCGACGTTTTACTTCGACCGAGACTTGCCCTGAATTCTTGGCCTGCTGAAGGTCT
AGTCGGCCGTGATGAAGCTCTTCTCGGTGTCGCGCTAGAGGAGCTTGTATGATTATTGTATATATCTTGA
AACGATTTACTCGCAGGCGTGTACTCCGGTGTGTTCCCGTTTGAAGAAGGTGTACCCATAGGATCTTCTG
AAGAAGCCTTCCTTCTGTTA

>Negative Sample 354
AAACAAAAACATTACATGGACAGCGGCTTAGTGATATTCGATAAACAAAAACACTTTTTCTGTCTACCAA
TCGCAATAATGCTGCAATTTTCGCCTATCCAAGAGTTTTTCCATGGTGATAAGGAATGGTTTTGGCTTAG
TTTGTTTATTTCCAAAAAAGAGTTTACTTTTTATCCAATAGAGGCTTCGAATGTAGGAAGATTAGAGAAG
CCGGAGACTCTAGAAAGCAGTACAATCTGTTCTACCCAGCTATCTCACACGGACGTGTACGGTAATCTTT
TGTGGTTGAATGGCGGATTA

>Negative Sample 355
CCAATTCTGTTGTTTGCAGACATTCATTAGACCAAGGTATTACTGACGGGCTATATGCGGTTAGCCGGTC
ATTGTAGTTTCACTTCTAAAGTGTACAGTAACGTTTTGAAAGTTTCCTTTCTCAAATTGTCTATCGTACT
AACATTGCATCAACAAATCGTGTTCGAACCGCCCAAACTCTCACTTCTGCGGTATGGTTTTTTTAGCTTG
GTGTCATAAACTTGTCCATTAGACAAGCAAGGTCATTGCGGATTCAGCTAAATATGGGAAATAAAACAAA
TTTTTAGGGCTAGGGCAATT

>Negative Sample 356
CGCTGGTCATCACCGTCAATCTGCTCCAGCTGAAACTGTTATTTGTGATTACTTCTACACTTCCAAAAAG
CCACTATCAACAATTTGTTTCGAAGGTACTATTGTCGATGTCGGTCCAAAACATTATGCAGGTATTGATA
TGTTACATCGTTACTTGCACGTCCCTACCATGAGTATGGATGGATATGTTGGCGAGTACGCGGAAACTCT
TGAAGAAGTTGTGGACTACACCCAGAACAATGCTACTTACGCAGTTAGAAACACCGACAACTATCTTTAC
TATCTCGCTGACGTTTACAG

>Negative Sample 357
AGGGTACGATACATTGGTGGATGCTTACAGGAGCTATTCCAAGCCAGTTTTTTTTTCAGAATTTGGATGC
AATAAAGTATTGCCAAGGCAATTCCAGGAAATAGGTTATTTGTTTTCTGAGGAGATGTATTCAGTATTCT
GTGGAGGTCTGGTTTACGAATTTTCACAGGAGGATAATAATTATGGGTTAGTTGAATATCAAGAAGATGA

TTCAGTACAGCTCTTAGCAGATTTTGAGAAGCTTAAATCGCACTACCAAAACATTGAGTTTCCTTCTATG
AAGACTTTAAAGGAAACTGT

>Negative Sample 358
ATAAAAGACGATTTTTCAGAAATAAAGTCAAACTCACCTTCCAAACCTATTAGCTGCCCCAGCATCAAAA
GGATAAAGATCGACATTAGGGACTTGAAGATGGATCCGATTTTAAAATCGACACCTGGGGACGATATACA
ATACGTGTGCATCTCAAAACACCTTTGTGGTGTAGCTACAGATCTAACTTTGAGGTGTATAGGCAACAGC
TCAATTTTACATGGCGACGATAACAACGGATGCAACCCCAAACTAAAGGCAATTTGTATTGCCATGTGCT
GCAGGCATGTCTGTGACTAC

>Negative Sample 359
TACTATCGAACCATAGAAACCCATATATTCCCCAATATTAATAATTCTACTGAGAAATGGGTGAATTTTG
AAATAATTGTTGGGATTCCATCGTTGATAAAGGCTATAATATTAGGTATACAGAATGTACTAGAAGTTCT
CCTCGATGATATAGGAATCCCCATAATGGAATCTATATTTCTATGTACCAATATTACGATTATTCCTCAT
TCCATTTCATATGTTTCATTATCCTATTACATTATCGATCCTTGCATTTCAGCTTCCTCTAACTTCGGTG
ACAGCTTCTATAATAACTTA

>Negative Sample 360
CACGTGAATATGTCTATTTTATGTAAACTATAACAGCTGGTACTGCCCGTACATATGCTTAAATTAAATA
GCTATTGACGAAGGCCCATTTTCTCGTAGAATATCAGGGTAACATATGGAATAATCATTTTTCTCTGTCT
ATAAGTAGTAAGACTATTGCTTCTGATCCTTCTGTCAATCGCCTGTCTCCAGTTTTATTTCTTGTCACCT
AGAGAAACATACGCTGGAGTTCCCTGTACTATCAAAAGTTGTTTCGGTCCCGCCATTTCCGACTAGCTCT
ATAGCTAATGGTTGAGACTC

>Negative Sample 361
AATCGAGCTTCGGAAGAACTAAGGGCTTAGTACGAGGTTTGACCAGTTCAATTATTGGAAATCCAGGCAA
GTCGGTACATAAAGTTGGCCCGAAGAATTCACAGTTAACTTCAAGGAAGTTAATTGGGGTATCATCTTTA
TTAGCTTTTTTGGCGTATAGCTCACCAAGCTCTTCGTATACTGGGGCCAGAGTTTTACAATGTTGGCACC
AAGAAGTGTAGTATTTTATCATTGTGTAAGAATCATTCCTATTGCAGATATCGAAGTACTGCTCAATCGA
CTTGACCATCGTGACAGCTT

>Negative Sample 362
CAGGTGCCGAAGCTATCAAGCAAATAAATATATCTGAGGAGCTCAAGGAAAAAGGGGAACGTTTAACAAC
ACCAAACGATCCGTTATTACACGTTGAGGTGAGTAATGAGGATAACTCGCTGCACTTTATATTGTACAAT
AAAACGAATATCATAATTCCAGGAAATTGTACATTTGAATTTTCTAGCCAAATCTCCGAGGTATTCAGTA
TCAAAATGGGACCGCATGAGATAGGCATAAAGGGTCAGAAAGAGCTATGGTTTTTTCCATCATTACCTAC
TCCTCTCTCCAATTACACAA

>Negative Sample 363
ATCATTTAGTGTAATTCATATTTTATATGATATATAGAATAAGCAACATCCCATGAATCAAGCTGATAAT
CCGTTTTGGCAACTGATTACTTCCCAAAGACTCCTTATATTAGGATTGTCTAGACACCCCGGTATTACTC
GAGCCCTAATACAACAGTAAAAGTTTGTAAATATATAGAATTAATTGGTCACAATTGTTTGATGCTCCCC
TTTCAATTAACGAATATCTATTGACTGGTATTTATATTACTATTATATTATCATATAAGACGTGACAAGA
TGACATAAAGACTAAGAAAC

>Negative Sample 364
CTCATGACTAGATAGTTTTATACCAAATACATCATGCATCTTTTTTTTTTCCTTATAGTTTGTGTCATCA
ACATGTTTTTGCCCGTATTAATACACAATAATCTATCCTCTCAGGCAGGGAGAAGAAAACTAAATATACA
TAGCACCCTCACACAGGATGTCAAAACTATCGTCTTATCCTCATGCCGCCGATTTTATAAATATGGAAGA
ACCGCCAAAATCTAAAGAGTTTTTCGATGATCTTTGTGCCGTCCCTAATTTACTCAAAAGACGTTTTCCA
AACTCTCGAAGATCGACGCA

>Negative Sample 365
TGGCGTTAGAGAGTGCGCCCTACATACTTTGAAAAGAGGTTCACCACCACTAGGAGGTGGCGAAGTGCAT
TTGGTTGTTGATTCTTTGATTGCGCAACCTATAACTATGCATGAAATAGATAGGCCCATAATTTCATCGA
TTACCGGTGTAGCATACTCTACCAGAGTAAGTCCGTCGCTTGTGAATAGAATGATCGATGGTGCTAAGAA
GGTATTGAAAAATCTGCAATGCGAAGTTAACATAACGGCAGATGTCTGGAGAGGTGAAAATTCAGGGAAG
AGTCCAGGCTGGGGTATTAC

>Negative Sample 366
CTAAGATAATCCTGAGCATCAGGATGCCAGCAGTTTAGCAATTTGTGCTCTTGACCTCCTATTACTCTAT
TTTTAAGCACGATATCAAAGAAGTATTTTAATTCGACGAAACTCATATTCTCCACACAGTGCAGGAAAGG
TTTGGACTTGACAAGACTCTTAAATCCTCGTCCGGAAGCGAACCTGTCTCCACTCAAACTATCCAGATAG
TGATTGACGTTATCAATTGTAATCGCTTTTGAGCTAGGTTCAGCCCTTCTTTTAGCAATTTCTTCCACAA
GAAGAGAAGAAAGATTCCCA

>Negative Sample 367
TTAGTGGACACGTTTTTCAAAGGAGAACCAAAATCTCTAATACGAACTGGGGTCAACTTCATAAACTCTT
TAGAATTTGATTGATTTACCATGGTCGACACAGTCAACGGTGTTGCTAAAGAAGGGTTCATATGCGGTAT
AACTGTAGTAGACCCAGAATTTTCGCTGTTTCTCCTCGATTTAATGGGCAAAGGGGAAGAAGTTGCTGTC
TCCATTTCAACATCAAAAGTAGTGAATCCAAAATGAAACCTTTGAATTCTAATGCCAGAATGCTATATCT
GATGATTAAAAATAGCCTAT

>Negative Sample 368
TTCGGTTATCCTTTTGAATATATTAATTGCTTTGTATTCGACTGCGTACCAAAAAGTTATTGACAATGCA
GATGACGAGTACATGGCTTTGATGTCACAAAAGACGTTGAGATACATTAGAGCACCTGATGAAGATGTCT
ATGTTTCTCCATTGAACTTAATTGAAGTGTTCATGACACCTATCTTTCGTATTCTTCCACCGAAGCGTGC
TAAAGATTTGAGCTATACTGTAATGACAATAGTGTACAGCCCATTTTTGTTGCTTATTTCTGTTAAAGAA
ACTCGGGAGGCTAGAAGGAT

>Negative Sample 369
TGGATTTTACATCTTATGTTGGAGCCGCTTCAGTTCCCTTCGGTTTGATTCTTTTAGGCGCTACTCTTGG
TAGATTGAAAATTGGAAAATTATACCCTGGTTTCTGGAAATCCGCAGTGGTATTAGTCTTTCTCAGACAA
TGTATCATGCCGATCTTTGGTGTCTTGTGGTGTGACCGTCTAGTGAAAGCGGGATGGCTAAATTGGGAAA
ACGACAAGATGTTATTGTTTGTTACCGCCATTACTTGGAACTTACCAACAATGACCACCTTAATCTACTT
CACTGCAAGTTATACCCCTG

>Negative Sample 370
GCATGGTGGTATTGCTTGCTGTATTAAGTTAGTAATCTCCTTAAACGCGCATTGAGCACAGACTTTCTGG
TAGACCCAATCTTATCTATTTACGAAAACAAGAGTGTATAAGAGTGTATAAGTTATTTTTTGCATTTATT
CTTTTGTGGTAGGGGGGTTTCTTAGCCGCTCTTTTTGACTCAAAATTTCCTTTGATGTCTAGTGATAGTG
CACTTCCCATGTGCGCAACCTGTGTTTTTTGTACTCCGTAGTTTAAATGCAAGGGCTCGATATGCCCTTT
TTCCTTTTTCTACTTTGTAC

>Negative Sample 371
TGTTCCACAGGAACACTTTGGGTAACTGCTATACCATTTTTGATTAAATGCTCTTTTTGAATATATTTGT
CTTGTATCAATCTGATTGTTTCTGGAGAAGGGTAAATTTTTAATTTGGGATGTTTTACTTGAAGATTCTT
TAGTGTAGGAACATCAACATGCTCAATCTCAATCGTTAGCACATCACATTTTTCAGCTAGTTTTTCGATA
TCAAGAGGATTGGAAAAGGAGCCATTAACGTGGTCATTGGAGTTGCTTATTTGTTTGGCAGGAGAATTTT
CAGCATCTAGTATTACCGTC

>Negative Sample 372
TGCTATATCGAAACCAAAAAGAATATTGCAGACGTAATGACCAAACCTCTTCCGATAAAAACATTCAAAC
TATTAACAAACAAATGGATTCATTAGATCTATTACATTATGGGTGGTATGTTGGAATAAAAATCCACTAT
CGTCTATCAACTAATAGTTATATTATCAATATATTATCATATACGGTGTTAAGATGATGACATAAGTTAT
GAGAAGCTGTCATCGAAGTTAGAGGAAGCTGAAGTGCAAGGATTGATAATGTAATAGGATAATGAAACAT
ATAAAACGGAATGAGGAATA

>Negative Sample 373
CAGATTGGGATTTGTCAACCTTGAATATCCCGTTGGATAAAGCTATGAACTTACTTACTGCTTTCAAAAT
ACATTCAGATATATTTGCTAAATGGTCTAATGATAACAACAAGCAGTTAAGAATTATTATGGCTCGCTCT
TATACATTTGTTATCAACATCGCCCTGGAAAGCATATATAGAGCTGTTTTGGAAAAAGTAATCAAATATA
GAACCGAGGTCGAAAACACATGGTTACAACAACTCCAAGACGAATTGAATGGGTCCTATTCGCTCACTGA
CAATGTAAATACTCCAATCG

>Negative Sample 374
TTTATCAAGGAATTTGCTGAGAAAAACGCTTAATATCAGCGGAATATGCCTGTAATTTGGTTCAAAGAAA
AACTTTTTCAAGTCATATGTAAGTTTTAAATAAATCAGTTTACGAACATGCACAAGAATAACTTCTCTTC
TTTTTCTTGAAATAAAGGCCGAATTATACGATATGATATGAGACGTTCTAATTACAATACGATACCGTGA

GATAAAAATAGCGATATCCTCGTACTCAAAGAAGTCTAATTGTTTAGCCCGTTGATCAAAAAAATAAAA
TGTATTTTATTTTTTTGTC

>Negative Sample 375
TTGAGCACAACATCAAATGTACGCAAGCTTATCTGAATACAAAAATATACCAAGAGGGTTTGTCAGAACA
GTTTGTGGACTTATTGGGCGATTATTTTGCTCTGTACTGTAAAAACATTGCATTTCCTGAACTTGTAACA
CCGGTTATTATCTCTTTACGCCGTTACATCAAGACTTCAACTAATGTCAAACTTAACAAACGTTTATCAA
CTGTTGTAGAAAAATTGAACCAAAACAGCACATTCATCCAAGAAAAGAGATCAGATGTTGAATTTGGGCC
AACAAACAAATCTGAAGTTT

>Negative Sample 376
GGACGAATCATCGATGTGACCACTGGTAAAGCTCTTCTTTTCAAGAGTTCGTCTAATACCTCACTGGCCG
GCAAGAGACAGGAAGAAGAGGAAGGTGAATTACATAAATGGGGTGTGTTTGTACAGCACCAAAGCTCAAG
ACATAACTCTGGGCTTCCTTCATCCGCCAATTCTTCGAGAATTAGCGGGTCGTTAACACCAGATAGCAGT
GTCGCCGGAGGAAAGAAAGGGGAATCCTCTCGCACATCTGGTACAAGGCCAAAAATCTTACCAAAGATTC
CTACCGGTGCCGAATTAAGG

>Negative Sample 377
TACAGAAGCCCACAAGAGCATAGTCATAAAGAAAATGCGCTATACCTAATCTCTGGCCAATGGTCAGGTG
TTTCAACAATTATAAAAAAAGACTCGCAAGTTTCACATCAGTTTTACGATTCATCGGAAACTCCTACTGA
ACATTTATTAGTTAAGCCAATCGAAGAACAACATCCTCTGGAAAGTAGGAGGGCATGGAAGGATGTGGCA
GAAGCAATCAGACAAGGAAATATTAGTATGATAAAAAAGACTAAGGAAGAACTAGAAAATAAGCAAAGAG
CCTTGAGAGAACAAGAACGC

>Negative Sample 378
AGAAACATTAAAAAAAATATGCTTTTATACATTGATCAAGCTTAAAAATTGGACGCAACCGGAATCGAAC
CGATGACCTCTTCCTTGCAAGGGAAGCGCGCTACCAACTGCGCCATGTGCCCTGATGACTGTTGAAACTG
TTGTATCTCAAAATGAGATATGTCAGTATGACAATACGTCATCCTGAACGTTCATAAAACACATATGAAA
CAACCTTATAACAAAGCGAACAACATGAGACAAAACCCGTCCTTCCTTAGCTGAACTACCCAAAAGTATA
AATGCCTGAACAATTAGTTT

>Negative Sample 379
TCGAGGCTGTATTCAAATGGCTTGATGCGCTGTGTGTAGATTTTTTTCATAATAAAGGAGAAGGTCCCCC
TCTTGCAGAATTGAAAAATGAGTATTACCAAAATATATTGCTGATCCCAGCTTTTTATGATAAAAGTGTC
TCCGAATTGAAGGATGAAGTTCAGCGATTGATTCCCCTATATGAAGAGTATTTGAGATGGTTTTATCTGA
AGAAAACTCCGATTACTTTTACGAATAAATCCGCTGGAACAATATCTCGAAAAAAGTCGCTAGTAGCTAA
CATTGTAAAAGAGCCTAAGG

>Negative Sample 380
ATAAATTCCTTTATTGTACGGATATTCTGCATTACAAATGAGTCTTCTGCTTTATGCGGATCGTCAGCTT
ATATTGCCTTCGTACAGAAAATGAAAATTCACTACTGGAAACGGGTGAGCGGATGTATGAGTGTATAGTA
TACACCCGGTTTTTTTTACCCTTTCTAAGACCGGAGCTGGCGACAGTAAGTGATAAGCGGCCGTTGGCGG
CCGTGGGTGAGTTATGCATGGGAACCAGCATGAGCCAGCGATGTTCACTATAAAGAGATTAAATGTAGAA
AGTGCTGAGAGACCGATCGA

>Negative Sample 381
TGTAAGTATGACGACGAAAACGAAAAGACTAATAGTTTCATATAATACTAAACGATTTCAATTTTCGTTT
TATATCCTTCCTATTCATAAATTTTATTTCGCTATGCCAATAATGAGTGGATGTATAGTTGATATCTGTT
TCGATAATTTTAAAGTAGATAGTAAAATCTTACTGACATGAATAATCCGTCCAAAACTAGATTACTGTAT
ACAAGCATATTTAAAAAAATAATGAAACTATGATCAAAAAACTGTGCTACTACAGCGGTGTTGTTATCCG
ATACAACCGGATATTTTTCT

>Negative Sample 382
TGCTAGATCCATCAGACACGTTAAACTTTATGTAAAAGAGTATTATAGATTATGTGTATCGAGAAGCAAA
TTGATTTATAATTAGAGGGAATGGAATTGATGTTTAATACCTGCTTACTATATTTTATCTTTGATAATTG
CGCAGAAAGTAATATATCCGGTATTTGAAAATAGGAAATTTAAAGAAATCCCGTCACGCCTGGAATGAGG
GGTGAAACCTTGACTGATATTATATTCTTTTCTTGGACATAGTGTCTGAACCAAATGCAAATTGCACTGC
ATTGCACCAAATTGATGGTT

>Negative Sample 383
ACAATTAGGGGACTTTAAAACCGATACTTGATATATGAGCACAGTCAAGGGGCCGGTTCTTGAGATTTTA
ATGGAAAAGAGGAAAGTTGCACATACAGGTCTGCTTTTCTTTGCTTGCTCCAGACAACGATACTTAGAAC
TAAAACATATTTCTAGAACACTTTTCTCCTTCATTCAAAAAGAAAACTGGCCTTGCAATGACTCCAAAAA
GAGCGCTAATATCTCTTACTTCATACCACGGTCCCTTCTATAAAGATGGTGCGAAAACAGGCGTTTTTGT
AGTTGAGATTTTGCGGTCGT

>Negative Sample 384
CTTCAGGATCAGCAAAGATTTTCAACTACTTTGTTAACGTTGTTTCTATGTTCGGAATCTTGAGTTGGAT
CACCATTTTAATTGTTTACATCTACTTCGATAAAGCCTGCCGTGCTCAAGGGATTGACAAATCAAAATTT
GCTTATGTCGCTCCTGGCCAACGTTATGGTGCTTATTTTGCTTTATTCTTCTGCATTTTGATTGCTTTAA
TCAAAAACTTCACTGTTTTCCTAGGTCATAAATTTGATTATAAAACATTCATCACCGGGTATATTGGCCT
GCCTGTCTATATCATTTCTT

>Negative Sample 385
TCCCAAAAGATAAGCTTATTATTGTACAATTCTTGAGATTCCTGCCTTTTCAAGATCAGATTCTGGTCGA
GTTCAGCCTGCAAGGTTGCCAAAGCGGATTTGTTCTTCTTAATTAAATCAGCGCGTTCACCGTCCTTGGA
GTTCAGATTTTGCGTCAAATCTCTAACCTGTTTGAGATACTTTGCTTGTTTGGATTTTTTCTTATAAAGT
TCAAACCTGGACTTATTGAGATCTCGAAACTTGATGGTTTTGATTTCTCTTTCGCACTCAATCAATTGAG
TCCTGAGAGTATGTCTTTGG

>Negative Sample 386
TCCCTATTATTTTCCTTCCCATCCAATCACTGATAGTTACTACCAGTATCCTCTTCCCAGTAATAATAAC
ACCATCAATTATTTACCATCAGTAGATGTGCAGTATCCTTTGAATGTGAGCCCCTCCTCAACGAGCCATC
CGGCCTCTGAGGTAATCATATCGTCCTTTCCTCCGAGGTCCATGCCAAGTACTTCCTTCAAATATAAAGA
TTCTGCCGACTTTCAAGCACGGACAACTATGAACAAATACAATATTAGACCAAGCAATATCAATGTCAAT
ACTAGTAATATCAATAACCA

>Negative Sample 387
TTGGAAACTTTTTGTCCTATGATTTGTTTTTTTCATTTTATTTTTCATTTTATTTTTCATTTTATTTTCA
TTTAGTTAACATTATTATGCAGTTATTTTTTTTTTTCCCACCTTCATGGCCTCGTTTATGTCTTTTATCA
ATATATTTTTTACGTTTATAAGTCATTTACTCGACTTTTTTTACCTTTGATATGCAATCATATTATTCTT
CCCTCCATTATGGCTAAATAAATGATAATTATAATCTATAGTTTTCAAGCGTTCATATTTTGACAGATAA
GATTATGCAACAACTTGGAA

>Negative Sample 388
GGCTTTCGTTCACTGACGAGTACGGTGACCGGTTGAGATGTTGCCCATCATTAGTGGATTTCGCATTTTG
AACTAAATTTACTGGAAGCGTTAATTTTGGAGTCTTTTGGTGACTCCTATTGGATTCTGGTGGTCTGAAC
ATTGAAGCCATTATCCTGTGTATATAATTGGTATTTTGTAGATATGATAACAGTGCCTGCTATGCTAATC
AGGTAATACTTGCTACTAGTAAAATGTCTAATTTTTTGAGCGAACGAATTTCATGCAAATTAGAAGGTAG
CCAACATATAAATATATGAA

>Negative Sample 389
GATATCATTTTTAAAGACGCAGTTAACAGCGCACCTTCTAATTCTCTACTAAAGGCTACTGTCGAACCAG
CATCCACGTCGACAATTGCTCAAAGAATAATCAGATCTCATAGAAGCAACGCTTCCCCAGAAAAAGAACT
TCATGAAAACAAGCCGAGAAGCACGGAAAAACAGGAACAAAGGGAAATTAGAAGCGATACCAAGGTCAAG
CAGCTCTCGTCAAATAATAGAGCAGCTGAAACCCAAATTCCGTTTTTGCAACAAGAATTTTCCAGTGAGG
ATGAAGATGAAGAATACGTT

>Negative Sample 390
CAAGTCCTTTATGGTATATTGACGCTTCTAGTTGTGGTTCTTTCTGGCTGGCTGACCTACTATCACGTTC
ATGATTCATTTGCAAAGAAAAATCTTTTTATTACTATGGTTATGGGCATGATTCCTTTTGTCATTGGGTT
CATTTGCTGGCAACTAGATATTCACCTGTGTTCTTTTTGGATCTATATCCGGAGAACATATTTGGCCCTG
CCATTAGGTGTTCTATTGGAACTGCATGCTTGGTGGCATCTTTTGACCGGTACTGGTGTCTATATCTTCG
TCGTGTATTTGCAATATTTG

>Negative Sample 391
AAAGAGTTGATCGATTTGGTATTTCCATGGCTGTCTTATTTGAAGCAGGATATGCTTCTTATTGATAGGA
CGAATTACAAGCTTTATTCTCTCTGTGAACTATTTGAATTTATGGGCAGGGTTGCCATTCAGGATCTCCG
ATATCTGAGTCAACATCCCTTATTACTACCCAATATCGTAACATTCATTTCAAAATTATTCCTGAGTTA

TTCCAAAACGAAGAGTTTAAAGGAATCGGTTCAATTAAAAATTCAAACAATAATGCCCTGAACAATGTTA
CAGGAATAGAAACCCAATTT

>Negative Sample 392
CAGAACTTCTTATTCAGTGGTCCTCCCTATCTAGTGCTACTGCACAATCGGAAGACCGAAATAATACGCT
TGACAGCAGAGTACTTGTCTTAGGAGCAACGAATTTGCCATGGGCGATTGATGATGCAGCAAGAAGACGA
TTTTCACGGAAACTATACATTCCTTTACCAGATTATGAAACTAGACTATATCATTTGAAGAGATTAATGG
CCAAGCAGAAAAATAGCCTGCAAGATTTAGACTATGAATTAATAACAGAAATGACCGAGGGGTTTTCTGG
TTCTGATCTTACGTCATTAG

>Negative Sample 393
AAAAATTATCATGGAACATTTACTGATAACGAAGAACAATTCTCAACAGCAGAAGGACTATTCACACGTG
CCATGCAAATTTTTTAAGATGGGCAATTGCCAAGCTGGCTCGTCTTGTCCCTTTTCCCATTCCCCAGATA
TTATCAGTTCTGCGAATAATTTACCTTGCAAGTATTTCGCAAAGGGTAACTGTAAATTCGGTAACAAGTG
TGTTAATGCTCATGTGCTGCCAAATGGGTTCAAGATGAACAGTAAAGAACCTATTGACATCACTCCCCCT
TCACAAAACAACTACTTATC

>Negative Sample 394
ACAATATTTGCTATACTGGTCATCTAGAATAAAAATACTGCGTTGTAGTGCTGAGTGATAGAACTTTTAA
ATCATATTATGTTATATAACAAAGAGCAGGGAACAAGCGGCGCTAGTAGCTCTGGTCGTAGAACGAAATT
CCACTTCGATCGTTTTGTACAAATGGTTCTGTTCATTGCCGCTAATCCCAATTATTGCTGTTCAGTAGCC
AGTATCCCGAAATCTGGTGTCACGCCAGATTTAAAAAGAGCAGATATTCTTGAACAGAAGATCAAAAGCT
TAAATAGTGCGCTGAGTCCA

>Negative Sample 395
GAAATTGCACTTTCGGGCCCAACTATCATCTGAGGAACAGAACCTAAAATACCATAGACAAATGGGCTAA
TAGCCAAAGAATATAGTCCGCATAAAGGTGGGACATGTGCAATTGAAGTTGTGTACGATAGCGCAAGTGG
TATCTGAAATGACGCTACTGAGATACCTGCGATTACGTCGCCCCACAGTTTATTGAAAGTGTACTCAGGA
AGCCATGAAAAGCAGGGCAGGTAATAAGGTAAAGTTTCAAACACTGTATTCTCATCAACGCTATCATTAT
TGTTCGTATTACTTGTATTA

>Negative Sample 396
AGTTCTCTTCTCAATGGTAACAGAATCCATACTACCCTCTGCAATTGGTGGAAAAGCTGATGCCCTAATA
ATCAACAGTTTTTTTTCTGCCTGGCATTCTATTGTAGAAATAATGTTACCTGCATAAATTGGCCTTATAA
AGGTCTTAGGATCTTTGATTACAGTAACCTCACAAACAGGTTGGACGTCCAAGAGCGCACCCACCCGAGG
TAAAACACTTTTTCCAACAGAGGAGTTTGAGACAACAAAATGTGAATAGTCGCCGCCTTTTAATAGTTTC
ACTAATAACGGAGTTAGTTG

>Negative Sample 397
CAAGACTCGTTGGCCATACACCCCTATATAATCCCCTGAGACCGCCTTCGAGTCTGTAAGTTTCGCTTAG
TAATTGCCAGATCCCCGGAGGCTTTGATATACTTTTTGCCTTTGAGCGGTTTAAACTGCTCAAATTTGCT
GTCTGAATCGATAGTCTTGTTTTGATCAAGTCCAACGGGTAAGTAGCCACCACACTACAACCACCACACA
ATGCACCACTGAATAGTCTTTGGGTGTTCGTTAGTTGTTCTTGTCCATTATTGCCATTTACATGGAATAA
CTTTTTCTTACAAGCTTCGT

>Negative Sample 398
AACAGCTTGTCCAGATCCCGAAAGAAGGTTTTTGATATAGTTAAATCGCCCCAAAAATATCTTTTAAAAA
TCAATGTACTGGATATCTTTATAAAATCTGAACAATTCAAGGCTCTTGAAAGATTGGTTACTGAACCTAA
ATTATTGGTTATTTTAGAAAAGTGTAAACATTGTGATTTTGATACTGTCCAAATTTTTAAGGAGGAAATT
ATGCATTTTTTTGAGGTTTTGGAAACTCTACCCGGTGCATCAAGAATACTCCAAACTGTCTCATCAAAGG
CAAAAGAGCAAGTAACAAAT

>Negative Sample 399
TTTAAAGTCGCACCTGTAATAACTTGACAGTACATAGGAAGGTATGAAAAAATCGGGCAATCTCCCTCTA
AATTATACTGTTTTGTTTCAGAATTTAAAATATAAGTATCTGTTGGTTTATCATTGGTGGGCACCTCACT
GTTATTAATACAGTCCATATTATTTTCGAGCGCTTGTGAGTGATTCAGATAATAATTATAGAGATTCTCT
CTAGTGGTGAATCTCTGAAGATCTTGCGATGGATTGTAGTCGATTTTTTCGTTCCAATTATCGGCCAATT
CACTCAACTCACTCCATTTG

>Negative Sample 400
ATTTTGTTTGCTAAAGAGGTAACTATGTAGAGTTGCTTGGGATGAGCTTCCTTCTCTGTTGTACTTCAGT
TTGATCTGAAACTTAAAAAACGAAATTTAAAAGGAAGACAACGGTGAGAACAAGAAATGAATGAATCAAG
AAGTCACGATCAAGTATGTAGGTAAAAGATCAGGGGGGAGTATGTACGTAACCAAAAAGATTGGGAAAGA
GTTTATATATGAATCATGCCTACGATCGTACCTTCTTGGCTTTCCCAAGAAAATTTTTCATCTCTCGAAC
TTCTCGAGAGATTGCGAACG

>Negative Sample 401
TTGAAAGTTTGGTTGGATATACACCTTTTAGTGGTTCTTCTACTAATGAAACTTACGAGAATTTAAGATA
TTGGAAAAAAACTTTAAGAAGGCCCCGTACTGAGGATAGAAGAGCAGCATTCTCAGATAGAACCTGGGAT
TTAATTACAAGACTAATAGCTGACCCCATCAACAGAGTACGATCTTTTGAGCAAGTTCGCAAAATGTCTT
ATTTCGCAGAGATCAATTTTGAAACCCTAAGAACAAGTTCTCCACCATTTATTCCTCAATTAGATGATGA
AACGGATGCAGGTTATTTCG

>Negative Sample 402
GAATTTCTAGTATAATCTGTATACATAATATTATAGGCTTTACCAACAATGGAATTTCGACAATTATCAT
ATTATTCACCAATTAATCACAAGTTGGTAATGAGTTTGATAACAAGTTACTTTCTTAACAACGTTAGTAT
CGTCAAAACACTCGGTTTTACTCGAGCTTGTAGCACAATAATACCGTGTAGAGTTCTGTATTGTTCTTCT
TAGTGCTTGTATATGCTCATCCCGACCTTCCATTTTTTTTTCTTGGAATCAGTACATAGCAGGTATGAG
TTGTTAGAGCTGTTACAAGT

>Negative Sample 403
AGAGGACGACGACTTCCCAAGAAAGAAAAAATCGAAAAACAGCAAACATGATGATGGTTCTACTGGATTT
TCTGCTGCAGTAAATGCTATTTTATCATCACATCTGAAAGCATACGACAGGAAGGACCCCATCATGGCTA
GAAACAAAAAGGTGCTGAAGCAAAGTGAGTCAGAAAAGCTAGAATACAAGGCCAAAAAAGCATTGTTGGC
AGAAAAAAAAAAGTTATTAGGTAAAGCCAGAAAGACGGATATTATTCCAATTGCTTCCGGAGAAGACAGG
TCAGAAAATATTAGAAAAGT

>Negative Sample 404
CGGAAAAGAAAACAGCAAGATAGTGACGATGGTGGGGATACTATACTGGAAAGACCGTTGATTTTTAGTG
CTAGAGGCACGCATGCTAATTATGCATCCGCTGGGCAGCATGCTCATGACATTCCTTTTTTTTTCATGCC
TTTGAGTGATTTTACCGATCGTGGTCCCCTGTGGGATCCATCTTTGAACTTTTACTCCTATACTTTTGAT
GGTAAAACTGTGACACCCTCCTCAGAGAGAGAAGAATCTCTCGGTTTGGATTGGTTGCATTTTCAAGGTG
GATGGGGTGATCAACAACTA

>Negative Sample 405
AACTGTCTCTTTCGAGAAAAAGAAAGAGAAAAGAAATGGTCTCTAGCGGGATCGAACCGCTGATCCCCGC
GTTATTAGCACGGTGCCTTAACCAACTGGGCCAAGAGACCATTATATTGTTAAAGATGTTGTATCTCAAA
ATGGGATACGTCAGTATGACAATACTTCATCCTAAACGTTCATAAAACACATATGAAACAACTTTATAAC
AAAACGAACAACATGAGGTAAAACCCGGCCTCCCCTAGCTGAACAACTCAAACGTATAAATGCCTGAACA
ATTAGTTTAGATCCGAGTTT

>Negative Sample 406
CTCTAGCATGGAAGTTGGTACTAAATAAGAAGGATATGCGGATGAATTTTTTTTCGAATACTAACGCCAC
ACACATTCCAGTCGAAATAGCCAAGGATATGTTACAGGATACACTTTTAACTCCAATCGACCTATATGAT
GTGCATGGTCCTGTGATACCAATGAAGGCGCTAGAAATAGCCAATGCATTGGTAGACGTCGTAAGTAAGT
ATGATCACAATATGAAGTTGGAGGCTTGGAATATTTTGTGCGATGTATCTAAGTTCGTTTTCTCCCTGAA
ACATTGCAATCATAAAATGT
